# The complete genome of *Trypanosoma cruzi* reveals 32 chromosomes and three genomic compartments

**DOI:** 10.1186/s12864-025-12482-0

**Published:** 2026-01-08

**Authors:** G. Greif, M.L. Chiribao, F. Díaz-Viraqué, Carlos E. Sanz-Rodríguez, C. Robello

**Affiliations:** 1 Laboratorio de Interacciones Hospedero-patógeno — UBM, Institut Pasteur de Montevideo, Montevideo, Uruguay; 2 Unidad Académica de Bioquímica, Facultad de Medicina, Facultad de Medicina, Universidad de la República, Montevideo, Uruguay

**Keywords:** *Trypanosoma cruzi*, Chromosome complement, Karyotype, TriTryps, Telomere-to-Telomere

## Abstract

*Trypanosoma cruzi*, the causative agent of Chagas disease, exhibits remarkable genomic variability and possesses an expanded genome rich in multigene families. However, its precise chromosomal composition has remained elusive due to the challenges posed by extensive repetitive regions. In this work, we determined the complete molecular karyotype of the *T. cruzi* Dm28c strain, which comprises 32 chromosomes. Comparison with two independently isolated Clade A (TcI) strains revealed clear chromosomal homology, indicating that the 32-chromosome karyotype is stable, at least among this clade. *T. cruzi* is diploid for all chromosomes, except chromosome 16, which is consistently tetrasomic in all strains analyzed. This tetrasomic chromosome is homologous to chromosome 31 of *Leishmania major*, which is also tetrasomic.

Through a comprehensive annotation pipeline, we refined gene content and resolved haplotypes. Comparative analysis of chromosomal architecture revealed that all chromosomes exhibit a conserved distribution of core and disruptive compartments, along with notable conservation in GC content transitions. These findings demonstrate a high degree of structural conservation, challenging the prevailing paradigm of extensive chromosomal rearrangements and high genomic plasticity in *T. cruzi*.

Subtelomeres—enriched in *RHS*, *DGF-1*, and *TS* genes, and depleted in *MASP* and mucins—constitute a distinct third genomic compartment that is transcriptionally active and represents the primary source of interstrain genomic variability. Furthermore, a reclassification of the TS gene family revealed differential distribution patterns across subtelomeric and disruptive compartments.

Altogether, this work defines the complete chromosomal complement of *T. cruzi*, establishing a robust framework for comparative genomics and enabling detailed investigations into genome organization, antigenic variability, and evolutionary dynamics across strains and clades, opening new avenues for exploring *T. cruzi* biology, pathogenic diversity, and adaptive mechanisms.

**Supplementary Information:**

The online version contains supplementary material available at 10.1186/s12864-025-12482-0.

## Background

*Trypanosoma cruzi*, the causative agent of Chagas disease, belongs to the family Trypanosomatidae, which consists exclusively of parasitic organisms. Three of these, known as the “TriTryps”*—Leishmania spp.*, *Trypanosoma brucei*, and *T. cruzi*—pose significant challenges to human and animal health and have been extensively studied. They share several biological features, including RNA editing, polycistronic transcription, a single mitochondrion with kinetoplast DNA (kDNA), *trans*-splicing, and the absence of chromosomal condensation during mitosis. Additionally, each of these species possesses unique characteristics. In *T. cruzi*, the most notable feature is its genome expansion, primarily driven by the presence of multigene families, most of which encode surface GPI-anchored proteins. These include trans-sialidases (TS) [1–6], mucins (MUC) [7–10], mucin-associated surface proteins (MASP) [11–13], GP63 [14, 15], disperse gene family 1 (DGF-1) [16, 17], and retrotransposon hot spot proteins (RHS) [18, 19]. This expansion in *T. cruzi* represents an evolutionary acquisition linked to antigenic variability, invasion, infectivity, and immune system evasion, among other factors.

Regarding the karyotype of *T. cruzi*, the absence of chromosomal condensation during mitosis has impeded the use of classical cytogenetic studies. Instead, pulsed-field gel electrophoresis (PFGE) emerged decades ago as a valuable tool for determining their karyotypes. Using this approach, it was established that *T. brucei* has 11 chromosomes [20, 21], *Leishmania major*, *L. infantum*, and *L. donovani* each have 36 [22], and *L. mexicana* and *L. braziliensis* contain 34 and 35 chromosomes, respectively [23]. However, the exact chromosome number of *T. cruzi* remains unknown. PFGE studies consistently indicate that chromosomal sizes vary among different strains, ranging from 0.5 to 3 Mb, and estimate that it may have between 20 and 40 chromosomes [24–30]. The challenge in determining its precise number, compared to *Leishmania* and *T. brucei*, arises from several factors. First, *T. cruzi* shows significant variability in chromosomal sizes across strains, which limits the exact resolution of well-defined bands in PFGE. Additionally, its genome exhibits extreme plasticity, characterized by multigene families, repetitive elements, and retroelements [31]. Furthermore, homologous chromosome pairs often differ in size, resulting in a complex, unresolved banding pattern.

After completing the genome projects for these three organisms - the “TriTryps” - many results obtained through PFGE were confirmed for *L. major* [32] and *T. brucei* [21]. These studies also established a comparative chromosomal map between these two species, revealing regions of high synteny and providing insights into their common ancestor [33]. However, *T. cruzi* was not included in this comparison because the scaffolds obtained were highly fragmented due to its repetitive genome, leading to a high incidence of regional collapse [34]. Consequently, although syntenic regions were identified as shared among the TriTryps, chromosomal mappings systematically excluded *T. cruzi*. Later, studies based on BAC-end sequencing combined with chromosomal co-location and synteny with *T. brucei* and *Leishmania*, enabled the assembly into 41 in silico chromosomes [35]. Although this estimation was derived from genome sequences prone to collapse due to repetitive regions, it has served as a valuable reference for subsequent studies.

The advent of long-read sequencing marked a significant advancement in studying complex genomes, such as *T. cruzi*, which contain numerous repetitive elements and multicopy genes that are highly similar or even identical and arranged in tandem. The use of PacBio or Nanopore methodologies enabled researchers to determine a more complete genome landscape by overcoming repetitive regions and generating scaffolds that, in some cases, have lengths compatible with the expected size of chromosomes [36–40]. These studies provided a comprehensive view of genome organization and opened the door for comparative genomic analyses among the TriTryps. For instance, it was determined that multigene families encoding surface proteins are not located in subtelomeric regions, as previously proposed, but clustered along different chromosomes. In this regard, a key discovery was the identification of two distinct genomic compartments in *T. cruzi*: the core and disruptive regions [36], the latter being non-syntenic with other trypanosomatids and housing hundreds of gene variants encoding surface proteins such as TS, mucins, and MASP. Furthermore, recent findings have shown that these genomic compartments correlate with specific three-dimensional chromatin organizations designated as C and D, which represent lower and higher levels of chromatin compaction, respectively, and influence global gene expression [41, 42].

However, the lack of telomeric sequences at their ends prevents them from being classified as chromosomes. The sole exception is the genome of the Dm25 strain, which consists of 24 scaffolds with telomeres at both ends, confirming their identity as complete chromosomes [43]. This significant advancement is primarily attributed to PacBio HiFi technology. Considering these precedents, we aimed to determine the complete karyotype of *T. cruzi*, elucidate the chromosomal organization of its core and disruptive compartments, and characterize the structural features of its telomeric and subtelomeric regions.

## Results

### Updated web platform for genome visualization

In our previous study, we developed an accessible online platform to explore *T. cruzi* genomic data (ref. 36). In this work, we expanded this resource to include the Dm28c telomere-to-telomere (T2T) genome, along with both fully resolved haplotypes, which are accessible through the web platform: https://cruzi.pasteur.uy. The updated platform maintains the original color scheme with minor modifications: RHS genes are shown in brown, TS in orange-red, DGF-1 in red, mucins and MASP in shades of blue, GP63 in orange, conserved genes in green, and pseudogenes in magenta (Supplementary Fig. 1). Chromosome orientation is displayed from left to right as 5′ to 3′, following the same convention in all figures and descriptions involving chromosomal organization, directional gene clusters (DGCs), telomeric and subtelomeric features, and comparative analyses.

### Chromosome-level assembly defines the 32-chromosome karyotype of *T. cruzi*

**Fig. 1:**
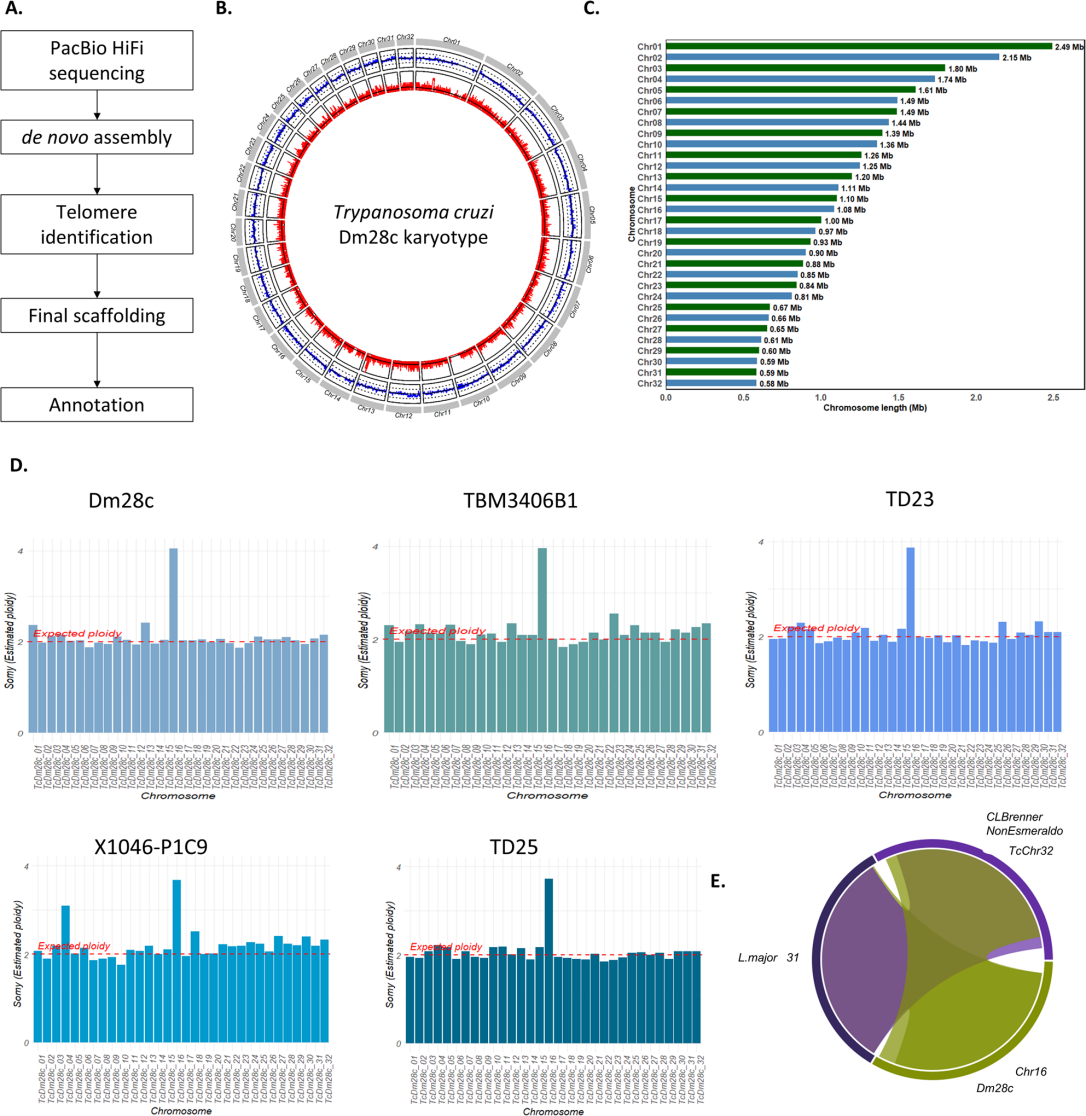
The karyotype of *T. cruzi*. **A** Assembly pipeline. **B** Circos plot karyotype representation. The outer grey lines represent chromosomes; the middle circle represents the percentage of GC content along chromosomes (the black line indicates 50% GC, and the dotted black lines indicate 25% and 75% GC content). The inner circle in red indicates gene density calculated using overlapping windows of 5000 bases, sliding by 500 bases. **C** Bar charts showing the *T. cruzi* chromosome lengths in Mb. **D** Ploidy analysis for Dm28c, TBM3406B1, TD23, X1046-P1C9, and TD25 Clade A strains. **E** Syntenic regions (Symap representation) between *T. cruzi* chromosome 16, chromosome 31, *L. major* and *T. cruzi* CL Brenner strain

The reference strain Dm28c was selected for this study due to its extensive characterization of cell cycle dynamics [44] and prior genomic studies [36, 42, 45]. Total genomic DNA was sequenced using PacBio HiFi technology, generating 301,047 reads, 2.93 Gb of sequenced bases, and an average read quality of Q29 (Table 1). The assembly was performed with the HiFiAsm algorithm [46], resulting in an estimated genome size of 36.18 Mb with approximately 80× coverage. The assembly comprises 32 scaffolds, with an average GC content of 51.06% (Table 1). Analysis of scaffold ends revealed the presence of telomeric repeats (CCCTAA/TTAGGG) [47] at both ends of all 32 scaffolds, immediately adjacent to the conserved 189 bp junction sequence [48], indicating that each scaffold represents a complete chromosome. Chromosomes were numbered sequentially from 1 to 32 in order of decreasing size (Fig. 1A and B), and chromosome lengths range from 2.49 Mb to 0.58 Mb (Fig. 1C; Tables 1 and 2). A complete step-by-step description of the assembly process is provided in the Materials and Methods section.

**Table 1:** Sequencing, genome assembly statistics. Final genome assembly features and annotation

Elements	Metric	Value
Sequencing Statistics	Number of reads	301047
	Number of bases sequenced	2.92 Gb
	N50	10.68 Kb
	Average read quality	Q29
Genome Assembly statistics	Number of contigs	51
	Total length of assembly	46.64 Mb
	G+C content	51.35%
	Average coverage HiFi reads	62.7 X
	N50	1.2 Mb
Genome	Number of chromosomes	32
	Genome length	36.18 Mb
	Longest chromosome	2.49 Mb
	Shortest chromosome	0.58 Mb
	Average coverage HiFi reads	80.80 X
	N50	1.26 Mb
	G+C content	51.06%
Number of genes	Protein coding genes	13766
	RHS	683
	Trans-sialidases	1126
	Mucin	381
	MASP	530
	DGF-1	248
	Transposable elements	1875
	Putative pseudogenes	1781

**Table 2:** Chromosome classification by structure. Choromosome name, length in bases, core, disruptive and GC percentages and chromosome classfication were included. Dm25 and Sylvio homologs chromosomes and their classification

Chromosome	Length (bases)	Core Percentage	DisruptivePercentage	GC Percentage	Chromosome Classification	Dm25	Length (bases)	DisruptivePercentage	Chromosome Classification	Sylvio	Length (bases)	DisruptivePercentage	Chromosome Classification
Chr 01	2492231	68.38	31.62	49.89	Core	Chr04*	2852776	34.35	Core	JBMETK010000004.1	2070488	26.32	Core
Chr 02	2151424	77.86	22.14	49.01	Core	Chr02	2145383	21.24	Core	JBMETK010000002.1	2204867	24.63	Core
Chr 03	1802204	13.99	86.01	53.13	Disruptive	Chr32	1869386	85.55	Disruptive	JBMETK010000031.1	2110958	87.12	Disruptive
Chr 04	1735261	29.17	70.83	52.02	Disruptive	Chr30*	2497974	75.25	Disruptive	JBMETK010000029.1	2632980	78.82	Disruptive
Chr 05	1610701	77.20	22.80	49.02	Core	Chr03	1882095	34.62	Core	JBMETK010000003.1	1638490	23.51	Core
Chr 06	1492875	34.07	65.93	52.02	Disruptive	Chr09	1349720	68.79	Disruptive	JBMETK010000009.1	1425371	68.13	Disruptive
Chr 07	1490547	27.31	72.69	52.70	Disruptive	Chr06	1647762	79.15	Disruptive	JBMETK010000006.1	1182010	72.13	Disruptive
Chr 08	1436163	63.69	36.31	49.97	Core	Chr07	1458509	39.94	Core	JBMETK010000007.1	1423805	39.51	Core
Chr 09	1394721	58.36	41.64	49.99	Mixed	Chr05	1362421	43.82	Mixed	JBMETK010000005.1	1374848	44.78	Mixed
Chr 10	1361090	31.69	68.31	51.26	Disruptive	Chr22*	900376	48.39	Mixed^#^	JBMETK010000022.1	1381918	59.40	Mixed^#^
Chr 11	1261511	59.67	40.33	50.04	Mixed	Chr08	1228545	40.98	Mixed	JBMETK010000008.1	1284254	41.21	Mixed
Chr 12	1250845	33.92	66.08	53.50	Disruptive	Chr17	1222364	63.31	Disruptive	JBMETK010000017.1	1129116	64.35	Disruptive
Chr 13	1200139	61.10	38.90	50.28	Core	Chr16*	1308678	28.92	Core	JBMETK010000016.1	1479766	35.03	Core
Chr 14	1111135	41.50	58.50	51.91	Mixed	Chr11*	962143	65.69	Disruptive^#^	JBMETK010000011.1	1247523	71.28	Disruptive^#^
Chr 15	1100981	80.11	19.89	48.54	Core	Chr10	1139640	23.09	Core	JBMETK010000010.1	1349194	30.85	Core
Chr 16	1084488	25.26	74.74	52.74	Disruptive	Chr31*	1639103	53.22	Mixed^#^	JBMETK010000030.1	1183316	74.70	Disruptive
Chr 17	1002814	41.19	58.81	51.78	Mixed	Chr18	1022790	60.28	Disruptive^#^	JBMETK010000018.1	1036108	60.75	Disruptive^#^
Chr 18	965874	63.91	36.09	50.26	Core	Chr14	962143	35.45	Core	JBMETK010000014.1	1067752	44.34	Mixed^#^
Chr 19	933234	80.62	19.38	48.87	Core	Chr15	944107	21.04	Core	JBMETK010000015.1	921364	19.64	Core
Chr 20	903723	37.92	62.08	51.26	Disruptive	Chr13	811800	54.36	Mixed*	JBMETK010000013.1	839773	61.95	Disruptive
Chr 21	883590	40.55	59.45	51.46	Mixed	Chr21*	902719	62.25	Disruptive^#^	JBMETK010000021.1	938715	61.65	Disruptive^#^
Chr 22	849045	48.91	51.09	50.55	Mixed	Chr19	967695	42.75	Mixed	JBMETK010000019.1	914153	41.74	Mixed
Chr 23	843191	18.27	81.73	53.26	Disruptive	Chr12	1140931	87.53	Disruptive	JBMETK010000028.1	641127	77.31	Disruptive
Chr 24	811364	41.06	58.94	51.77	Mixed	Chr24*	893315	59.87	Mixed	JBMETK010000024.1	843343	58.66	Mixed
Chr 25	671853	31.70	68.30	52.89	Disruptive	Chr20	779959	67.35	Disruptive	JBMETK010000020.1	606623	68.40	Disruptive
Chr 26	663682	19.99	80.01	53.10	Disruptive	Chr23	637003	77.14	Disruptive	JBMETK010000023.1	703263	81.14	Disruptive
Chr 27	653362	48.21	51.79	51.29	Mixed	Chr25	719266	56.16	Mixed	JBMETK010000025.1	653302	47.16	Mixed
Chr 28	614808	55.64	44.36	50.90	Mixed	Chr28	594674	44.91	Mixed	Chromosome missing in Sylvio Assembly			
Chr 29	599670	41.27	58.73	51.65	Mixed	Chr01	752486	63.63	Disruptive^#^	JBMETK010000001.1	897124	53.15	Mixed
Chr 30	588781	17.35	82.65	54.24	Disruptive	Chr29	566767	76.91	Disruptive	JBMETK010000012.1	694675	74.11	Disruptive
Chr 31	585579	48.00	52.00	51.44	Mixed	Chr27	628314	57.44	Mixed	JBMETK010000027.1	627031	57.75	Mixed
Chr 32	584768	70.45	29.55	49.73	Core	Chr26	769906	48.12	Mixed^#^	JBMETK010000026.1	582340	30.50	Core

Classification: %Core > 60% =Core. % Disrutpive> 60%=Disruptive. Otherwise=Mixed

^#^Not equal classified

*Not T2T in Dm25

We then compared the Dm28c karyotype with the PacBio HiFi–sequenced genomes of the Dm25 and Sylvio X10 strains (PRJNA1039288 and PRJNA1237338). For Dm25, each Dm28c chromosome had a matching homolog in that assembly. Although 24 of 32 Dm25 chromosomes are assembled telomere-to-telomere, the remaining 8 showed a clear one-to-one correspondence with the Dm28c karyotype. As additional confirmation of the completeness of the Dm28c karyotype, we compared the chromosomes with those of the Sylvio X10 strain, and again found a clear correspondence, except for chromosome 28 (Dm28c.Ch28; 0,615 Mb; Table 2), which was absent in the Sylvio X10 assembly. This result can be explained either by chromosomal translocations that redistributed its sequences to other chromosomes or by an incomplete assembly of the Sylvio X10 genome, since no regions of Dm28c.Ch28 were detected on any other chromosomes; we back-mapped from a prior Sylvio X10 strain assembly (ADWP00000000.2) and Sylvio X10 RNA-seq reads (SRR9202394) onto the Dm28cT2T genome, and both aligned to Dm28c.Ch28. Moreover, among the Sylvio X10 RNA-seq reads that mapped to Dm28c.Ch28 (*n* = 270,804), more than 90% (*n* = 250,370; 92.45%) did not align to the Sylvio X10 genome (JBMETK000000000), confirming that chromosome 28 is missing from the Sylvio X10 genome assembly (Supplementary Fig. 2). We also identified a duplicated and inverted region at the beginning of one Sylvio X10 chromosome, likely representing an assembly artifact, as detailed in the Materials and Methods section.

Overall, these results provide a clear resolution of the *T. cruzi* Dm28c strain karyotype, confirming that its genome comprises 32 complete chromosomes. This discovery marks a major milestone in T. *cruzi* genomics, finally yielding the first T2T genome of a member of Clade A (TcI) and demonstrating the conservation of the karyotype in at least two independent strains. clarifying the parasite’s chromosomal composition, a question that has remained unanswered until now.

### Genome annotation

The annotation pipeline (Supplementary Fig. 3 A) enabled the re-annotation of the Dm28c genome, uncovering a haploid gene content of 13,766 protein-coding genes, including 1,781 putative pseudogenes (Table 1). Approximately 22% of these genes belong to the most expanded multigene families: TS, mucins, MASP, RHS, GP63, and DGF-1 (Table 1). Compared to our previous annotation of Dm28c [36], the most significant difference is a ~ 25% reduction in the number of core genes, from 12,230 to 9,916 (Supplementary Table 1). This reduction results from methodological differences: earlier assemblies were unable to fully resolve haplotypes. Consequently, genes in regions where both haplotypes were distinguishable were counted twice, whereas those in collapsed regions were counted only once. In contrast, the current annotation, based on PacBio HiFi sequencing, benefits from improved haplotype resolution. Gene counts for other multigene families varied, with some increasing and others decreasing (Supplementary Table 1), reflecting the enhanced continuity and resolution of repetitive regions (e.g., tandem, direct, and inverted repeats) and the more accurate gene copy number estimation enabled by haplotype phasing.

For multigene families, we performed manual curation based on sequence alignments, cluster analysis, and the evaluation of coding sequence completeness. Although a detailed analysis exceeds the scope of this work, iIt is worth noting that in one case, this process led to the identification of a previously unrecognized clade within the TS family. Further sequence analysis revealed that this clade corresponds to the retroelement L1Tc, which had been misannotated as TS in UniProt (https://www.uniprot.org). As a result, 59 coding sequences originally classified as TS were removed from the annotation because they are part of the L1Tc retrotransposon family (Supplementary Fig. 4). This finding underscores the need for comprehensive manual curation of the entire genome annotation and for revising misclassified proteins in public repositories.

### *T. cruzi* exhibits a diploid genome and a conserved tetrasomy of chromosome 16

To determine the ploidy of *T. cruzi*, Illumina sequencing was performed, and the reads were mapped to the 32 chromosomes. The analysis showed a diploid state across all chromosomes except for chromosome 16, which exhibited tetrasomy (Fig. 1D). Analysis of haplotype coverage confirms tetrasomy of the chromosome (Supplementary Fig. 5).To assess whether this pattern is specific to the Dm28c strain or conserved across Clade A (TcI) lineages, we mapped available Illumina datasets from additional Clade A strains. Strain TBM3406B1 (Ecuador; SRA: SRR3676268), X10462-P1C9 (Venezuela; SRA: SRR3676274), TD25 (Texas; SRA: SRA3676273), and TD23 (Texas; SRA: SRR3676272) show the same pattern of diploidy across all chromosomes, except for chromosome 16, which consistently exhibits a clear pattern of tetrasomy (Fig. 1D). Given that tetrasomy of a single chromosome has been reported previously in *Leishmania spp.* and in the *T. cruzi* CL Brener strain [49–51], we aimed to determine whether these chromosomes are homologous to Dm28c.Chr16, through a synteny analysis. Strikingly, *L. major* chromosome 31 was found to be homologous to chromosome 16 in our assembly and to the tetrasomic pseudochromosome 31 of the *T. cruzi* CL Brener strain [51] (Fig. 1E). Together, these findings suggest that the tetrasomic state of this chromosome reflects an ancestral tetrasomy ploidy conserved among trypanosomatids. It is worth noting that the X10462-P1C9 strain, in addition to tetrasomy of chromosome 16, also shows trisomy of chromosome 4, indicating strain-specific variation in aneuploidy. Future studies across all clades will be necessary to fully characterize the *T. cruzi* pangenome.

### Conservation of chromosome-architecture in *T. cruzi*

**Fig. 2:**
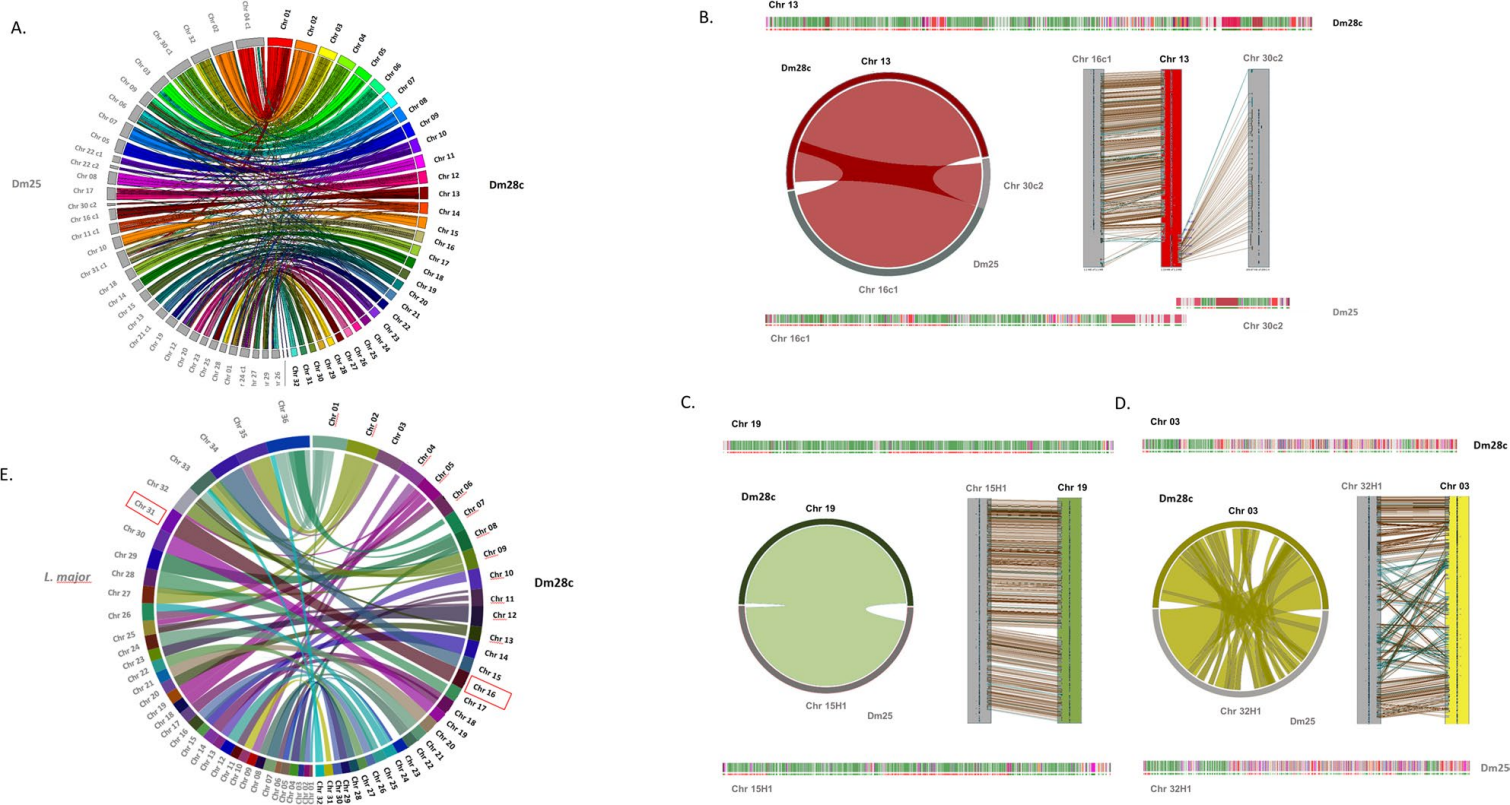
Comparison of the Dm28c and Dm25 genomes.** A** The 32 chromosomes of *Dm28c* (colored) were mapped against all contigs present in haplotype 1 of *Dm25* (in gray). All contigs in the Dm25 assembly are represented in the 32 chromosomes of the Dm28c strain. **B** The fragmented chromosome in Dm25 is assembled into the T2T chromosome in Dm28c. At the top (Dm28c) and bottom (Dm25) are schematic representations of chromosomes retrieved from the web page. In the middle, Symap results: Left panel: circos synteny plot; Right panel: synteny blocks. **C** Example of synteny in the core chromosome and **D** in the disruptive chromosome. At the top and bottom are schematic representations of chromosomes. In the middle, the results of synteny blocks obtained with Symap are represented by a circos plot and chromosomal representation, like **C.** In all figures, gene representation colors align with the web interface’s color code (https://cruzi.pasteur.uy/): RHS (brown), TS (orange-red), DGF-1 (red), Mucin and MASP (shades of blue), GP63 (orange), Conserved genes (green), and Pseudogenes (magenta). **E** Circos representation of syntenic regions between the Dm28c, CLBrener, and *L. major* determined with Symap. The red square shows the proposed conservation of aneuploidy in trypanosomatids

It is widely accepted that *T. cruzi* exhibits significant genomic plasticity and frequent chromosome rearrangements [52–54]. To determine whether the chromosomal architecture is specific to the analyzed strain or a broader characteristic across the species, we used the Dm28cT2T karyotype as a reference and completed the assembly of Dm25. We found that Dm25 also has 32 chromosomes, ranging from 0.77 to 2.85 Mbp (Fig. 2A; Table 2, and https://cruzi.pasteur.uy*).* It is important to note that, although their names are similar, Dm28c and Dm25 are independent isolates obtained from different regions and at different times [43, 44]. Besides having the same number of chromosomes, the two genomes also exhibited high degree of chromosomal structural conservation (Fig. 2A), with synteny and structural features confirming their homology (Figs. 2B–D and Supplementary Fig. 5). In Fig. 2B, we show an example of Dm25 karyotype reconstruction: Dm28c.Ch13 maps to two separate pseudochromosomes in Dm25 (contig c1 of pseudochromosome 16 and contig c2 of pseudochromosome 30), yet maintaining a highly conserved overall structure (Fig. 2B). In Fig. 2C and D, we present two representative examples of complete Dm25 chromosomes: a core chromosome and a disruptive chromosome, respectively. In both cases, each Dm28c chromosome has a corresponding homolog in Dm25, and this is consistently observed across all chromosomes (Supplementary Fig. 5). Synteny is well preserved in the core-enriched chromosomes (Fig. 2C and Supplementary Fig. 6), whereas the disruptive chromosomes exhibit local differences—likely caused by gene duplications or rearrangements—while retaining their overall structure (Fig. 2D and Supplementary Fig. 56). As expected, this conservation results in highly similar chromosome sizes. In T2T chromosomes from both strains, size differences are largely confined to telomeric and subtelomeric regions, while the largest discrepancies occur in chromosomes incomplete in Dm25. This high level of conservation across strains strongly suggests that a chromosome complement of 32 chromosomes is a defining feature of the *T. cruzi* species, at least for Clade Aa (TcI). This conservation prompted us to ask whether it extends across species, particularly given the remarkable similarity observed between Dm28c.Ch16 and L. major chromosome 31. We therefore broadened our comparative analysis to include all L. major chromosomes. This revealed a striking pattern: each core chromosome of *T. cruzi* has a homologous counterpart in *L. major*, suggesting that the core chromosomal set is deeply conserved across trypanosomatids (Fig. 2E).

**Fig. 3:**
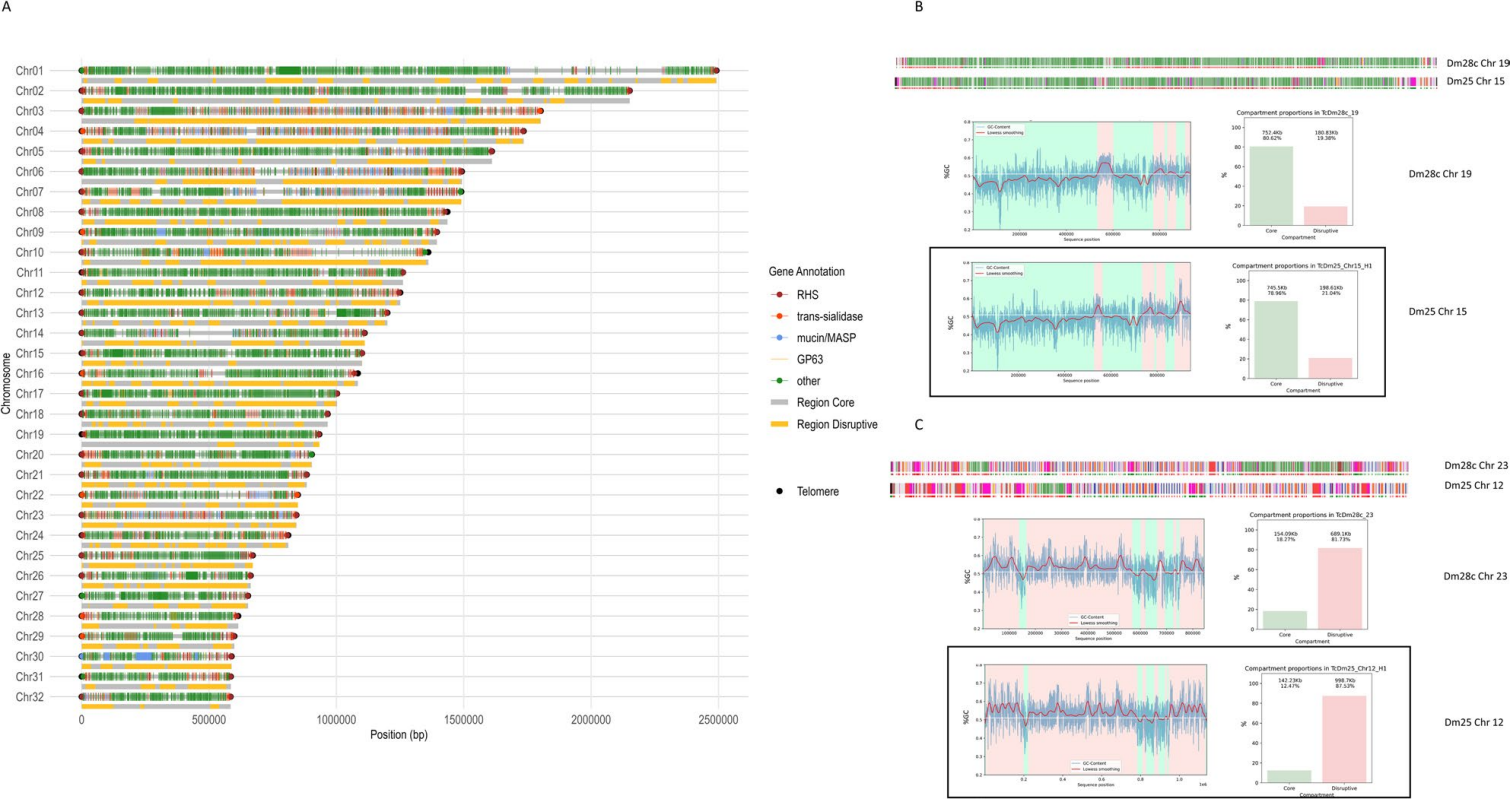
Architecture conservation.** A** Representation of the 32 *T. cruzi* chromosomes. Below each chromosome, core and disruptive regions are shown in gray and orange, respectively. Telomeres are represented as black dots, and the first post-telomeric gene is indicated as dots or lines within chromosomes, color-coded according to the legend. **B** Representation of a chromosome predominantly classified as Core. The plot below displays the GC content and categorization into core and disruptive regions, as determined by the GCScanner program [56]. The graph on the right displays the percentage of each region type. The inset shows the same profile for the homologous chromosome of the Dm25 strain. **C** Like **B**, displaying the profile of a chromosome classified as predominantly disruptive

To gain deeper insight into the conservation of chromosomal architecture, we examined the genomic compartmentalization of *T. cruzi*, a feature now well established [36, 40, 41, 55]. A notable difference previously reported is that the core and disruptive compartments vary significantly in their GC content, with the disruptive compartment exhibiting much higher GC levels [36]. In fact, GC content alone is often enough to predict whether a region belongs to the core or disruptive compartment [36]. To analyze the distribution of core and disruptive regions across chromosomes relative to GC content, we used the GCanner application [56]. Based on this analysis, chromosomes were classified into three groups: core (more than 60% of genes labeled as core), disruptive (more than 60% labeled as disruptive), and mixed (neither category exceeding 60%) (Table 2). This classification was performed for the Dm28c, Dm25, and Sylvio strains. In Dm25, nine chromosomes differed in category relative to Dm28c, while in Sylvio only four chromosomes showed a different classification, and one chromosome was absent from the assembly (see Results Sect. 2). These discrepancies are primarily explained by differences in the lengths of homologous chromosomes that influence GC-based classification, causing some chromosomes to fall on opposite sides of the 60% threshold. Such variation may reflect true biological differences between strains (i.e. chromosome rearrangements) or assembly-related artifacts. In several cases, the discrepancies arise from only marginal numerical differences that cross our arbitrary category boundaries. The chromosomal landscape resulting from this classification is shown in Fig. 3A. The graphic clearly illustrates the correlation (Fig. 3A), identifying nine core chromosomes (example in Fig. 3B), twelve disruptive chromosomes (example in Fig. 3C), and eleven mixed chromosomes. When we applied the same analysis to Dm25 chromosomes, they displayed a nearly identical pattern (Fig. 3B and C, boxed regions; Supplementary Fig. 6). The corresponding chromosomes in both strains kept the same classifications: core chromosomes remained core, disruptive ones remained disruptive, and mixed chromosomes remained mixed (Supplementary Fig. 7). These consistent compartmental profiles across strains further support a high level of structural conservation in chromosome organization.

Overall, the finding that both the number of chromosomes and their compartmental architecture are conserved across *T. cruzi* strains challenges the current paradigm that genomic plasticity dynamically alters chromosome size and organization. Our results strongly indicate that the presence of 32 chromosomes and their organization into compartmental blocks are deeply conserved biological features of the *T. cruzi* species from Clade A. Specifically, the distribution pattern of core and disruptive compartments shows remarkable conservation across strains, further supporting the preservation of structural genomic organization as a fundamental property of *T. cruzi* genome architecture.

### Subtelomeric regions define a distinct genomic compartment in *T. cruzi*

**Fig. 4:**
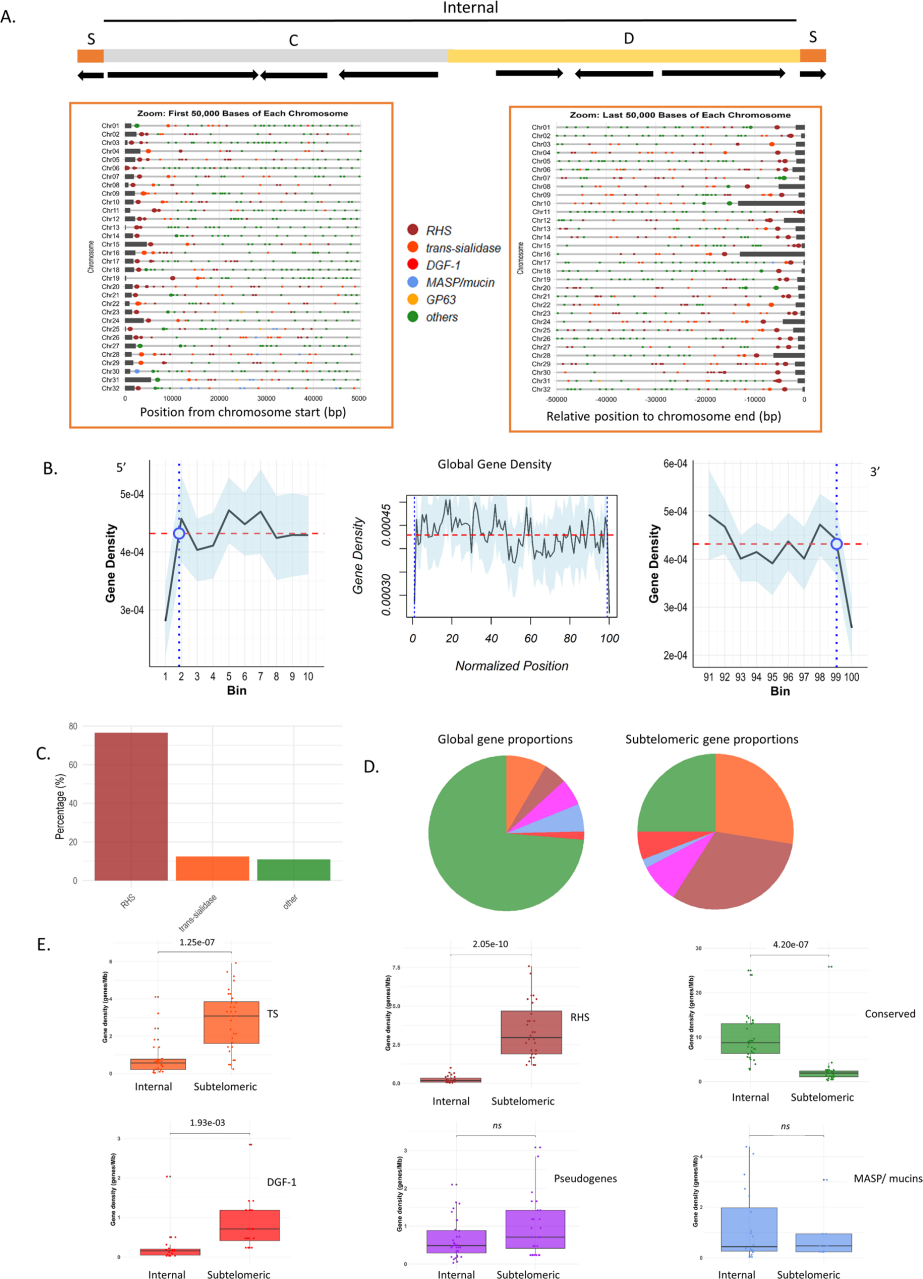
Telomeres and subtelomeres in *T. cruzi*. **A** Representation of chromosomes including three possible compartments (C = core = gray, D = disruptive = yellow, and S = subtelomeric = orange) with a zoom-in on the first and last 50 Kb. Dots indicate genes, and dark gray rectangles represent telomeric sequences. **B** Gene density across chromosomes. Data aggregated from all 32 chromosomes, with lengths normalized into 100 bins. Gene density is shown for the first 10 bins (Crossing point = bin 1.83, bin length: 10.70–45.70.61Kb) (left plot), the entire chromosome (center plot), and the last 10 bins (Crossing point = bin 98, bin length: 5.85–24.92 Kb)(right plot). The red dotted line represents the mean gene density, while the blue line marks the crossing points at the beginning and end. **C** Proportion of RHS, TS, mucin/MASP, or conserved genes detected as the first gene post-telomere sequence. **D** Proportions of genes in the subtelomeric region (right) and whole chromosomes (left). **E** Boxplot representing global gene density (normalized by Mb) comparing internal and subtelomeric regions for Tran-sialidases, RHS, Conserved, DGF-1, MASP/mucins and pseudogenes

All 64 chromosomal termini were confirmed to contain the expected hexameric repeat (CCCTAA/TTAGGG), followed by the 189 bp junction [57] (Fig. 4A). To examine the properties of *T. cruzi* subtelomeres, we employed a stepwise approach. First, we evaluated gene density in the first 50 kb adjacent to each telomere. The overall analysis across all chromosomes revealed a consistent decrease in gene density at the chromosomal ends. To more precisely evaluate this pattern, we compared the mean gene density in the first 0–50 kb of each chromosome with the immediately adjacent 50–100 kb region. A paired Wilcoxon signed-rank test showed a significant reduction in gene density at the subtelomeric 0–50 kb for both the 5′ chromosome ends (V = 116, *p* = 0.0029) and the 3′ ends (V = 72.5, *p* = 1.8 × 10⁻⁴). When averaging both ends per chromosome, the effect became even stronger (V = 55, *p* = 1.3 × 10⁻⁵). Together, these results demonstrate a consistent and statistically significant depletion of genes within the first 50 kb of chromosome ends compared to immediately internal regions The overall analysis across all chromosomes showed a significant decrease in gene density at the chromosomal ends, providing a rough initial boundary of the putative subtelomeric regions (Fig. 4B). This decrease in gene density is observed in core, disruptive, and mixed chromosomes. Next, we analyzed their gene composition using sliding windows of five genes across the entire chromosomes and compared putative subtelomeres with internal regions. A primary observation was that all the subtelomeres comprise a unique DGC oriented toward the chromosomal ends (arrows in Fig. 4A). Second, RHS is the most frequent gene adjacent to the 189 bp junction, appearing in over 80% of cases (Fig. 4C). Third, RHS, TS, and DGF-1 are the most frequent protein-coding genes in the subtelomeres; although core genes are also present, they do not consistently recur across subtelomeres (Fig. 4D and Supplementary Fig. 7, 8). These results allowed us to define the subtelomeric boundary as the + 1 nucleotide of the first conserved gene within the subtelomeric DGC—i.e., the gene located directly next to the internal (non-subtelomeric) region. We then compared the number of genes per megabase between subtelomeric and internal regions, confirming that TS, RHS, and DGF-1 are overrepresented in the subtelomeres, with statistical significance across all chromosomes (Fig. 4E). This pattern persisted regardless of their classification as core, disruptive, or mixed (Supplementary Fig. 8, 9 A). The same trend was observed for pseudogenes of TS, RHS, and DGF-1 (Supplementary Fig. 8, 9B).

To determine if subtelomeres are a preferred environment for pseudogenization, we analyzed the distribution of genes and pseudogenes in the three gene families (Table 3). For the TS family, we observed an uneven distribution: only 28.7% of its functional genes reside in subtelomeric regions, while 42.6% of its pseudogenes are located there, indicating an accumulation or preferential retention of TS pseudogenes in these regions (Table 3). In the RHS and DGF-1 families, gene and pseudogene distributions were similar, showing no bias toward pseudogenization in either (Table 3). Finally, we noted an overrepresentation of L1Tc and SIRE retroelements in the subtelomeres (Supplementary Fig. 9, 10).

**Table 3:** TS, RHS and DGF-1 gene counts and proportions in subtelomeric and internal regions

	TS				
	Internal				
Genes and Pseudogenes	Core	Disrutptive	Internal total	Subtelomeric	Total
Number	25	573	598	326	924
Percentage	2.71	62.01	64.72	35.28	100.00
Genes					
Number	15	332	347	140	487
Percentage	3.08	68.17	71.25	28.75	100.00
Pseudogenes					
Number	10	241	251	186	437
Percentage	2.29	55.15	57.44	42.56	100.00

	RHS				
	Internal				
Genes and Pseudogenes	Core	Disrutptive	Internal total	Subtelomeric	Total
Number	30	159	189	446	635
Percentage	4.72	25.04	29.76	70.24	100.00
Genes					
Number	28	108	136	293	429
Percentage	6.53	25.17	31.70	68.30	100.00
Pseudogenes					
Number	2	51	53	153	206
Percentage	0.97	24.76	25.73	74.27	100.00

	DGF-1				
	Internal				
Genes and Pseudogenes	Core	Disrutptive	Internal total	Subtelomeric	Total
Number	33	88	121	90	221
Percentage	14.93	39.82	54.75	40.72	100.00
Genes					
Number	16	55	71	55	126
Percentage	12.70	43.65	56.35	43.65	100.00
Pseudogenes					
Number	17	33	50	35	85
Percentage	20.00	38.82	58.82	41.18	100.00

Overall, these findings support the notion that the subtelomeres constitute a distinct genomic compartment in *T. cruzi*. While the disruptive compartment represents a genomic expansion predominantly expressed in the trypomastigote stage and mainly composed of *MUC*,* MASP*, and *TS* genes, the subtelomeric regions are composed mainly of *DGF-1*, *RHS*, and *TS* genes.

### Subtelomeric organization and expression of *TS*, *RHS*, and *DGF1* genes

**Fig. 5:**
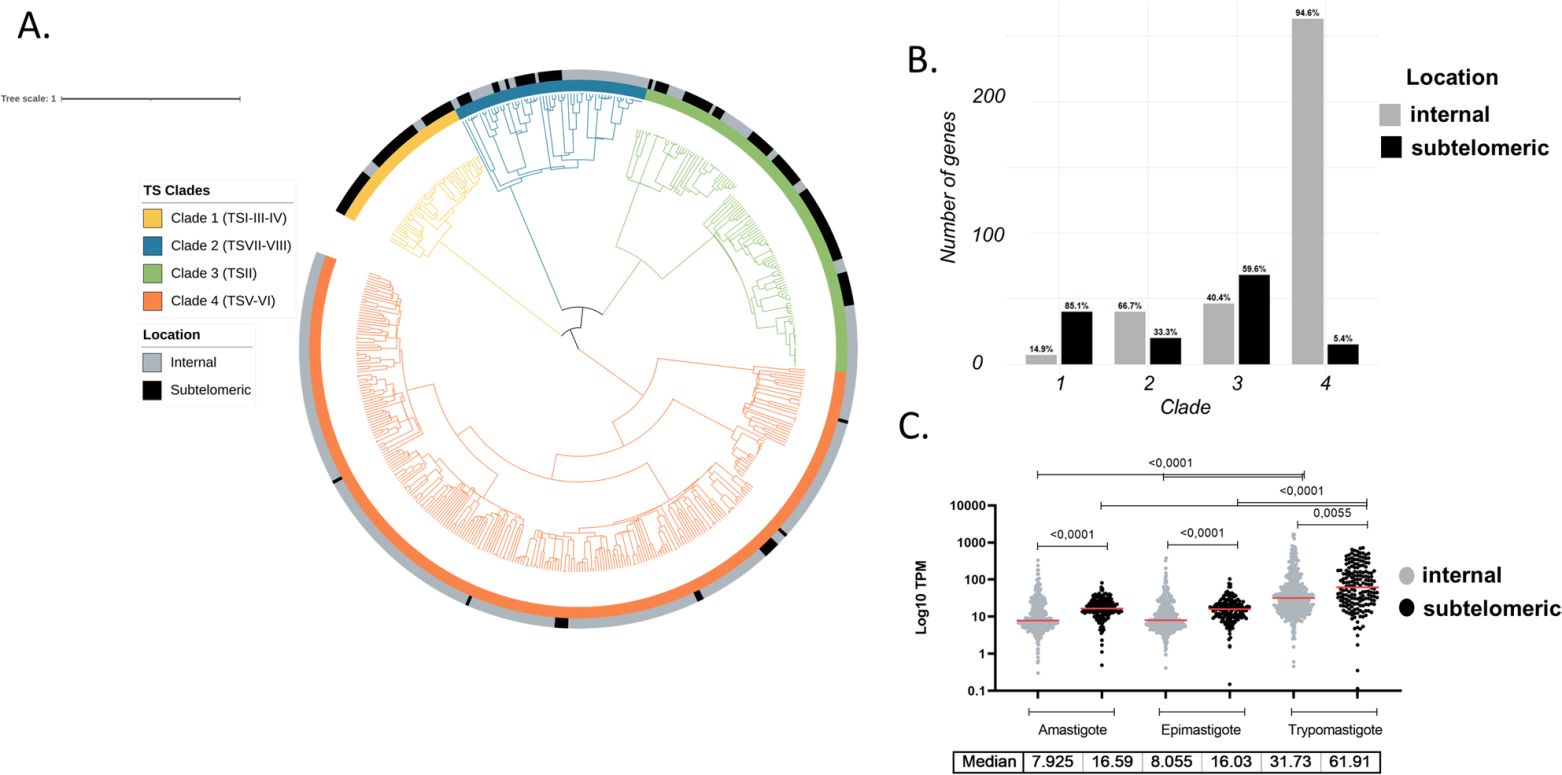
Guide tree and expression of Trans-sialidases. Sequences were aligned with Clustal Omega, the tree was generated using the Neighbor-Joining method based on pairwise sequence distances, and the tree was visualized in iTOL. **A** Tree colored by clade (inner circle) and location (outer circle, subtelomeric or internal). **B** Barplot showing the number of TS genes by clade and location. **C** Expression levels (TPM) by genomic location in epimastigotes, amastigotes, and trypomastigotes. Statistical differences were assessed using the ANOVA test. Significant *P*-values < 0.05 are indicated. Median TPM values for each stage/location are indicated behind the plot

The discovery that TS genes are present in both the disruptive and subtelomeric compartments prompted us to explore whether the same TS groups are spread across them. To date, the most detailed classification of TS genes describes eight TS groups (I-VIII) [4]. However, the sequences used at that time might no longer accurately reflect the current completeness, considering advancements in long-read sequencing technologies. Therefore, we reexamined the TS family using sequence alignments and similarity-based clustering, which revealed four main clades—groups 1–4—each containing clearly defined subclades (Fig. 5A, Supplementary Table 2). Although the overall classification roughly aligns, we prefer to refer to the clades defined here for the following reasons: (i) Clade 1 includes groups I, III, and IV and is divided into three subclades, but these do not match the previous groups; (ii) Clade 2 contains groups VII and VIII, but they are mixed; (iii) Clade 3 corresponds to the most numerous group, group II, which is further split into at least two subclades; (iv) Clade 4 comprises groups V and VI, forming a single clade with five subclades (labeled a–e in Supplementary Fig. 11 and Supplementary Table 2), each holding members from both groups. We then examined whether there was a preferred distribution among compartments and found that Clades 1 and 3 are mostly subtelomeric genes (85% and 59%, respectively), while Clade 4 members mainly reside in internal regions (95%), and Clade 2 has an intermediate distribution (Fig. 5A, B, and Supplementary Table 2). Finally, we compared their expression patterns and discovered that TS expression was highest in trypomastigotes, followed by amastigotes and epimastigotes (Fig. 5C), aligning with the stage-specific roles of these surface proteins. Additionally, subtelomeric TS genes showed significantly higher expression than disruptive TS genes across all stages, highlighting the impact of genomic location on gene expression.

**Fig. 6:**
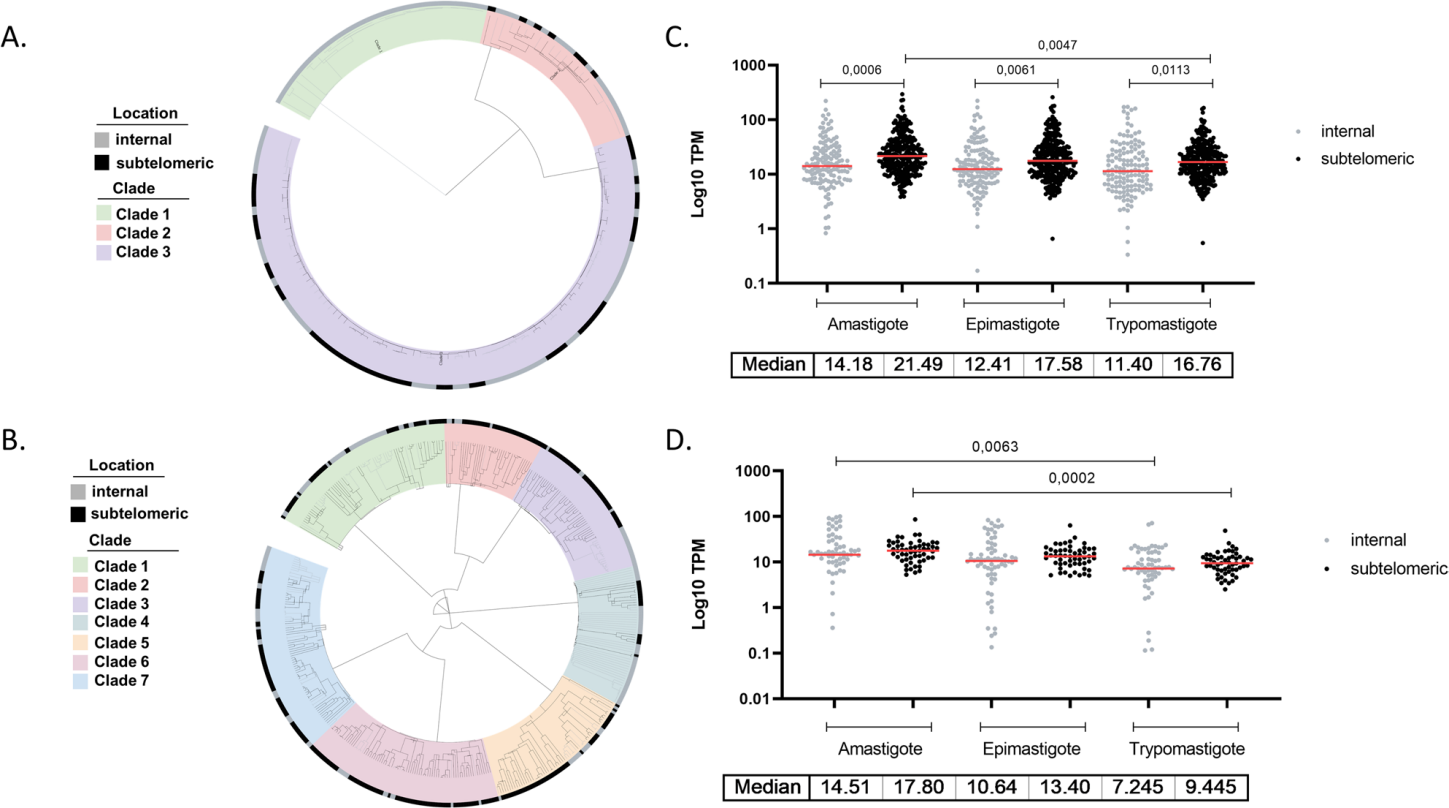
Guide tree and expression of RHS and DGF-1 proteins.** A** RHS guided tree colored by clade. Sequences were aligned using Clustal Omega based on pairwise sequence distances, and the tree generated by the Neighbor-joining method was visualized with iTOL. Subtelomeric or internal genomic location is indicated for each sequence (outer circle). **B** Guided tree colored by clade as **A** for DGF-1 proteins. **C** Expression levels (TPM) by location in epimastigotes, amastigotes, and trypomastigotes were plotted for RHS genes. Statistical differences were assessed using the ANOVA test. Significant *P*-values < 0.05 are indicated. **D** Same expression analysis shown in **B**, for DGF-1 genes. Median TPM values for each stage/location are indicated behind the plots **B** and **D**

Sequence-based clustering was also conducted for the *RHS* and *DGF-1* genes. For DGF-1, we identified three main clusters that differ in their chromosomal distribution (Fig. 6A and Supplementary Table 2). Cluster 1 includes genes exclusively located in internal regions; cluster 2 is mainly composed of internal genes, with about 30% in subtelomeric regions; and cluster 3 shows a balanced distribution, with roughly half of the genes in each compartment. Regarding RHS genes, because most are located in the subtelomeric region, they do not display a clear pattern of compartment-specific distribution (Fig. 6B). The dendrogram indicates no tendency for subtelomeric genes to cluster within specific clades. Expression analysis shows that, for DGF-1, genes in the subtelomeric compartment exhibit higher expression levels than those in internal regions across all stages (Fig. 6C and Supplementary Table 2). Additionally, we found a statistically significant difference in expression levels between the replicative amastigote and non-replicative trypomastigote stages for DGF-1 genes in the subtelomeric compartment. For RHS genes, a similar expression pattern to that of DGF-1 was observed. Significant differences in expression levels were detected for genes in both compartments between the replicative (amastigote) and non-replicative (trypomastigote) stages, with higher expression in the former (Fig. 6D).

## Discussion

Twenty years ago, the draft genomes of *T. cruzi*, *L. major*, and *T. brucei* (referred to as TriTryps) were published, along with a comparative genomic analysis [21, 32–34]. At that time, only the *T. cruzi* genome could not be assembled at chromosomal resolution due to its highly repetitive nature, which caused extensive fragmentation. As a result, its chromosome number remained unknown. Additionally, chromatin does not condense sufficiently during mitosis to allow for the visualization of individual chromosomes, which hampers cytogenetic approaches. Therefore, pulsed-field gel electrophoresis (PFGE) became the standard method for estimating the karyotype in *T. cruzi*. Rigorous PFGE studies showed that the parasite has 20–40 chromosomes, with sizes ranging from approximately 0.46 to 3.27 Mb across different lineages [24–30]. However, although these studies produced distinguishable bands that appeared to be chromosomes, stoichiometric resolution was not always achieved due to overlapping bands, which made it difficult to determine the exact chromosome number. The emergence of long-read sequencing technologies, such as Nanopore and PacBio, has significantly advanced genome characterization and organization. However, while some studies referred to scaffolds assembled from contigs as chromosomes [39], these designations were overstated; it was only with the introduction of PacBio HiFi sequencing that complete chromosomes were finally recovered [43]. Nonetheless, a fully complete chromosomal complement has yet to be achieved.

In this study, we successfully assembled the T2T genome of the *T. cruzi* Dm28c strain, resulting in a comprehensive characterization of its karyotype, comprising 32 linear chromosomes ranging in size from 0.58 to 2.49 Mb. Each chromosome contains canonical telomeric repeats of variable length and the conserved 189-bp junction sequence at both ends [48, 58, 59], confirming its chromosomal identity. Notably, PFGE analysis of the Dm28c strain previously reported chromosome sizes ranging from 0.57 to 2.50 Mb [30], which closely matches our findings and highlights the accuracy of earlier studies. Importantly, this karyotype is conserved even in genetically and geographically distant strains. Each Dm28c chromosome maps to a syntenic homolog in Dm25 and Sylvio X10, allowing the reconstruction of the Dm25 karyotype, the identification of an incomplete chromosome assembly in the Sylvio X10 genome, and enabling detailed comparative genomics studies. Chromosome sizes are largely conserved, with most differences due to the absence of telomeric ends in the Dm25 strain. To evaluate the conservation of chromosomal architecture, we compared the GC content profiles of both karyotypes and found a high degree of similarity, with nearly identical GC content along each chromosome. However, it is essential to note that GC content and compartmental organization are not equivalent. For example, while DGF-1 genes have a high average GC content (~ 63%), they are not limited to the disruptive compartment; they can also be found in subtelomeric regions or within the core. Nonetheless, GC profiling proved to be a helpful proxy for broadly delineating genomic compartments and identifying structural conservation across strains [36]. We also observed remarkable conservation of ploidy among additional, genetically and geographically diverse Clade A strains. All studied strains are diploid, except for chromosome 16, which is consistently tetrasomic in all analyzed strains. Strikingly, the tetrasomic chromosome in *T. cruzi* corresponds to chromosome 31 in *L. major*, which is also tetrasomic. Finally, extensive synteny was observed between the core genomes of *T. cruzi* and *L. major*, highlighting a conserved chromosomal architecture among trypanosomatids.

By resolving the complete *T. cruzi* karyotype of this strain, we establish a reference framework and propose a standardized chromosomal nomenclature (chromosomes 1 to 32, based on length) to serve as a basis for future genomic, transcriptomic, and proteomic studies. This facilitates the identification of structural variations associated with pathogenicity, virulence, host adaptation, and drug resistance, ultimately aiding in the development of more effective strategies to combat Chagas disease. Additionally, the remarkable conservation of chromosomal architecture across strains challenges the prevailing notion that *T. cruzi* exhibits extensive genome plasticity driven by widespread rearrangements. Instead, the discovery of a well-organized genome and conserved chromosomal structure suggests a paradigm shift: large-scale chromosomal rearrangements are not the primary drivers of plasticity. The presence of a conserved genome structure across strains strongly supports a model in which plasticity occurs within a stable, regulated genomic framework. This means that *T. cruzi* generates diversity not through random, large-scale rearrangements but through localized and controlled mechanisms such as recombination, duplication, and small-scale rearrangements. This model aligns with our previous findings on the conservation of three-dimensional chromatin organization [42]. The genome is organized into chromatin folding domains (CFDs), which are closely associated with gene expression and conserved across strains and life-cycle stages. Furthermore, our 3D studies revealed that the *T. cruzi* genome is partitioned into two 3D compartments, C and D, corresponding to the core and disruptive genomic regions, respectively. While predominantly intrachromosomal interactions characterize compartment C, compartment D shows extensive interchromosomal contacts, a conformation that favors discrete recombination and rearrangement sites, thereby aiding gene family diversification and antigenic variability without threatening chromosomal integrity. We propose that chromatin dynamics are the key mechanisms linking this stable chromosome framework to the high genomic plasticity.

The observed synteny across chromosomes from different *T. cruzi* strains is absent at the chromosomal ends, as shown in the CIRCOS plots, highlighting the variability of these terminal regions. This prompted us to identify and analyze the gene organization of all 64 subtelomeric regions. These regions exhibit a sharp decrease in gene density, strong enrichment of retroelements, and a complete loss of synteny between strains. Each subtelomere contains a unique DGC oriented toward the telomere, along with a characteristic buildup of the multigene families RHS, TS, and DGF-1. In contrast, mucin and MASP genes are nearly absent. The specific subtelomeres previously reported and analyzed through cloning and sequencing strategies have been shown to be enriched in these genes [48, 58–60]. RHS genes appear to play a crucial role in subtelomeric structure and function, as most are located in these regions and, in approximately 80% of cases, are immediately adjacent to the 189-bp telomeric junction. Conversely, DGF-1 genes are more broadly distributed across the genome, found in both disruptive and subtelomeric regions, as well as 20% in core regions, indicating a broader function not limited to subtelomeres. Regarding TS genes, a global analysis suggests they are relatively evenly spread across disruptive and subtelomeric compartments. However, a closer examination shows that it is not the case. The trans-sialidase family, initially divided into eight groups (I-VIII), requires reevaluation based on the full-length gene sequences now available. Our analysis shows that groups I, III, and IV form a single, well-supported clade, which we now call TS group 1. Within this clade, three distinct subclades are identifiable. Notably, these subclades do not directly correspond to the previously described groups I, III, and IV. Similar findings were observed for the other main clades, 2, 3, and 4, emphasizing the need for a revised classification based on phylogenetic relationships derived from complete TS gene sequences across strains. Most genes within the three subtelomeric families are transcriptionally active. In contrast to the disruptive compartment, which is largely composed of genes needed during the trypomastigote stage, the subtelomeric regions contain genes expressed during either the replicative or non-replicative stages. Finally, the association of the *RHS* and *DGF-1* genes emerges as a hallmark of sub-telomeric regions, while the co-occurrence of *MASP* and *MUC* genes characterizes the disruptive compartment. *TS* genes are distributed across both compartments, with specific clades tending to be enriched in one or the other. In summary, these findings support a model in which the *T. cruzi* genome is divided into three functionally distinct compartments—core, disruptive, and subtelomeric—each contributing differently to the adaptability and evolution of the parasite. The core compartment provides a conserved backbone necessary for parasite survival and is conserved across trypanosomatids; the disruptive compartment contains expansions of genes overexpressed in the infective and non-replicative trypomastigote stage; and the subtelomeric compartment, now clearly characterized, acts as a hotspot for gene diversification and pseudogenization, serving as a reservoir for antigenic diversity and genomic change.

## Conclusions

By resolving the *T. cruzi* Dm28c genome from telomere to telomere, this study establishes a definitive chromosomal framework for the species and resolves the long-standing uncertainty about its karyotype. The identification of a conserved complement of 32 linear chromosomes, together with preservation of chromosomal homology across Clade A (TcI) strains, demonstrates that the *T. cruzi* genome is organized around a stable, ordered chromosomal architecture. This structural conservation is reflected in the highly conserved distribution of core and disruptive genomic compartments across chromosomes and strains, indicating that genome plasticity in *T. cruzi* does not primarily arise from pervasive large-scale rearrangements. Instead, our results support a model in which essential genomic functions are maintained within a conserved chromosomal framework, while variability is spatially constrained.

Within this context, subtelomeric regions emerge as a distinct third genomic compartment, enriched in RHS, DGF-1, and specific clades of trans-sialidase genes, that concentrates structural variability, gene diversification, and pseudogenization. This compartment represents a significant source of interstrain diversity and highlights the functional relevance of genomic location in shaping parasite adaptability.

Together, the results presented here provide a foundation for future comparative genomics, functional studies of genome evolution, antigenic diversity, and host–parasite interactions in *T. cruzi* and related trypanosomatids.

## Materials and methods

### Genome visualization and web interface

As described in the Results, to facilitate data visualization and usability, we utilized that model again with minor modifications. The new Dm28cT2T genome assembly and annotation are accessible through https://cruzi.pasteur.uy/. As in the previous version, the direction of the DGC is indicated underneath each represented chromosome. From each chromosome, the circos [61] and Yass [62] plots can be displayed, and the fasta and gff files can be downloaded (Supplementary Fig. 1). As before, the additional functionalities were preserved, such as viewing gene annotations, retrieving nucleotide and amino acid sequences, and conducting various searches by annotation or keyword.

The genome is also available at NCBI (NCBI: PRJNA1173111, Genome accession number: JBIQOJ000000000).

### DNA extraction and sequencing

Cryopreserved epimastigotes of *T. cruzi* Dm28c_2018 [36], were grown in liver infusion tryptose (LIT) medium supplemented with 10 % fetal bovine serum at 28° C; total DNA was extracted using the Quick DNA Universal kit and Quick DNA HMW MagBeads Kit (Zymo Research, USA), quantified with Qubit (Invitrogen, USA). Five micrograms of each extraction were pooled and sent to the Macrogen service to perform PacBio Hi-Fi sequencing. 301,047 reads were obtained (Read N50 = 10688), representing 2,926,558,665 bases (73x coverage for an estimated 40 Mb genome) with an average read quality of Q29. Raw reads were deposited in SRA (SRR31351846). Illumina sequencing was performed by Macrogen service. 54,374,830 paired-end reads (101 bp) were obtained (274X coverage). Raw Illumina data were deposited in SRA under the accession number SRR33678280.

### Genome assembly

The default HiFiAsm pipeline [46] (hifiasm -o Prefix -t 32 Reads.fq.gz) was used to generate an initial assembly comprising 51 contigs and two haplotypes with 80 and 86 contigs, respectively (Supplementary Fig. 12). Among the 51 contigs, one—assembled as circular—corresponded to the maxicircle sequence (TcDm28c_aMaxicircle; 50,477 bp), confirming its recovery for this strain as previously reported [63]. The remaining 50 contigs included 25 telomere-to-telomere (T2T) chromosomes, 18 of which overlapped with the 24 T2T chromosomes previously assembled for the Dm25 strain. Contigs containing a single telomere were manually extended and joined using three complementary approaches: (i) DotPlot (YASS) analyses to detect overlaps suitable for merging; (ii) Circos comparisons of all chromosomes against the complete Dm25 chromosomes; and (iii) YASS analyses between Dm25 chromosomes and homologous contigs from the present assembly. All 32 assembled chromosomes contained telomeric repeats (CCCTAA/TTAGGG) at both ends, confirming their telomere-to-telomere continuity. The Sylvio genome was used to validate the seven manually assembled chromosomes (Supplementary Fig. 12), confirming that all assembled chromosomes were correctly reconstructed. The complete assembly workflow is shown in Supplementary Fig. 12. Chromosomes were sorted and numbered by decreasing length, ranging from TcDm28c_01 (2.5 Mbp) to TcDm28c_32 (0.6 Mbp).

### Genome annotation

To annotate the coding sequences, we first trained Augustus [64, 65] using *T. cruzi* (previous annotation of the Dm28c genome) to produce the initial annotation GFF file. We then corrected the start and stop codons of each CDS using the getorf method as previously described [66]. All CDS were retrieved and annotated using MMseqs [67] against the UniProt database (uniref_100). The coding sequences were categorized into three possible outcomes: no similarity to the UniProt database, similarity to the UniProt database, or truncated/multiple possible annotations (Supplementary Fig. 1 A). Busco [68] was used to evaluate the completeness of the gene space relative to the Euglenozoa database, indicating the quality of the assembly (Supplementary Fig. 1B). The complete pipeline is summarized in Supplementary Fig. 1A. The web page featuring the new genome and its annotation was constructed based on a previous publication by our group [36] and is available at https://cruzi.pasteur.uy.

Tandem repeats and satellite sequences were annotated with the Tandem Repeat Finder (TRF) algorithm [69]. Satellite regions were retrieved from the TRF output by filtering the repeat lengths between 190 and 200 bp. Additionally, we tested our annotation by comparing it with the algorithm developed by Dean et al. (2025) [13] for classifying MASP genes, obtaining equivalent results.

### Ploidy analysis

For ploidy analysis, Illumina reads (SRR3676268, SRR3676272, and SRR33678280) were mapped against the Dm28c T2T genome assembly using minimap2 [70]. Samtools depth [71] was used to calculate depth using sliding windows. Finally, Rscript (see next) was used to plot data.

### Computational analysis

All scripts used to produce data and figures were available on github (www.github.com/Gon1976/TcruziGenome). R scripts were used to estimate gene density along the chromosomes. Gene density was computed using the countOverlaps function from the GenomicRanges package in R [72]. This analysis was performed for all chromosomes. Circos [61] was used to create Fig. 2A, using data from minimap2 comparison with Dm28c and Dm25 genomes. SyMap [73] was used for synteny mapping, and circos in Fig. 2B, C, D, and E. The Circlize package [74] in R was used to create Fig. 1, including information on GC content (calculated using the BioString package [75]) and gene density. The ggplot package in R was used to create gene representation in the subtelomeric regions using gff data. For analysis of GC content and compartment classification, we used the GCanner application (https://github.com/virginiabalouz/GC-content/).

### TS, RHS, and DGF-1 analysis

For the analysis of TS coding genes, we first retrieved the protein sequences corresponding to the eight previously defined TS groups [4]. From the initial dataset of 505 sequences, those shorter than 400 amino acids and lacking the VTV motif were excluded. The remaining sequences were aligned using Clustal Omega with default parameters. The resulting guide trees were visualized in iTOL to identify clusters of similar TS proteins. Based on these analyses, the sequences were regrouped into TS1, TS2, TS3, TS4, TS5-6, and TS7-8, and clustered using CD-HIT at an 85% identity threshold. TS sequences from the Dm28c strain were manually curated by removing those lacking the characteristic TcS (VTV) motif of this family. A guide tree was then constructed using these sequences, along with TS sequences from CL Brener, allowing classification of Dm28c TSs into the previously defined groups.

For RHS and DGF-1, sequences were retrieved from the annotation file generated in this work (gff) and classified as internal or subtelomeric based on their coordinates. The sequences were aligned, and a guide tree was constructed with Clustal Omega, as previously described for TS, and visualized in iTOL. The generated trees were used exclusively for sequence clustering and visualization of similarity, not for phylogenetic inference.

### Transcriptomics analysis

Raw RNA-seq reads were retrieved from NCBI (BioProject ID PRJNA850400) and fully reprocessed. Read quality was assessed with FastQC [76], and adapter sequences were trimmed using Cutadapt [77]. Clean reads were analyzed using the new genome assembly and its updated gene annotations. Transcript quantification was performed using Salmon [76] v1.5.173 to obtain gene-level read counts and transcript abundances expressed in transcripts per million (TPM). Replicate consistency was evaluated using Pearson correlation and principal component analysis (PCA). An expression data table was generated, including TPM values for all genes across the three parasite stages (trypomastigote, epimastigote, and amastigote). Genes were classified as belonging to the core or disruptive compartments based on their genomic coordinates in the new assembly. Finally, statistical analyses were performed using DESeq2 to assess differential expression between compartments.

## Supplementary Information

Supplementary Material 1.

Supplementary Material 2.

Supplementary Material 3.

Supplementary Material 4.

Supplementary Material 5.

Supplementary Material 6.

Supplementary Material 7.

Supplementary Material 8.

Supplementary Material 9.

Supplementary Material 10.

Supplementary Material 11.

Supplementary Material 12.

Supplementary Material 13.

Supplementary Material 14.

## Data Availability

The raw data (PacBio HiFi and Illumina reads) and genome were deposited in NCBI under Accession number PRJNA1173111. The genome and annotation can also be accessed using our webpage: [cruzi.pasteur.uy/] (http://cruzi.pasteur.uy/). All scripts used to produce data and figures were available on github ([www.github.com/Gon1976] (http:/www.github.com/Gon1976).

## Abbreviations

DGC: Directional gene cluster
DGF-1: Dispersed gene family 1
DTU: Discrete typing unit
kDNA: Kinetoplast DNA
MASP: Mucin-associated protein
MUC: Mucin
PFGE: Pulsed field gel electrophoresis
RHS: Retrotransposon hot spot
TS: Trans-sialidase
T2T: Telomere to telomere
