# Supplementary material for: The complete genome of Trypanosoma cruzi reveals 32 chromosomes and three genomic compartments
Source: BMC Genomics. 2026 Jan 8;27:159. doi: 10.1186/s12864-025-12482-0 (PMC12879350; doi:10.1186/s12864-025-12482-0)

Supplementary Figure 3. A. Genome Annotation pipeline

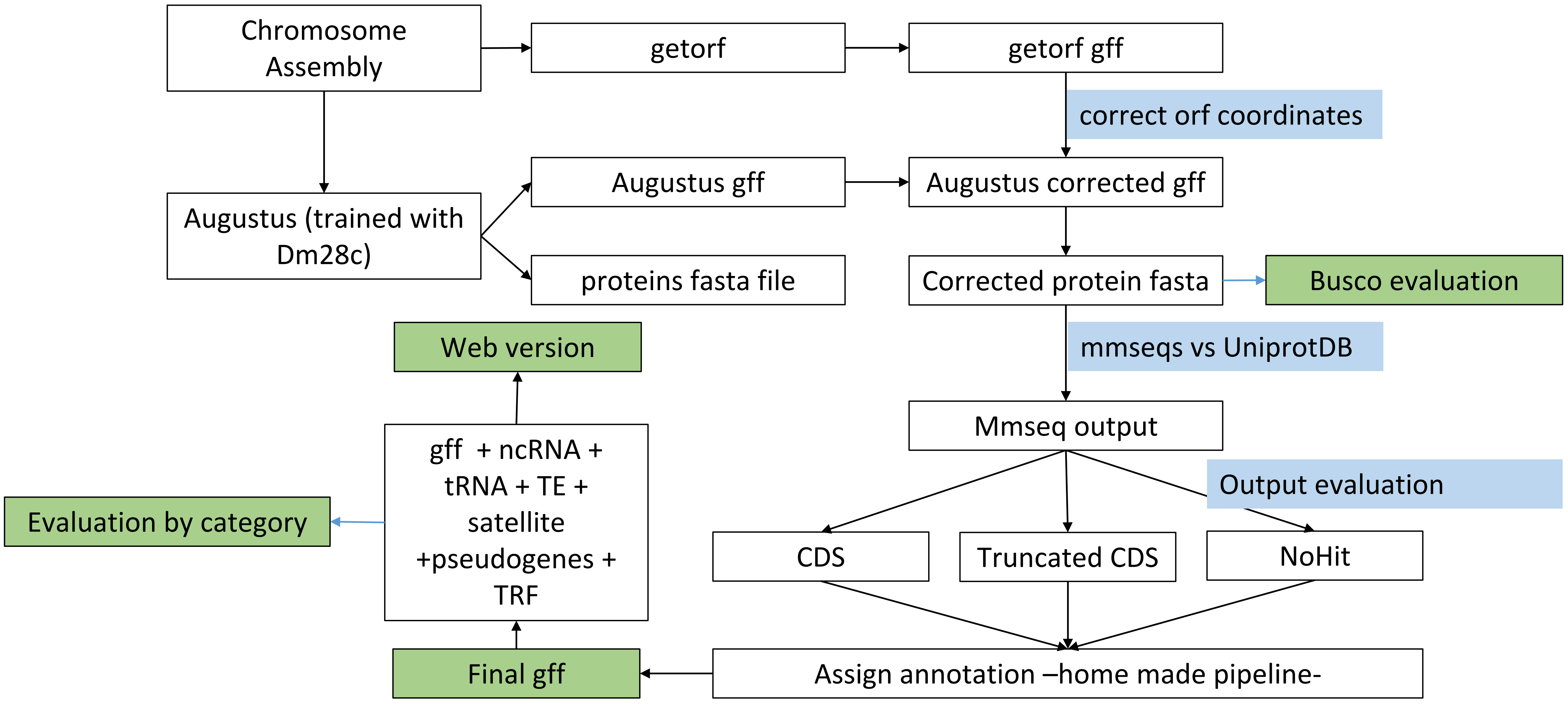

Supplementary Figure 3. B. Busco results. Complete genomes TcI_A4, TcI_Dm25, and TcI_Dm28c_2018 were included with the new genome assembled.

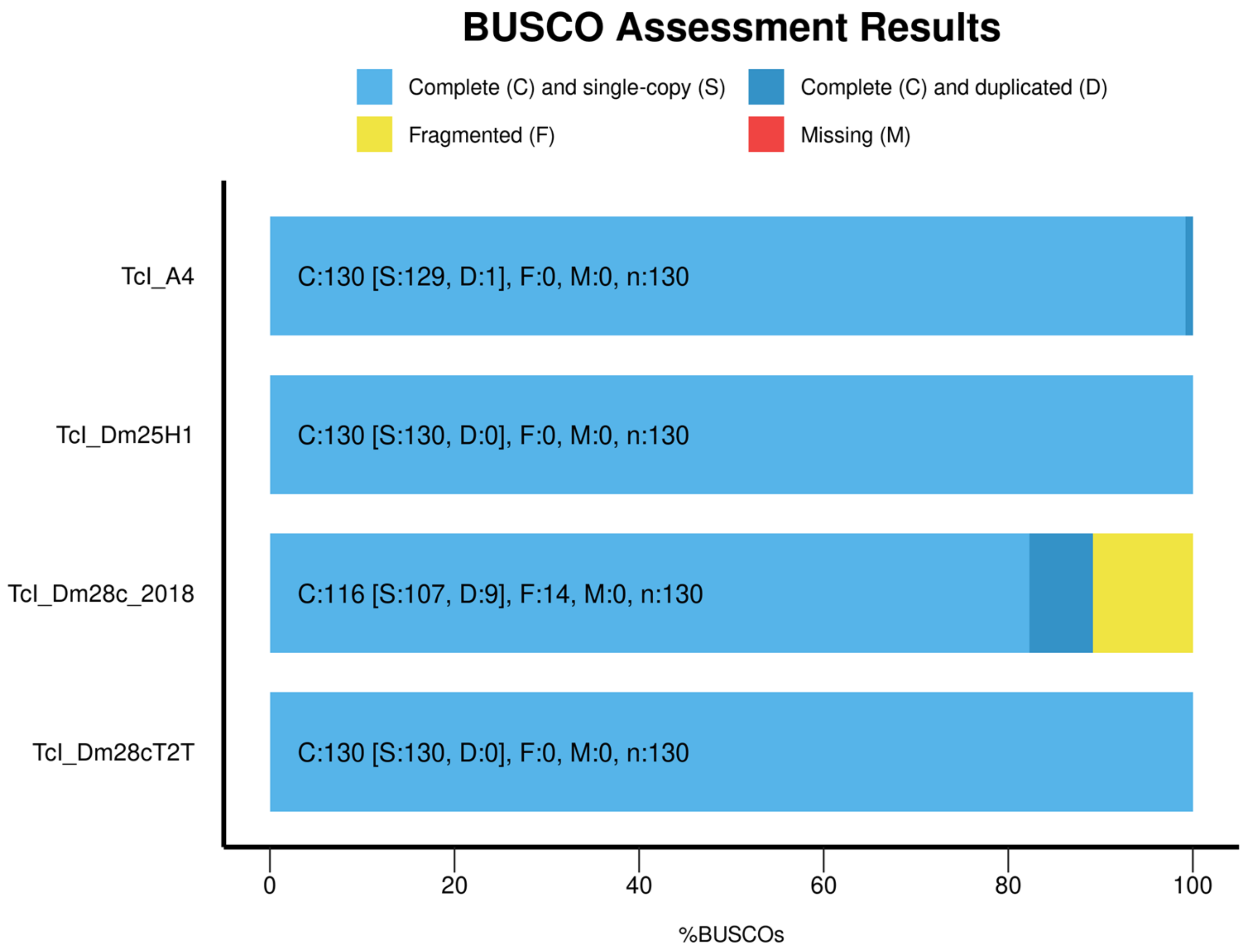

Supplement: Supplementary file 1 — Supplementary Material 1. [file 12864_2025_12482_MOESM1_ESM.pdf]
