# Supplementary figures and images for: The complete genome of Trypanosoma cruzi reveals 32 chromosomes and three genomic compartments
Source: BMC Genomics. 2026 Jan 8;27:159. doi: 10.1186/s12864-025-12482-0 (PMC12879350; doi:10.1186/s12864-025-12482-0)

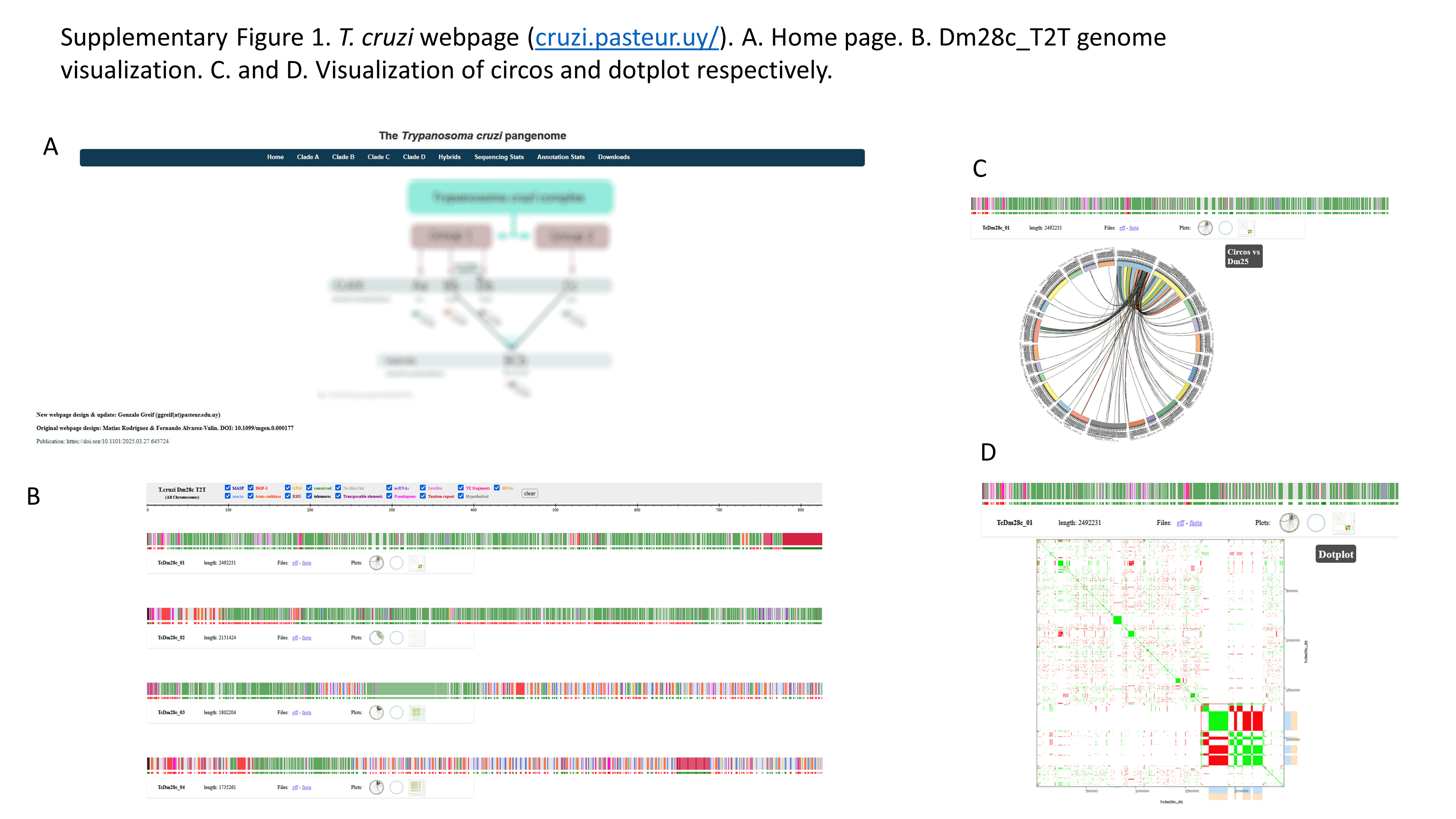

Supplement: Supplementary file 2 — Supplementary Material 2. [file 12864_2025_12482_MOESM2_ESM.tif]

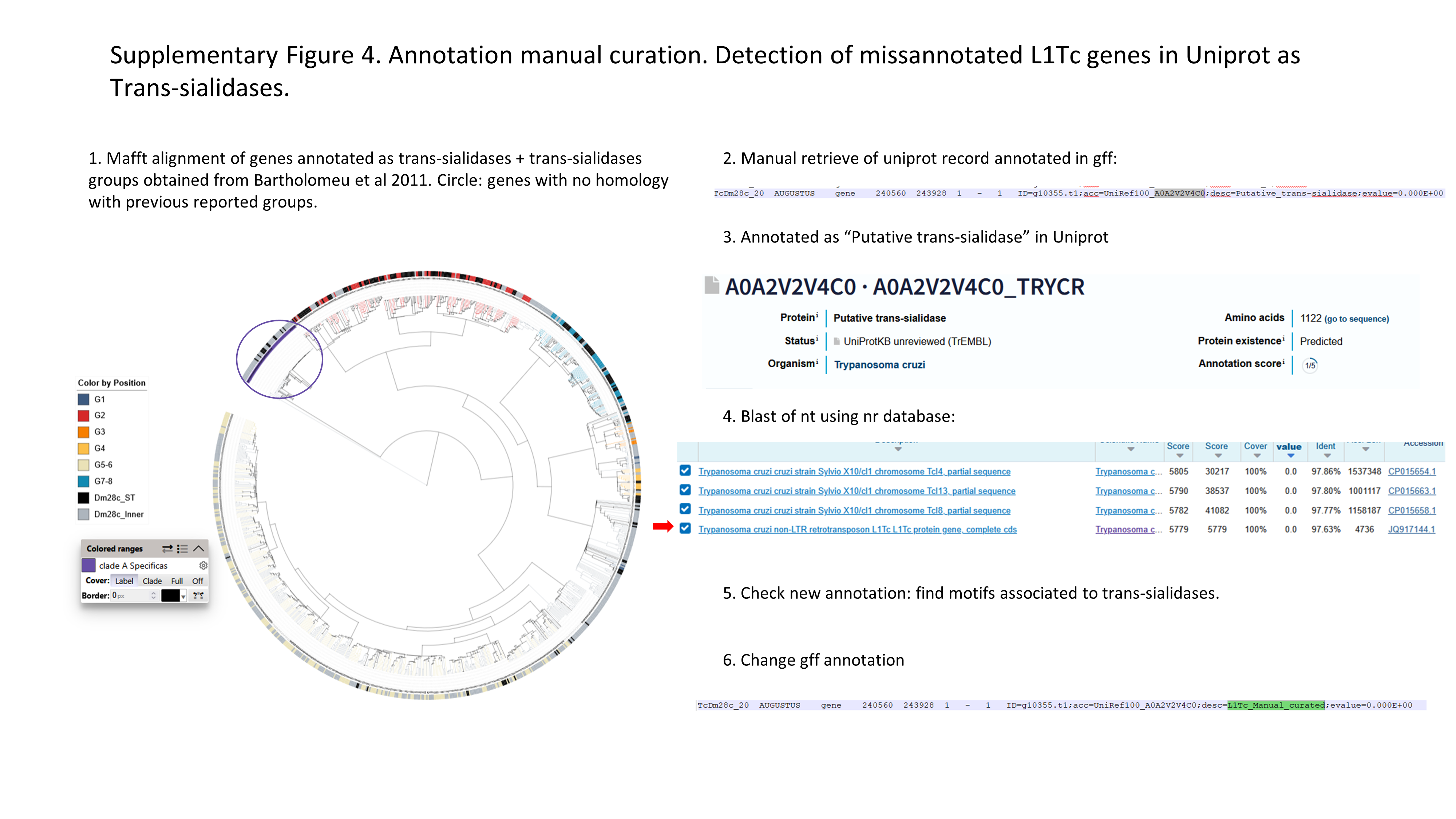

Supplement: Supplementary file 3 — Supplementary Material 3. [file 12864_2025_12482_MOESM3_ESM.tif]

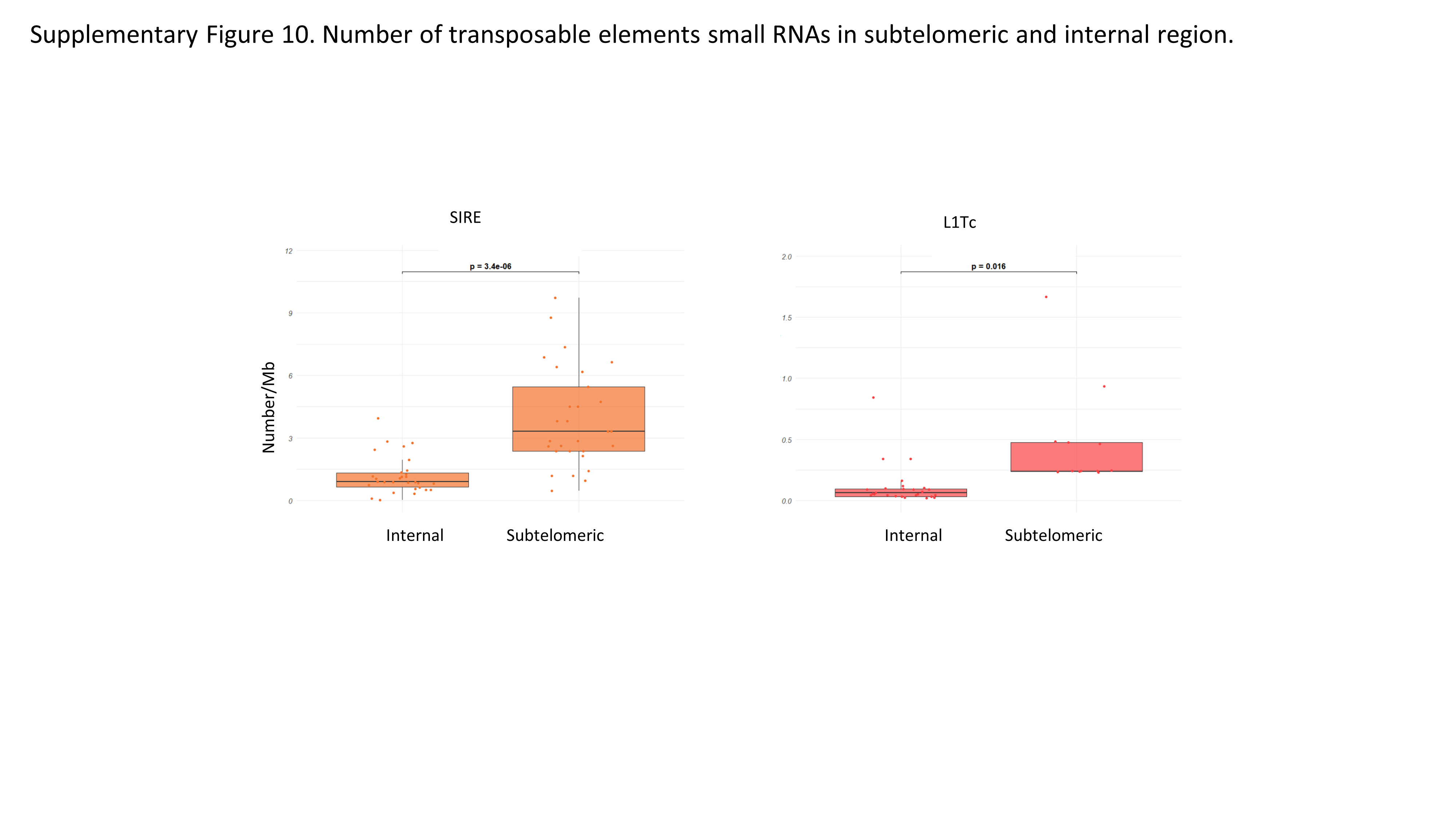

Supplement: Supplementary file 10 — Supplementary Material 10. [file 12864_2025_12482_MOESM10_ESM.tif]

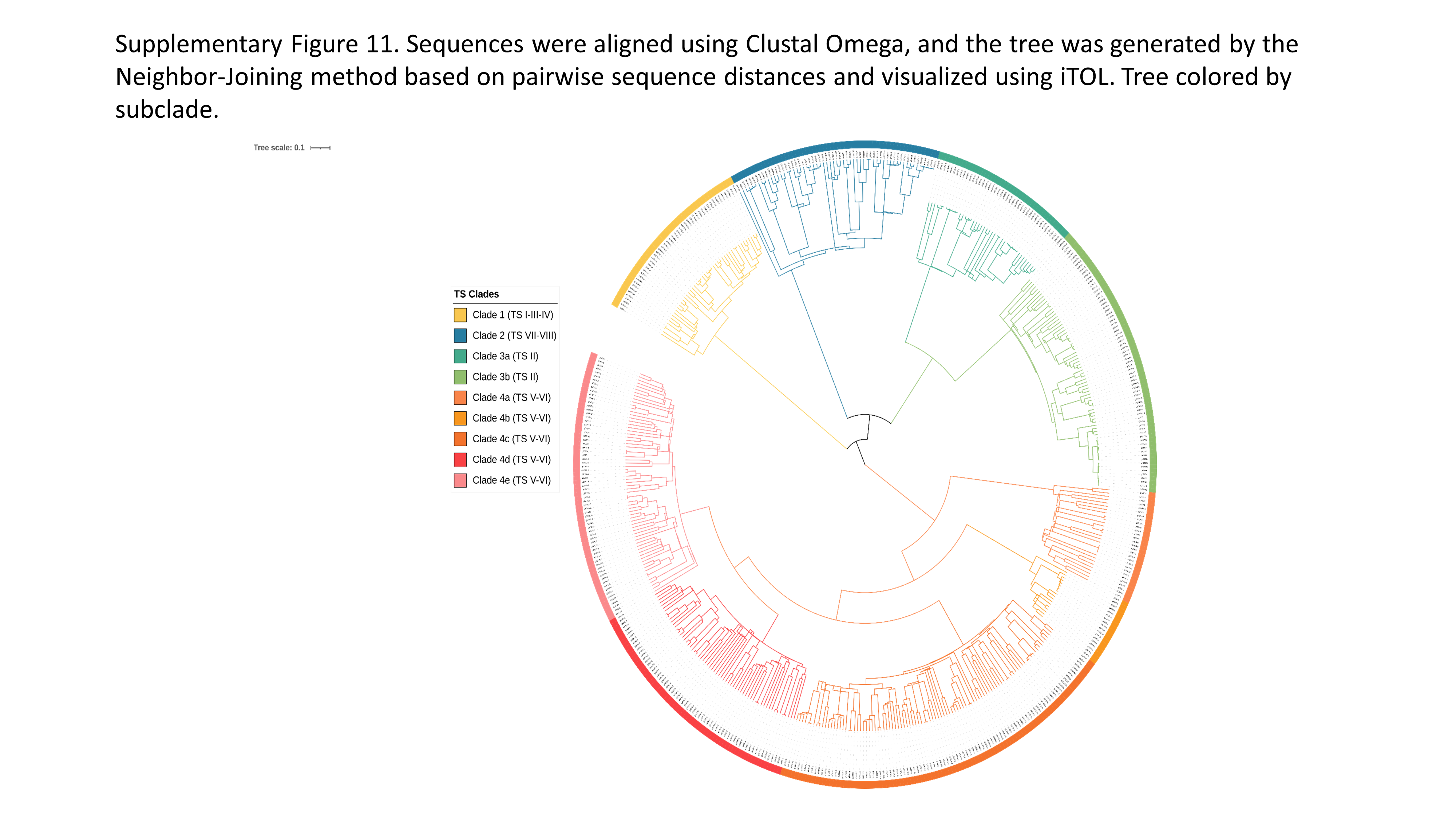

Supplement: Supplementary file 13 — Supplementary Material 13. [file 12864_2025_12482_MOESM13_ESM.tif]
