# Supplementary material for: The complete genome of Trypanosoma cruzi reveals 32 chromosomes and three genomic compartments
Source: BMC Genomics. 2026 Jan 8;27:159. doi: 10.1186/s12864-025-12482-0 (PMC12879350; doi:10.1186/s12864-025-12482-0)

Sylvio T2T (2025): 31 chromosomes + Maxicircle vs Dm28c T2T (2025): 32 chromosomes + Maxicircle

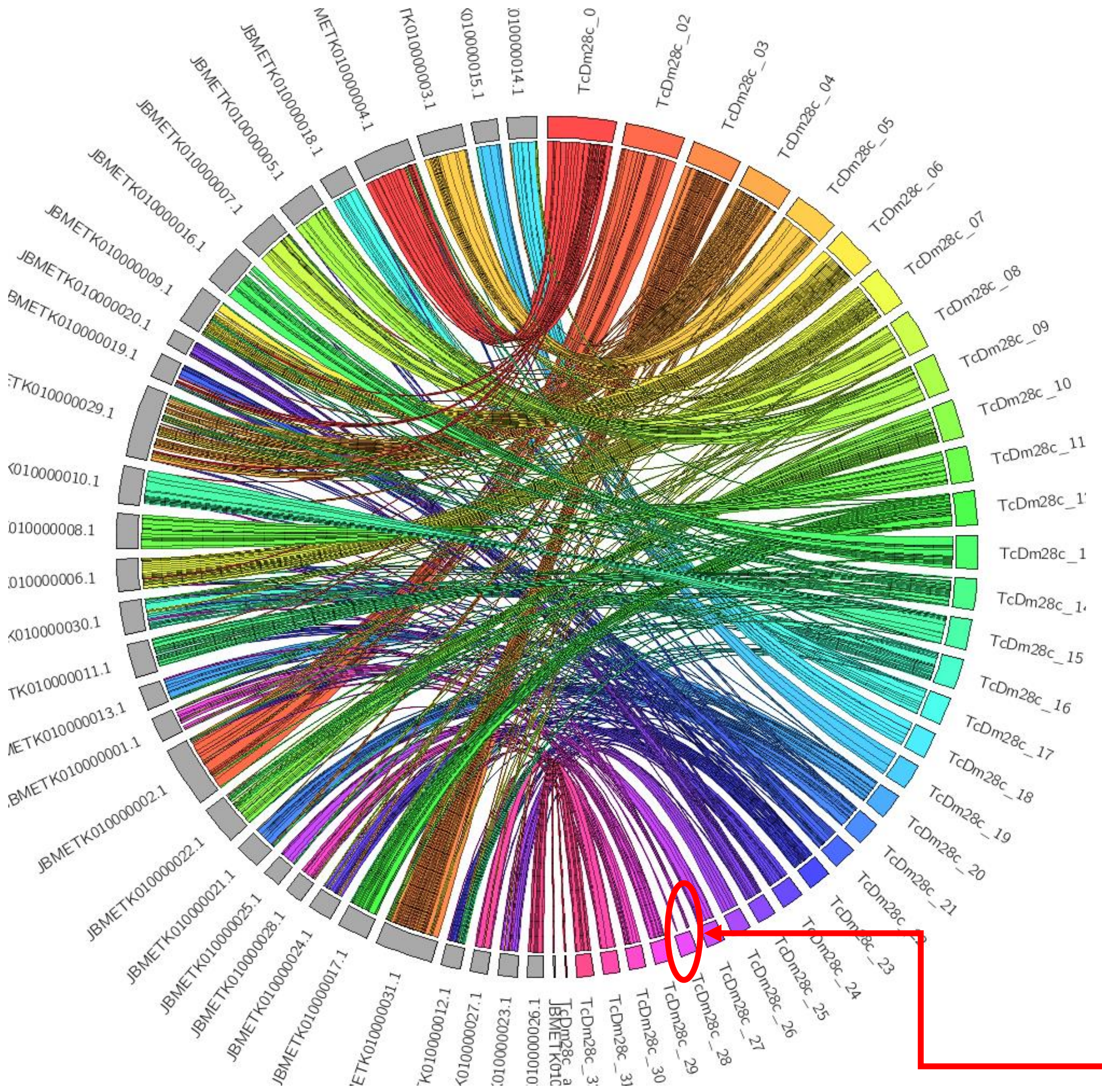

Chromosome 28 in Dm28c missing in Sylvio 2025 assembly

Is Chromosome 28 an artifact in Dm28c or is missing in Sylvio assembly?

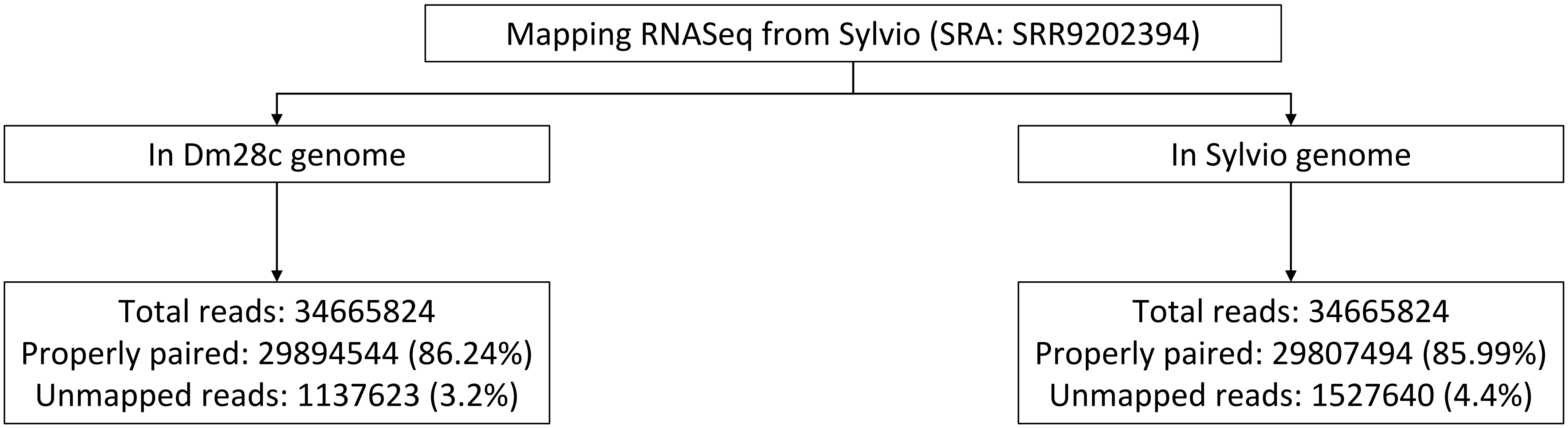

Evidence of missing Chromosome 28 in Sylvio assembly_

- Sylvio RNA-seq mapped to Dm28c genome: 1351853 (3.89%) reads map specifically to Chr28
- Sylvio RNA-seq uniq reads mapped to Dm28c Chr28: 270804 reads (250370 of these reads - 92.4%- not map to Sylvio genome).
- **Higher mapping efficiency**: Better properly paired rates in Dm28c
- **Reduced unmapped reads**: Fewer orphaned reads when using complete Dm28c reference

Visualize uniq mapped reads from Sylvio RNAseq in Dm28c Chromosome 28

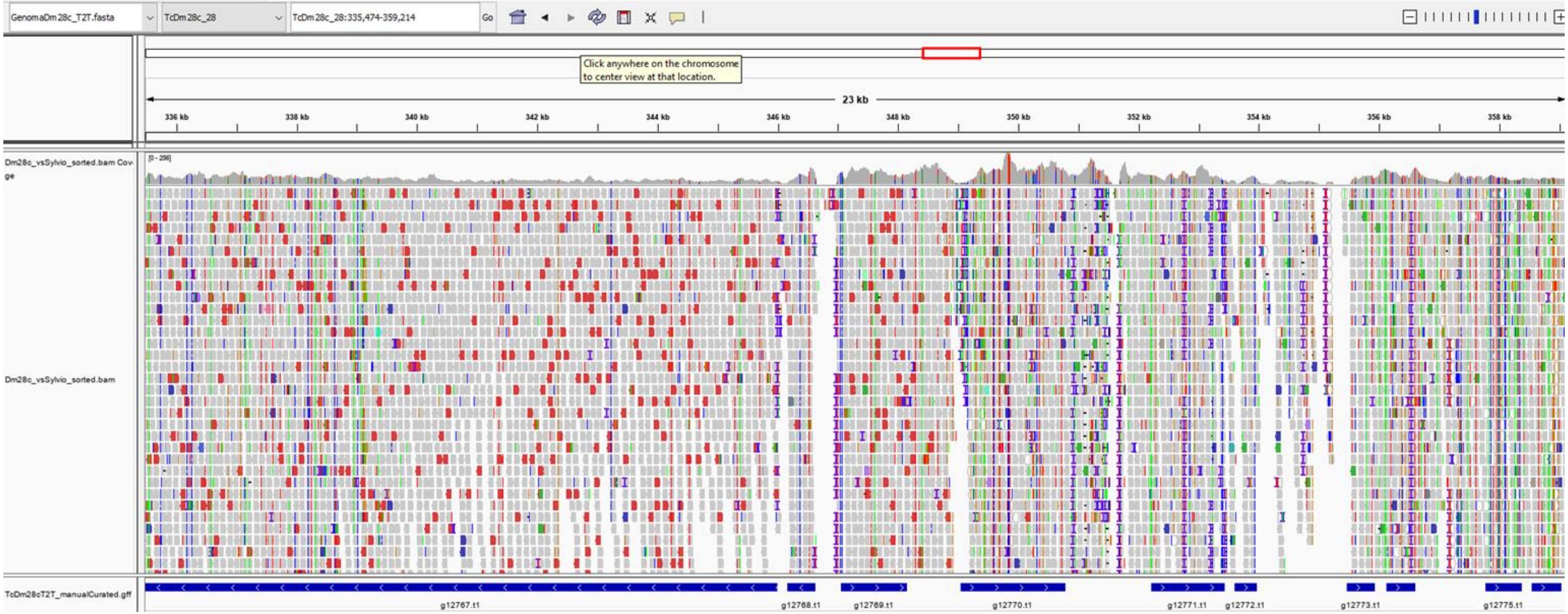

Circos plot representing the Sylvio X10 draft assembly (ADWP00000000.2, retrieved from NCBI in October 2025) against the TcDm28c T2T genome, highlighting exclusively the contigs that share homology with TcDm28c_Chr28.

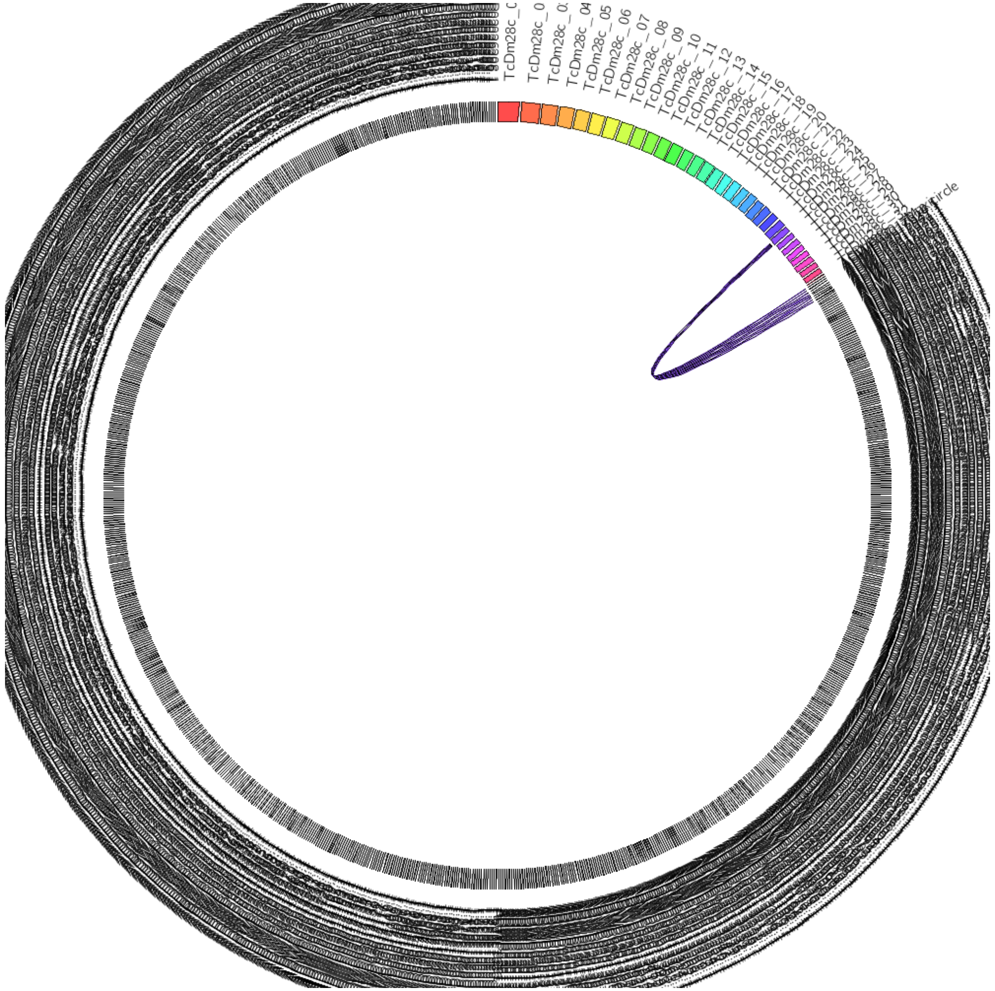

Supplement: Supplementary file 4 — Supplementary Material 4. [file 12864_2025_12482_MOESM4_ESM.pdf]
