# Supplementary material for: The complete genome of Trypanosoma cruzi reveals 32 chromosomes and three genomic compartments
Source: BMC Genomics. 2026 Jan 8;27:159. doi: 10.1186/s12864-025-12482-0 (PMC12879350; doi:10.1186/s12864-025-12482-0)

Supplementary Figure 5. A. Mean coverage per chromosome and haplotype. B. SNP coverage by chromosome

A.

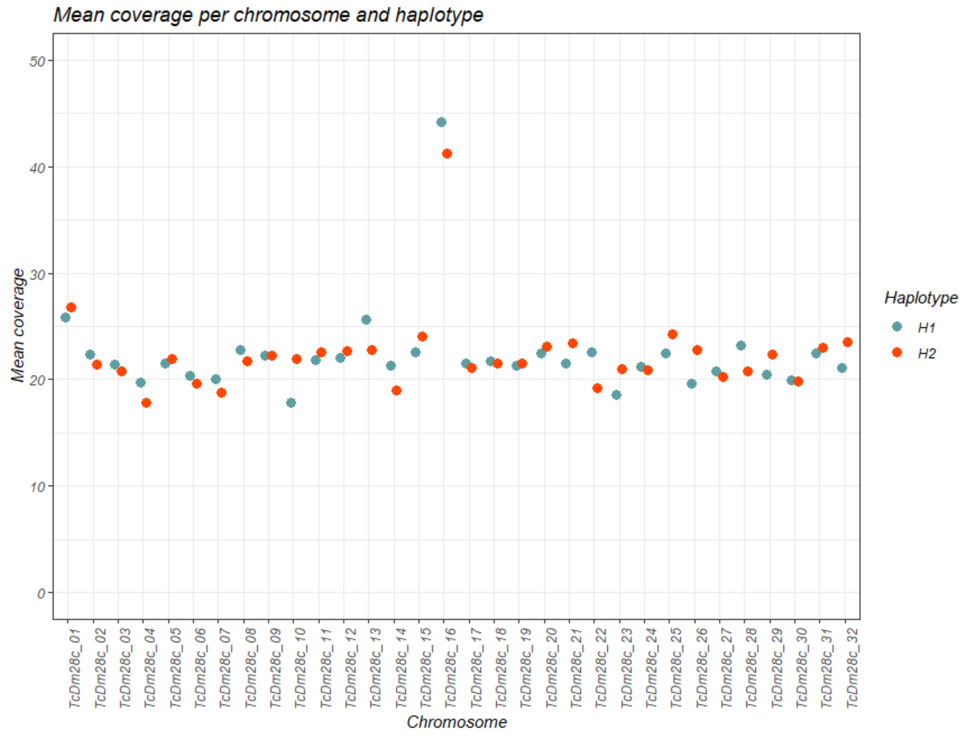

B.

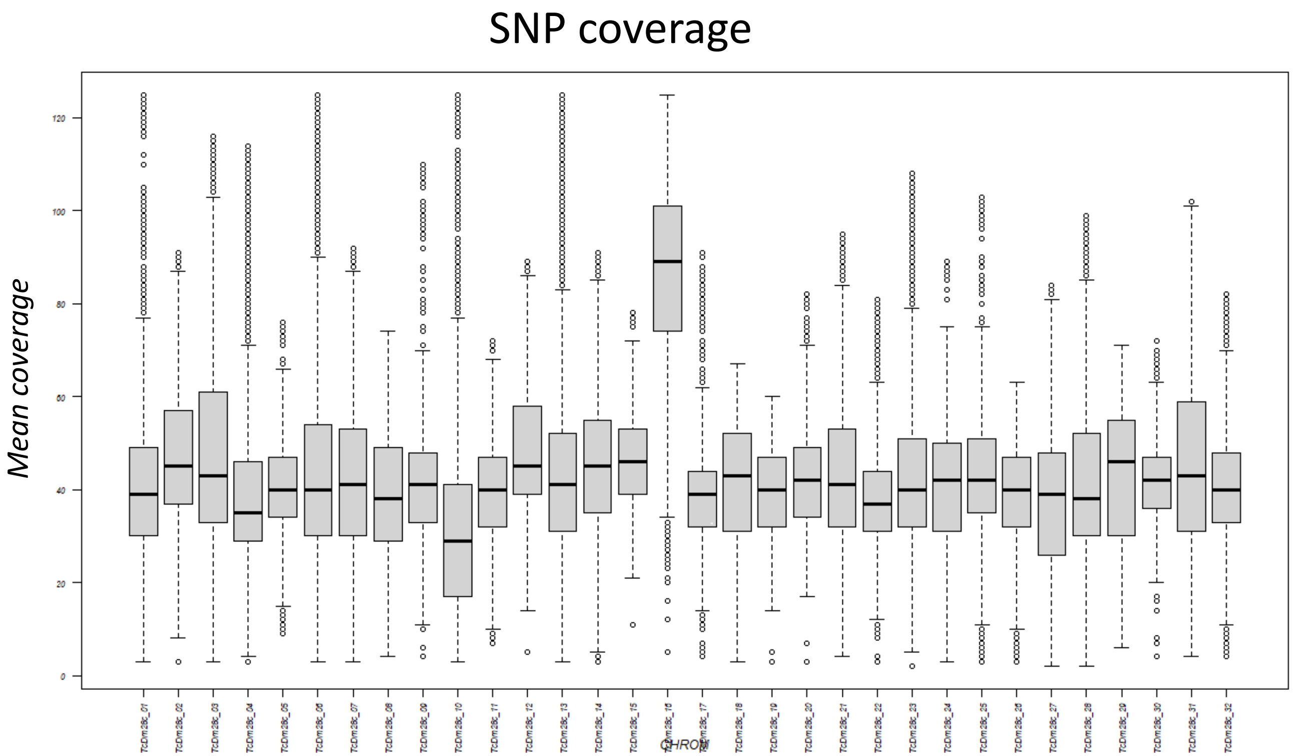

Supplement: Supplementary file 7 — Supplementary Material 7. [file 12864_2025_12482_MOESM7_ESM.pdf]
