# Supplementary material for: The complete genome of Trypanosoma cruzi reveals 32 chromosomes and three genomic compartments
Source: BMC Genomics. 2026 Jan 8;27:159. doi: 10.1186/s12864-025-12482-0 (PMC12879350; doi:10.1186/s12864-025-12482-0)
Supplement: Supplementary file 8 — Supplementary Material 8. [file 12864_2025_12482_MOESM8_ESM.pdf]

Supplementary Figure 8.

Gene proportions (Conserved, MASP/mucin, trans-sialidase and RHS) in 5 gene Windows across all chromosomes.

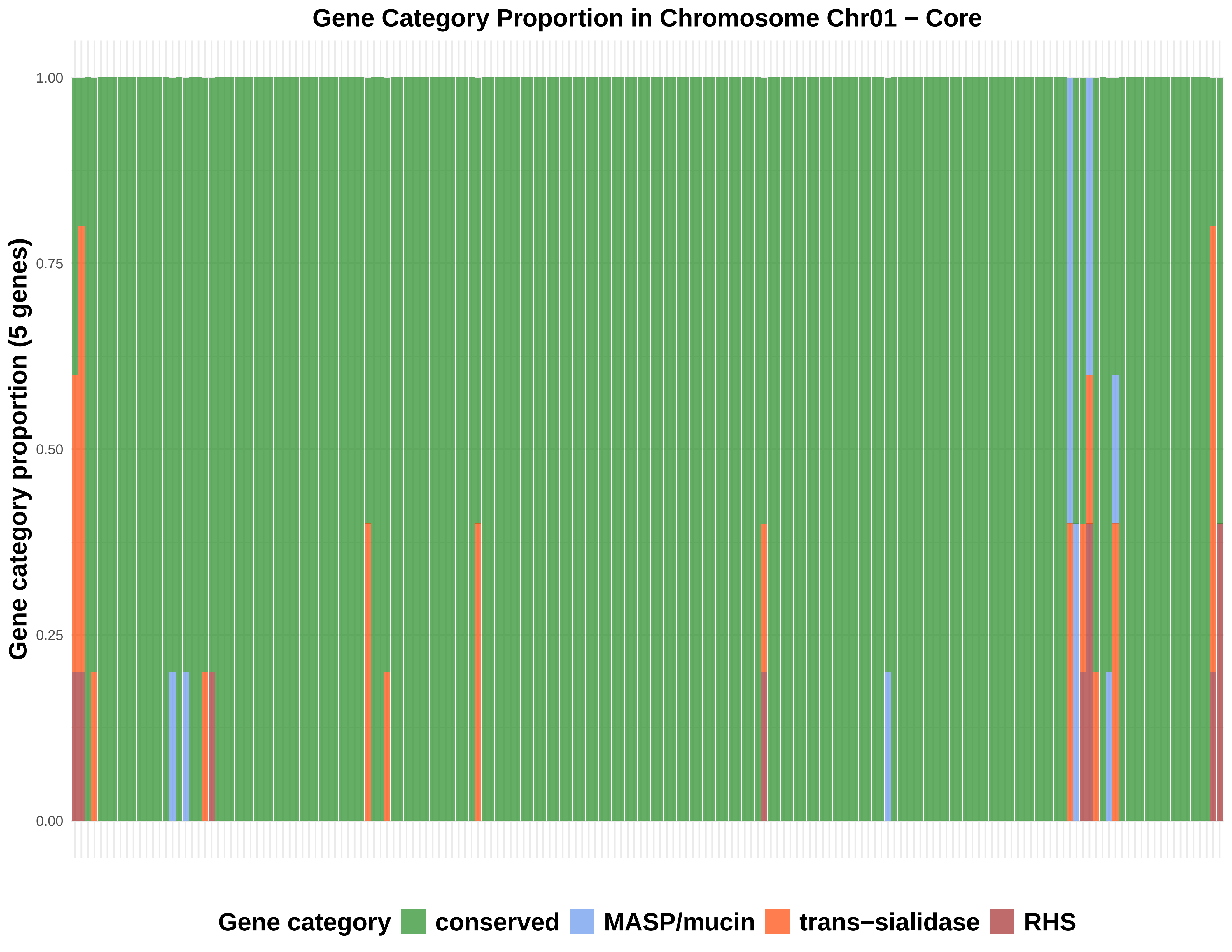

Gene Category Proportion in Chromosome Chr01 − Core
Gene category proportion (5 genes)
1.00
0.75
0.50
0.25
0.00
Gene category
conserved
MASP/mucin
trans−sialidase
RHS

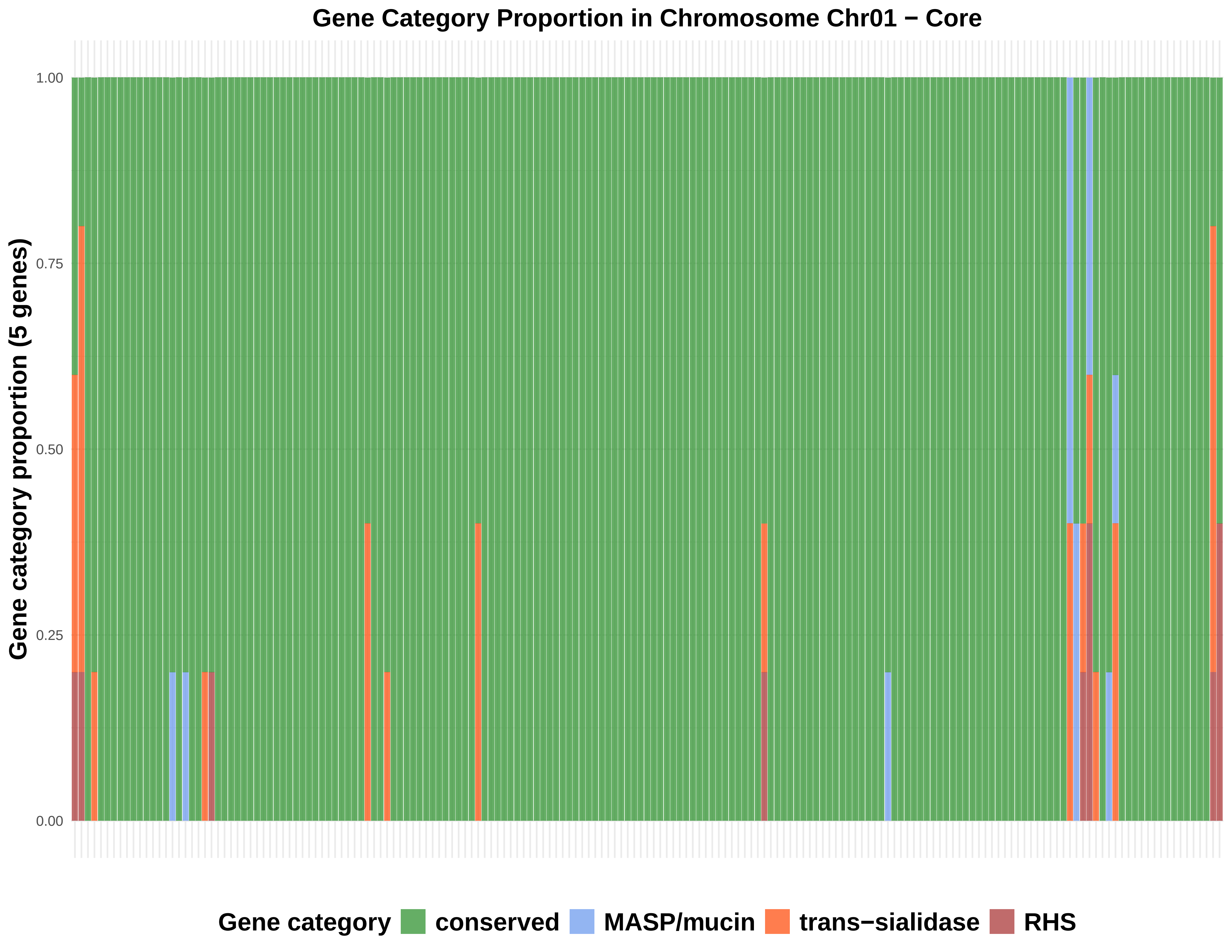
Gene Category Proportion in Chromosome Chr01 − Core
Gene category proportion (5 genes)
1.00
0.75
0.50
0.25
0.00
Gene category
conserved
MASP/mucin
trans−sialidase
RHS

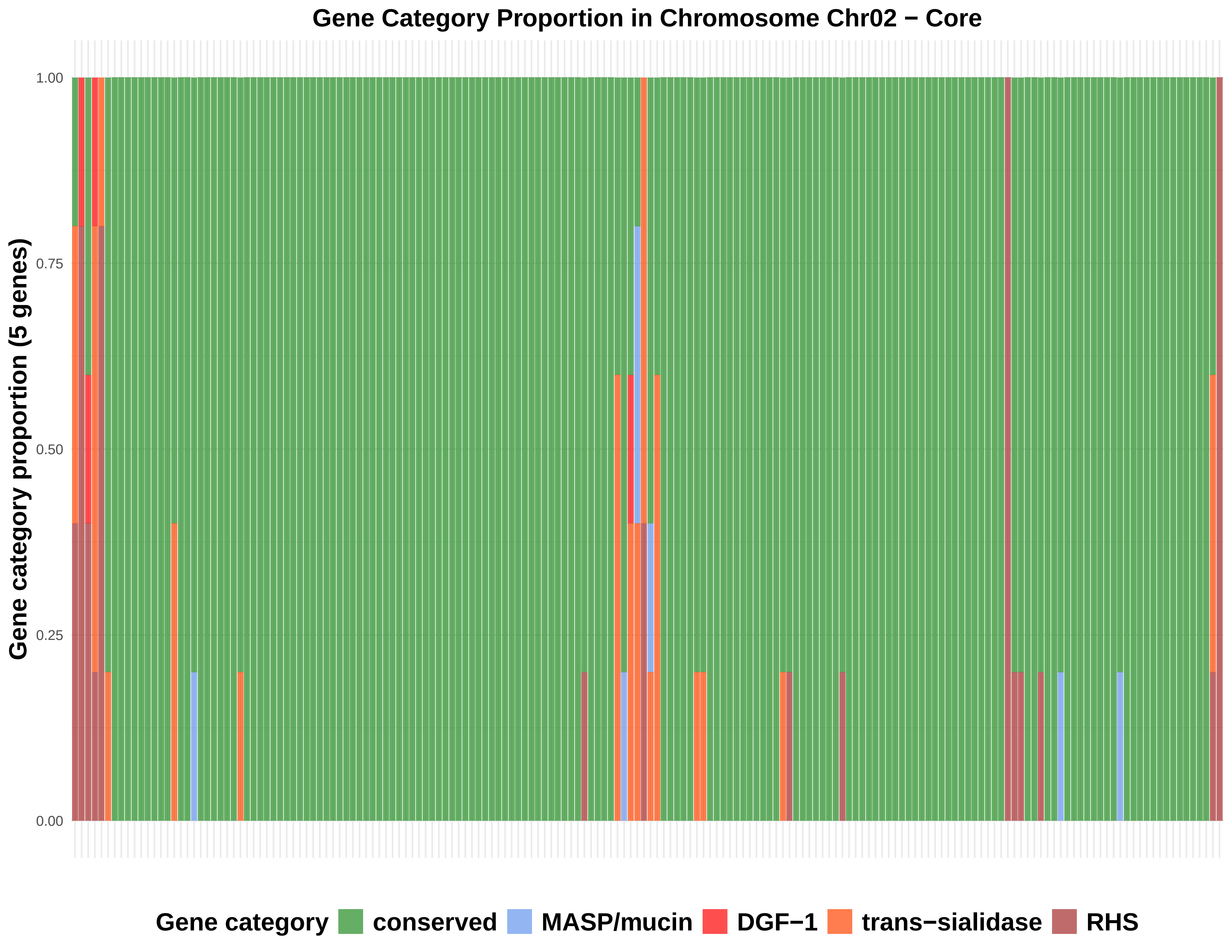

Gene Category Proportion in Chromosome Chr02 − Core
Gene category proportion (5 genes)
1.00
0.75
0.50
0.25
0.00
Gene category
conserved
MASP/mucin
DGF−1
trans−sialidase
RHS

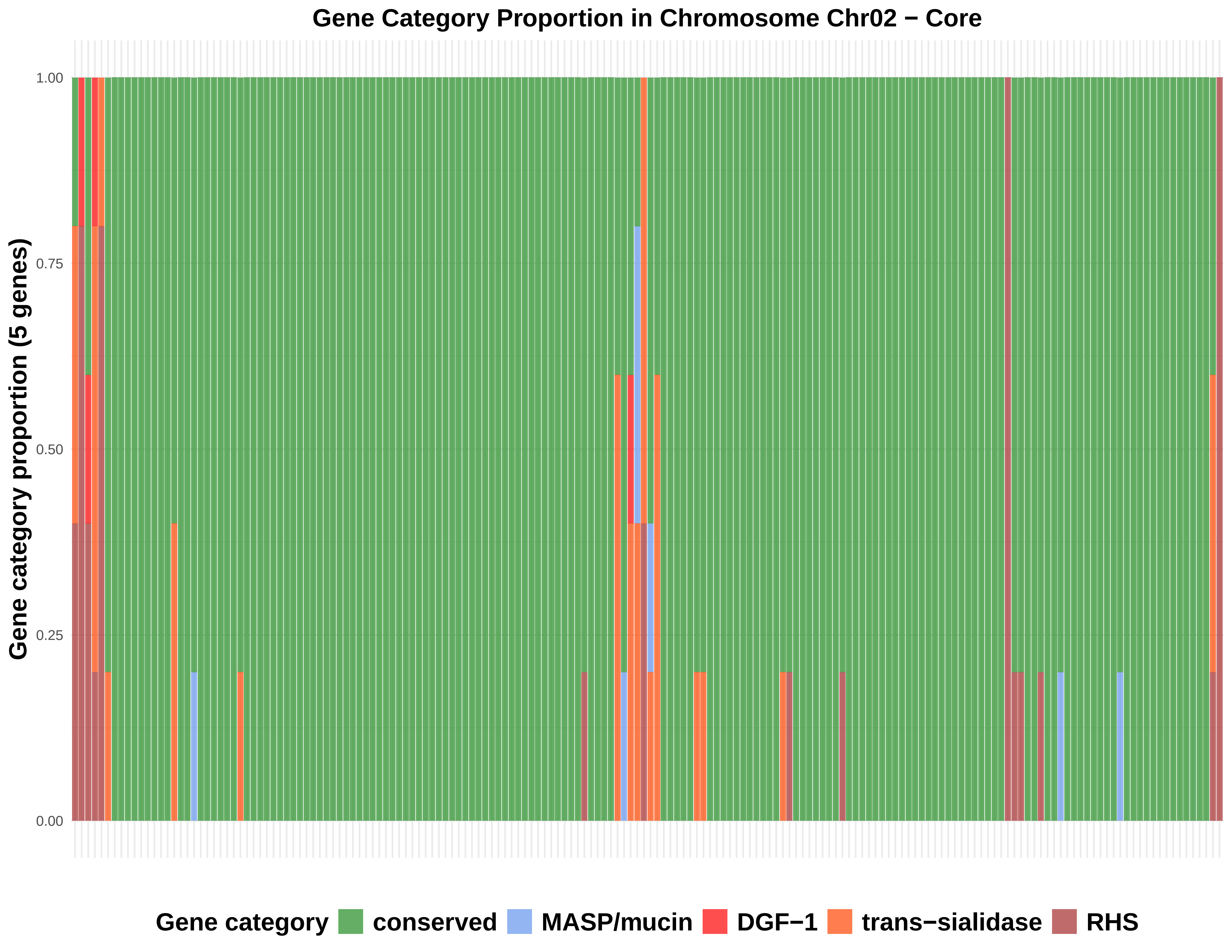

Gene Category Proportion in Chromosome Chr02 − Core
Gene category proportion (5 genes)
1.00
0.75
0.50
0.25
0.00
Gene category
conserved
MASP/mucin
DGF−1
trans−sialidase
RHS

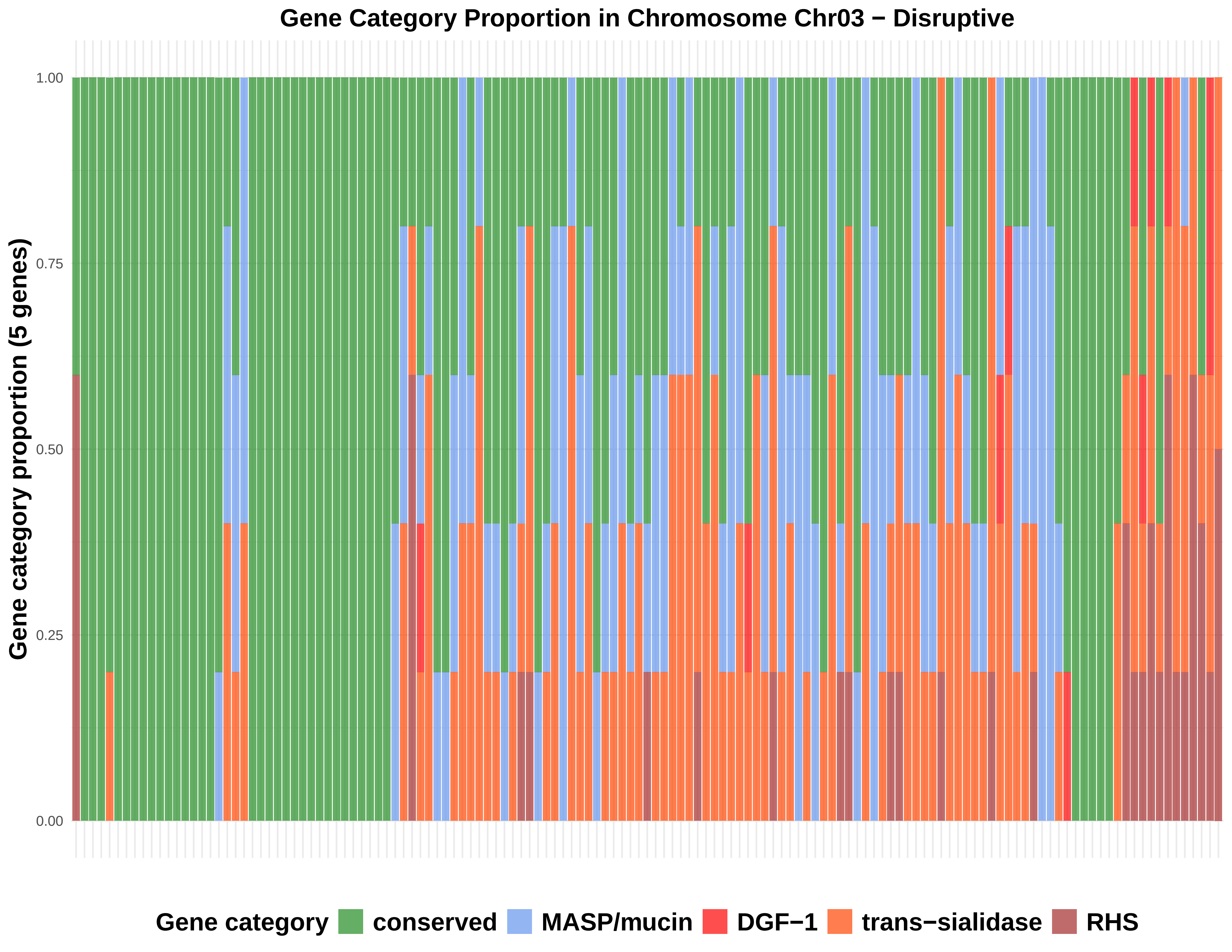
Gene Category Proportion in Chromosome Chr03 − Disruptive
Gene category proportion (5 genes)
1.00
0.75
0.50
0.25
0.00
Gene category
conserved
MASP/mucin
DGF−1
trans−sialidase
RHS

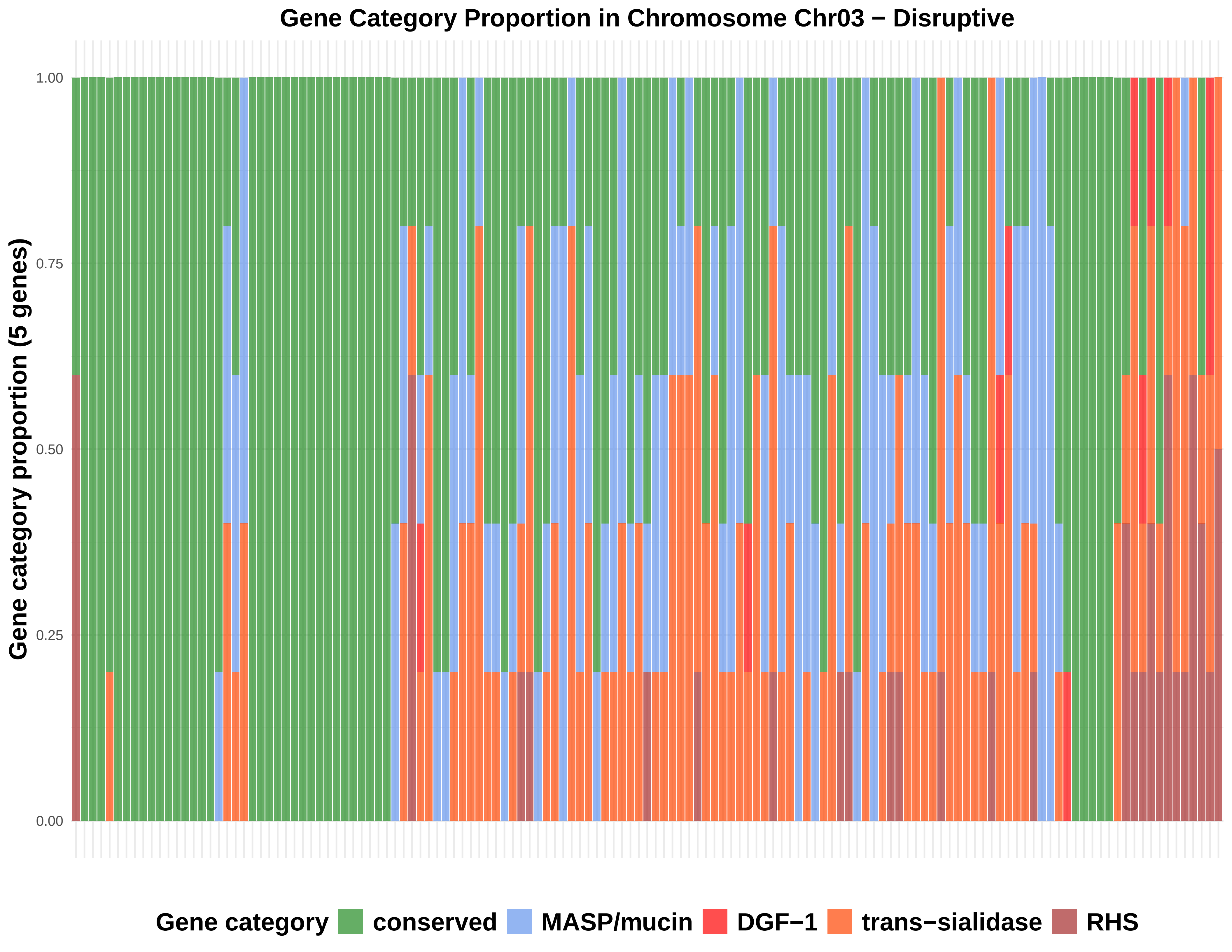

Gene Category Proportion in Chromosome Chr03 − Disruptive
Gene category proportion (5 genes)
1.00
0.75
0.50
0.25
0.00
Gene category
conserved
MASP/mucin
DGF−1
trans−sialidase
RHS

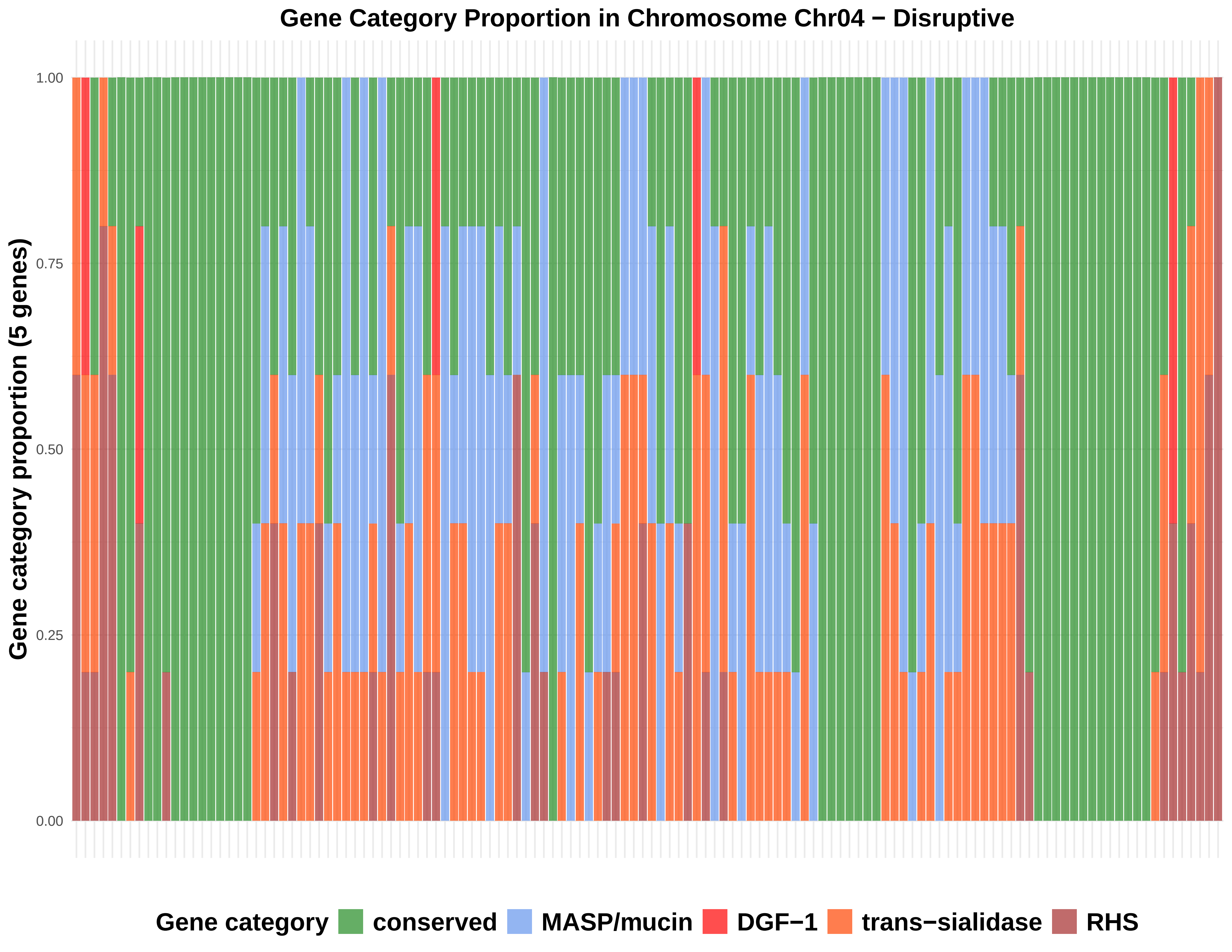
Gene Category Proportion in Chromosome Chr04 − Disruptive
Gene category proportion (5 genes)
1.00
0.75
0.50
0.25
0.00
Gene category
conserved
MASP/mucin
DGF−1
trans−sialidase
RHS

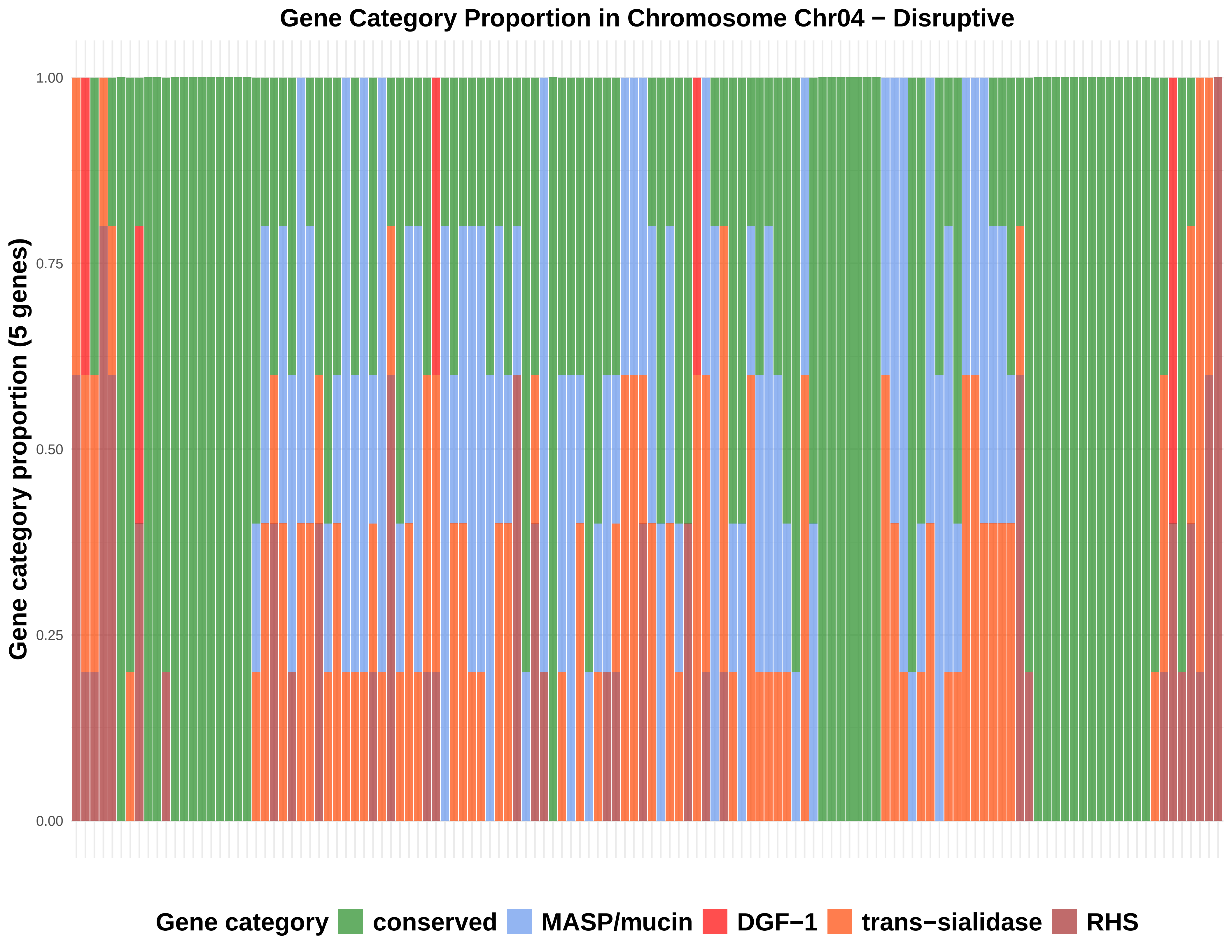
Gene Category Proportion in Chromosome Chr04 − Disruptive
Gene category proportion (5 genes)
1.00
0.75
0.50
0.25
0.00
Gene category
conserved
MASP/mucin
DGF−1
trans−sialidase
RHS

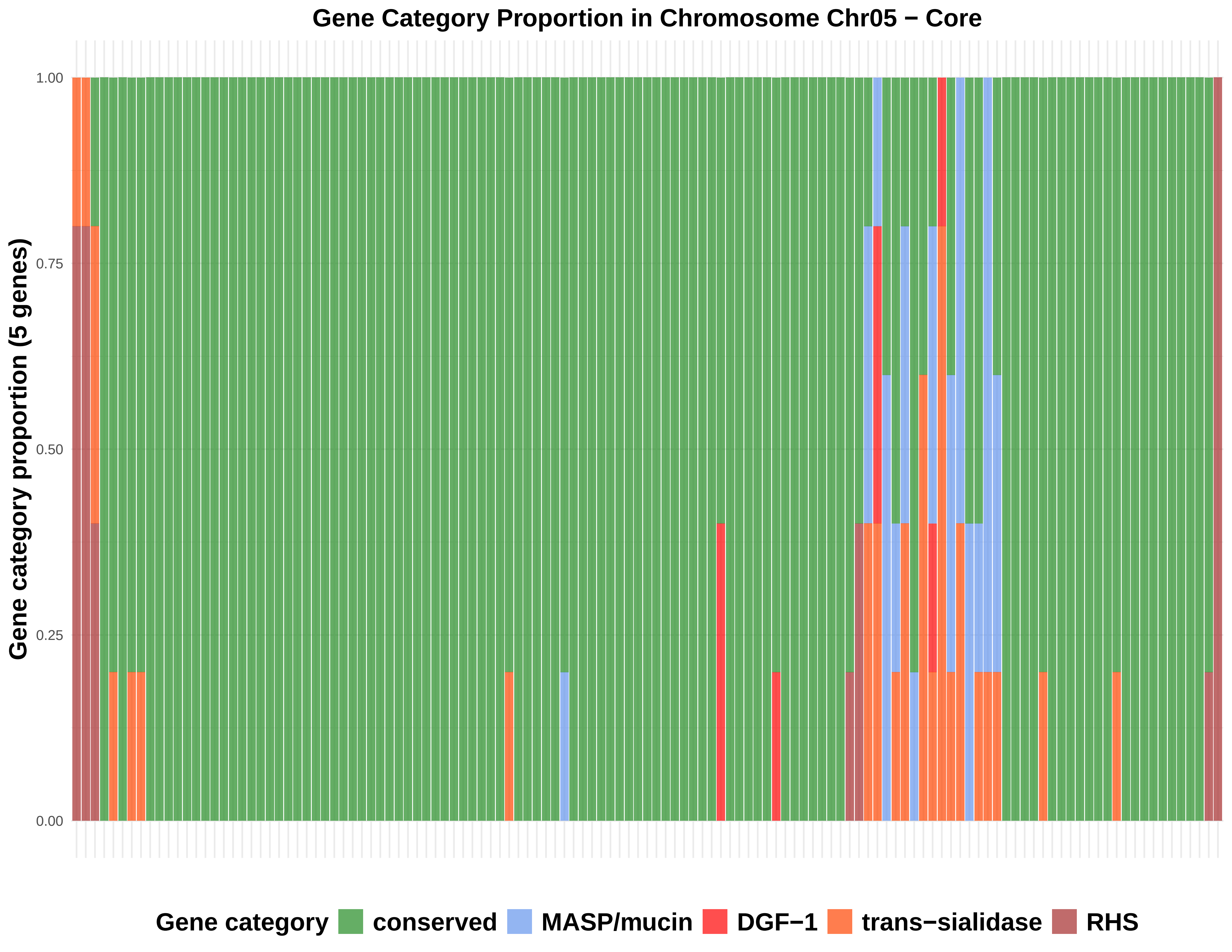

Gene Category Proportion in Chromosome Chr05 − Core
Gene category proportion (5 genes)
1.00
0.75
0.50
0.25
0.00
Gene category
conserved
MASP/mucin
DGF−1
trans−sialidase
RHS

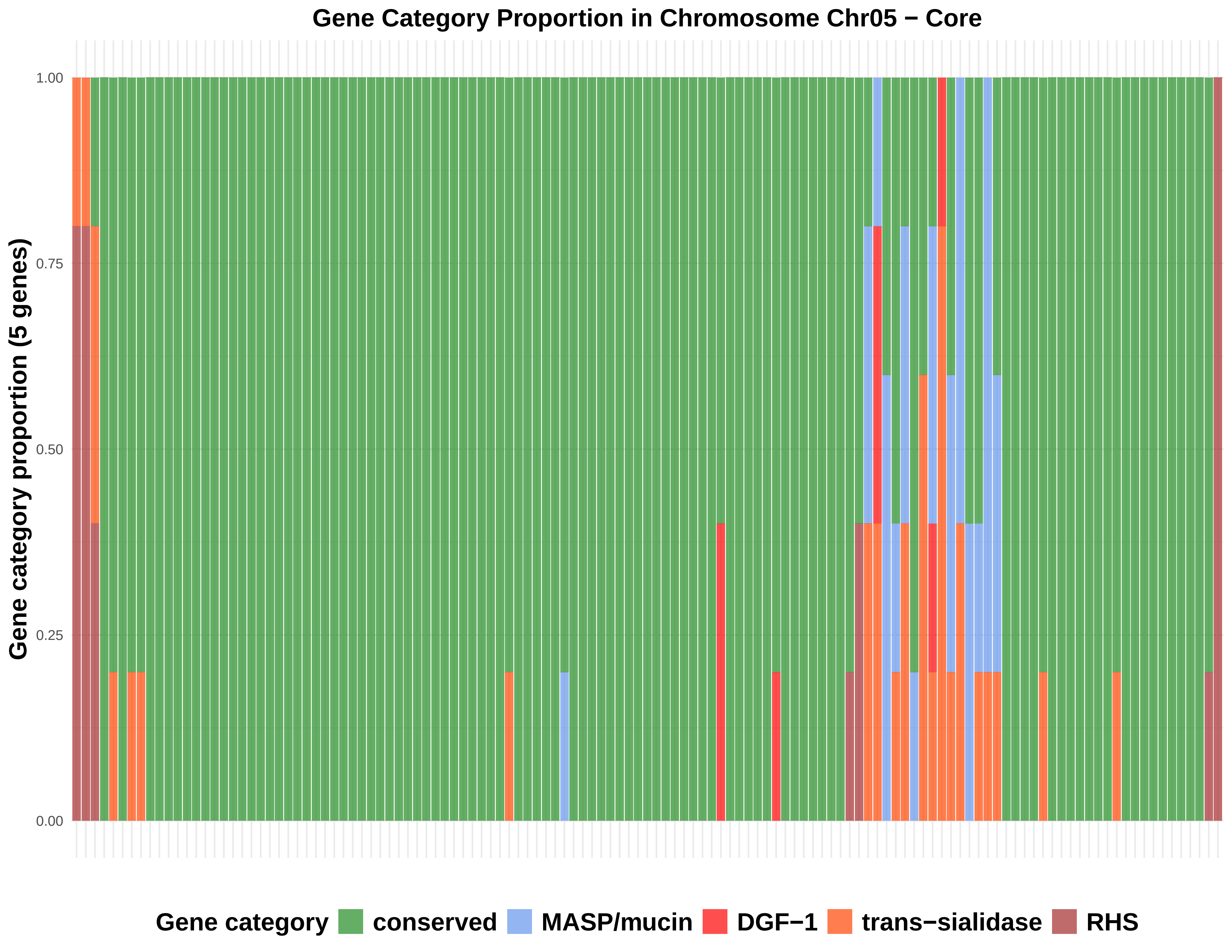
Gene Category Proportion in Chromosome Chr05 − Core
Gene category proportion (5 genes)
1.00
0.75
0.50
0.25
0.00
Gene category
conserved
MASP/mucin
DGF−1
trans−sialidase
RHS

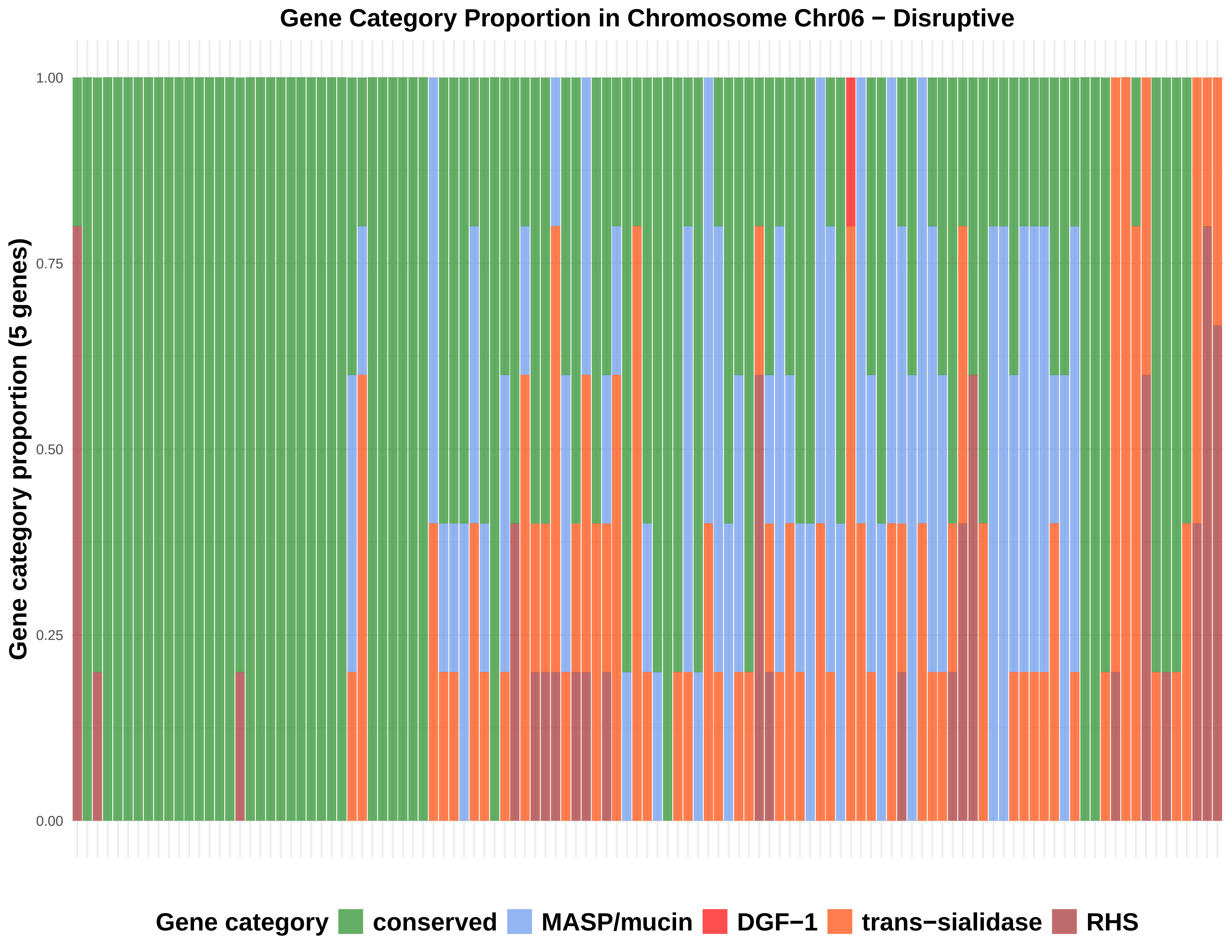
Gene Category Proportion in Chromosome Chr06 − Disruptive
Gene category proportion (5 genes)
1.00
0.75
0.50
0.25
0.00
Gene category
conserved
MASP/mucin
DGF−1
trans−sialidase
RHS

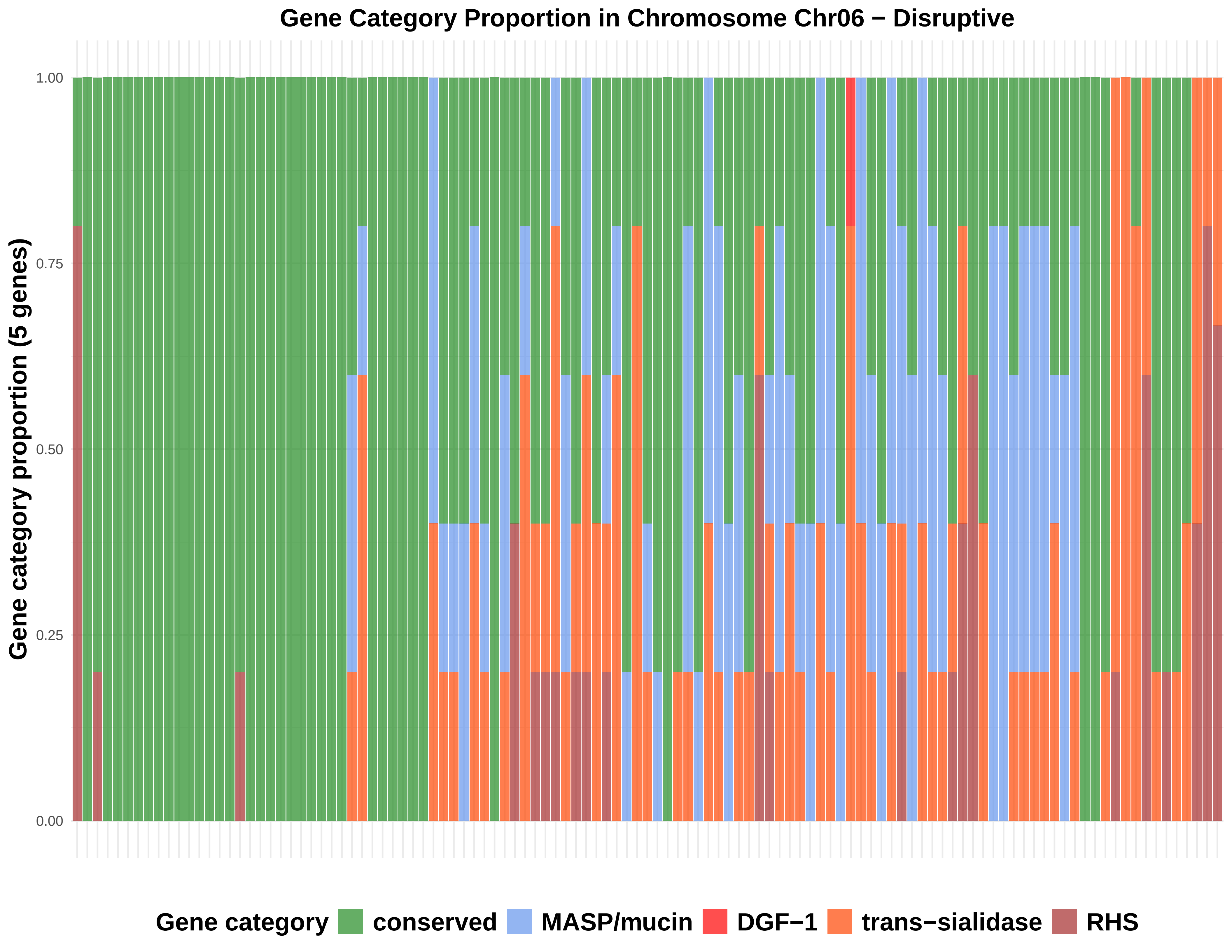
Gene Category Proportion in Chromosome Chr06 − Disruptive
Gene category proportion (5 genes)
1.00
0.75
0.50
0.25
0.00
Gene category
conserved
MASP/mucin
DGF−1
trans−sialidase
RHS

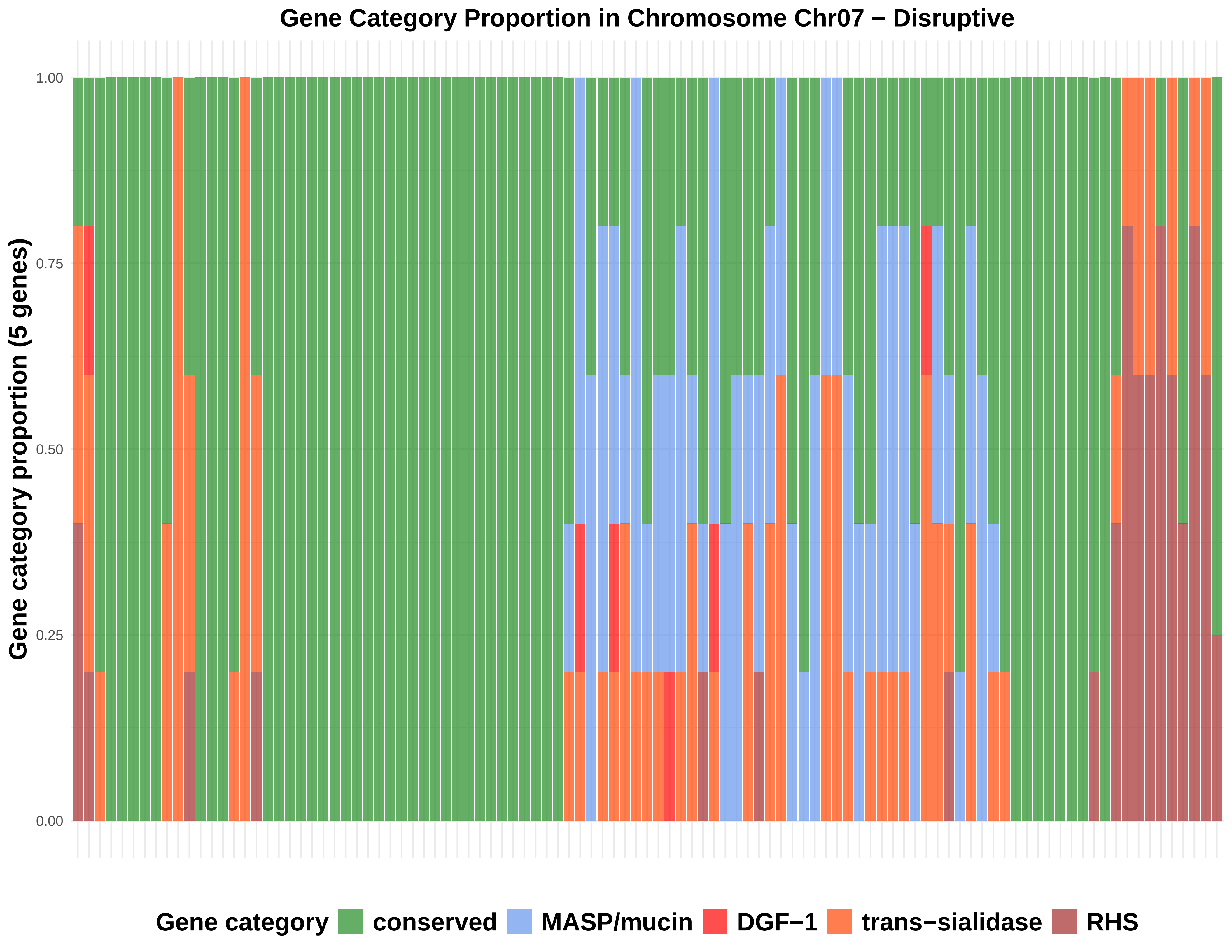

Gene Category Proportion in Chromosome Chr07 − Disruptive
Gene category proportion (5 genes)
1.00
0.75
0.50
0.25
0.00
Gene category
conserved
MASP/mucin
DGF−1
trans−sialidase
RHS

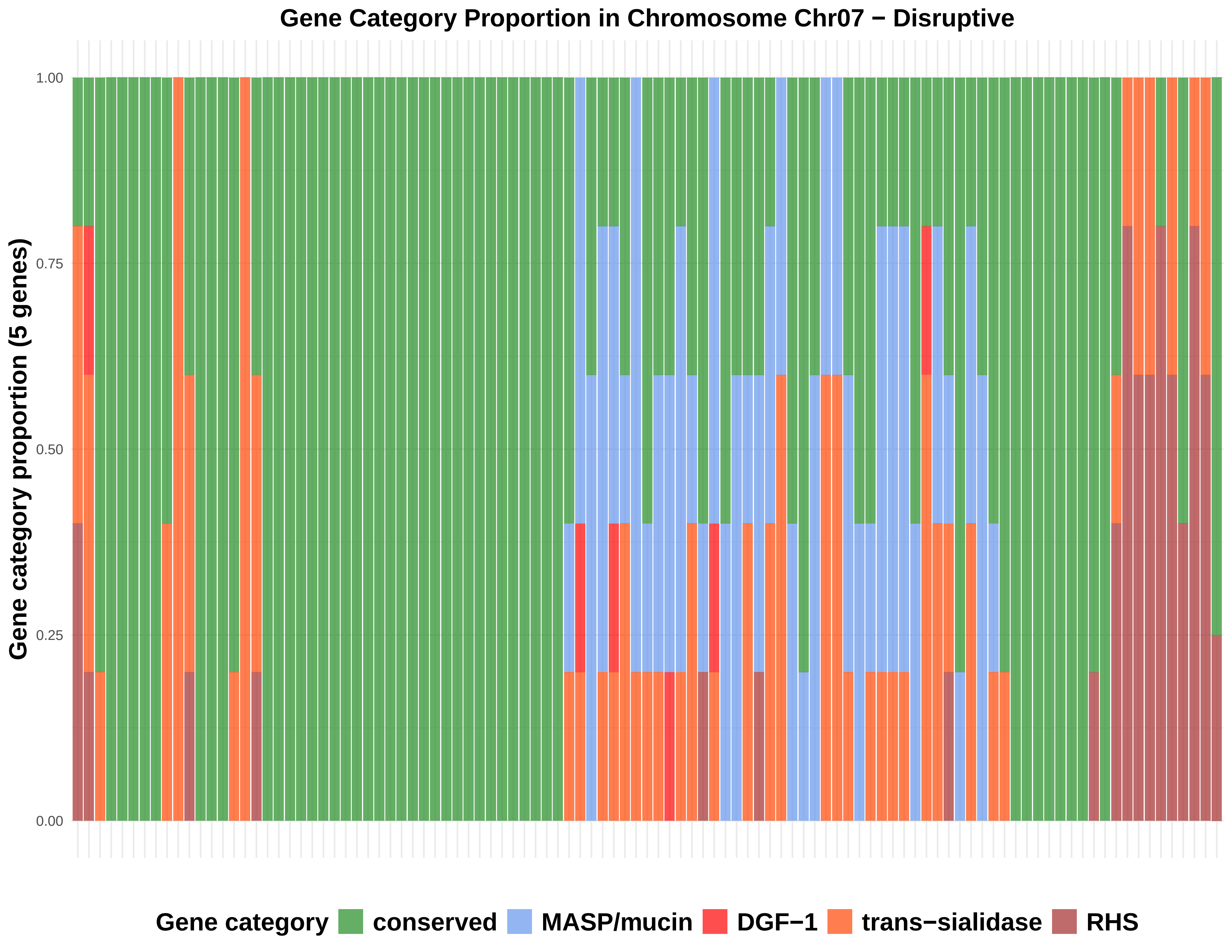

Gene Category Proportion in Chromosome Chr07 − Disruptive
Gene category proportion (5 genes)
1.00
0.75
0.50
0.25
0.00
Gene category
conserved
MASP/mucin
DGF−1
trans−sialidase
RHS

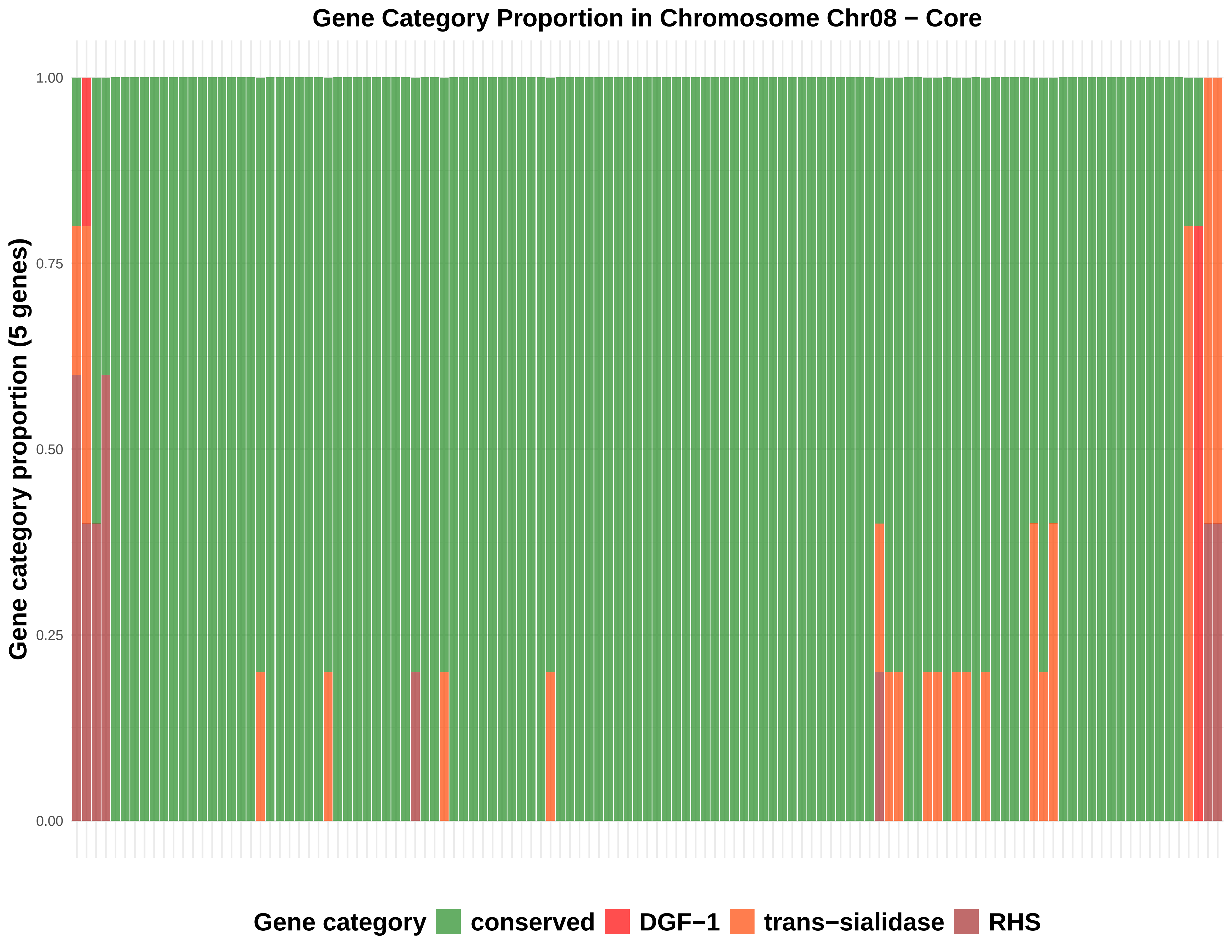
Gene Category Proportion in Chromosome Chr08 − Core
Gene category proportion (5 genes)
1.00
0.75
0.50
0.25
0.00
Gene category
conserved
DGF−1
trans−sialidase
RHS

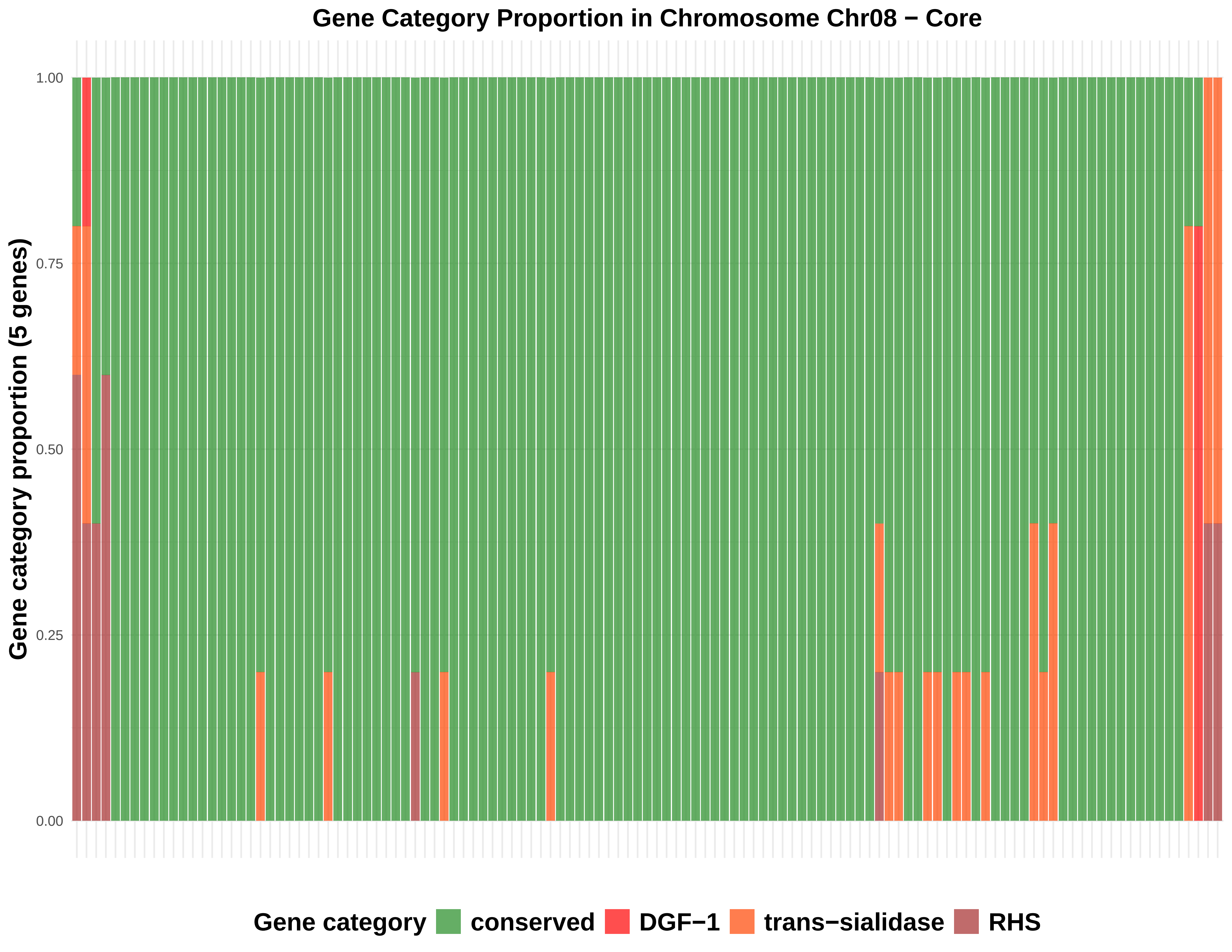

Gene Category Proportion in Chromosome Chr08 − Core
Gene category proportion (5 genes)
1.00
0.75
0.50
0.25
0.00
Gene category
conserved
DGF−1
trans−sialidase
RHS

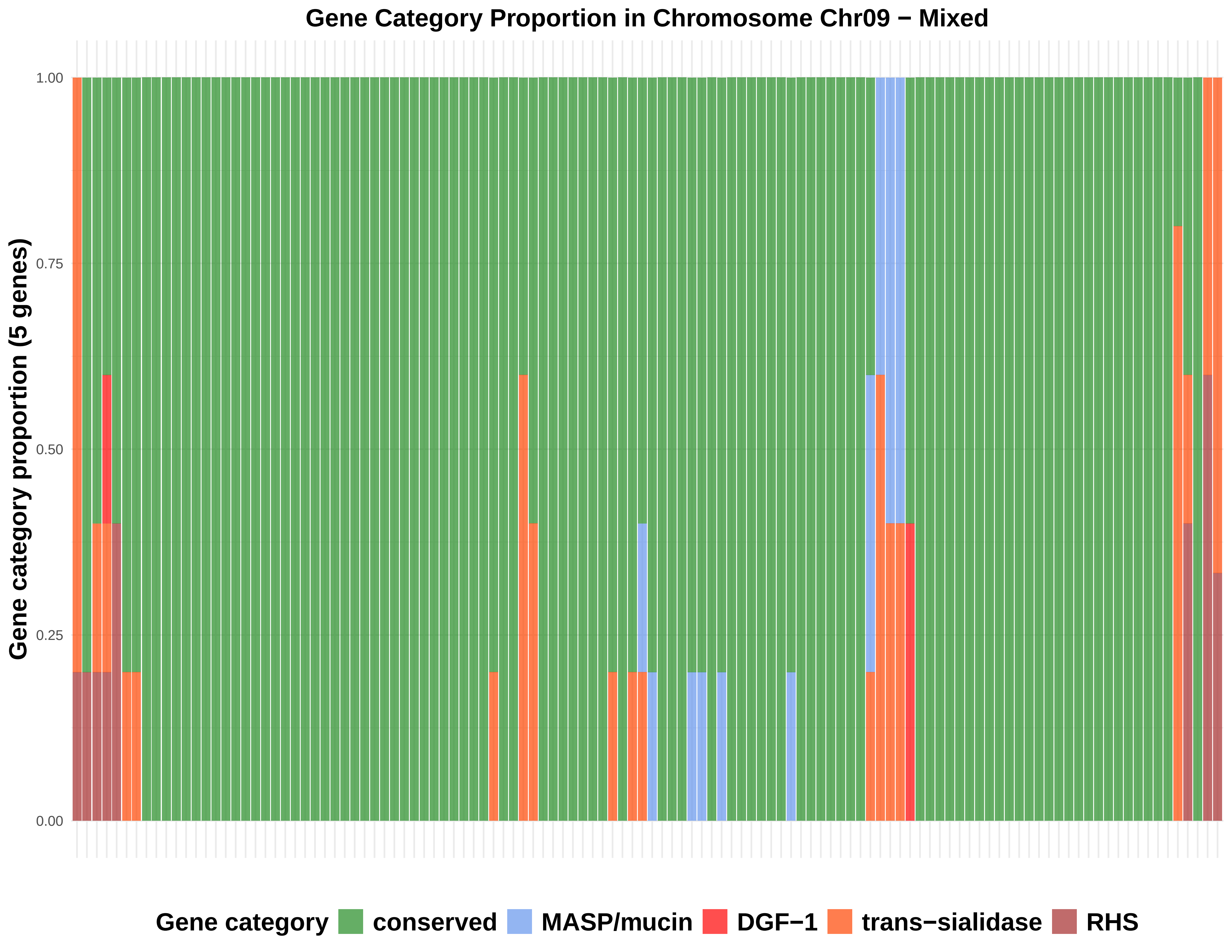

Gene Category Proportion in Chromosome Chr09 − Mixed
Gene category proportion (5 genes)
1.00
0.75
0.50
0.25
0.00
Gene category
conserved
MASP/mucin
DGF−1
trans−sialidase
RHS

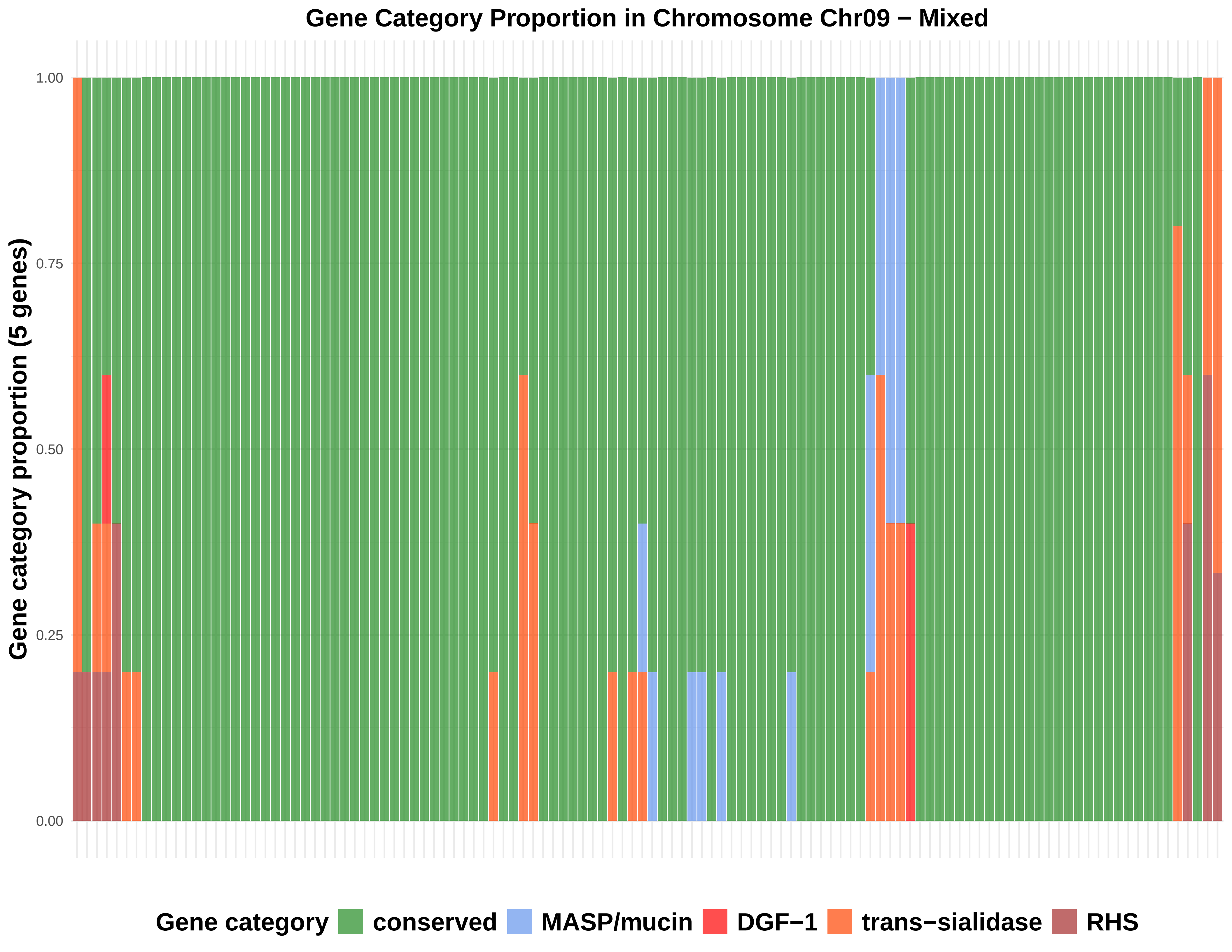
Gene Category Proportion in Chromosome Chr09 − Mixed
Gene category proportion (5 genes)
1.00
0.75
0.50
0.25
0.00
Gene category
conserved
MASP/mucin
DGF−1
trans−sialidase
RHS

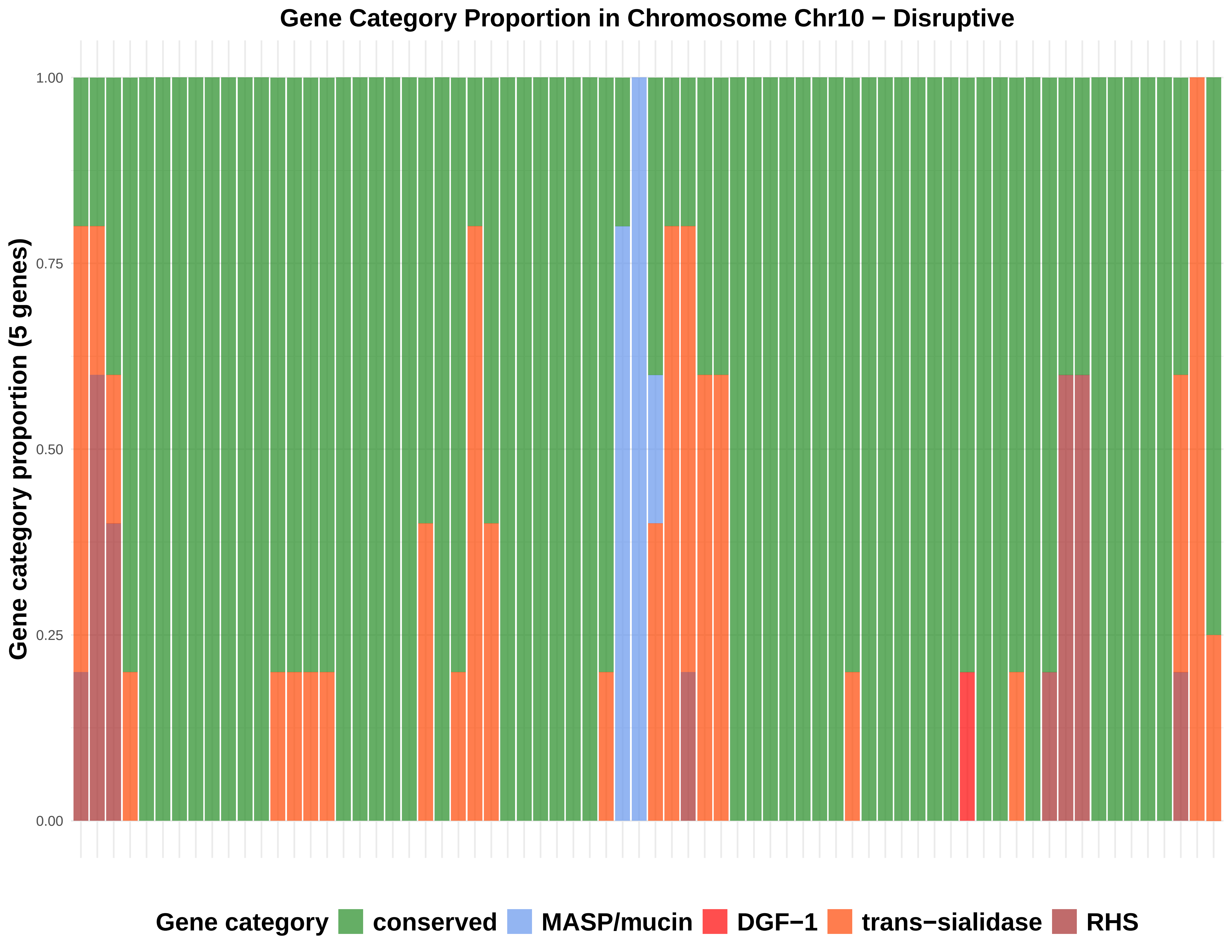
Gene Category Proportion in Chromosome Chr10 − Disruptive
Gene category proportion (5 genes)
1.00
0.75
0.50
0.25
0.00
Gene category
conserved
MASP/mucin
DGF−1
trans−sialidase
RHS

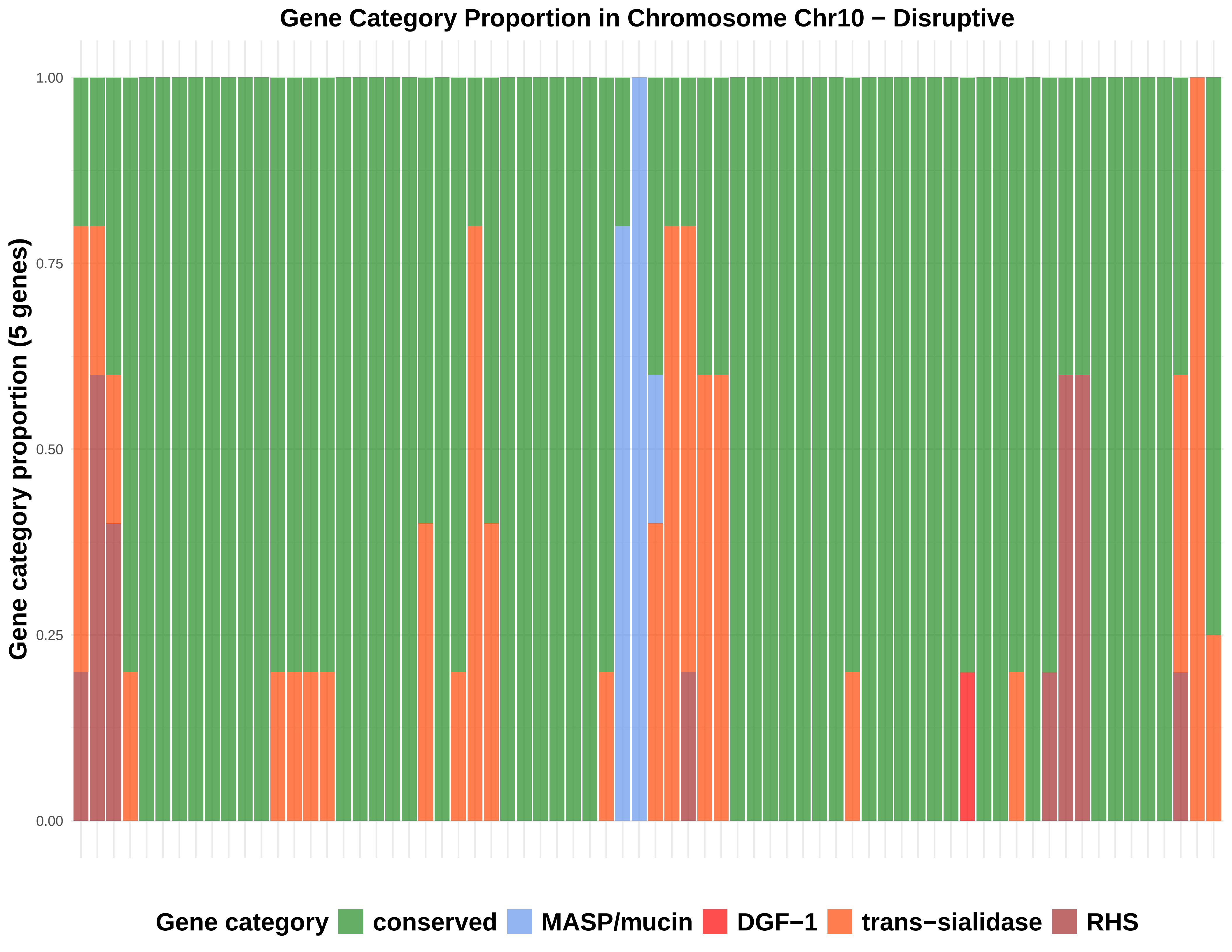

Gene Category Proportion in Chromosome Chr10 − Disruptive
Gene category proportion (5 genes)
1.00
0.75
0.50
0.25
0.00
Gene category
conserved
MASP/mucin
DGF−1
trans−sialidase
RHS

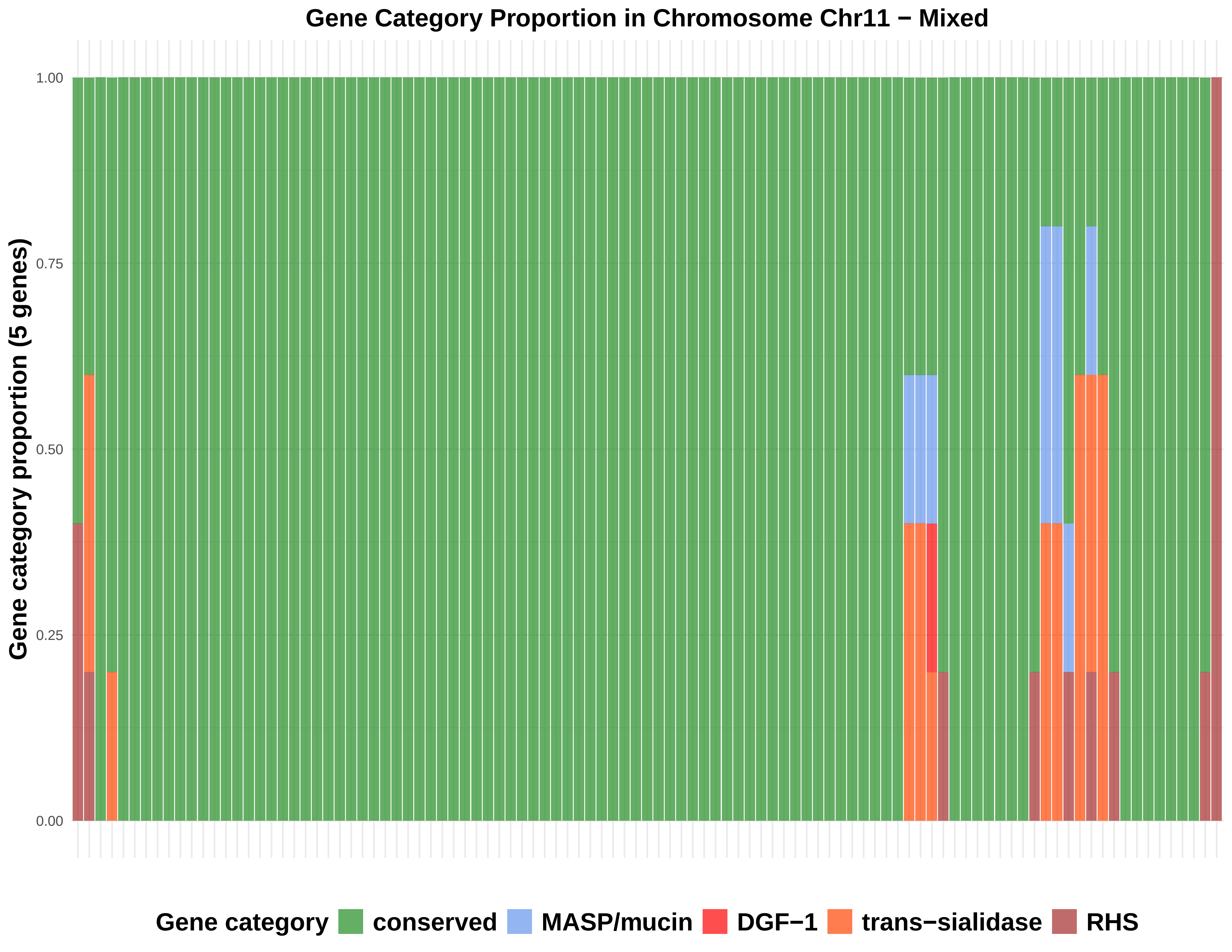
Gene Category Proportion in Chromosome Chr11 − Mixed
Gene category proportion (5 genes)
1.00
0.75
0.50
0.25
0.00
Gene category
conserved
MASP/mucin
DGF−1
trans−sialidase
RHS

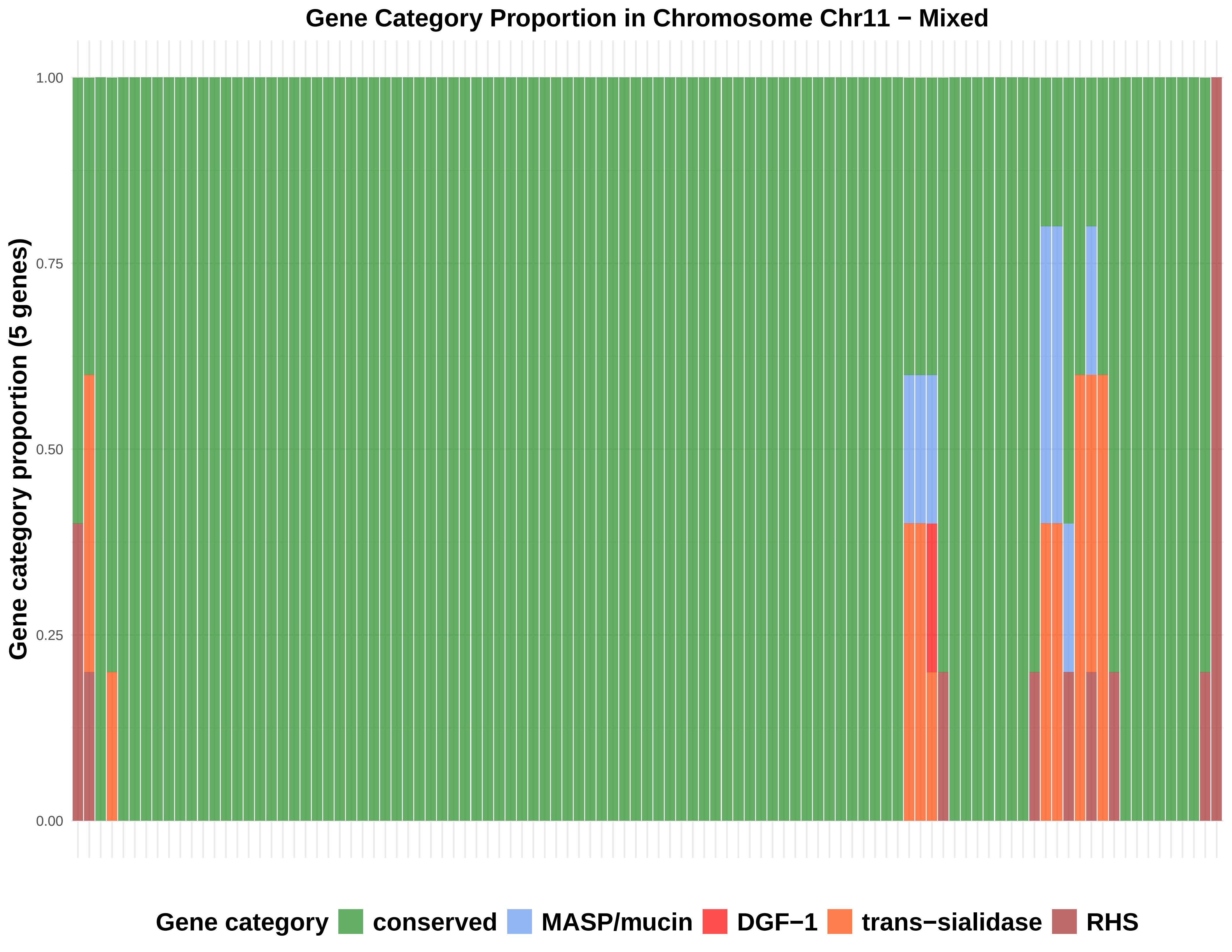

Gene Category Proportion in Chromosome Chr11 − Mixed
Gene category proportion (5 genes)
1.00
0.75
0.50
0.25
0.00
Gene category
conserved
MASP/mucin
DGF−1
trans−sialidase
RHS

Gene Category Proportion in Chromosome Chr12 − Disruptive

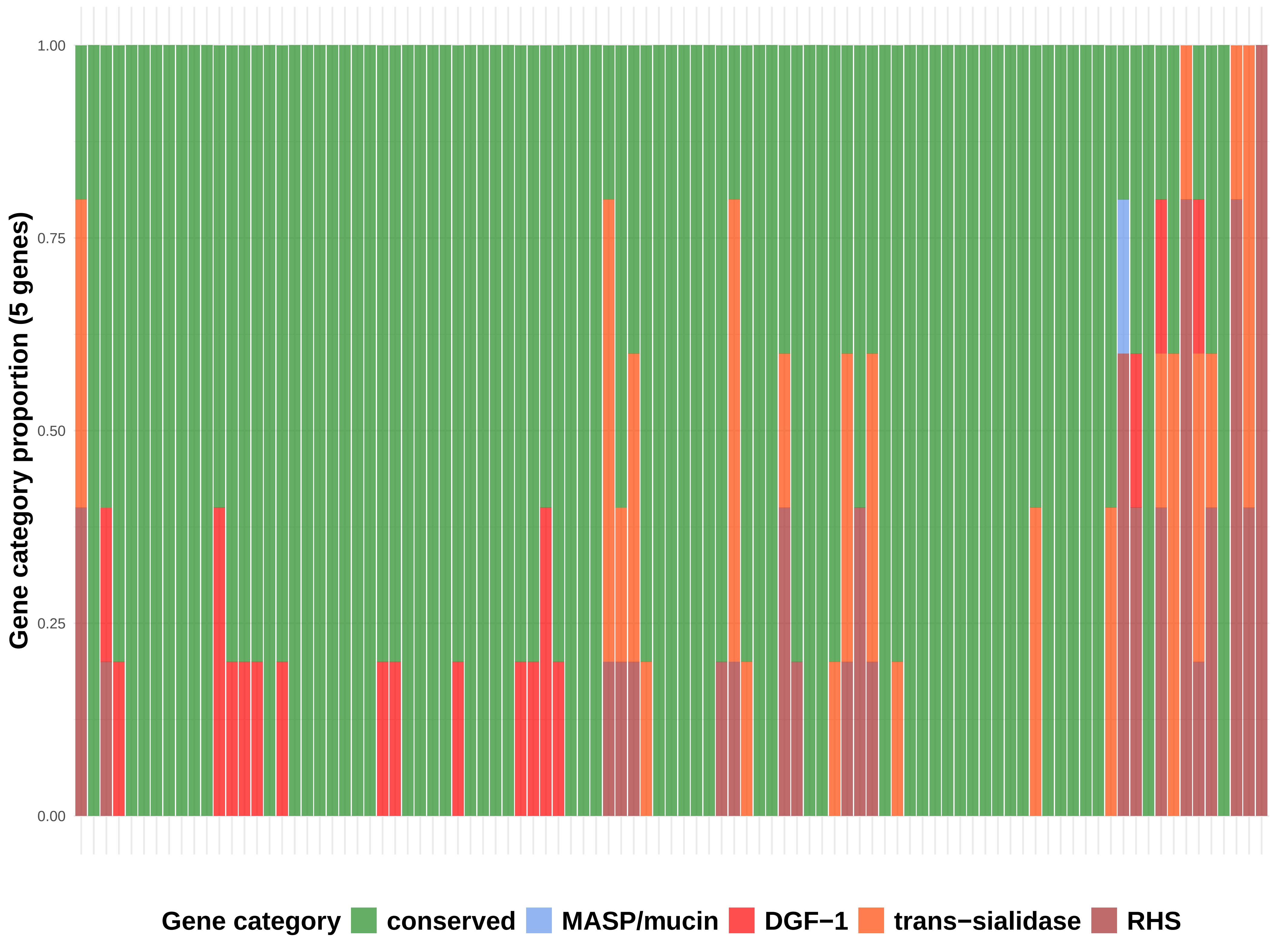

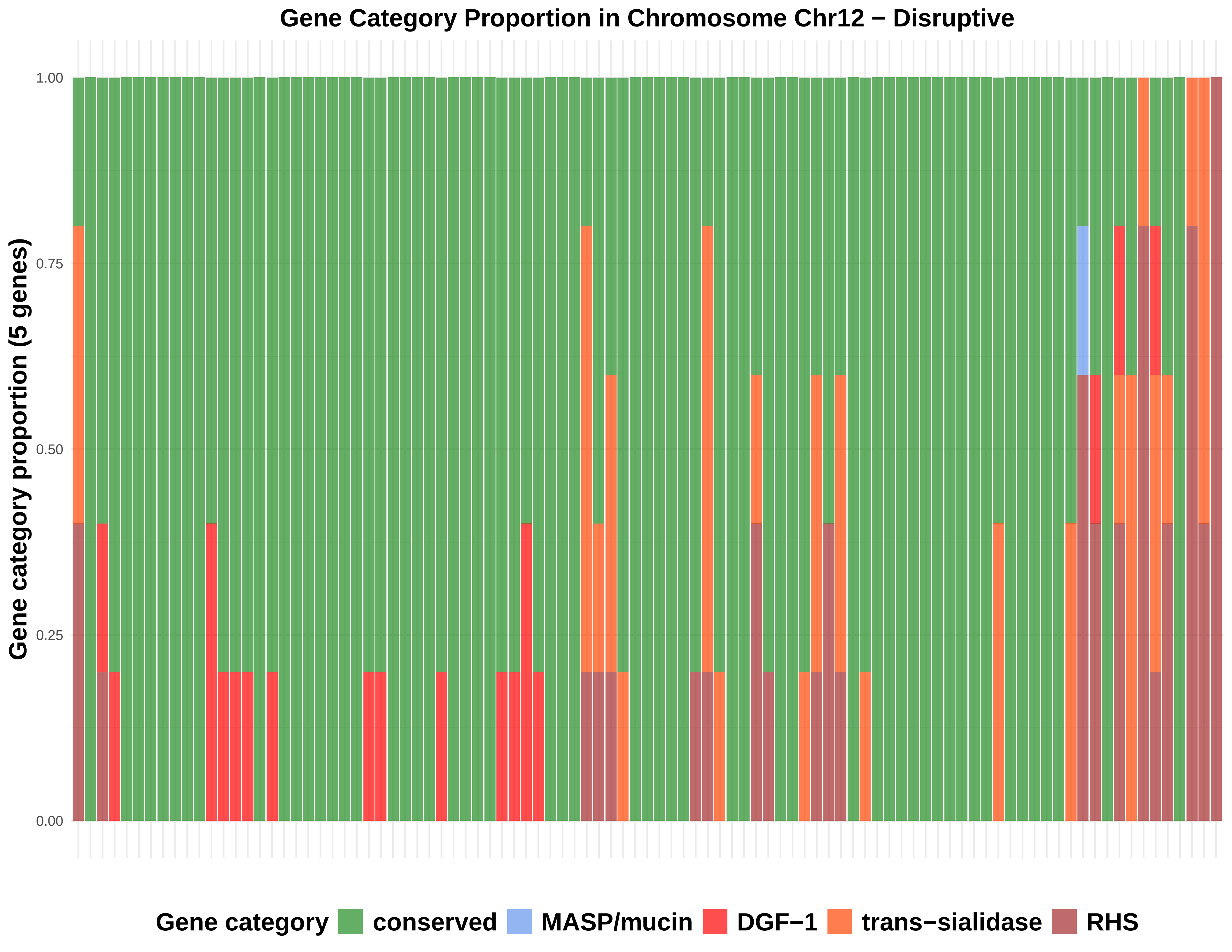

Gene Category Proportion in Chromosome Chr12 − Disruptive
Gene category proportion (5 genes)
1.00
0.75
0.50
0.25
0.00
Gene category
conserved
MASP/mucin
DGF−1
trans−sialidase
RHS

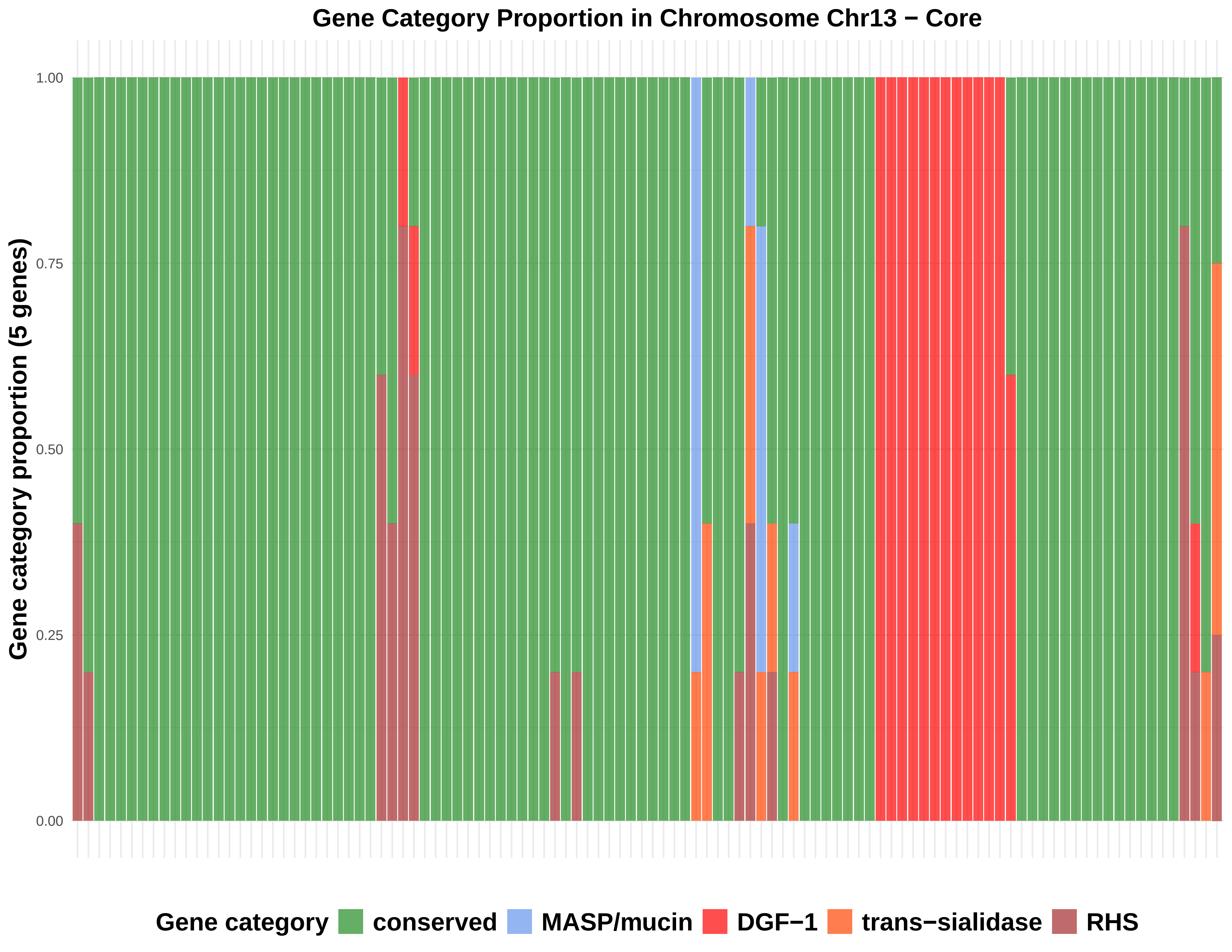

Gene Category Proportion in Chromosome Chr13 − Core
Gene category proportion (5 genes)
1.00
0.75
0.50
0.25
0.00
Gene category
conserved
MASP/mucin
DGF−1
trans−sialidase
RHS

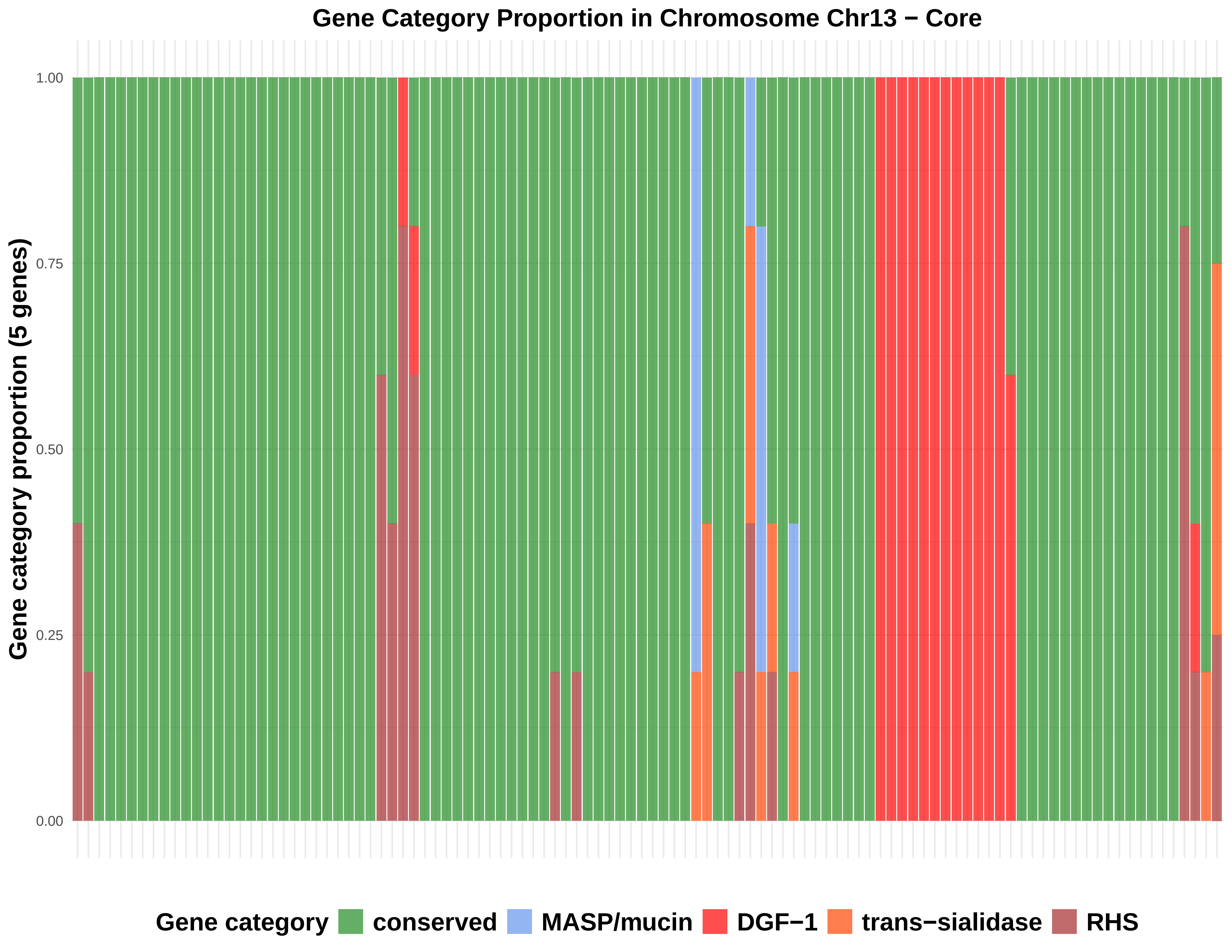
Gene Category Proportion in Chromosome Chr13 − Core
Gene category proportion (5 genes)
1.00
0.75
0.50
0.25
0.00
Gene category
conserved
MASP/mucin
DGF−1
trans−sialidase
RHS

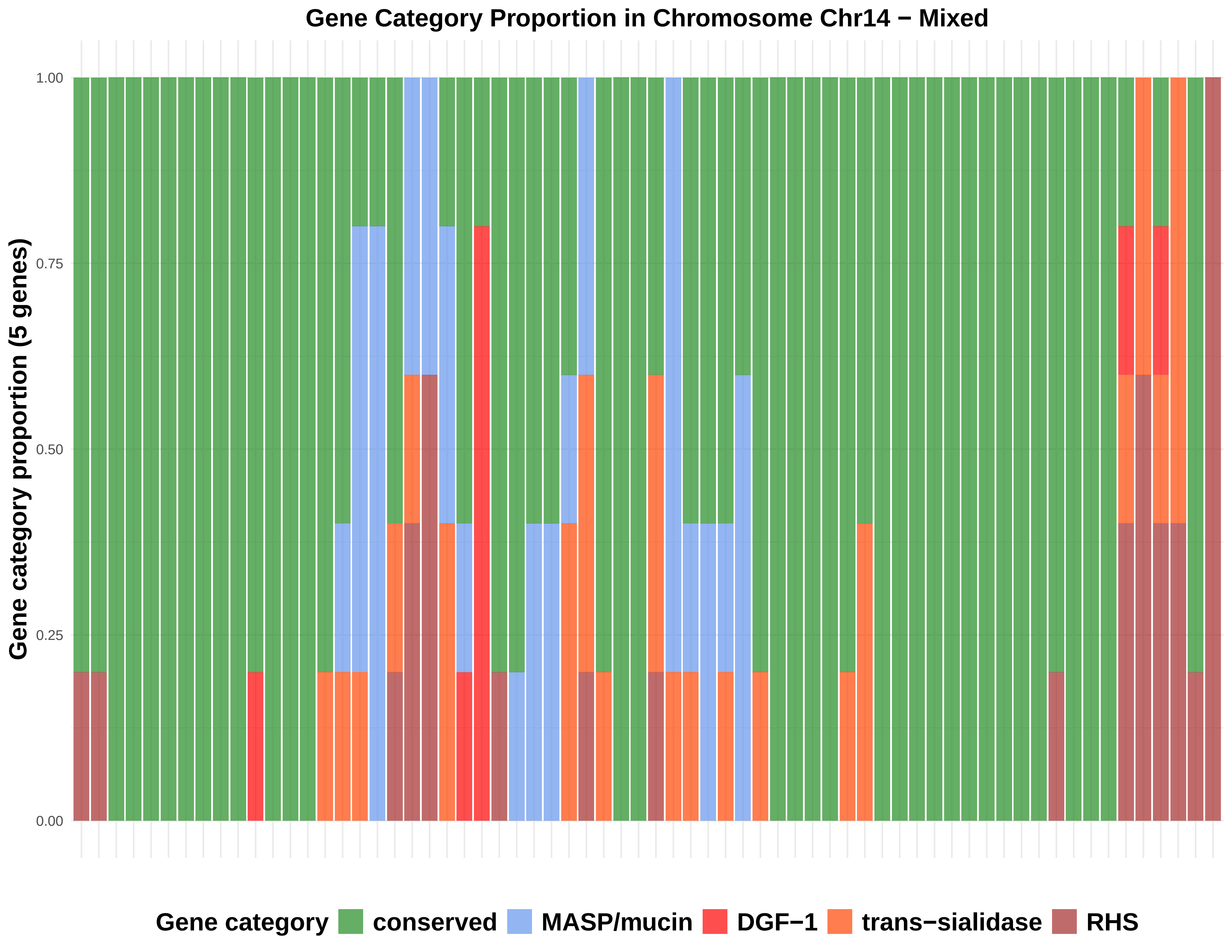
Gene Category Proportion in Chromosome Chr14 − Mixed
Gene category proportion (5 genes)
1.00
0.75
0.50
0.25
0.00
Gene category
conserved
MASP/mucin
DGF−1
trans−sialidase
RHS

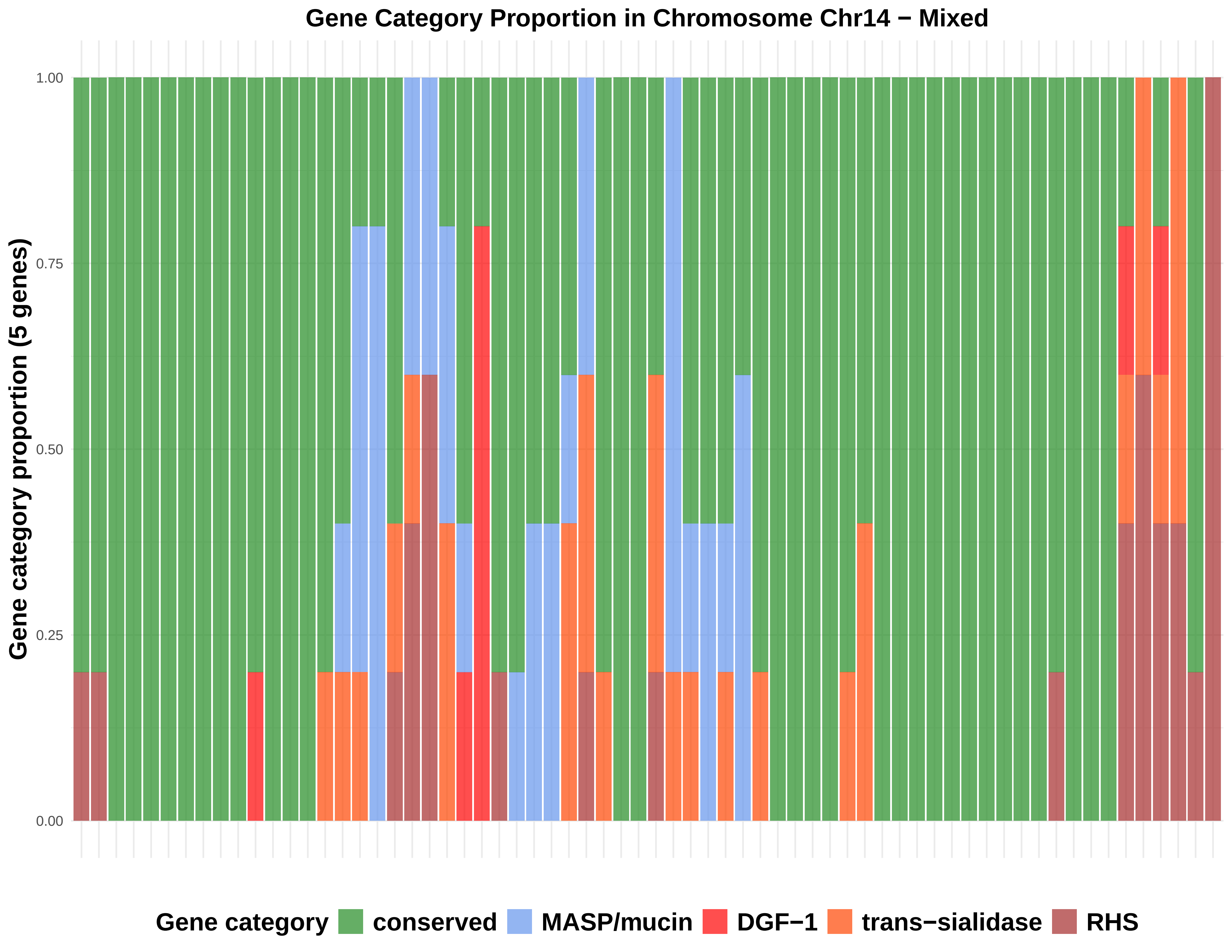
Gene Category Proportion in Chromosome Chr14 − Mixed
Gene category proportion (5 genes)
1.00
0.75
0.50
0.25
0.00
Gene category
conserved
MASP/mucin
DGF−1
trans−sialidase
RHS

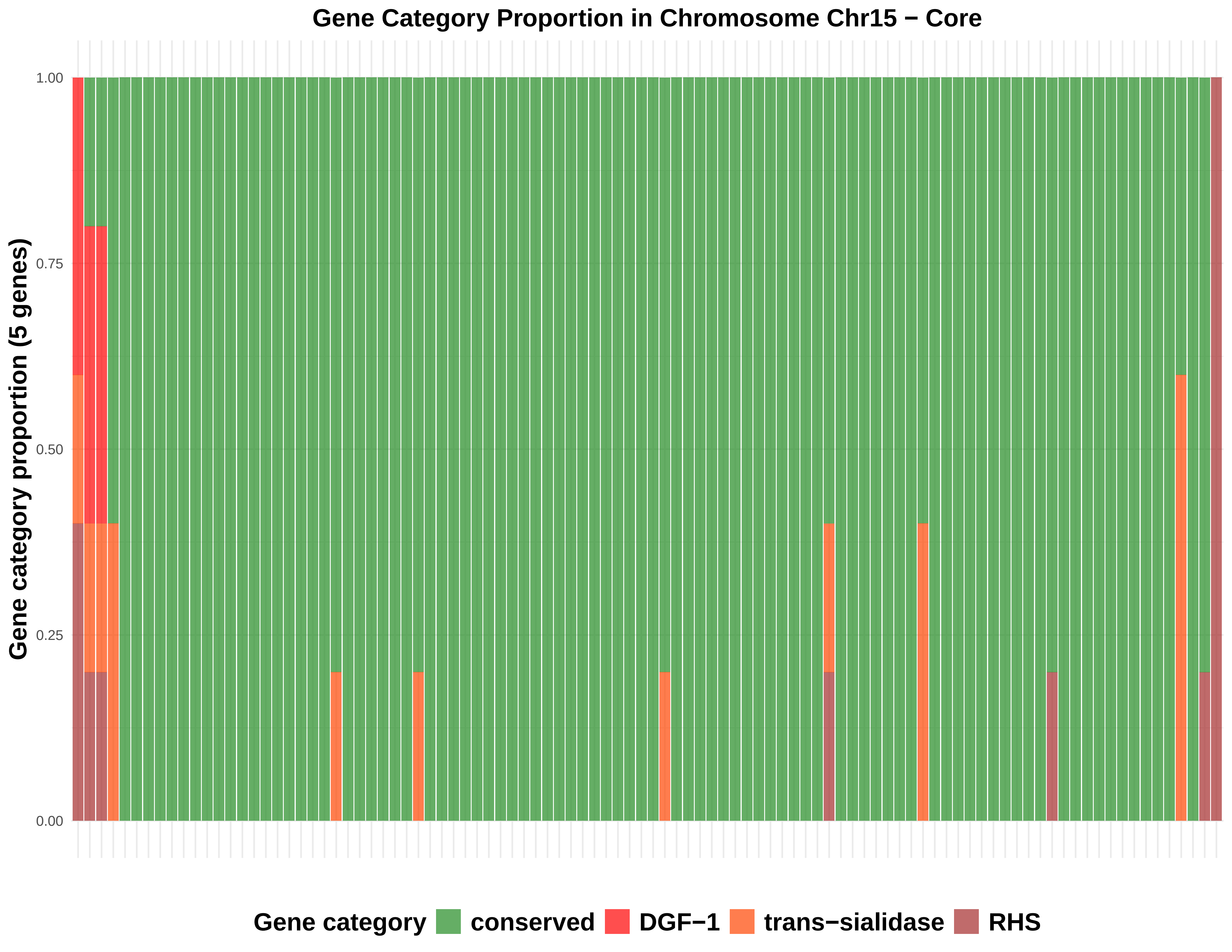

Gene Category Proportion in Chromosome Chr15 − Core
Gene category proportion (5 genes)
1.00
0.75
0.50
0.25
0.00
Gene category
conserved
DGF−1
trans−sialidase
RHS

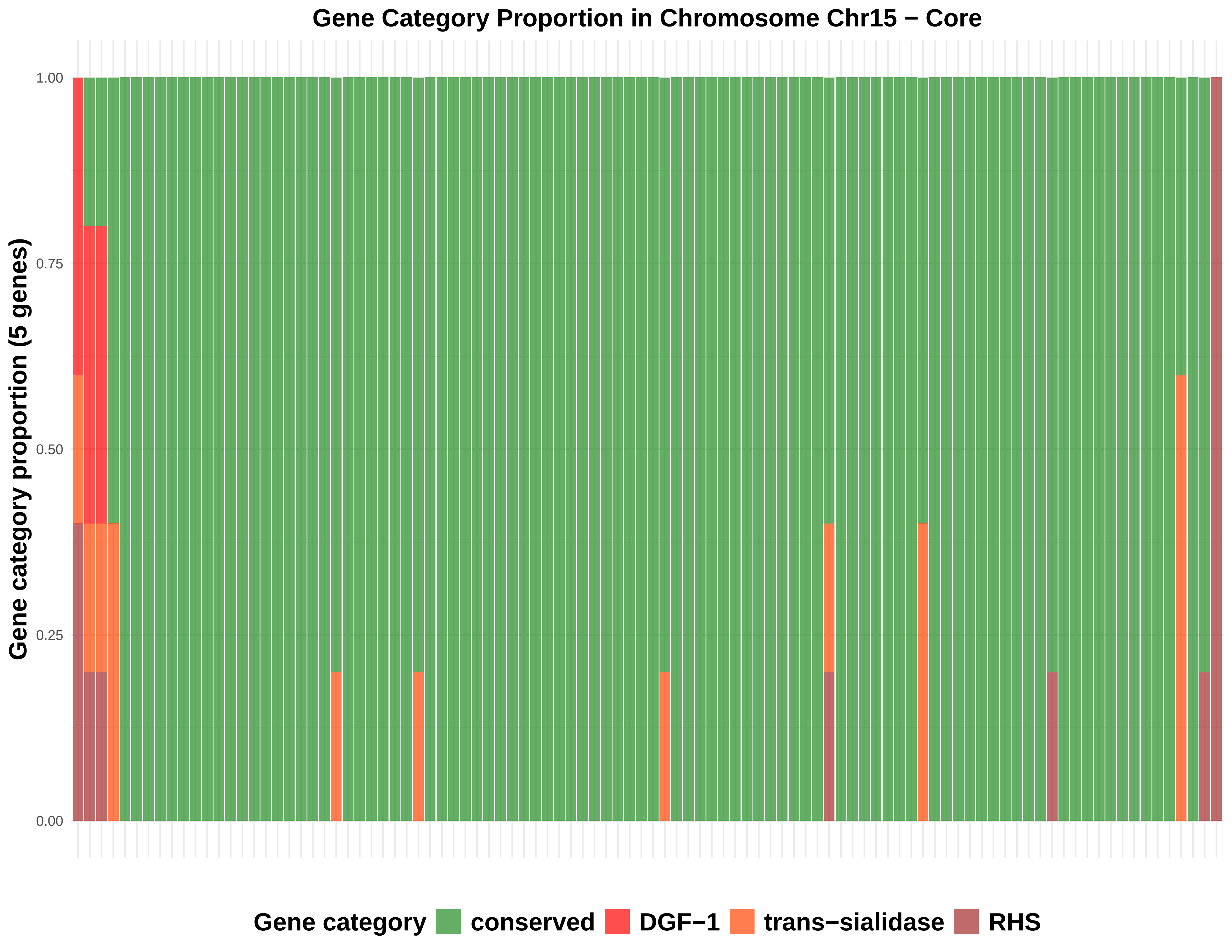

Gene Category Proportion in Chromosome Chr15 − Core
Gene category proportion (5 genes)
1.00
0.75
0.50
0.25
0.00
Gene category
conserved
DGF−1
trans−sialidase
RHS

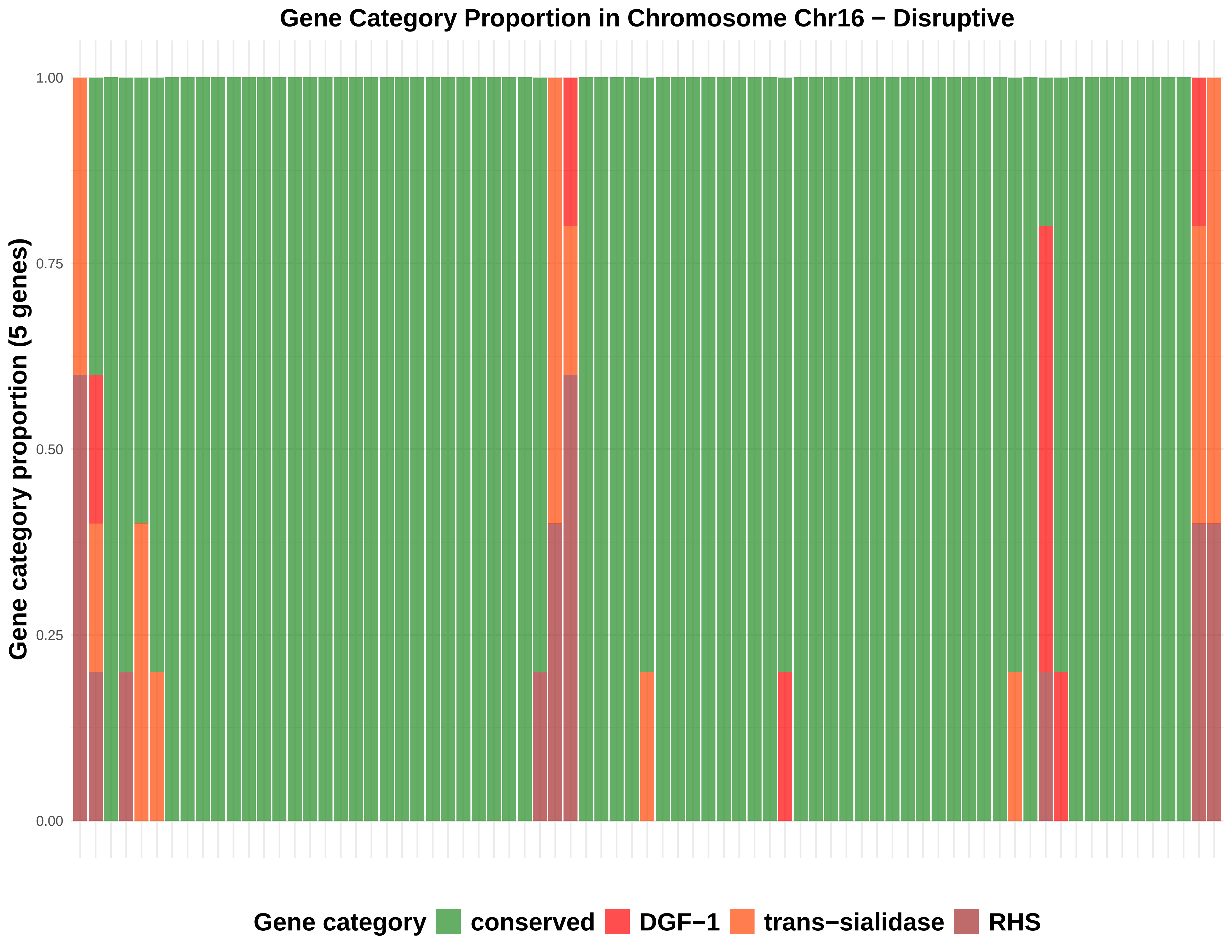
Gene Category Proportion in Chromosome Chr16 − Disruptive
Gene category proportion (5 genes)
1.00
0.75
0.50
0.25
0.00
Gene category
conserved
DGF−1
trans−sialidase
RHS

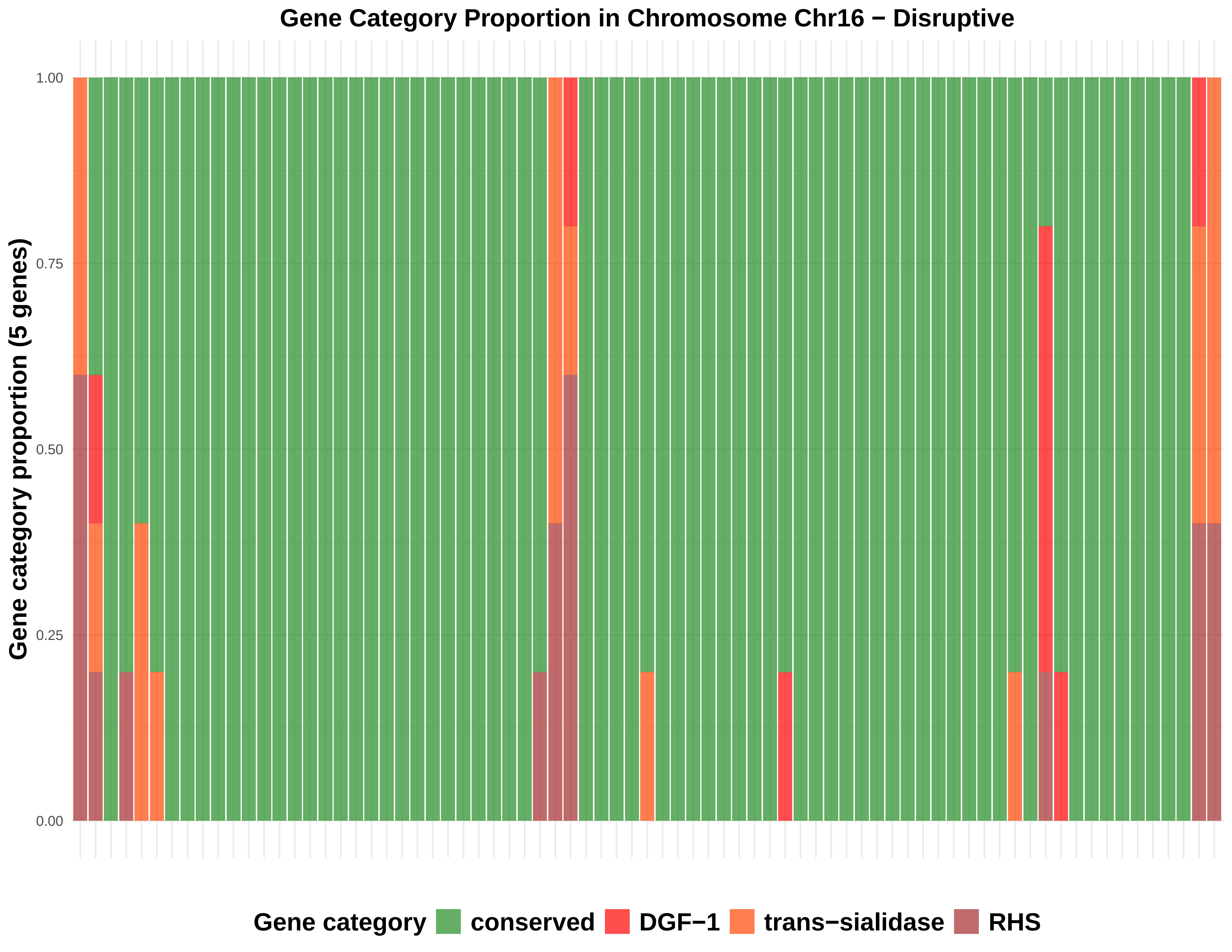

Gene Category Proportion in Chromosome Chr16 − Disruptive
Gene category proportion (5 genes)
1.00
0.75
0.50
0.25
0.00
Gene category
conserved
DGF−1
trans−sialidase
RHS

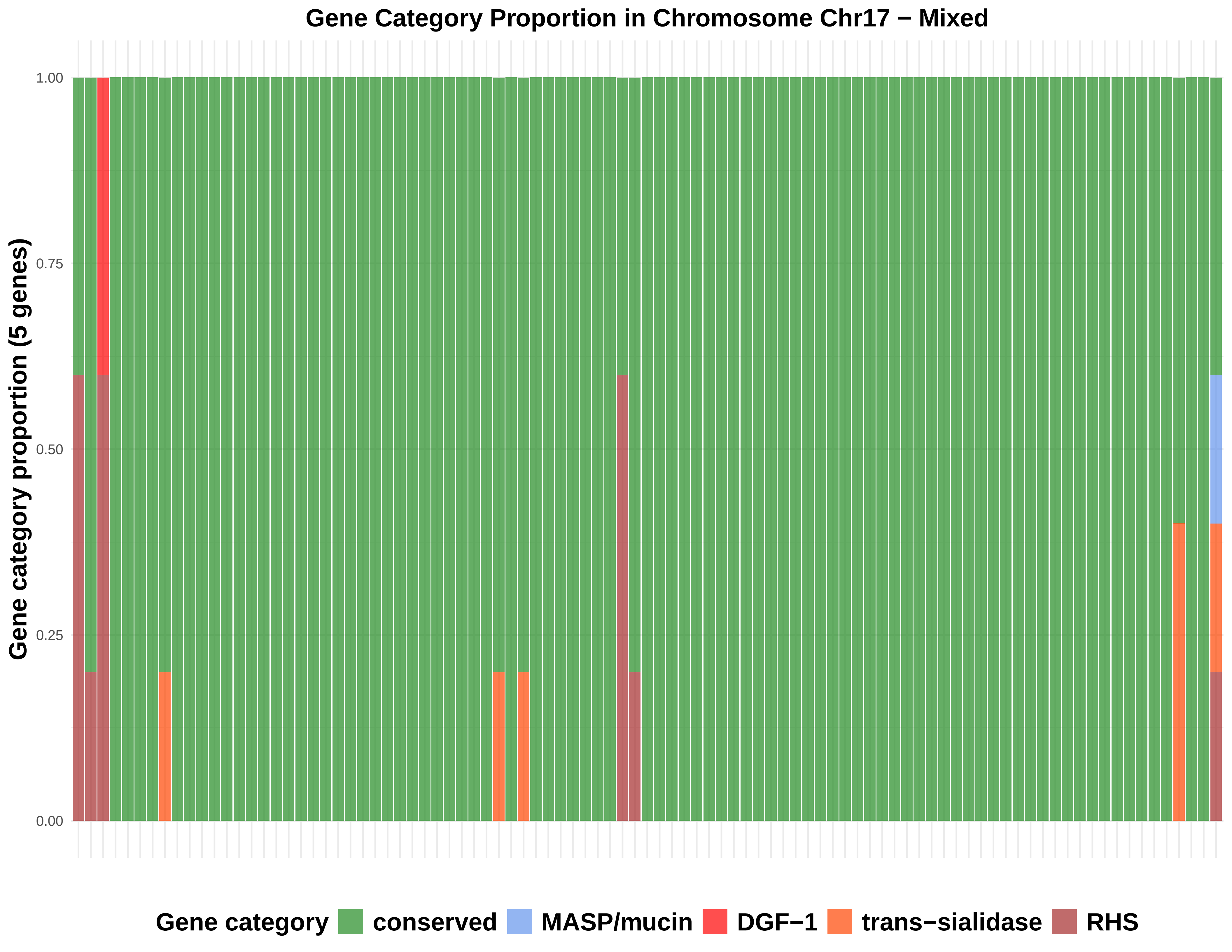
Gene Category Proportion in Chromosome Chr17 − Mixed
Gene category proportion (5 genes)
1.00
0.75
0.50
0.25
0.00
Gene category
conserved
MASP/mucin
DGF−1
trans−sialidase
RHS

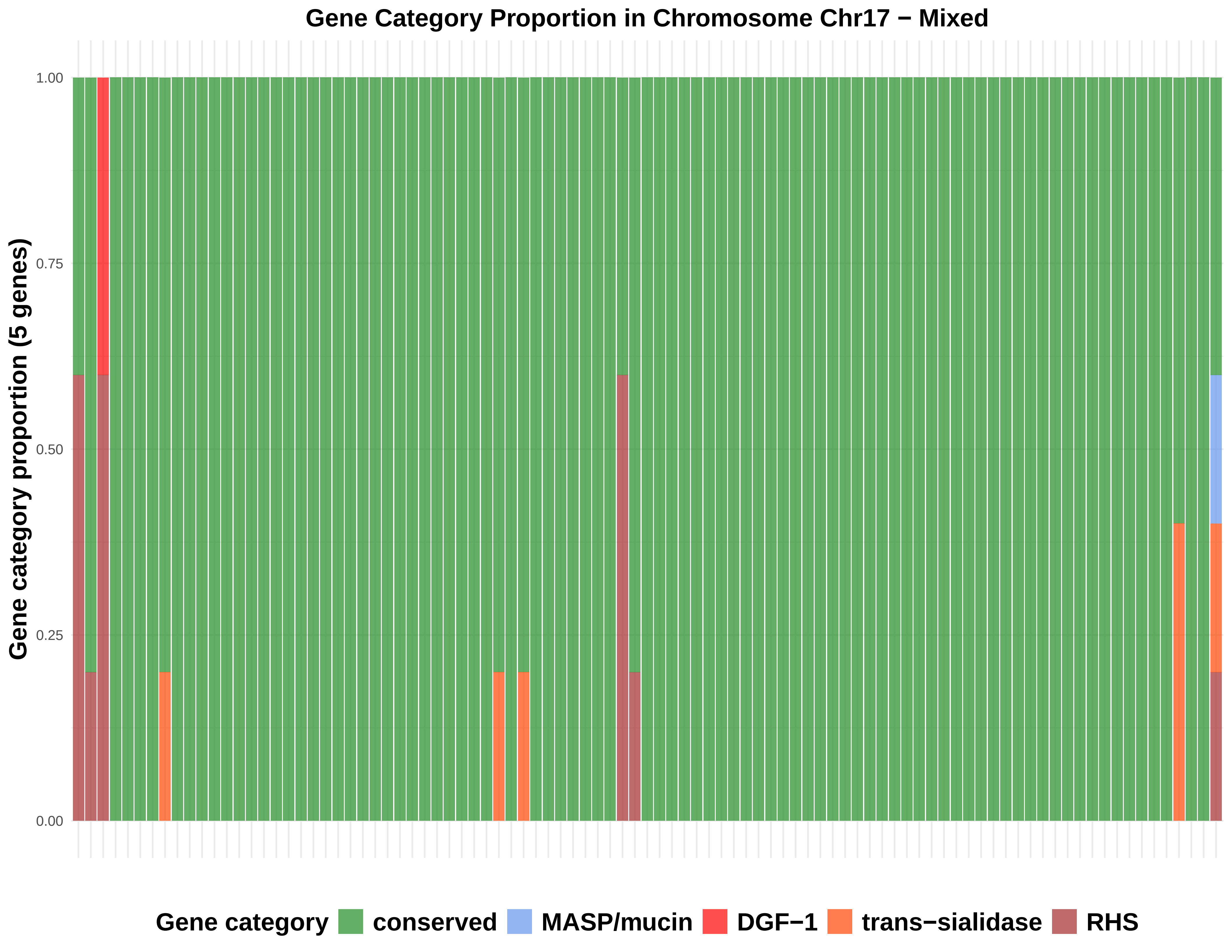
Gene Category Proportion in Chromosome Chr17 − Mixed
Gene category proportion (5 genes)
1.00
0.75
0.50
0.25
0.00
Gene category
conserved
MASP/mucin
DGF−1
trans−sialidase
RHS

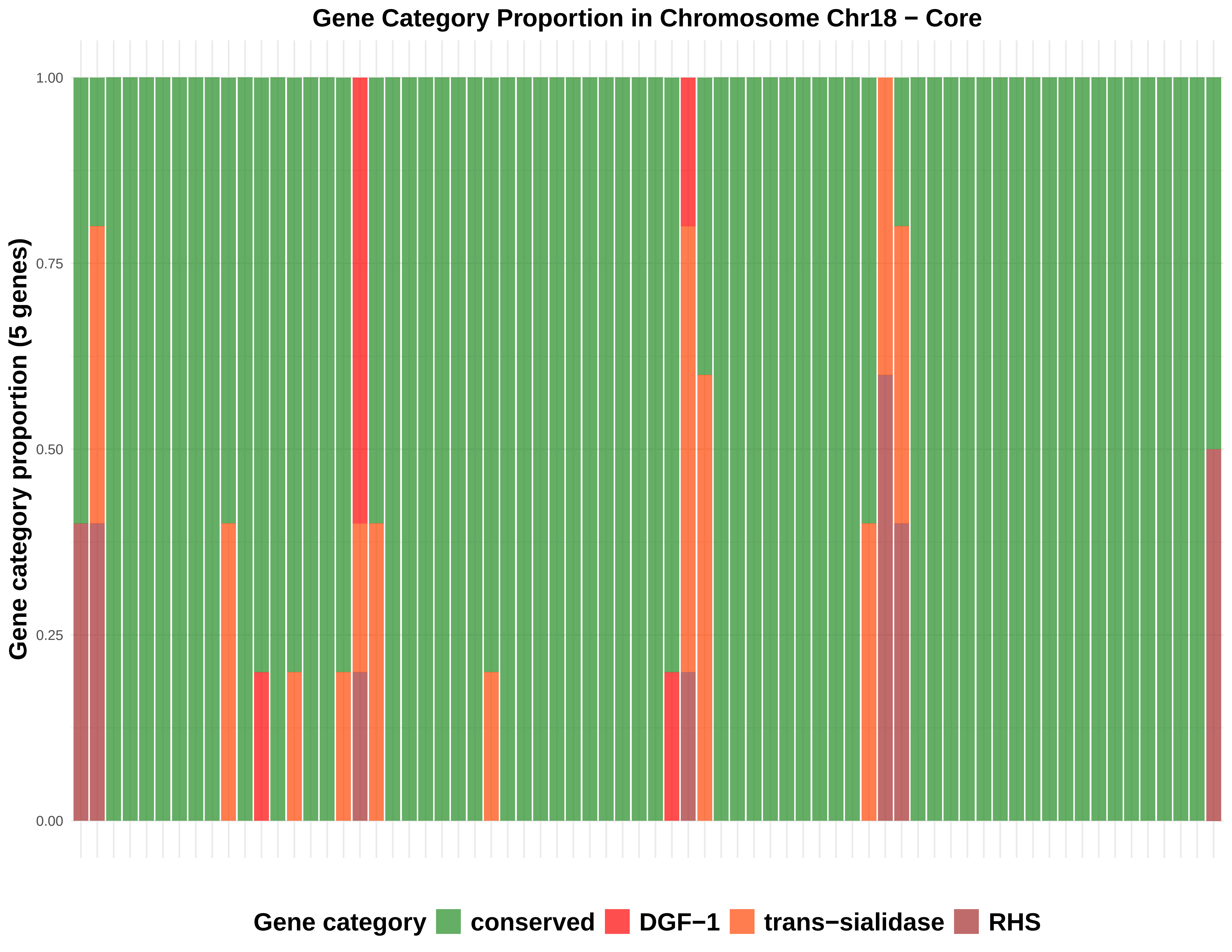

Gene Category Proportion in Chromosome Chr18 − Core
Gene category proportion (5 genes)
1.00
0.75
0.50
0.25
0.00
Gene category
conserved
DGF−1
trans−sialidase
RHS

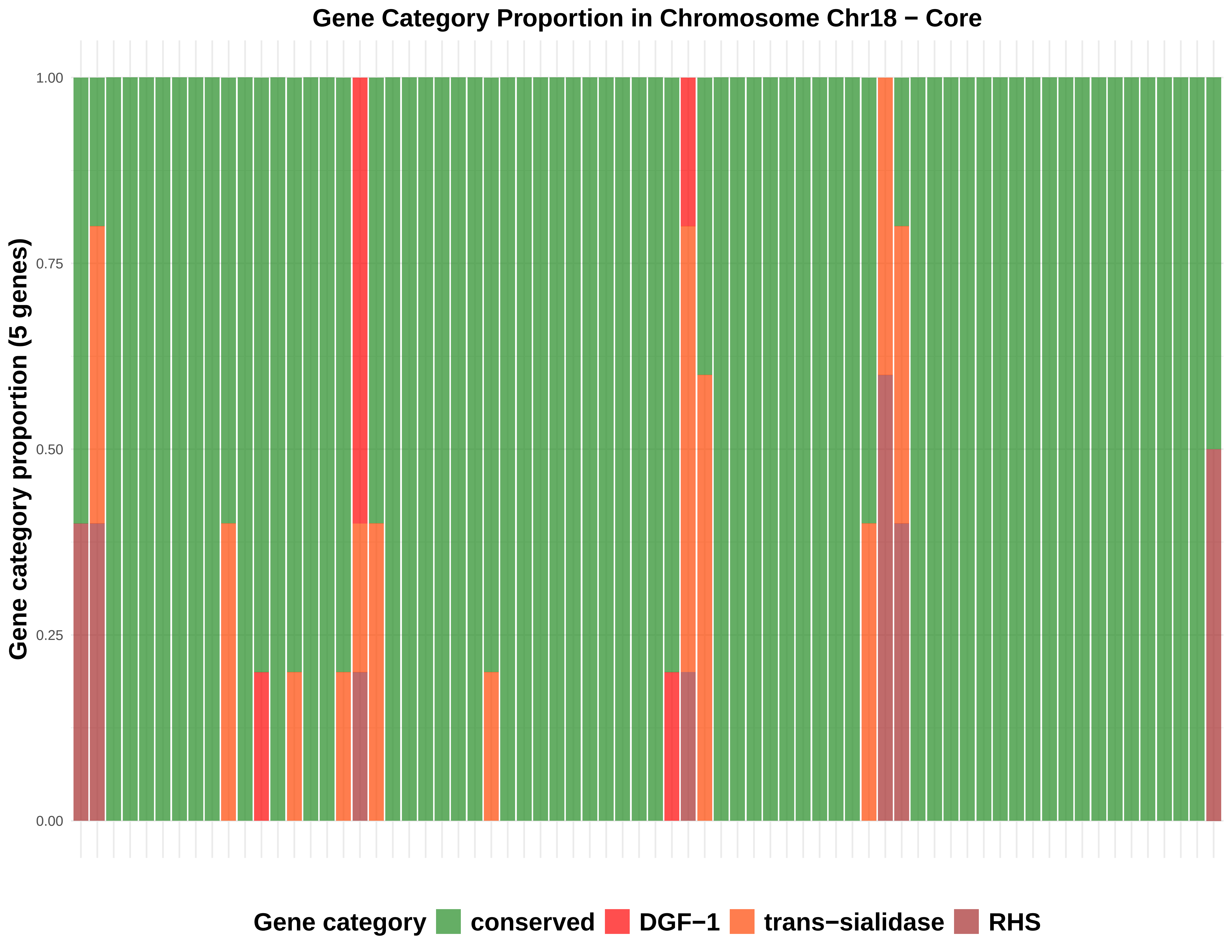
Gene Category Proportion in Chromosome Chr18 − Core
Gene category proportion (5 genes)
1.00
0.75
0.50
0.25
0.00
Gene category
conserved
DGF−1
trans−sialidase
RHS

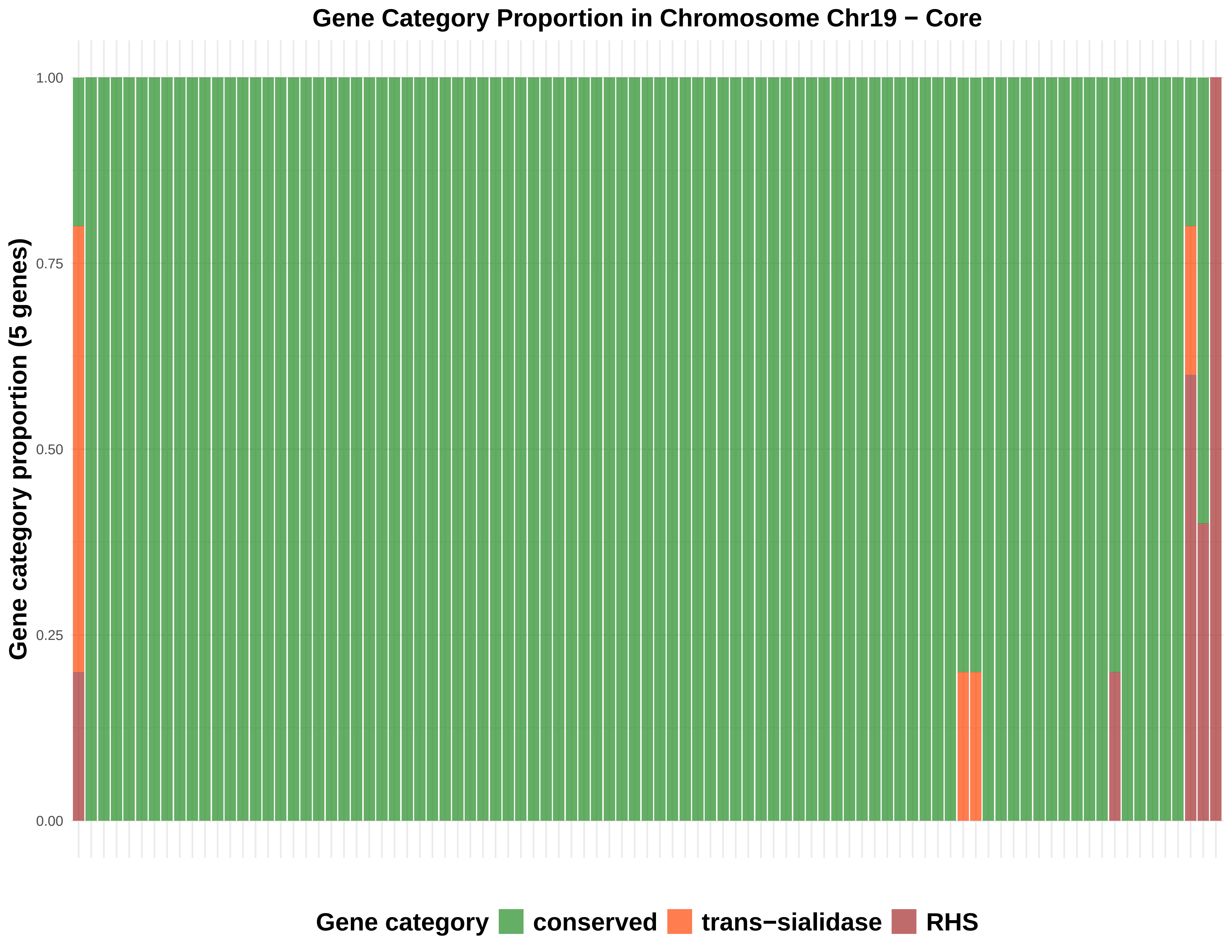
Gene Category Proportion in Chromosome Chr19 − Core
Gene category proportion (5 genes)
1.00
0.75
0.50
0.25
0.00
Gene category
conserved
trans−sialidase
RHS

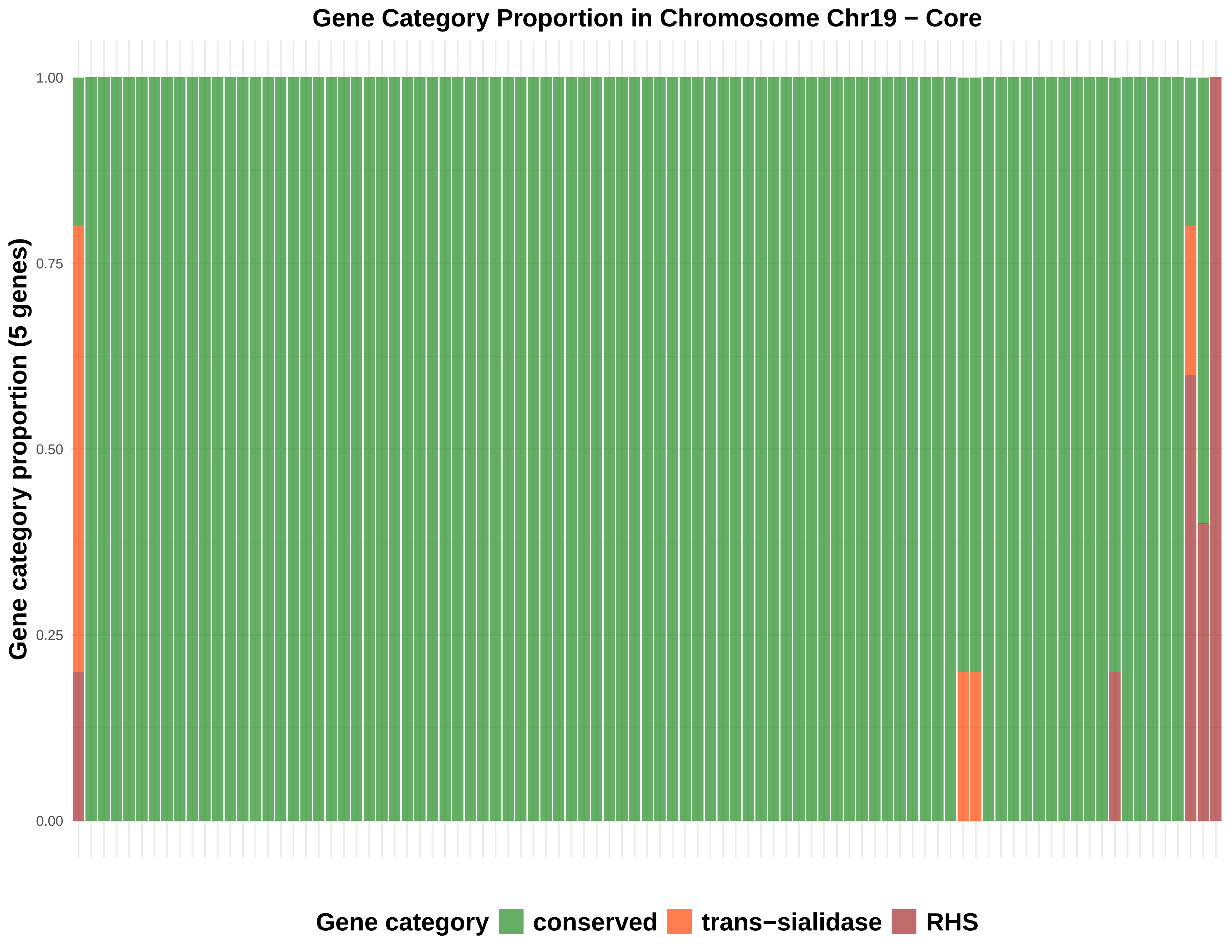

Gene Category Proportion in Chromosome Chr19 − Core
Gene category proportion (5 genes)
1.00
0.75
0.50
0.25
0.00
Gene category
conserved
trans−sialidase
RHS

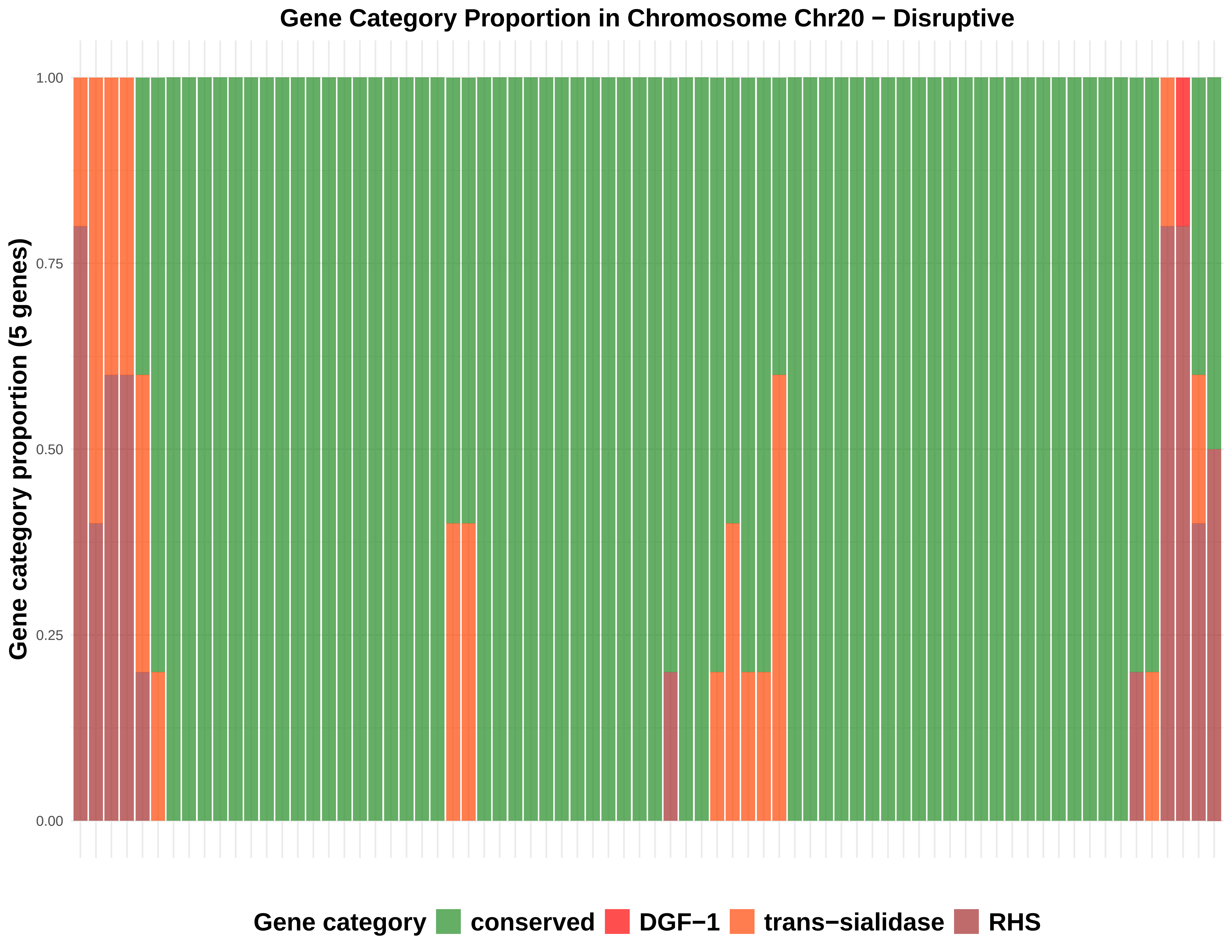

Gene Category Proportion in Chromosome Chr20 − Disruptive
Gene category proportion (5 genes)
1.00
0.75
0.50
0.25
0.00
Gene category
conserved
DGF−1
trans−sialidase
RHS

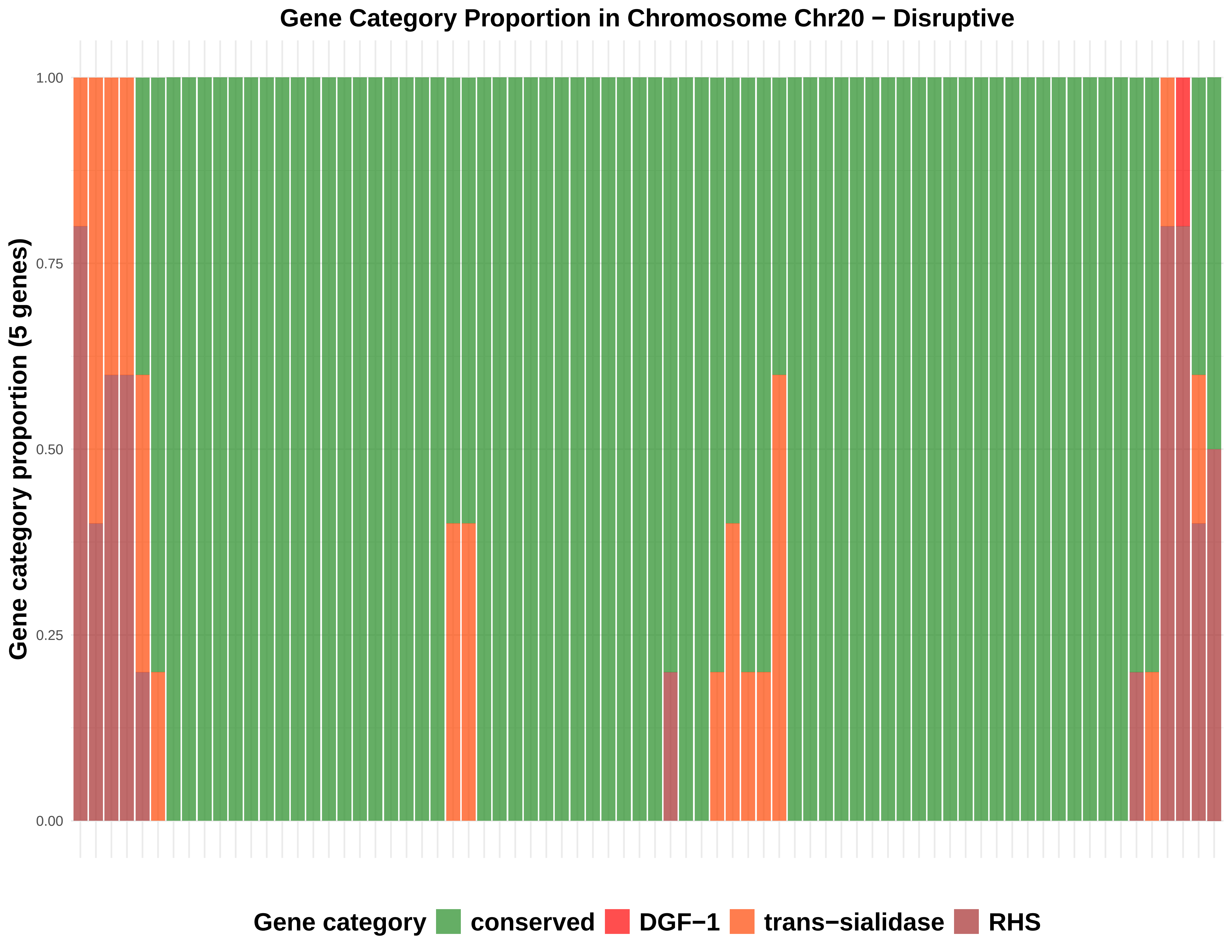
Gene Category Proportion in Chromosome Chr20 − Disruptive
Gene category proportion (5 genes)
1.00
0.75
0.50
0.25
0.00
Gene category
conserved
DGF−1
trans−sialidase
RHS

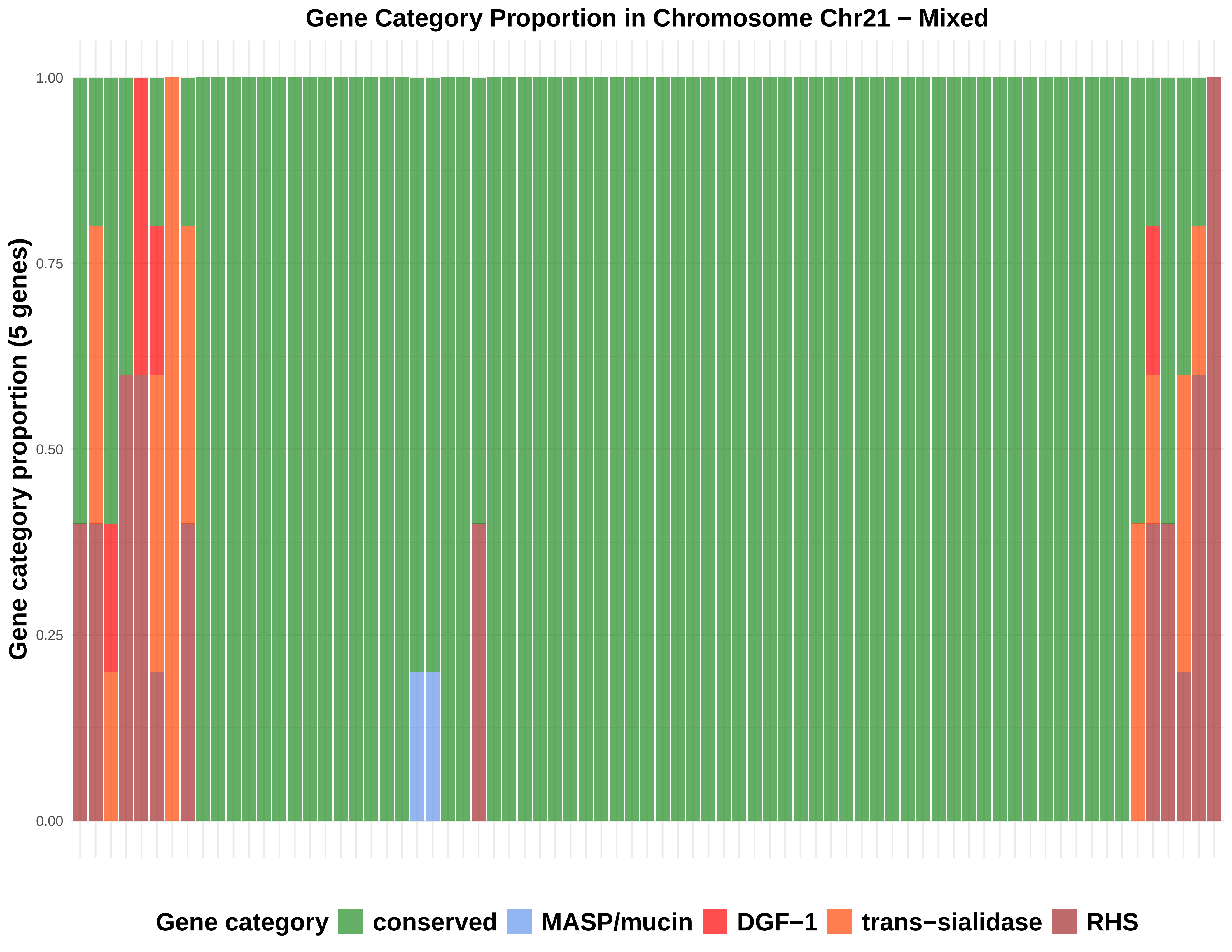

Gene Category Proportion in Chromosome Chr21 − Mixed
Gene category proportion (5 genes)
1.00
0.75
0.50
0.25
0.00
Gene category
conserved
MASP/mucin
DGF−1
trans−sialidase
RHS

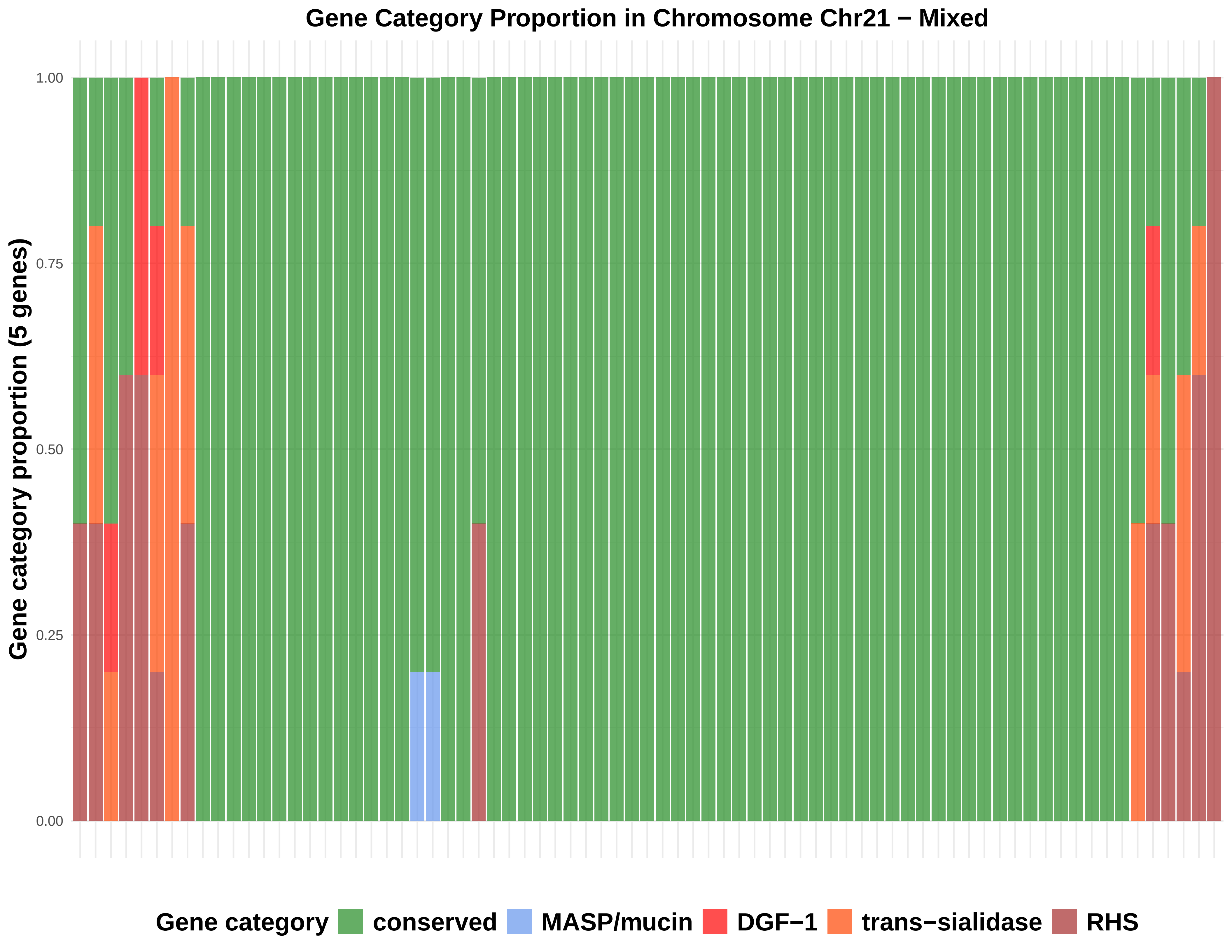

Gene Category Proportion in Chromosome Chr21 − Mixed
Gene category proportion (5 genes)
1.00
0.75
0.50
0.25
0.00
Gene category
conserved
MASP/mucin
DGF−1
trans−sialidase
RHS

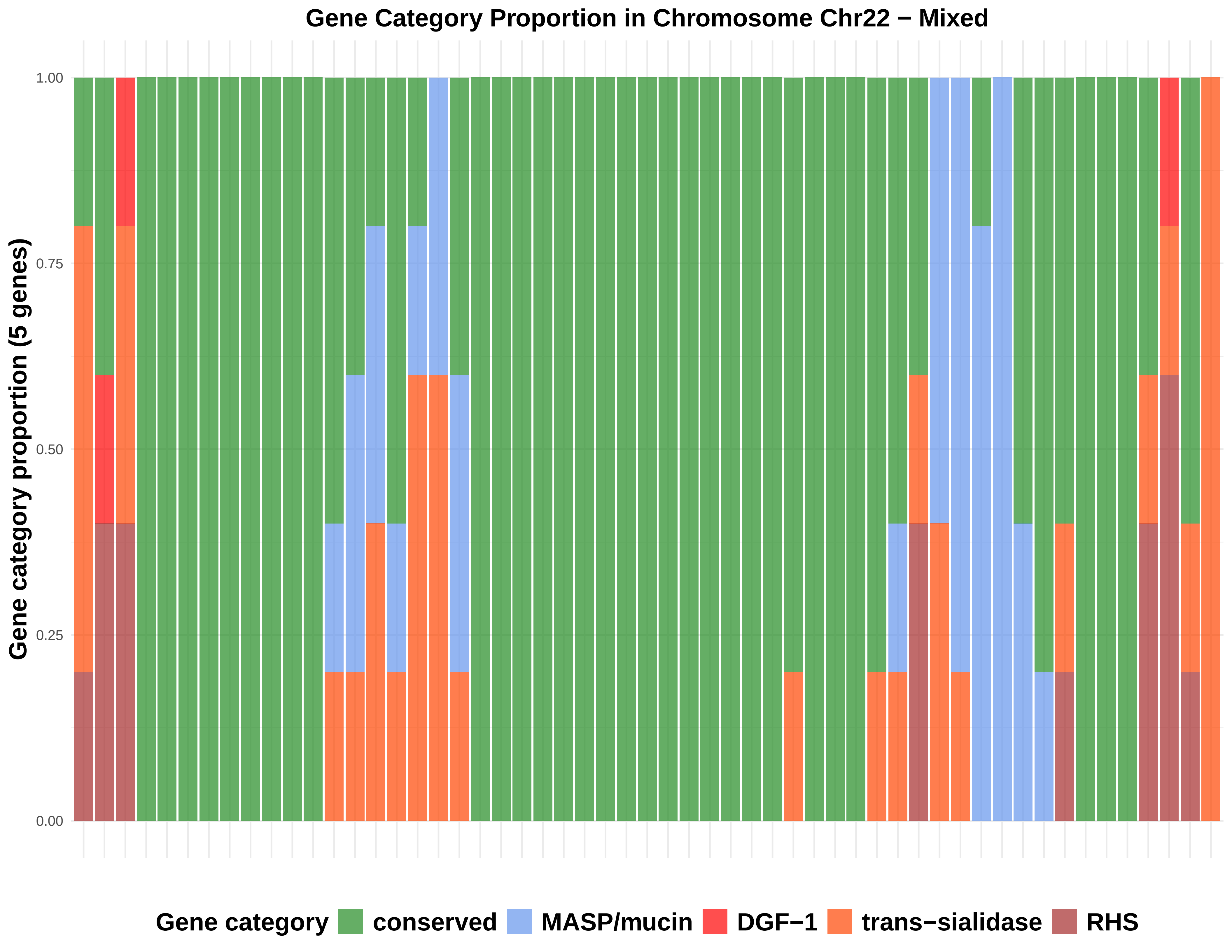
Gene Category Proportion in Chromosome Chr22 − Mixed
Gene category proportion (5 genes)
1.00
0.75
0.50
0.25
0.00
Gene category
conserved
MASP/mucin
DGF−1
trans−sialidase
RHS

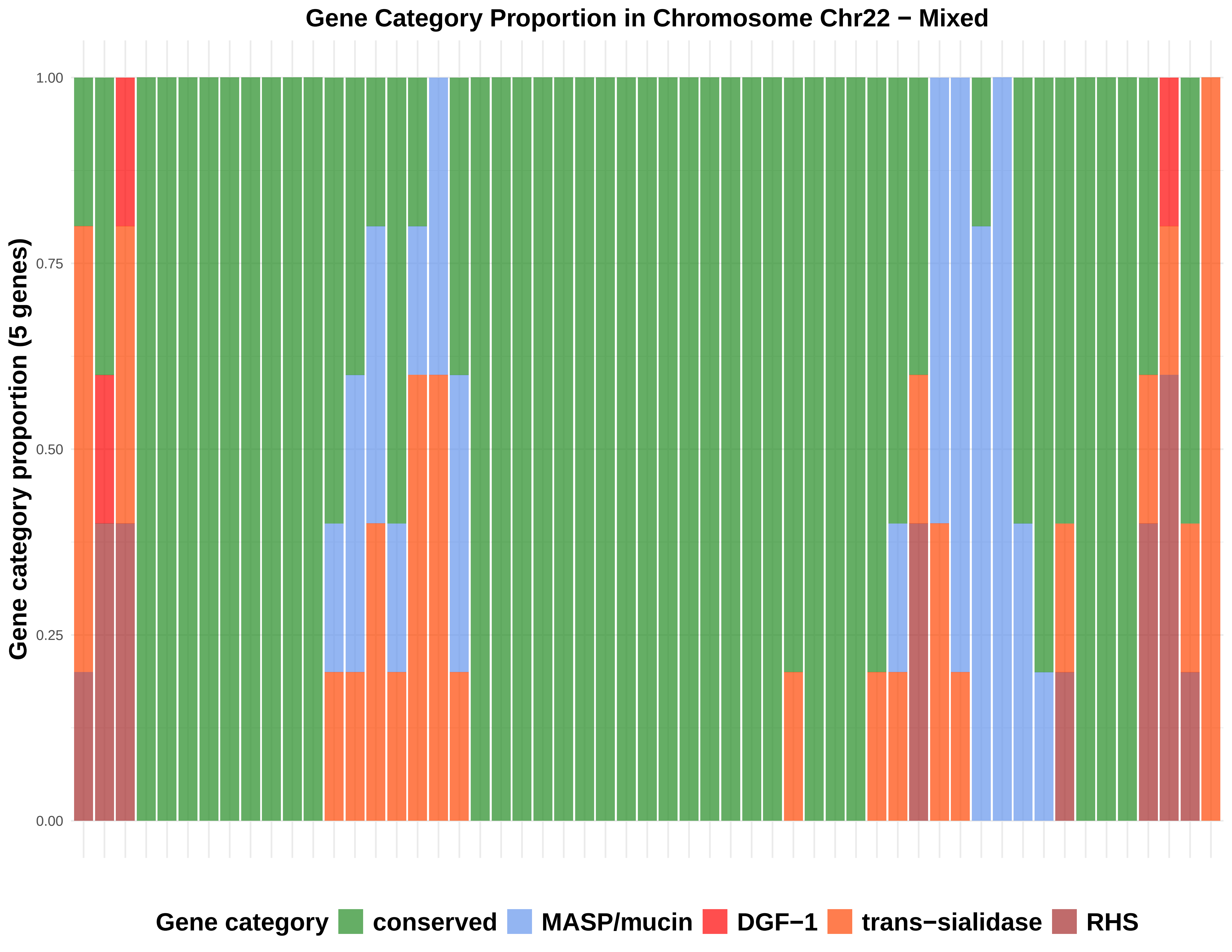

Gene Category Proportion in Chromosome Chr22 − Mixed
Gene category proportion (5 genes)
1.00
0.75
0.50
0.25
0.00
Gene category
conserved
MASP/mucin
DGF−1
trans−sialidase
RHS

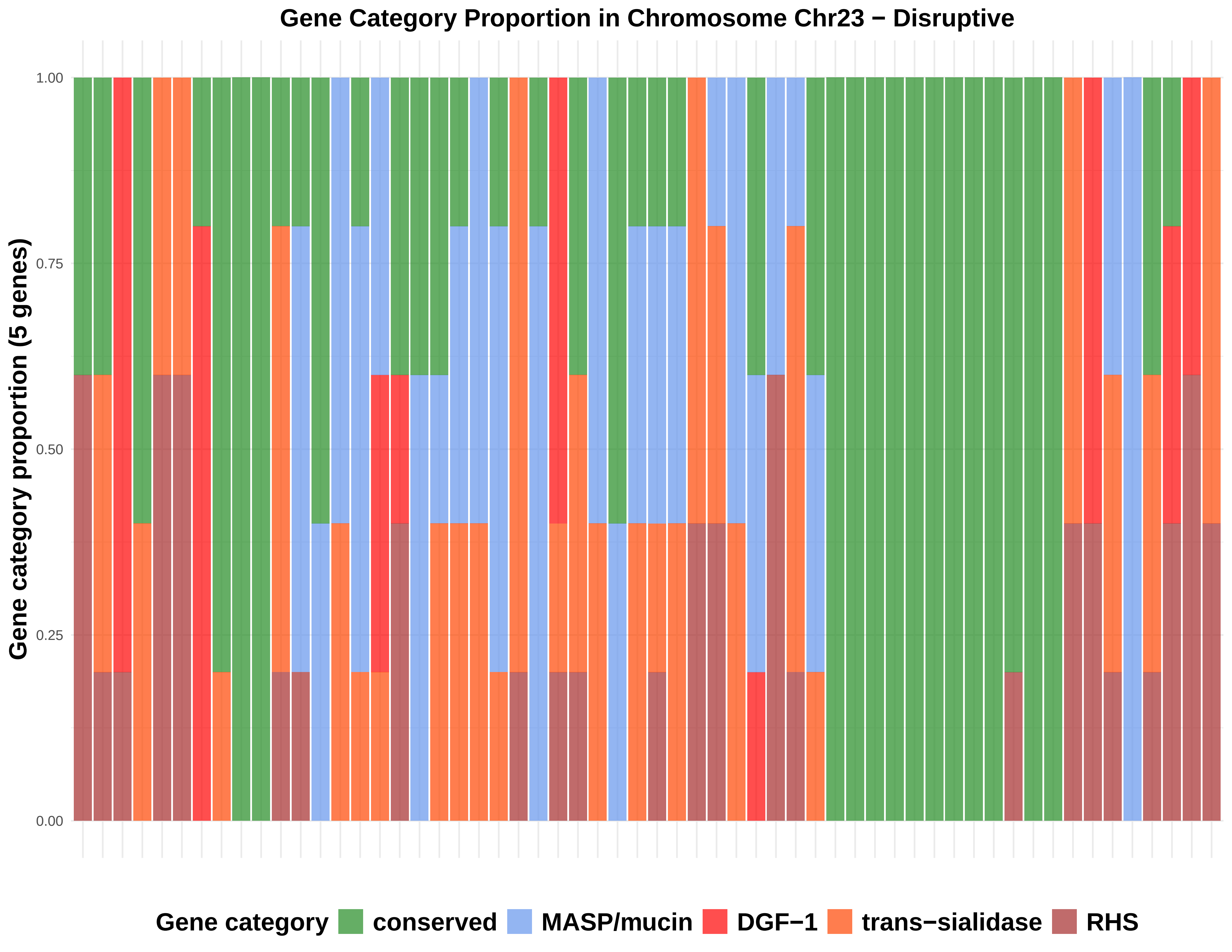
Gene Category Proportion in Chromosome Chr23 − Disruptive
Gene category proportion (5 genes)
1.00
0.75
0.50
0.25
0.00
Gene category
conserved
MASP/mucin
DGF−1
trans−sialidase
RHS

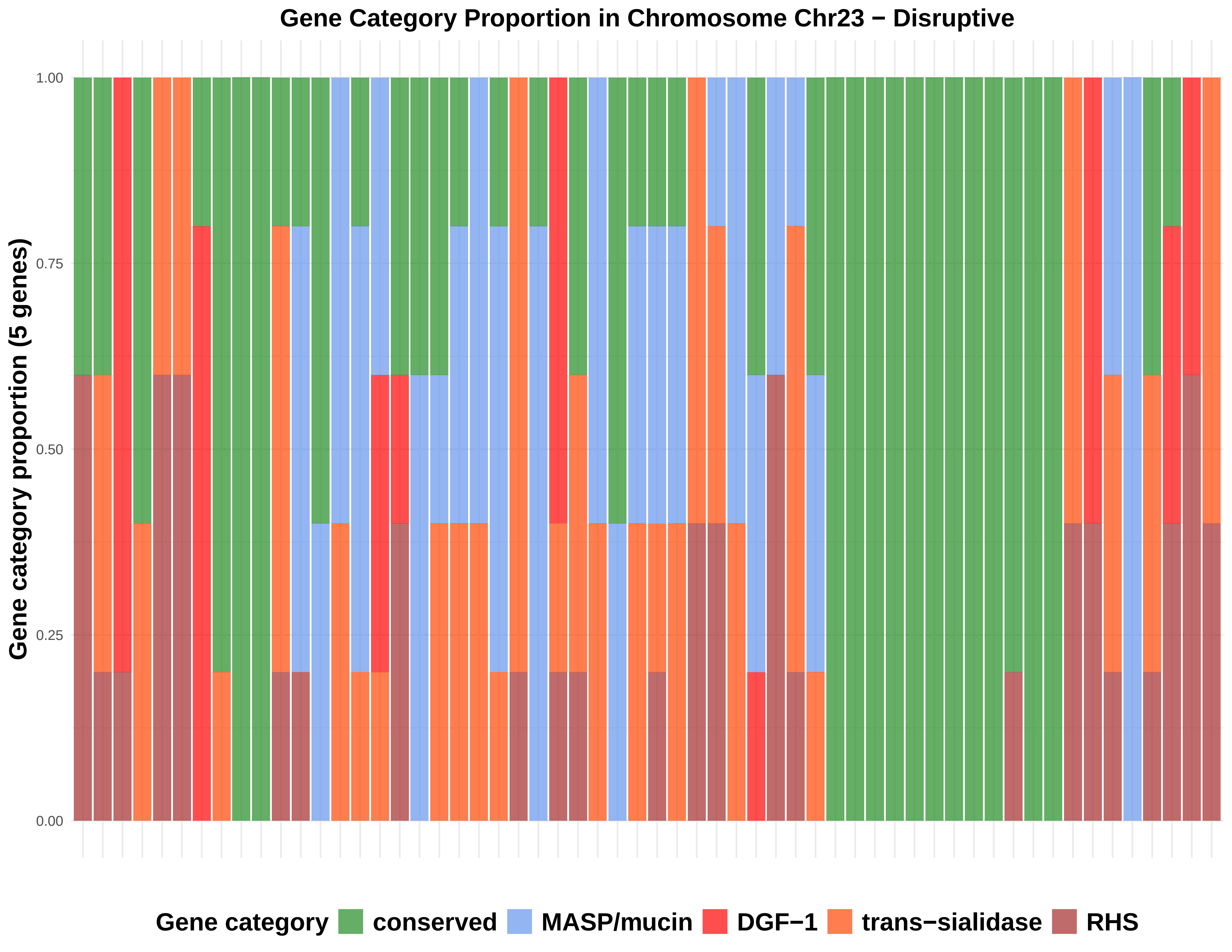

Gene Category Proportion in Chromosome Chr23 − Disruptive
Gene category proportion (5 genes)
1.00
0.75
0.50
0.25
0.00
Gene category
conserved
MASP/mucin
DGF−1
trans−sialidase
RHS

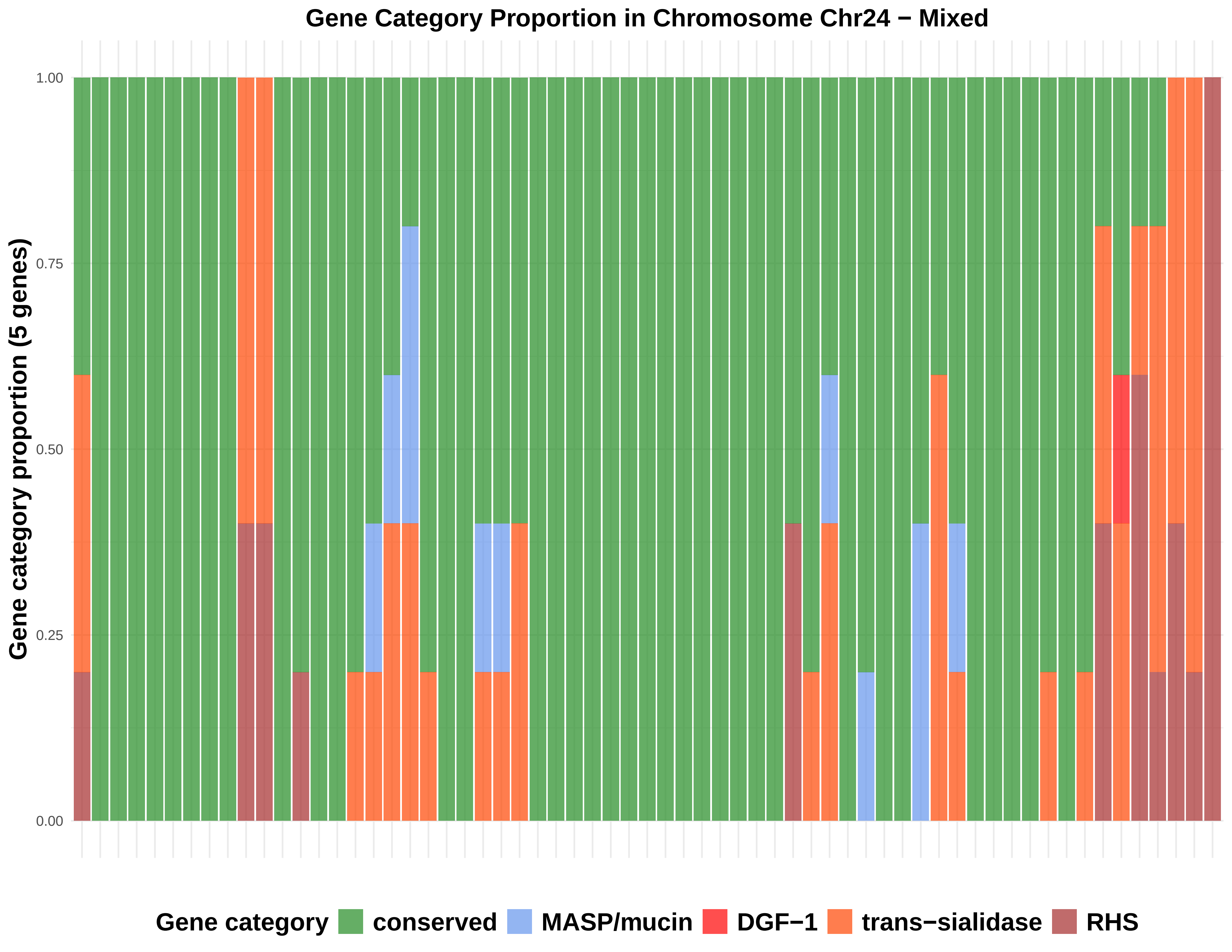

Gene Category Proportion in Chromosome Chr24 − Mixed
Gene category proportion (5 genes)
1.00
0.75
0.50
0.25
0.00
Gene category
conserved
MASP/mucin
DGF−1
trans−sialidase
RHS

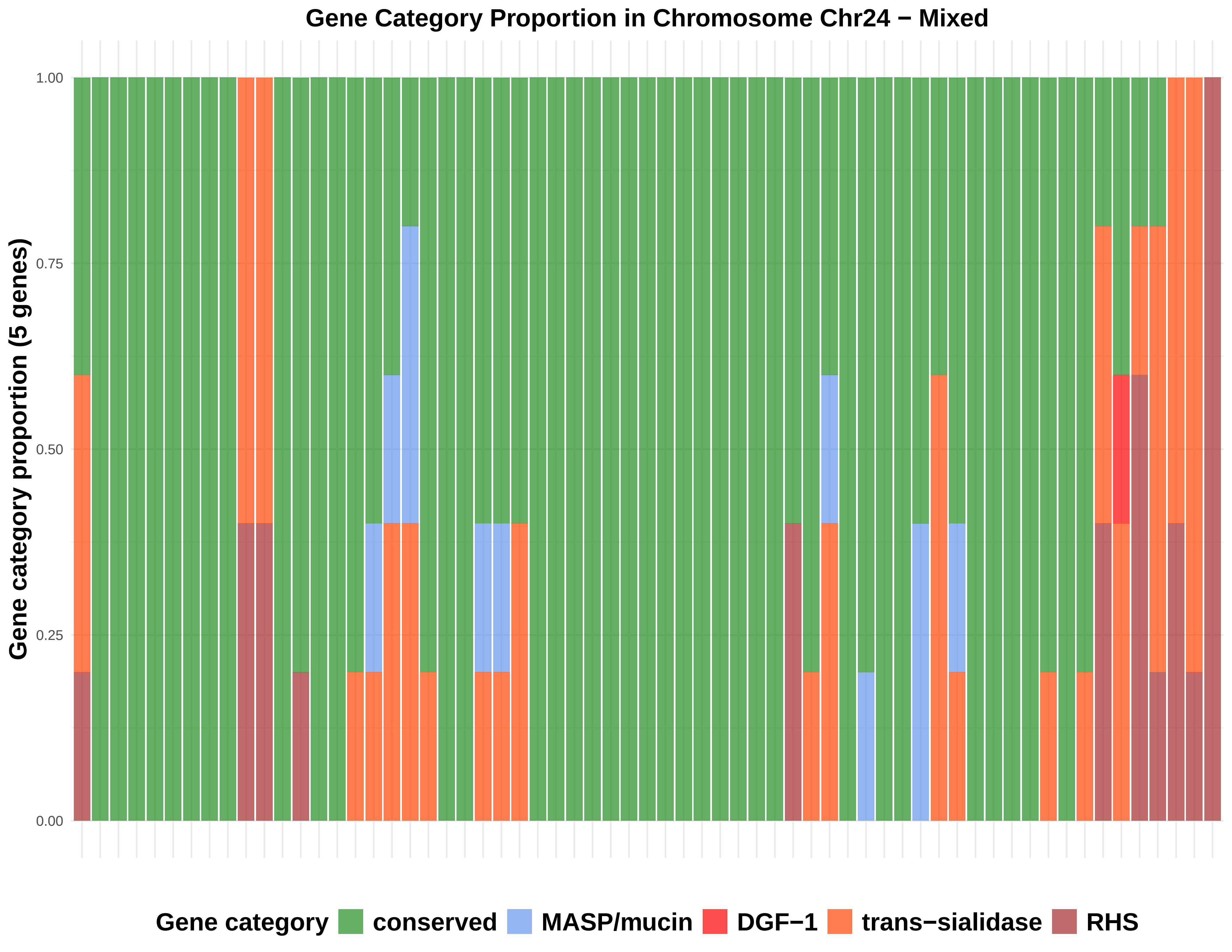

Gene Category Proportion in Chromosome Chr24 − Mixed
Gene category proportion (5 genes)
1.00
0.75
0.50
0.25
0.00
Gene category
conserved
MASP/mucin
DGF−1
trans−sialidase
RHS

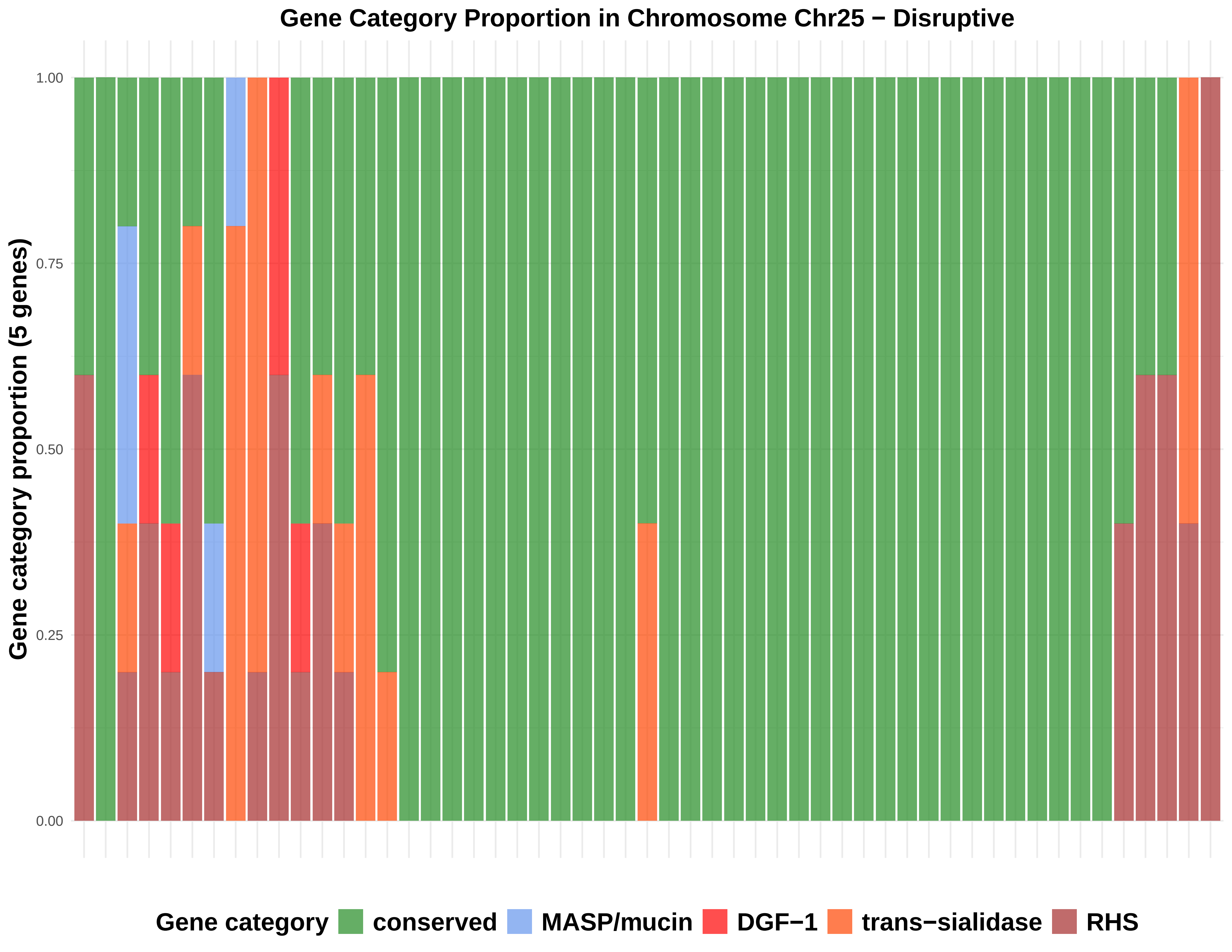

Gene Category Proportion in Chromosome Chr25 − Disruptive
Gene category proportion (5 genes)
1.00
0.75
0.50
0.25
0.00
Gene category
conserved
MASP/mucin
DGF−1
trans−sialidase
RHS

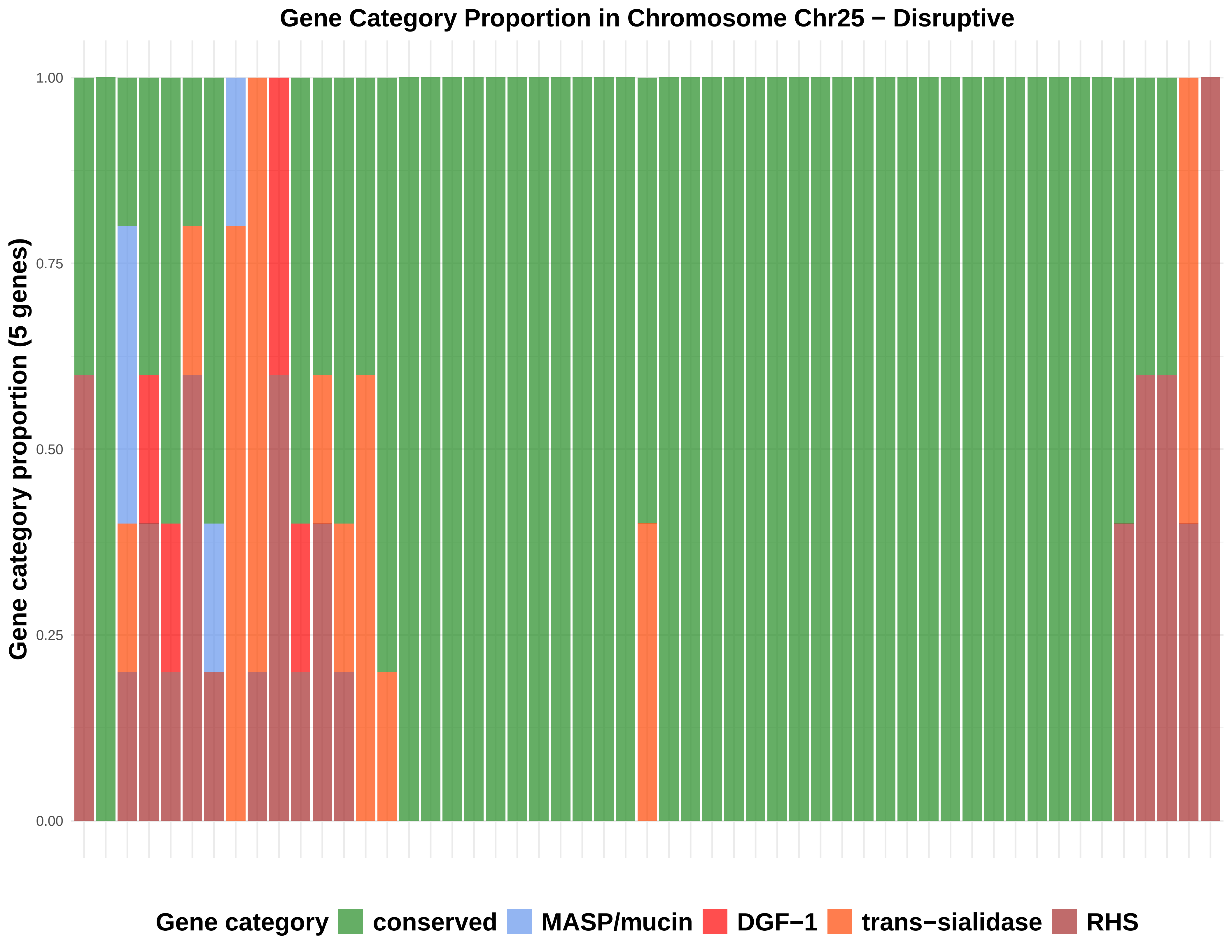
Gene Category Proportion in Chromosome Chr25 − Disruptive
Gene category proportion (5 genes)
1.00
0.75
0.50
0.25
0.00
Gene category
conserved
MASP/mucin
DGF−1
trans−sialidase
RHS

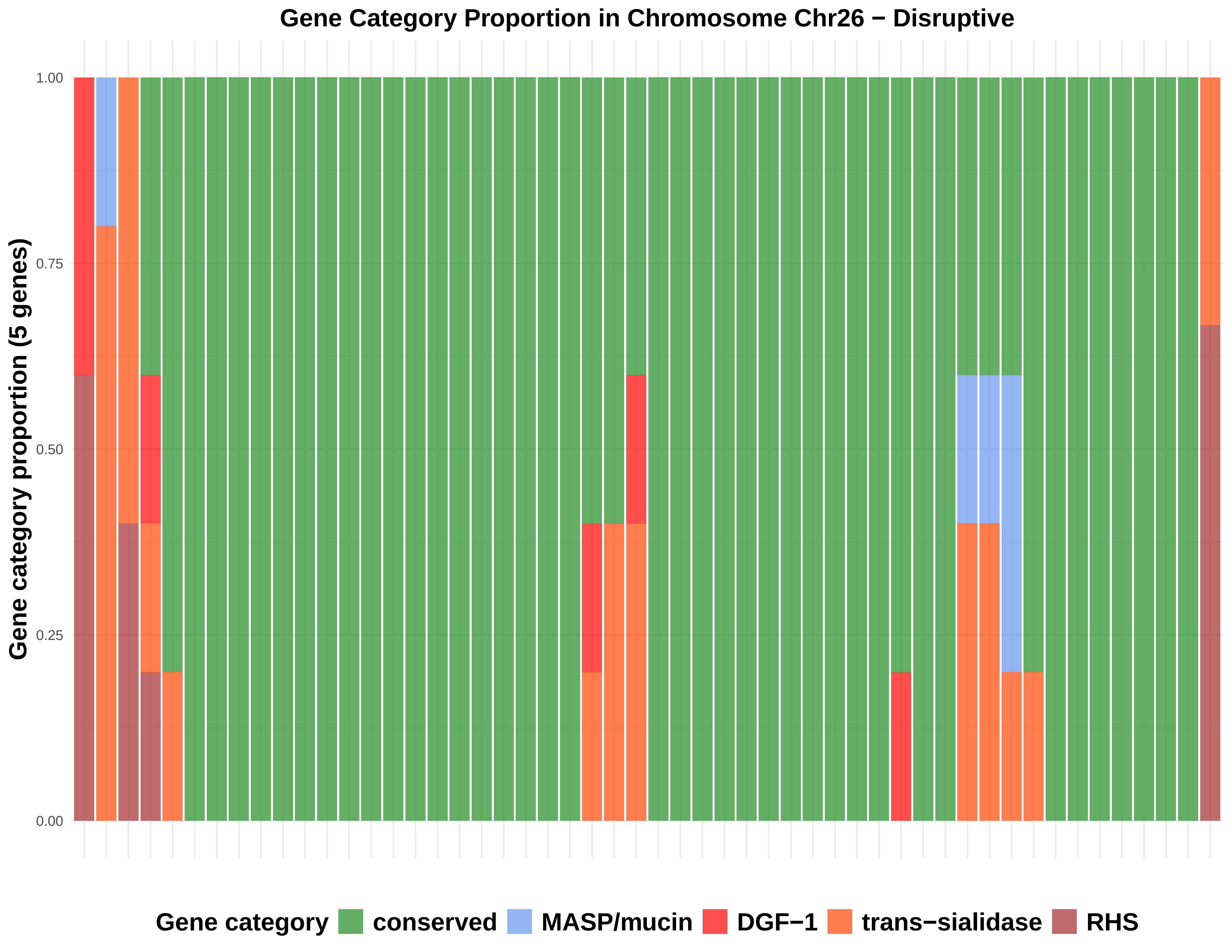

Gene Category Proportion in Chromosome Chr26 − Disruptive
Gene category proportion (5 genes)
1.00
0.75
0.50
0.25
0.00
Gene category
conserved
MASP/mucin
DGF−1
trans−sialidase
RHS

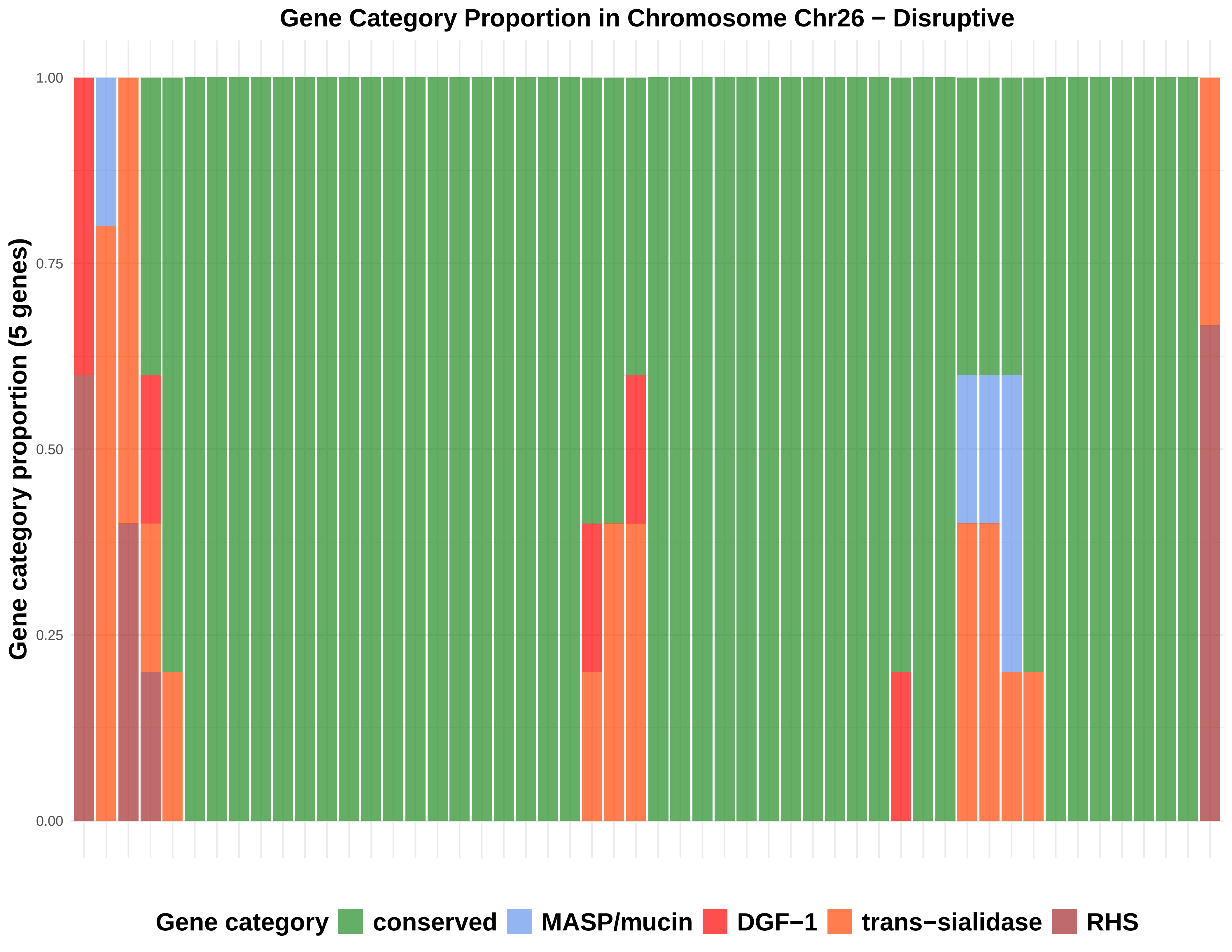

Gene Category Proportion in Chromosome Chr26 − Disruptive
Gene category proportion (5 genes)
1.00
0.75
0.50
0.25
0.00
Gene category
conserved
MASP/mucin
DGF−1
trans−sialidase
RHS

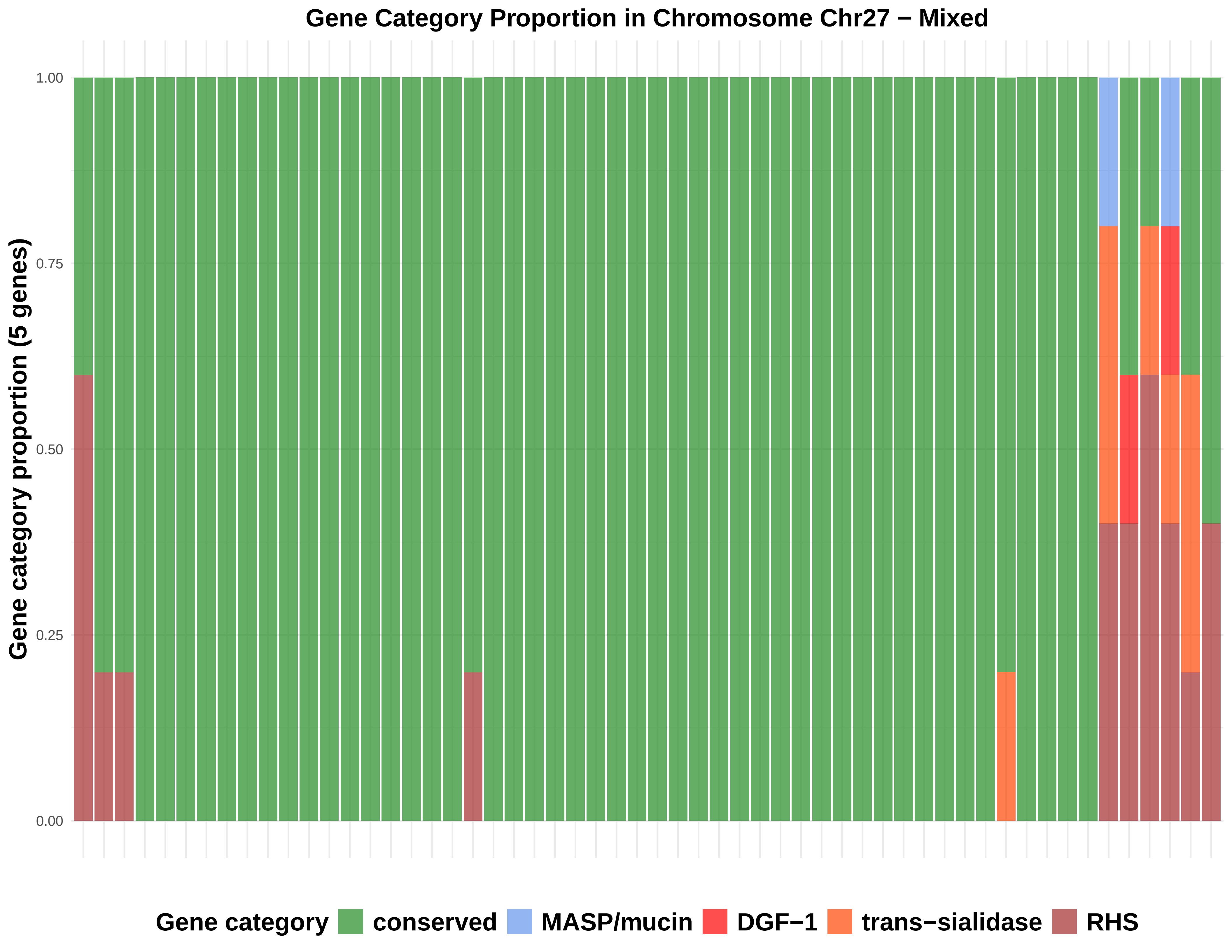

Gene Category Proportion in Chromosome Chr27 − Mixed
Gene category proportion (5 genes)
1.00
0.75
0.50
0.25
0.00
Gene category
conserved
MASP/mucin
DGF−1
trans−sialidase
RHS

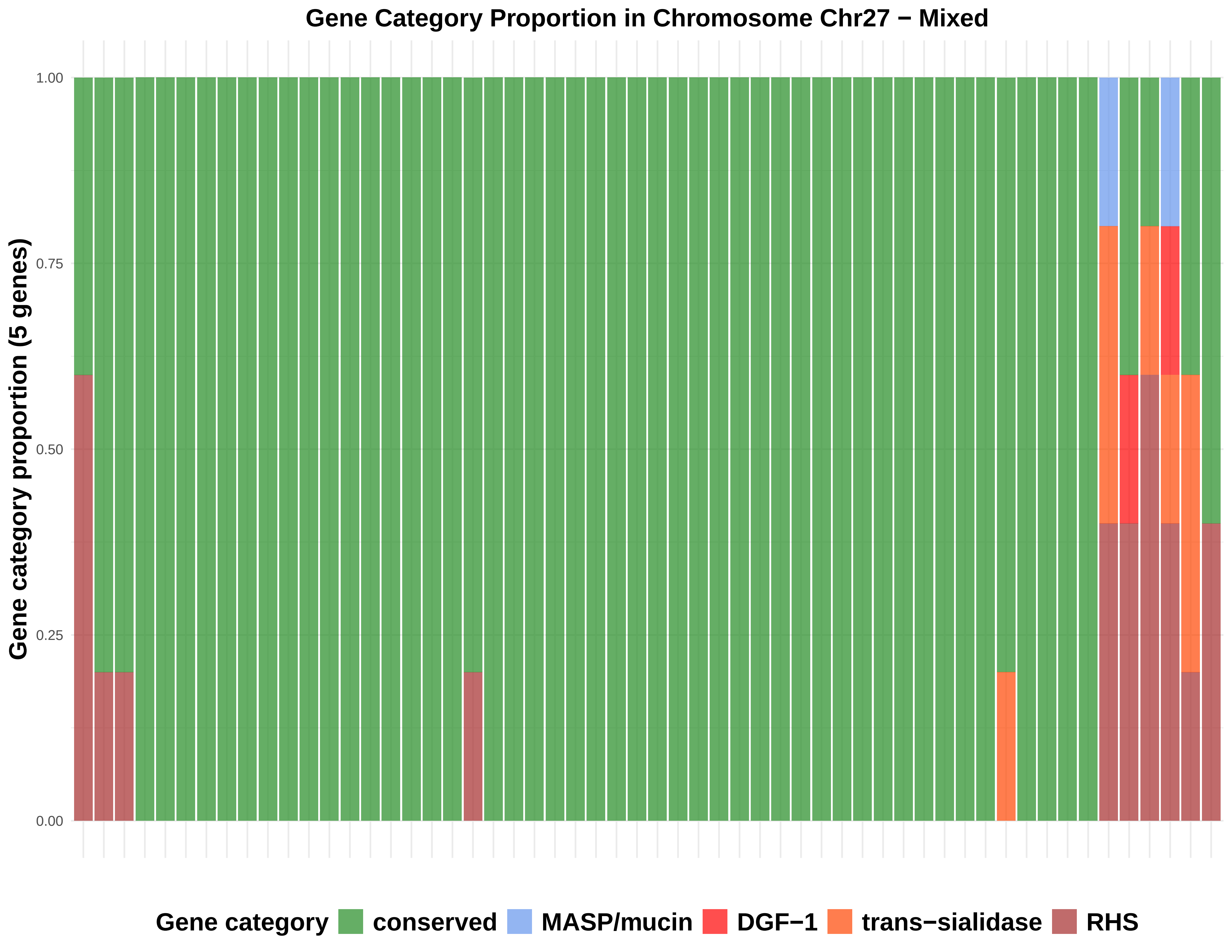
Gene Category Proportion in Chromosome Chr27 − Mixed
Gene category proportion (5 genes)
1.00
0.75
0.50
0.25
0.00
Gene category
conserved
MASP/mucin
DGF−1
trans−sialidase
RHS

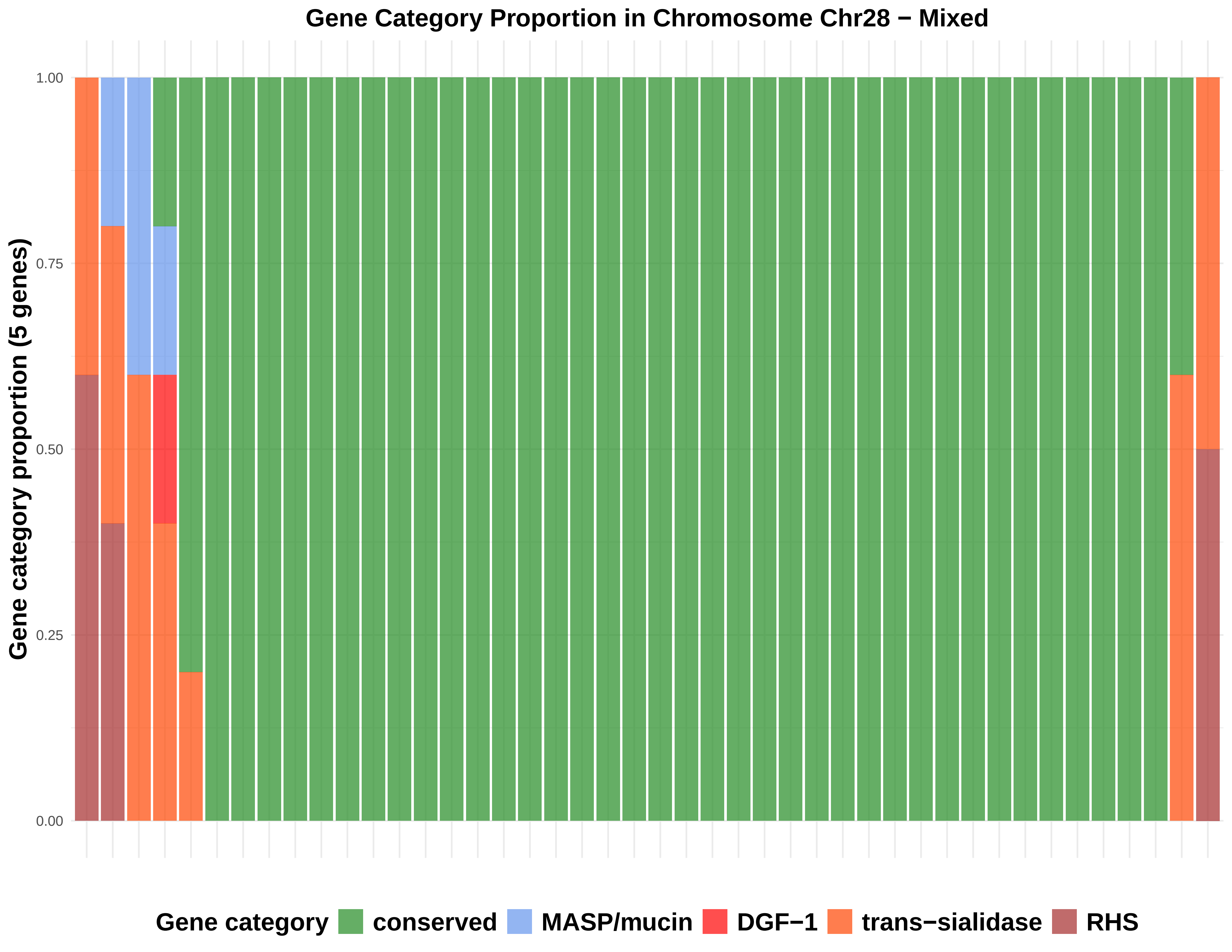

Gene Category Proportion in Chromosome Chr28 − Mixed
Gene category proportion (5 genes)
1.00
0.75
0.50
0.25
0.00
Gene category
conserved
MASP/mucin
DGF−1
trans−sialidase
RHS

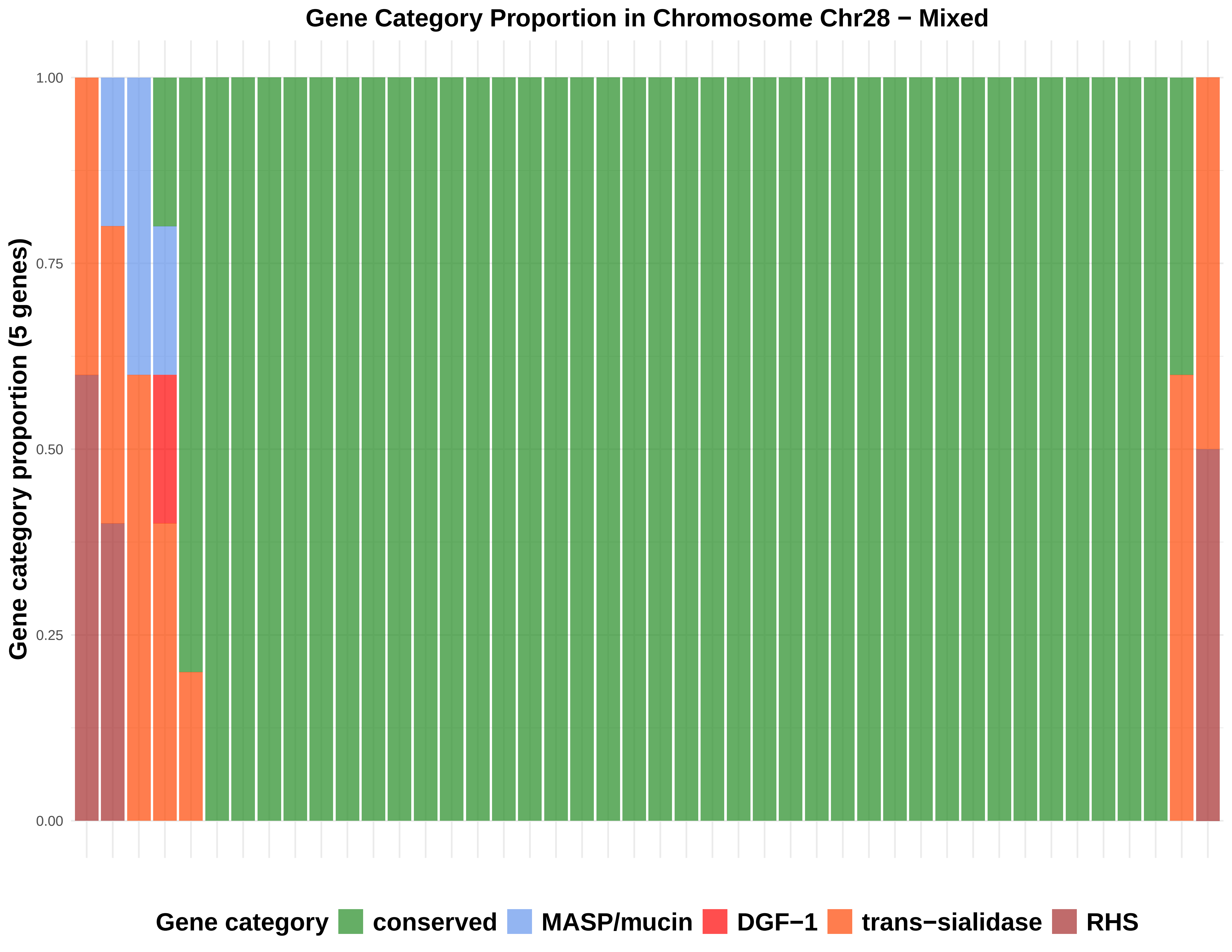

Gene Category Proportion in Chromosome Chr28 − Mixed
Gene category proportion (5 genes)
1.00
0.75
0.50
0.25
0.00
Gene category
conserved
MASP/mucin
DGF−1
trans−sialidase
RHS

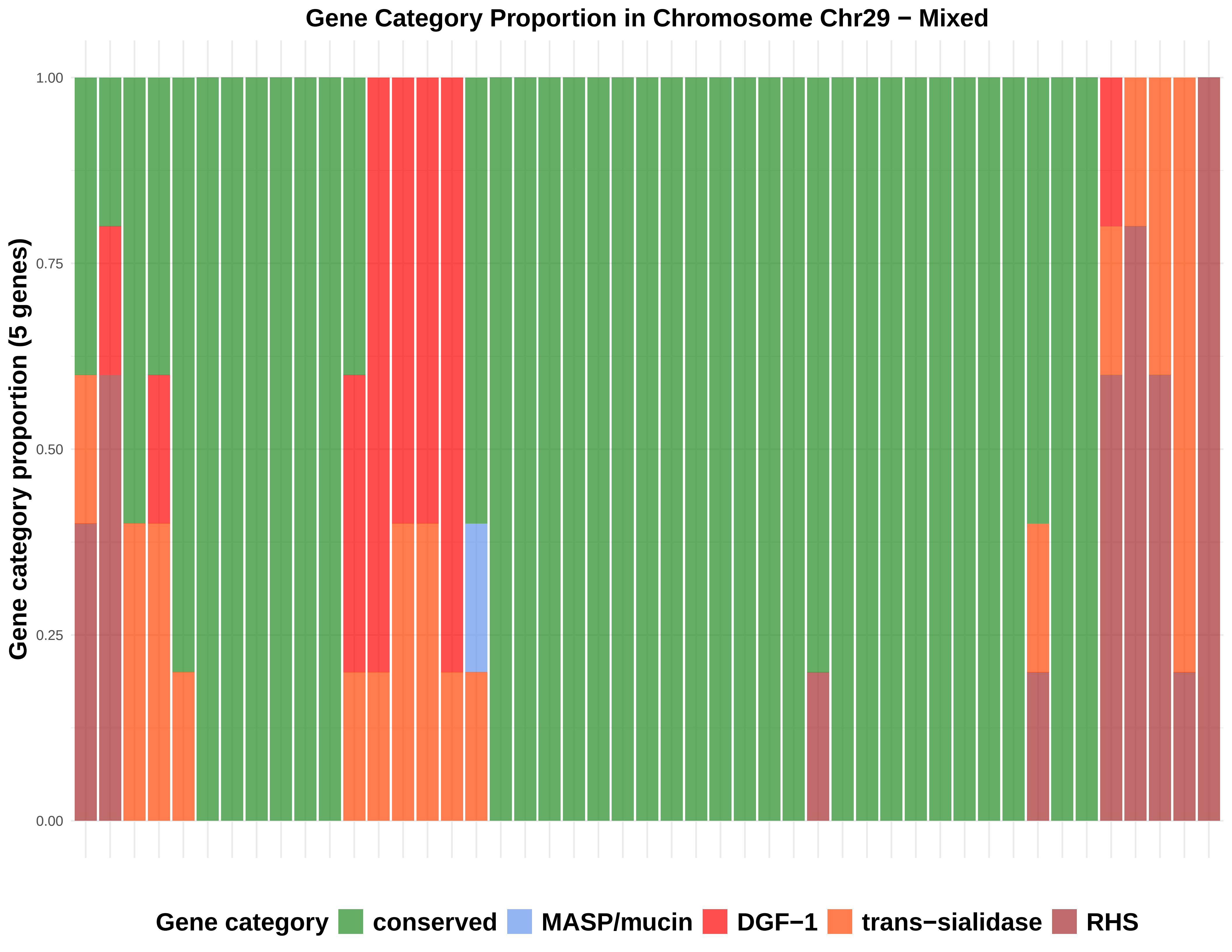
Gene Category Proportion in Chromosome Chr29 − Mixed
Gene category proportion (5 genes)
1.00
0.75
0.50
0.25
0.00
Gene category
conserved
MASP/mucin
DGF−1
trans−sialidase
RHS

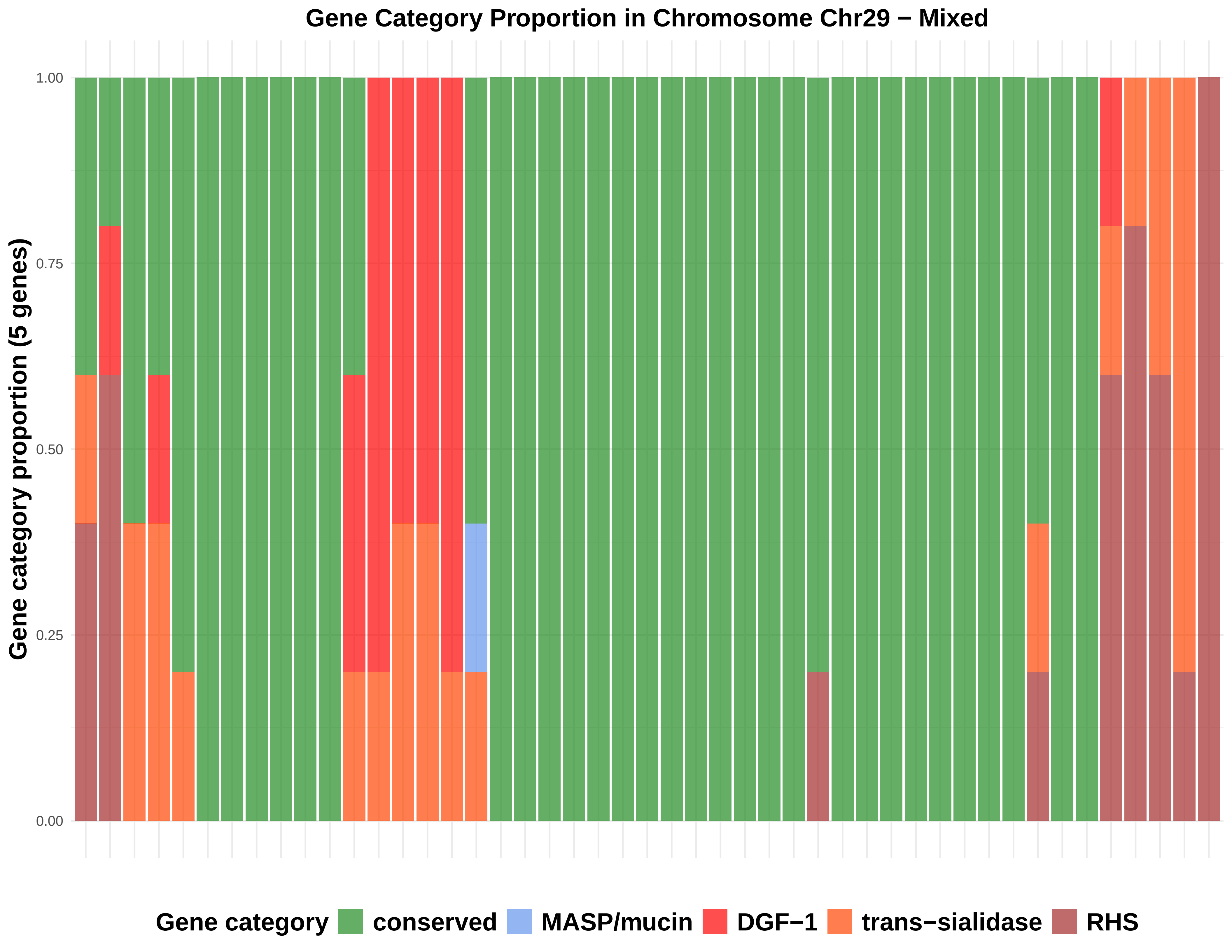
Gene Category Proportion in Chromosome Chr29 − Mixed
Gene category proportion (5 genes)
1.00
0.75
0.50
0.25
0.00
Gene category
conserved
MASP/mucin
DGF−1
trans−sialidase
RHS

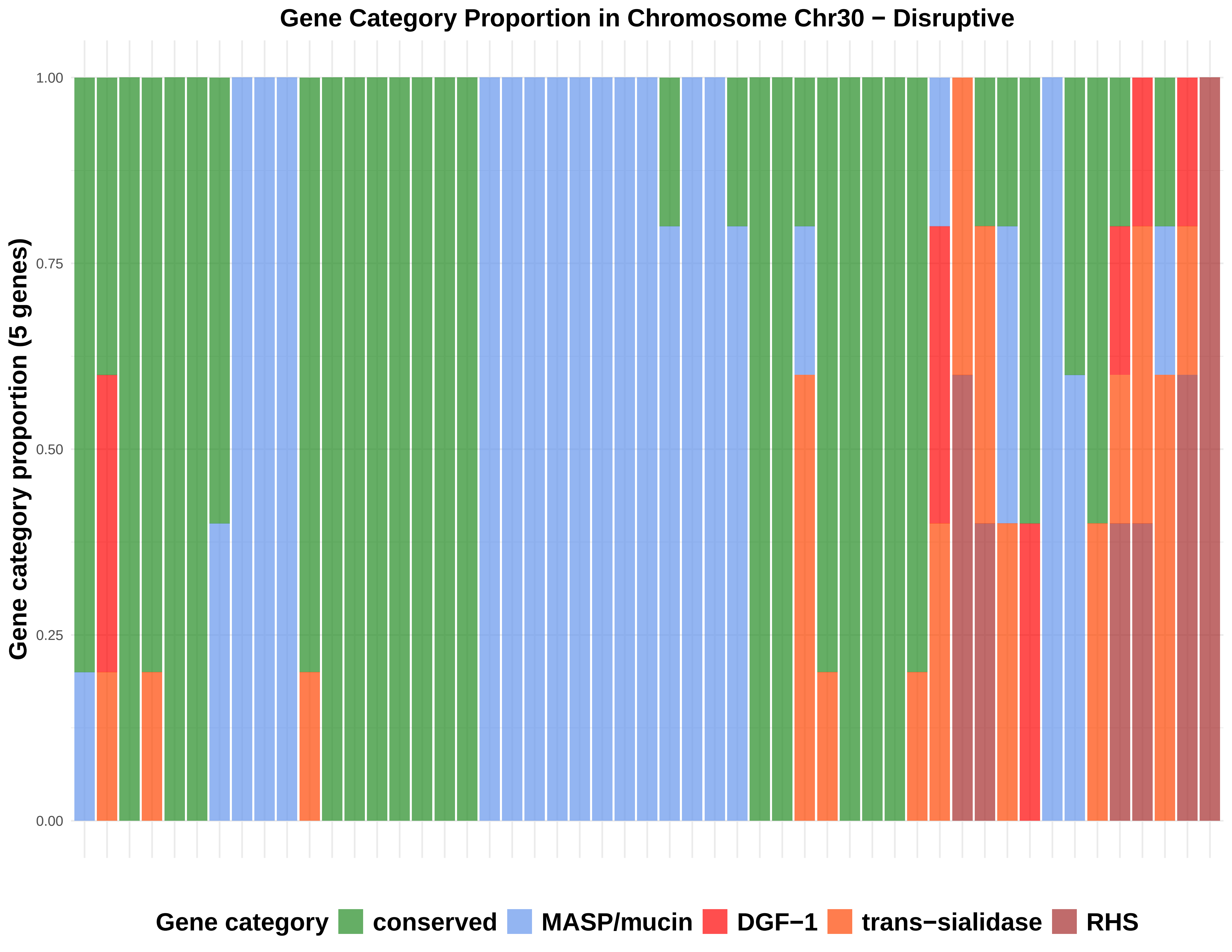
Gene Category Proportion in Chromosome Chr30 − Disruptive
Gene category proportion (5 genes)
1.00
0.75
0.50
0.25
0.00
Gene category
conserved
MASP/mucin
DGF−1
trans−sialidase
RHS

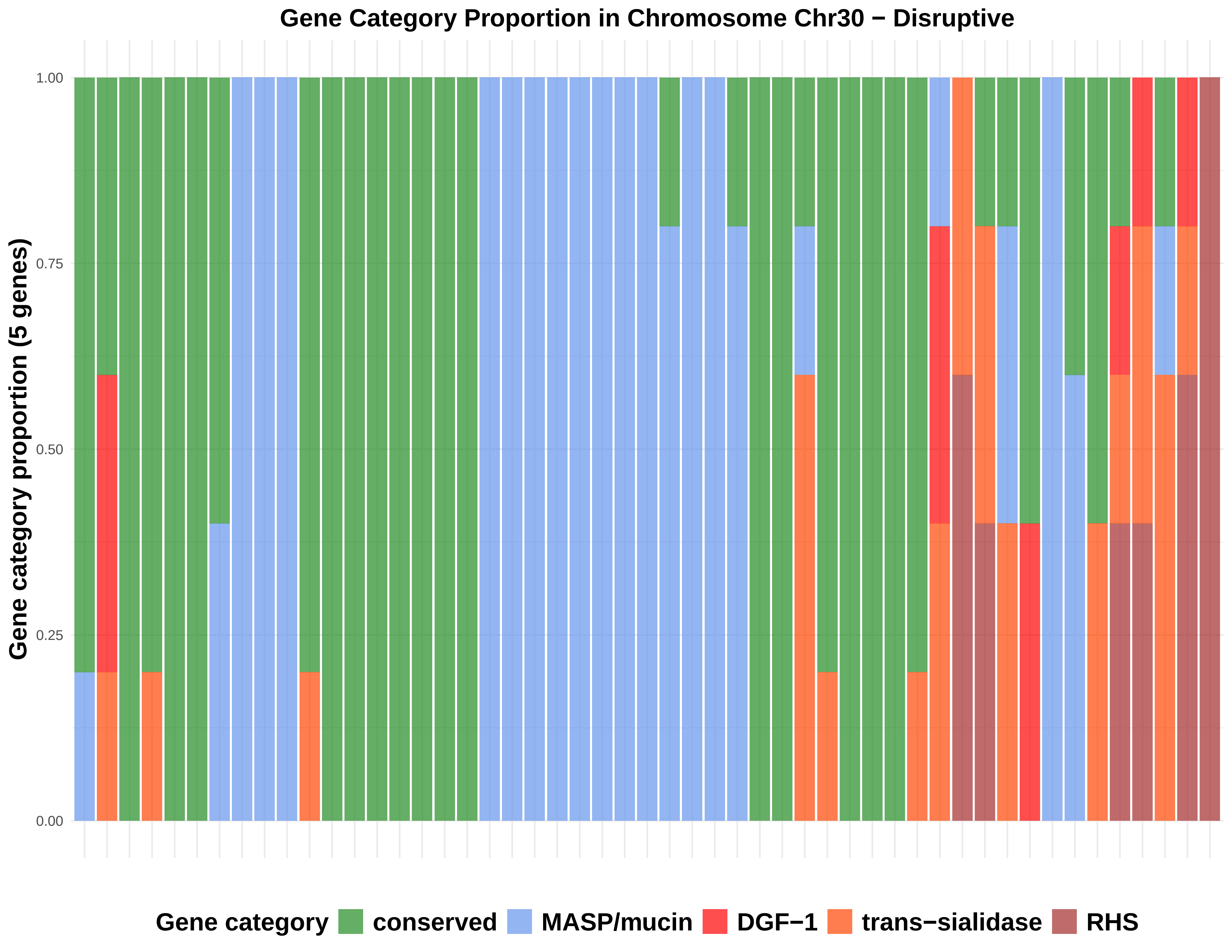

Gene Category Proportion in Chromosome Chr30 − Disruptive
Gene category proportion (5 genes)
1.00
0.75
0.50
0.25
0.00
Gene category
conserved
MASP/mucin
DGF−1
trans−sialidase
RHS

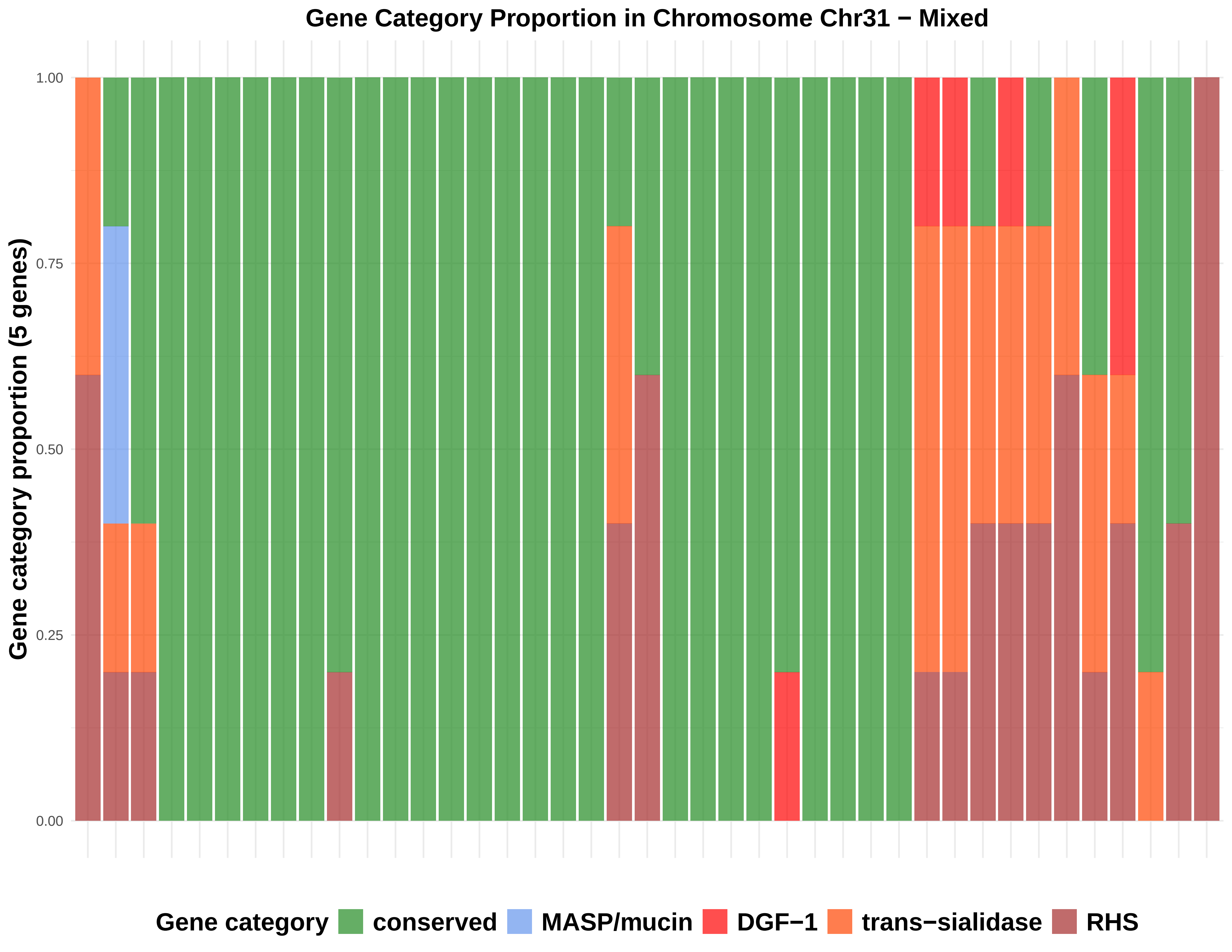

Gene Category Proportion in Chromosome Chr31 − Mixed
Gene category proportion (5 genes)
1.00
0.75
0.50
0.25
0.00
Gene category
conserved
MASP/mucin
DGF−1
trans−sialidase
RHS

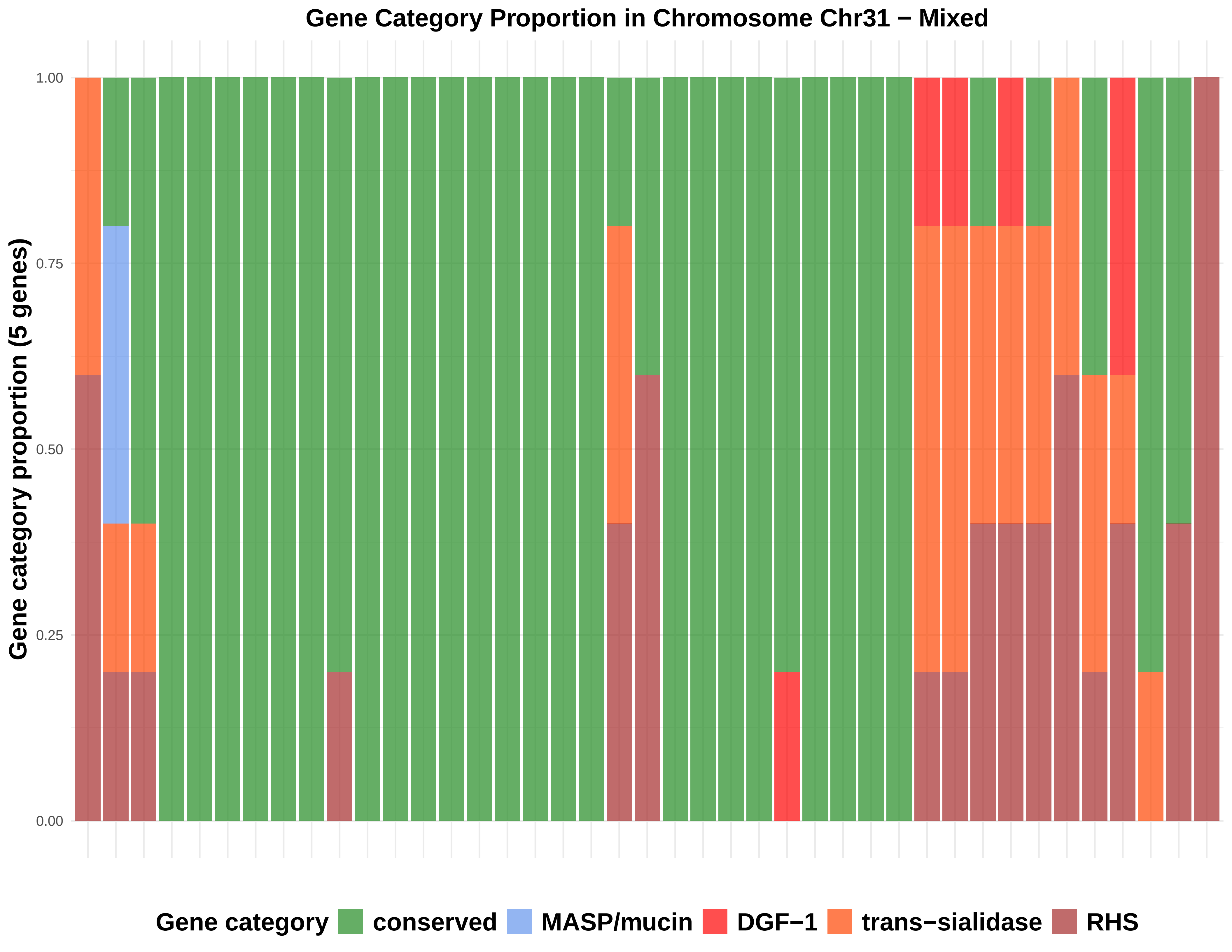

Gene Category Proportion in Chromosome Chr31 − Mixed
Gene category proportion (5 genes)
1.00
0.75
0.50
0.25
0.00
Gene category
conserved
MASP/mucin
DGF−1
trans−sialidase
RHS

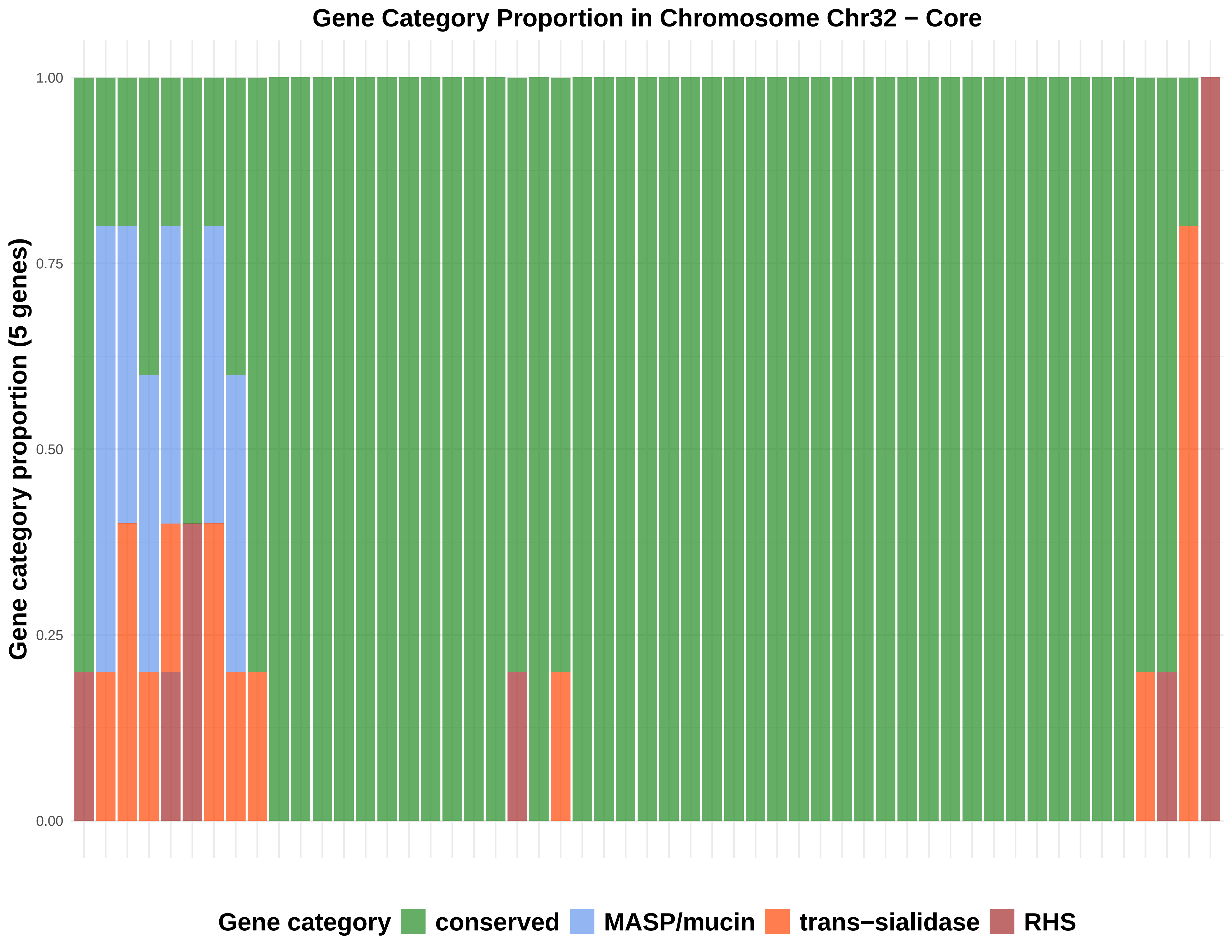
Gene Category Proportion in Chromosome Chr32 − Core
Gene category proportion (5 genes)
1.00
0.75
0.50
0.25
0.00
Gene category
conserved
MASP/mucin
trans−sialidase
RHS

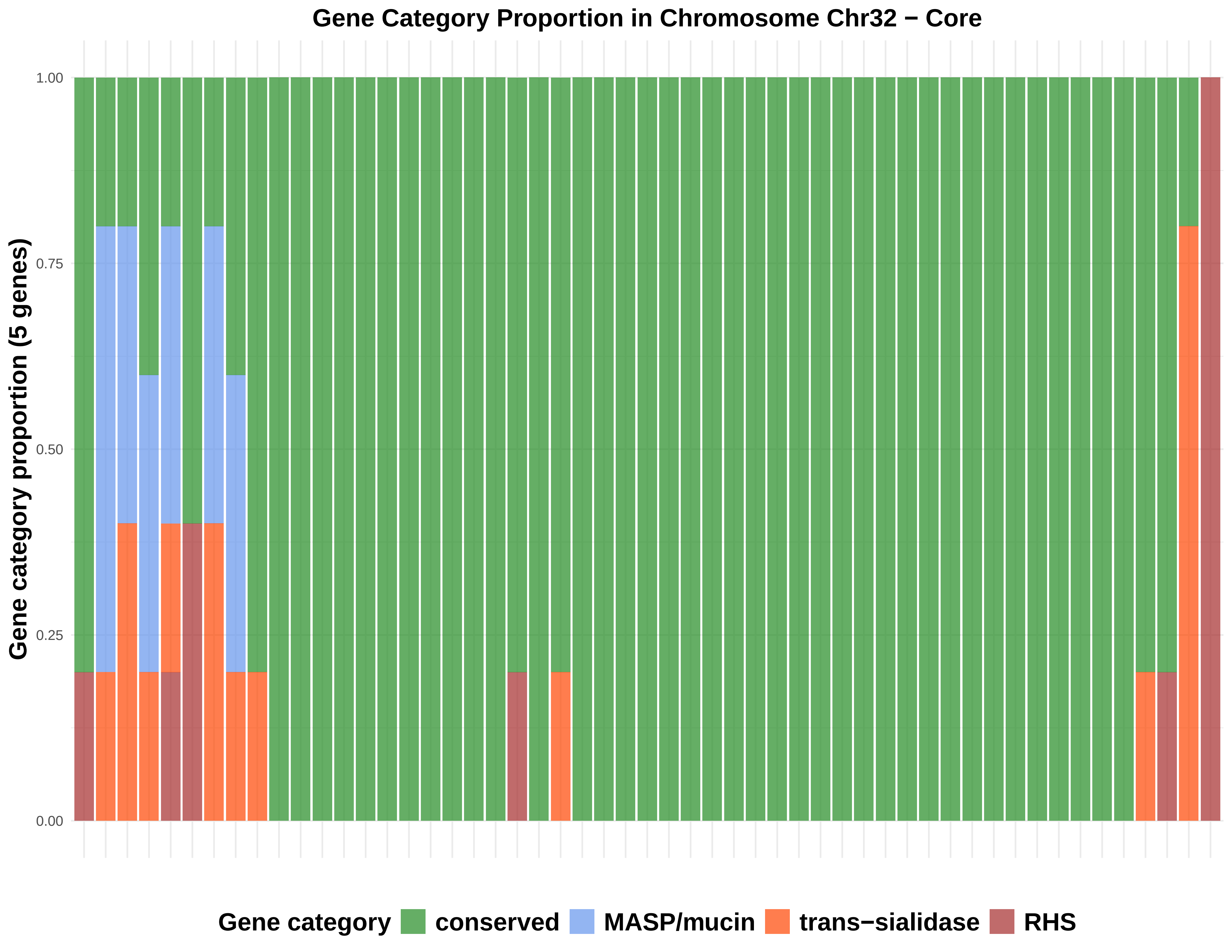

Gene Category Proportion in Chromosome Chr32 − Core
Gene category proportion (5 genes)
1.00
0.75
0.50
0.25
0.00
Gene category
conserved
MASP/mucin
trans−sialidase
RHS
