# Supplementary material for: The complete genome of Trypanosoma cruzi reveals 32 chromosomes and three genomic compartments
Source: BMC Genomics. 2026 Jan 8;27:159. doi: 10.1186/s12864-025-12482-0 (PMC12879350; doi:10.1186/s12864-025-12482-0)

Supplementary Figure 9.

A. Pages 2 to 4. Boxplot of gene density (number of genes/Mb) by category in internal and subtelomeric regions in Core, Disruptive and Mixed chromosomes.
B. Page5. Boxplot of gene density of pseudogenes.

A.

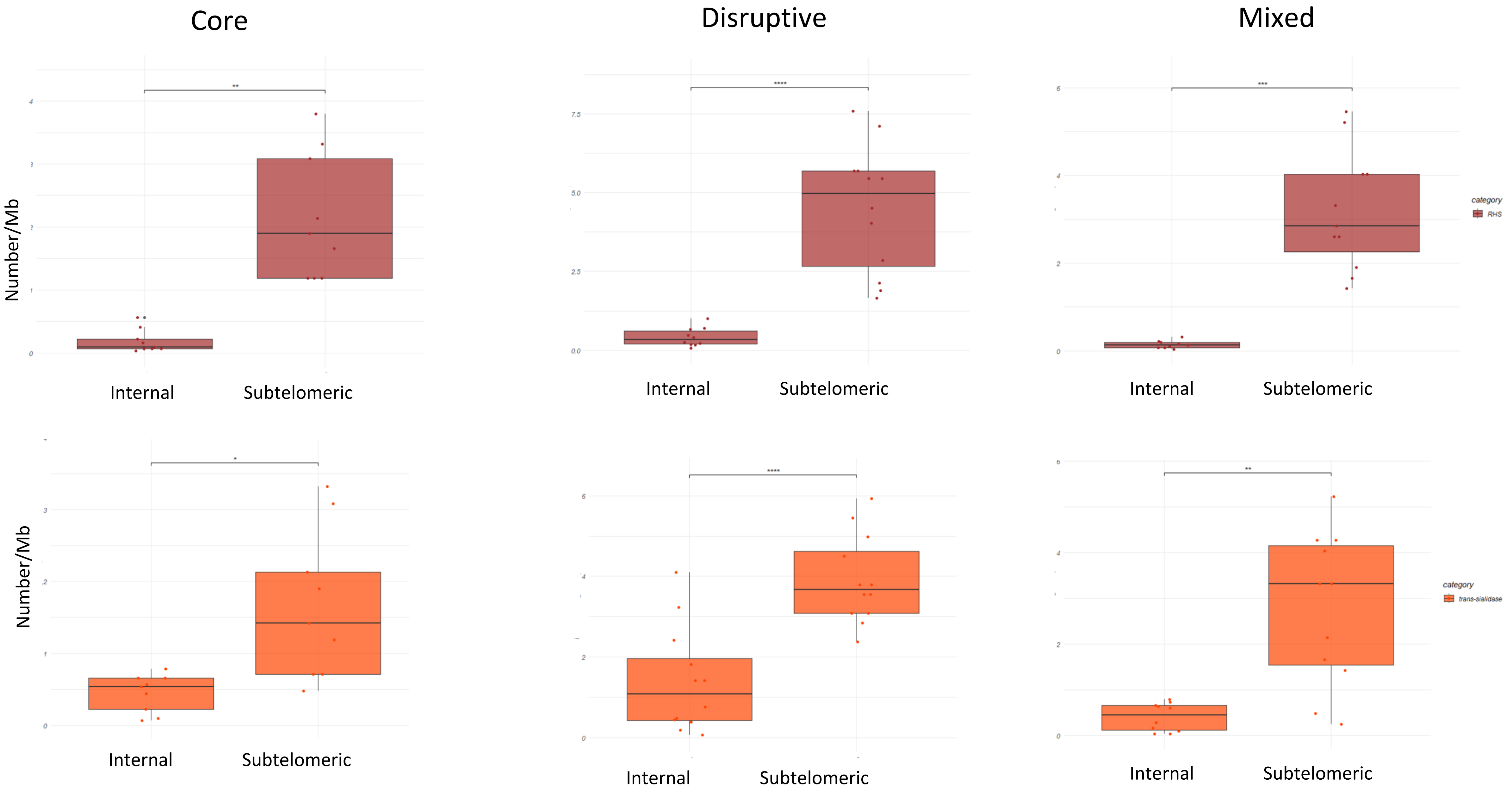
Core
Disruptive
Mixed
Number/Mb
Internal
Subtelomeric
category
RHS
trans-sialidase

A (continue)

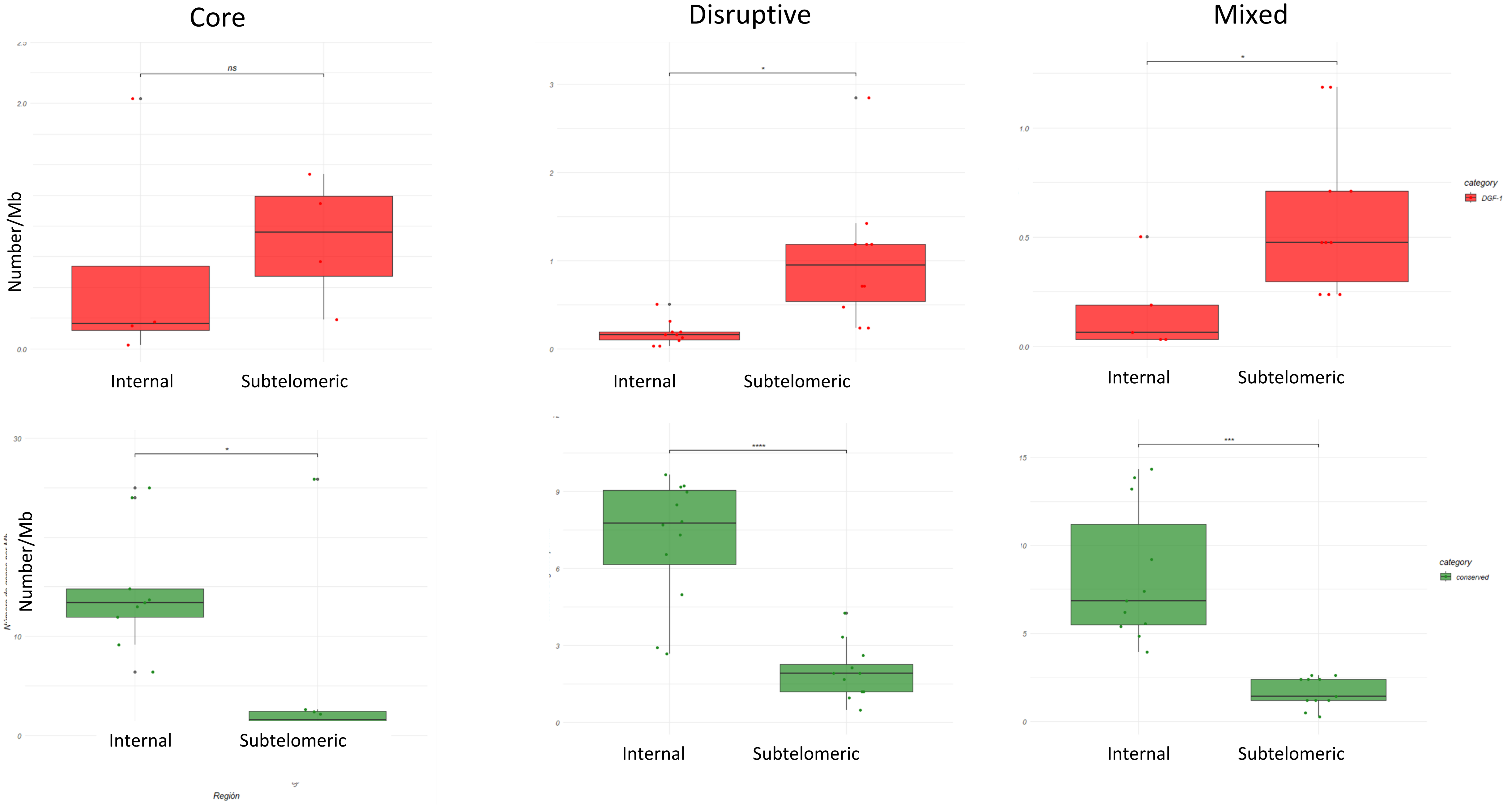

Core
Disruptive
Mixed
Number/Mb
ns
*
*
category
DGF-1
Internal
Subtelomeric
*
****
***
Number/Mb
category
conserved
Internal
Subtelomeric
Región

A (continue)

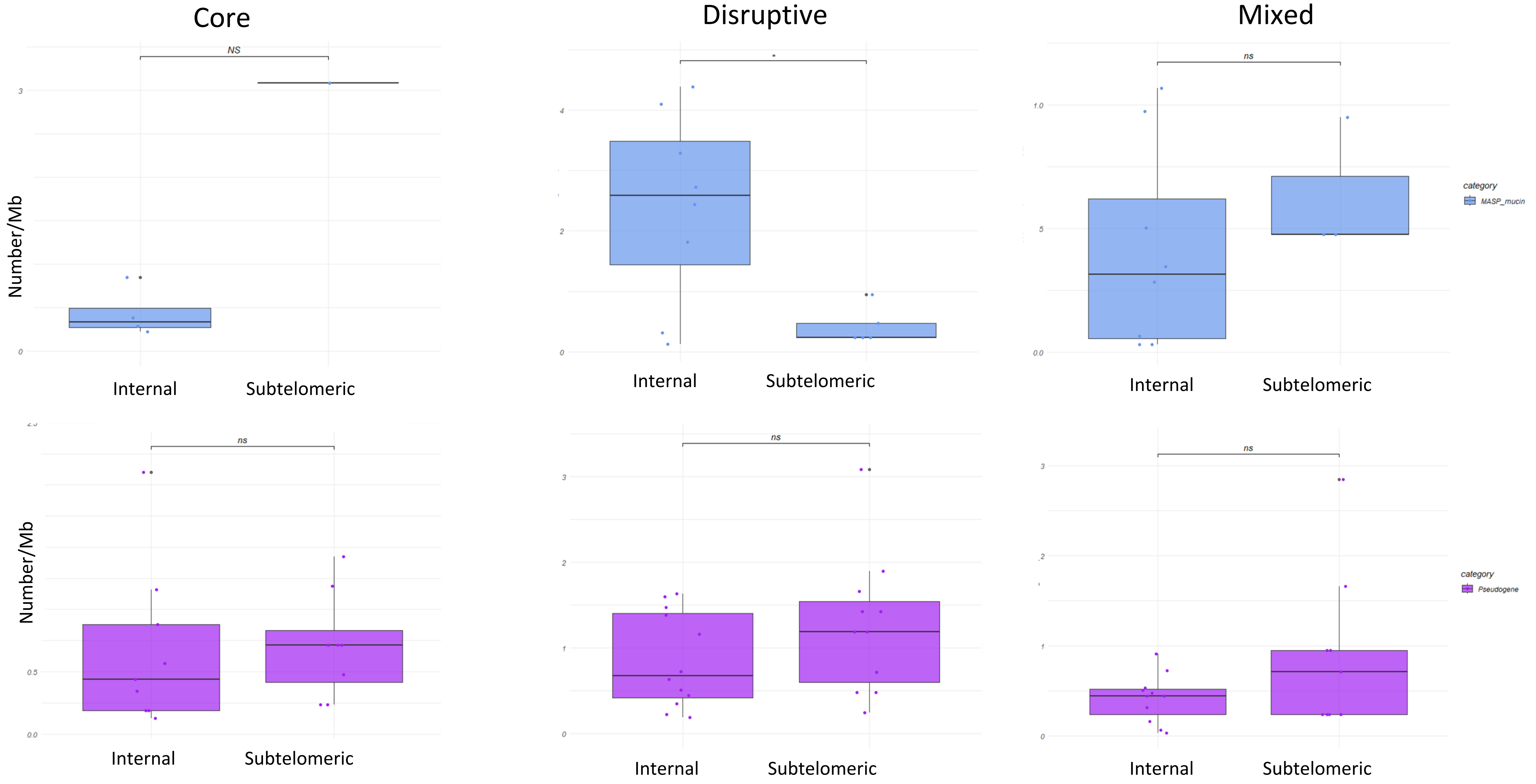
Core
Disruptive
Mixed
NS
*
ns
ns
ns
ns
Number/Mb
Number/Mb
Internal
Subtelomeric
Internal
Subtelomeric
Internal
Subtelomeric
Internal
Subtelomeric
Internal
Subtelomeric
Internal
Subtelomeric
category
MASP_mucin
category
Pseudogene

B.

Pseudogene enrichment in subtelomeric región.

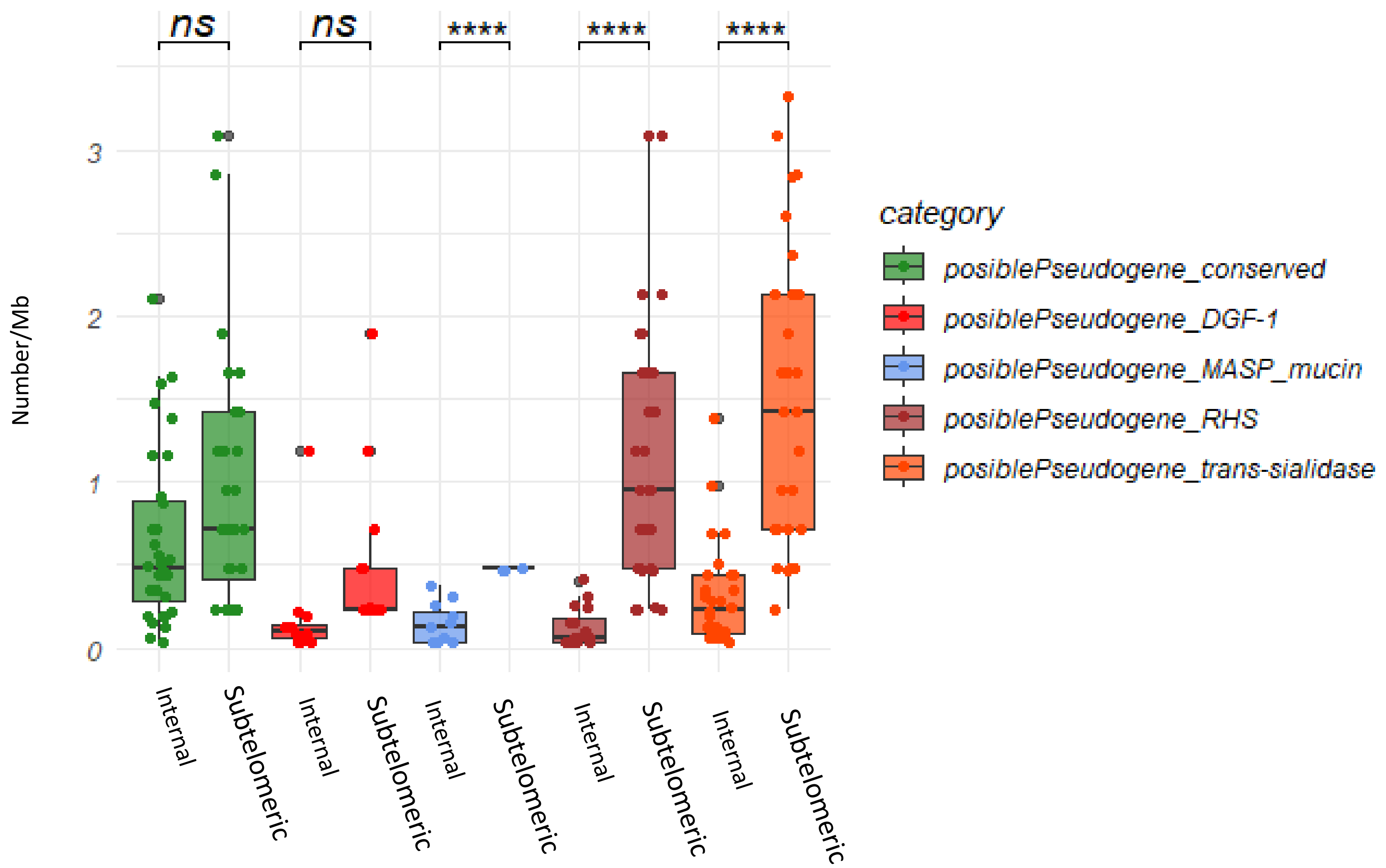

Supplement: Supplementary file 9 — Supplementary Material 9. [file 12864_2025_12482_MOESM9_ESM.pdf]
