# Supplementary material for: The complete genome of Trypanosoma cruzi reveals 32 chromosomes and three genomic compartments
Source: BMC Genomics. 2026 Jan 8;27:159. doi: 10.1186/s12864-025-12482-0 (PMC12879350; doi:10.1186/s12864-025-12482-0)

Supplementary Figure 7.

Core-Disruptive chromosomes structure conservation with Dm25 strain.

Chromosome 1

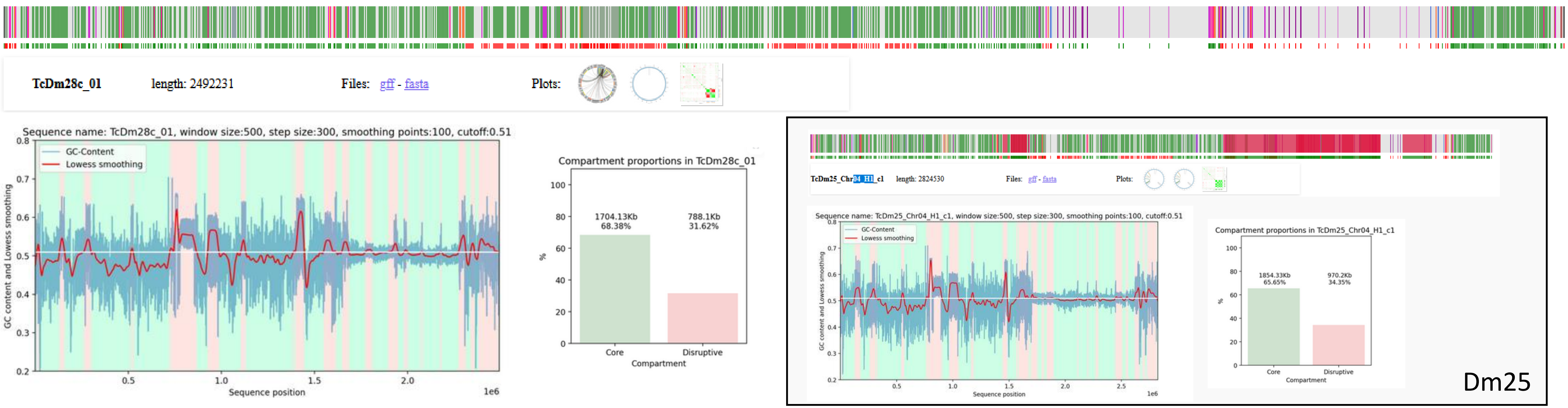

Chromosome 2

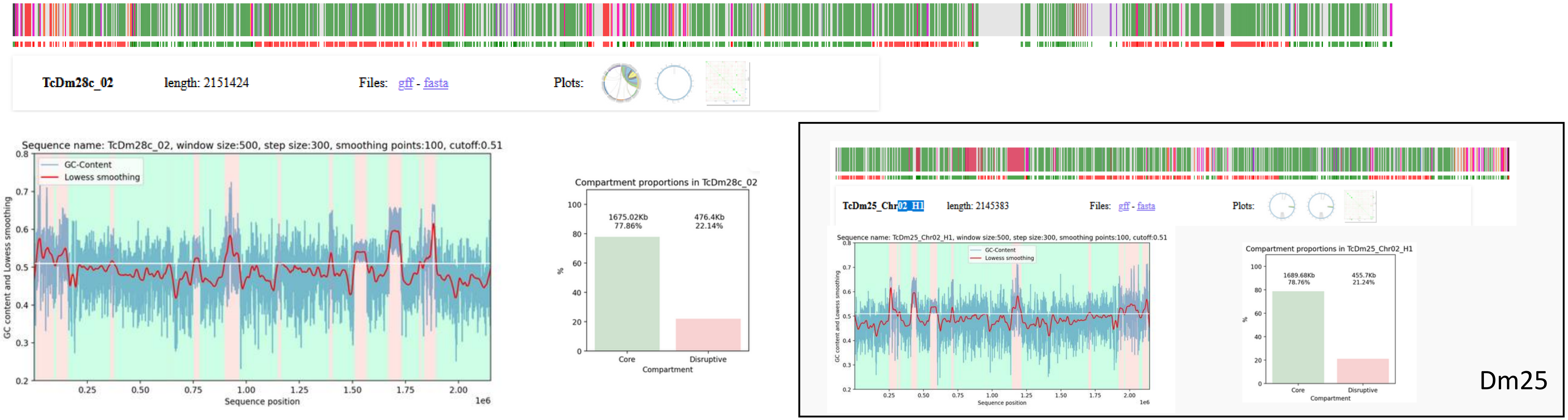

Chromosome 3

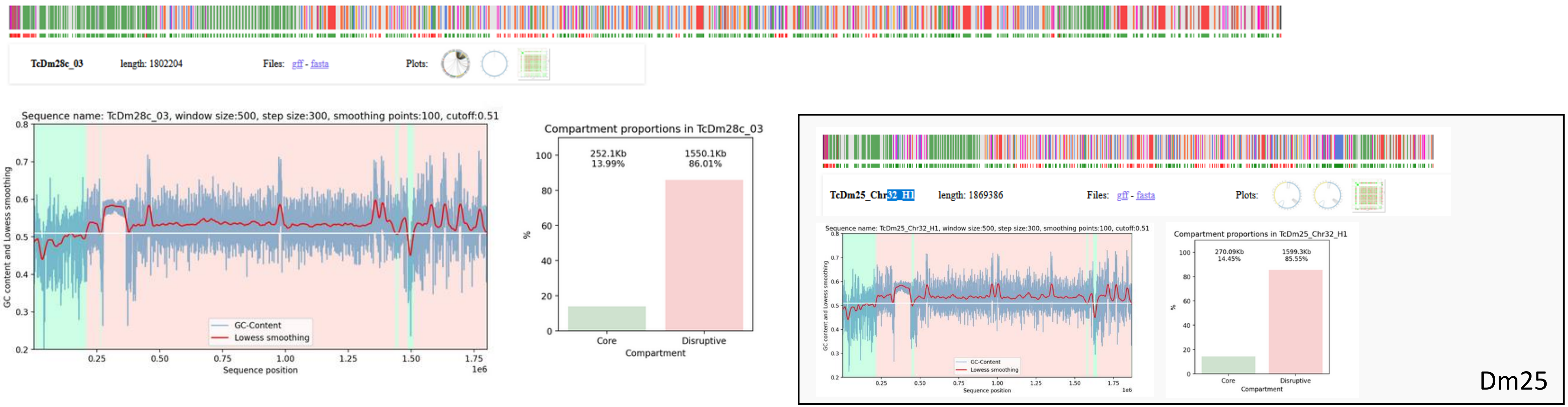

Chromosome 4

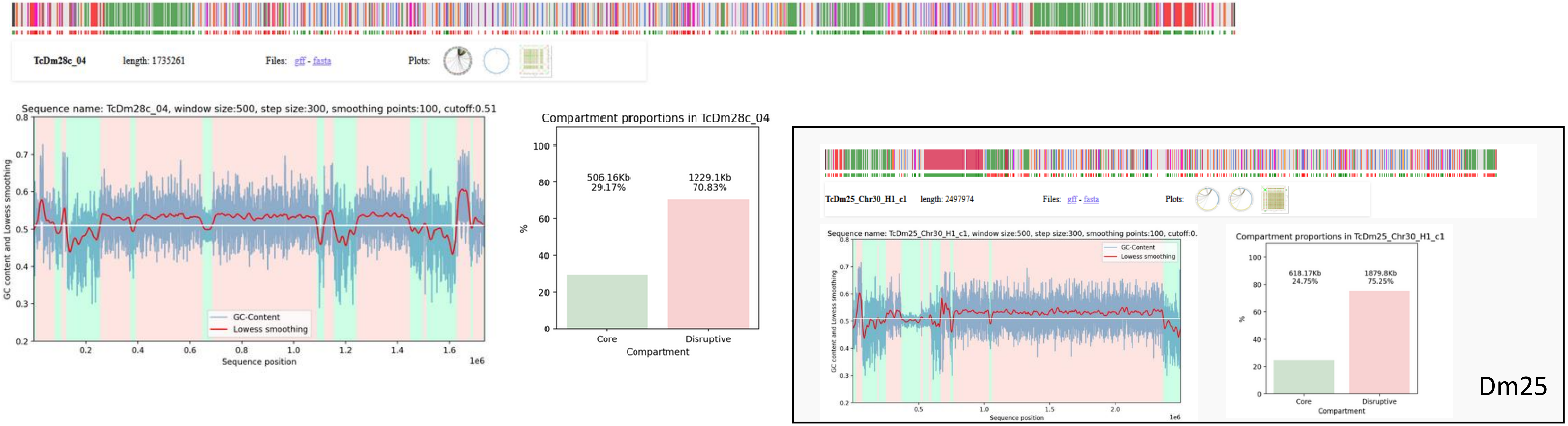

Chromosome 5

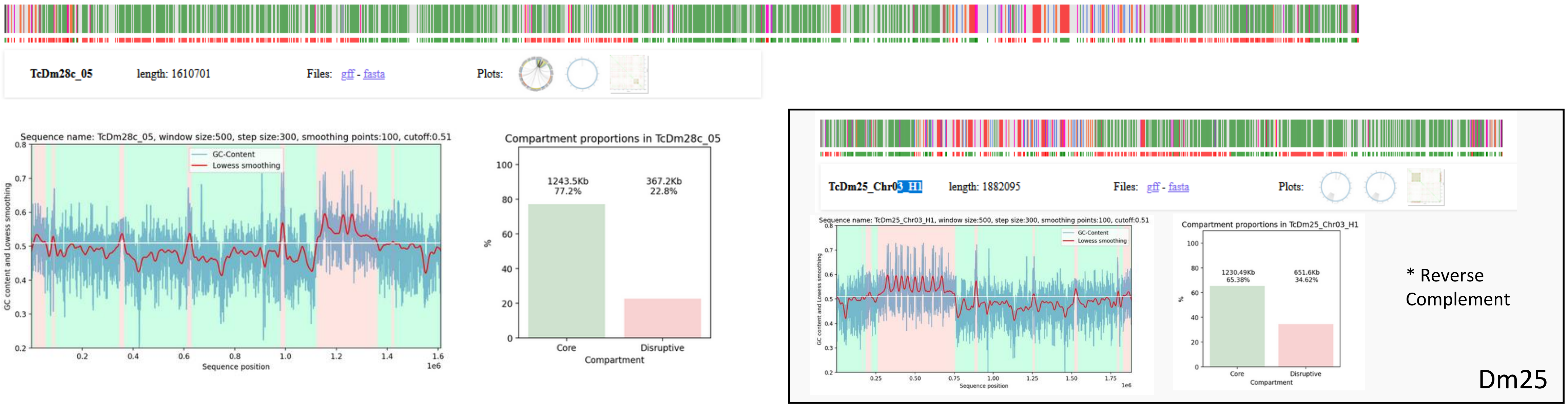

Chromosome 6

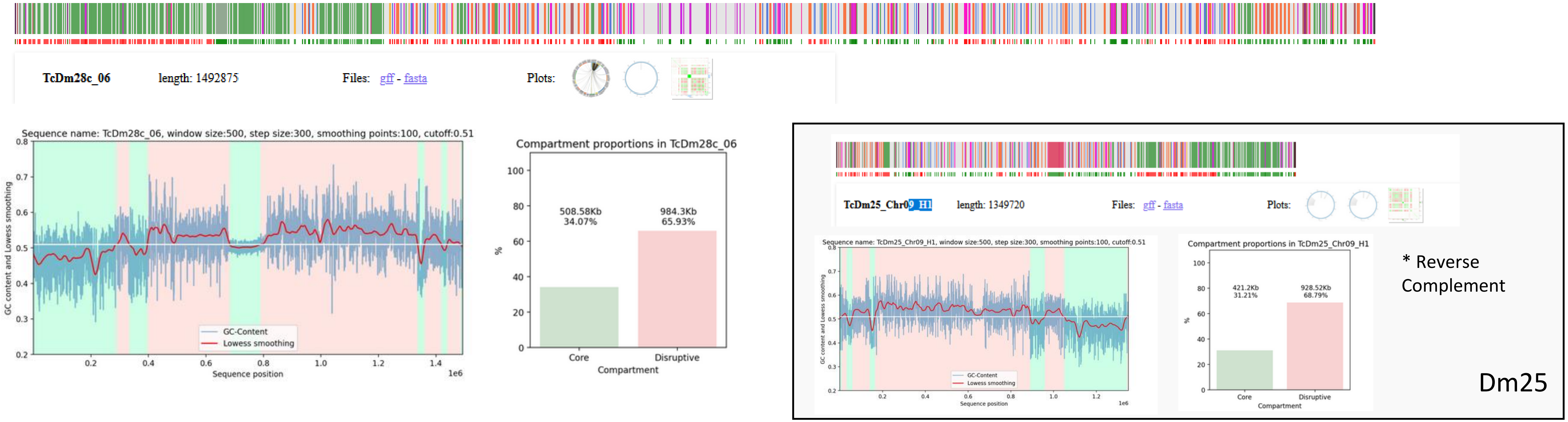

Chromosome 7

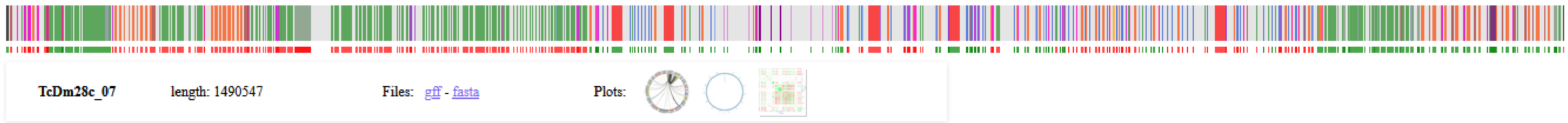

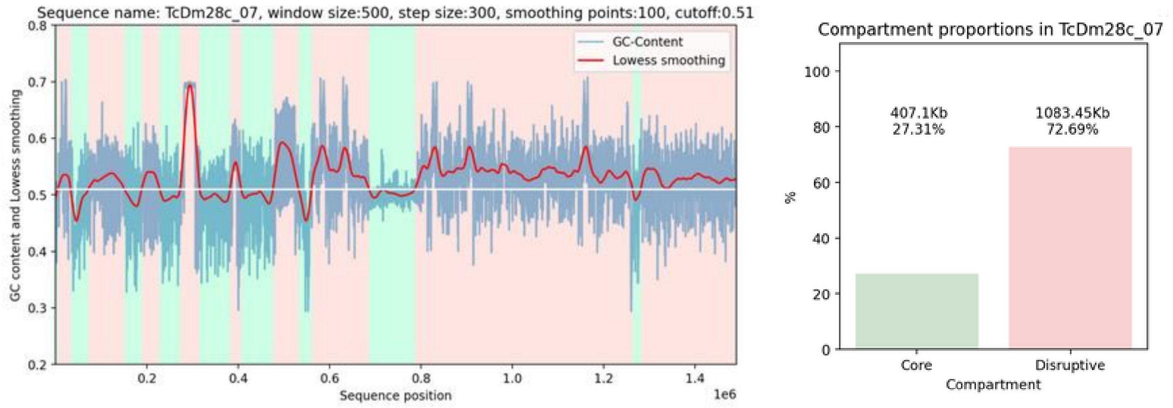

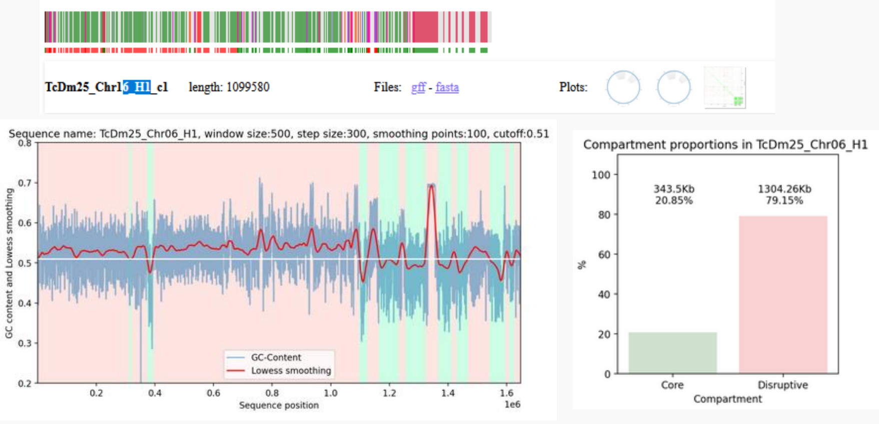

* Reverse Complement

Dm25

Chromosome 8

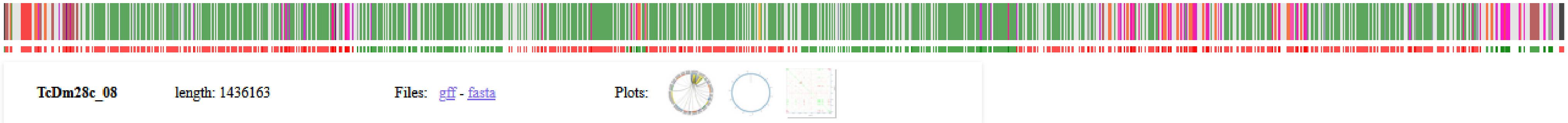

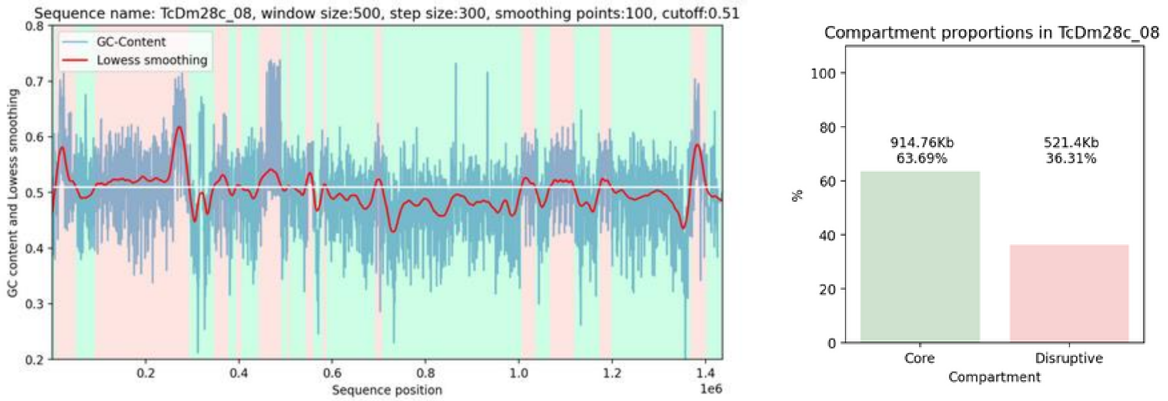

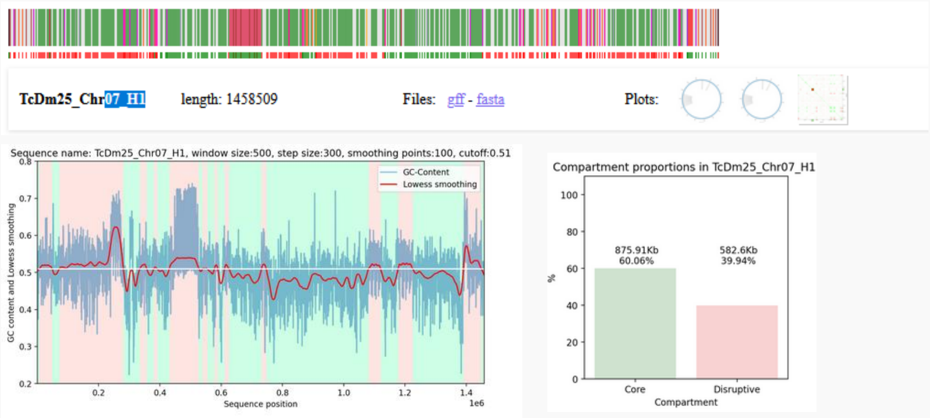

Dm25

Chromosome 9

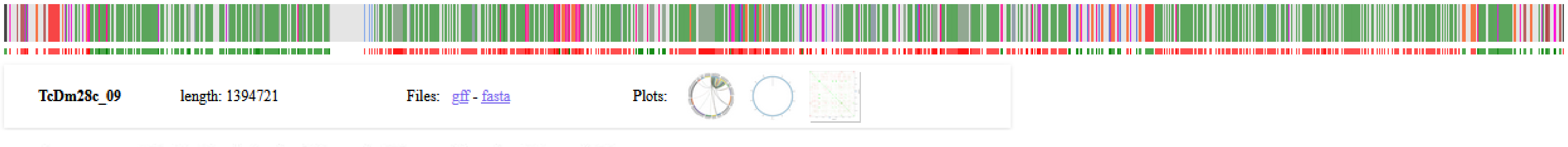

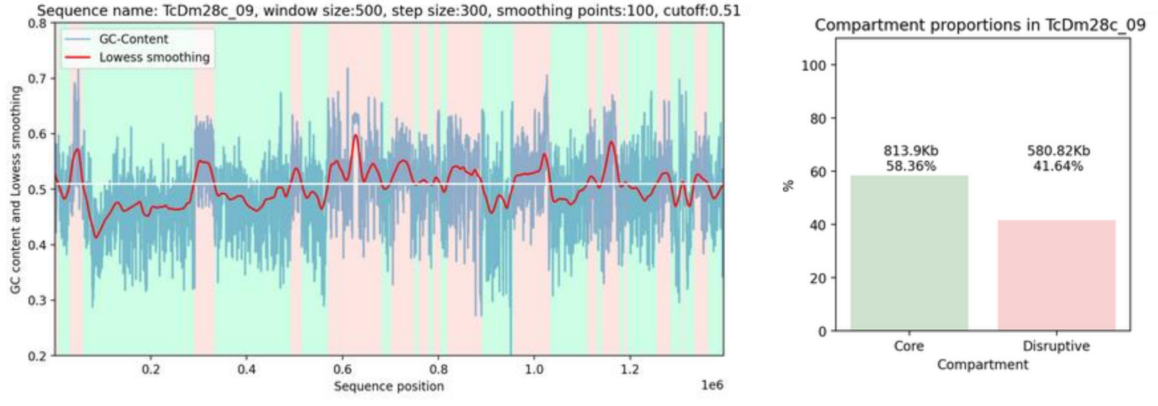

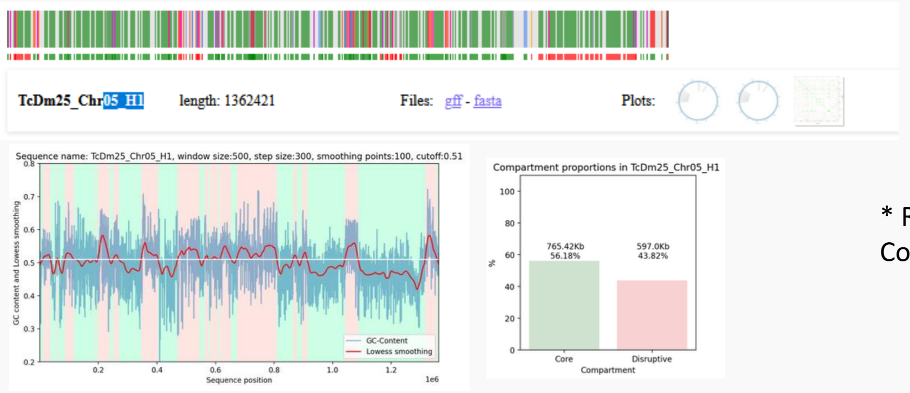

* Reverse Complement

Dm25

Chromosome 10

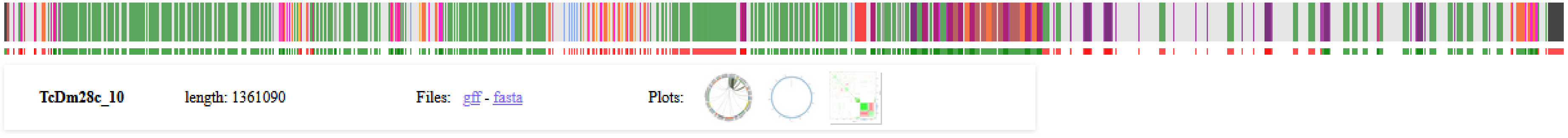

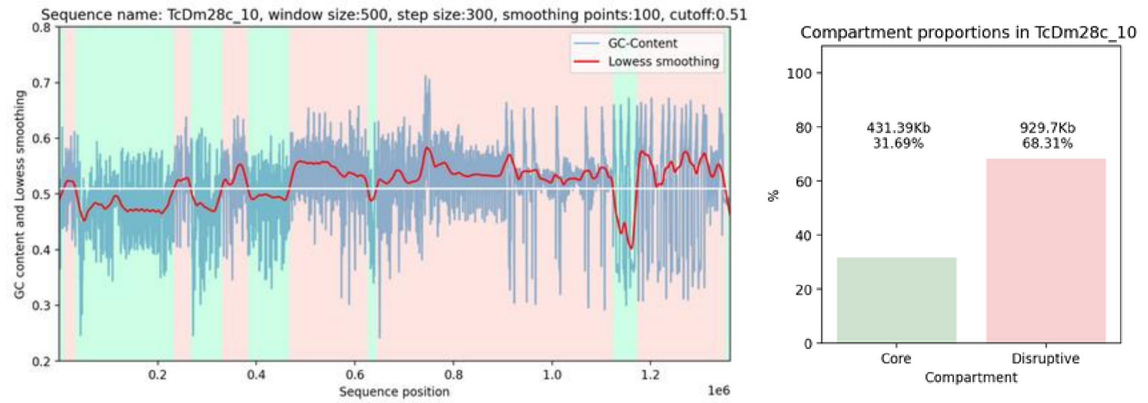

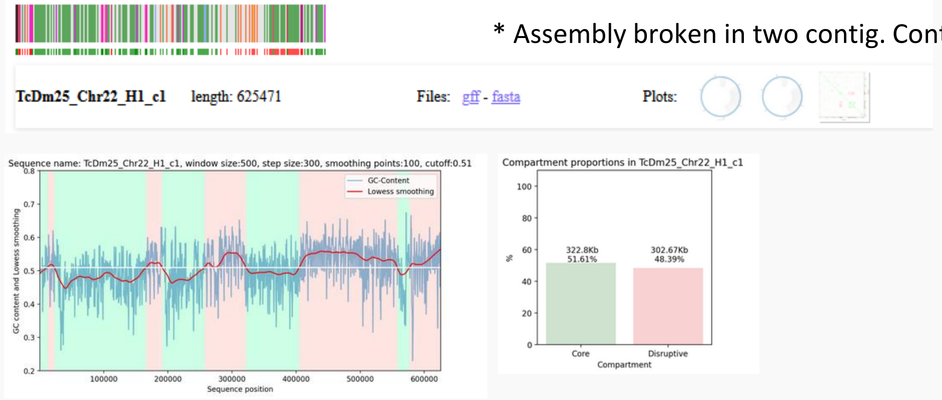

* Assembly broken in two contig. Contig 1

Dm25

Chromosome 11

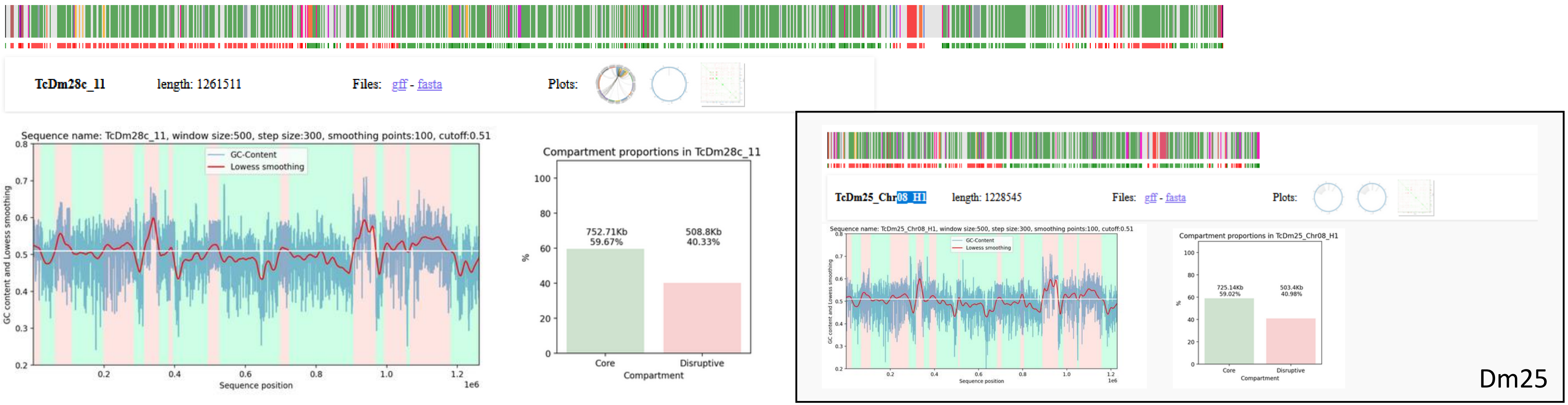

Chromosome 12

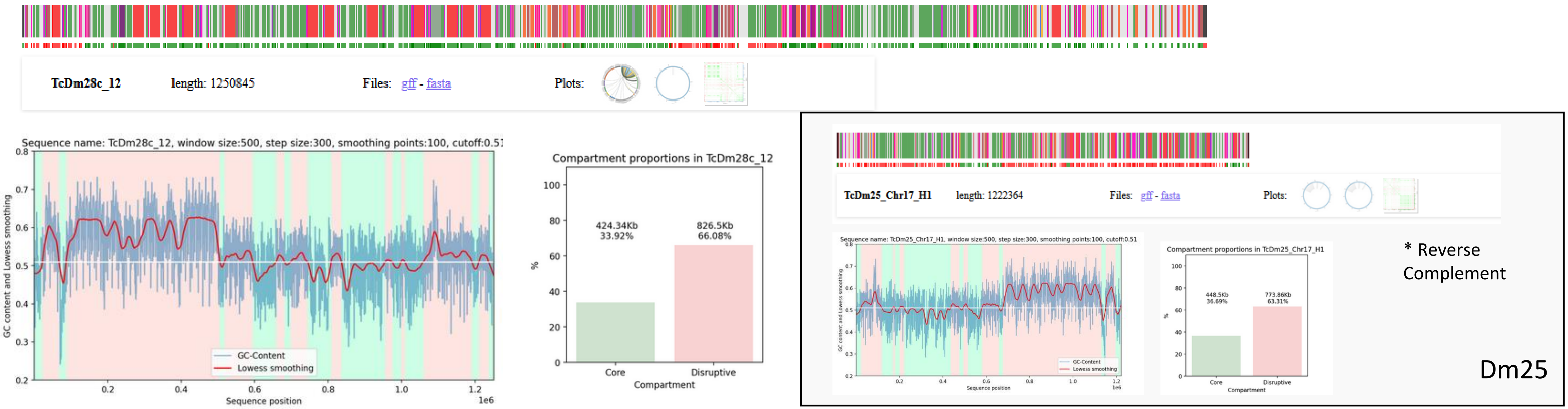

Chromosome 13

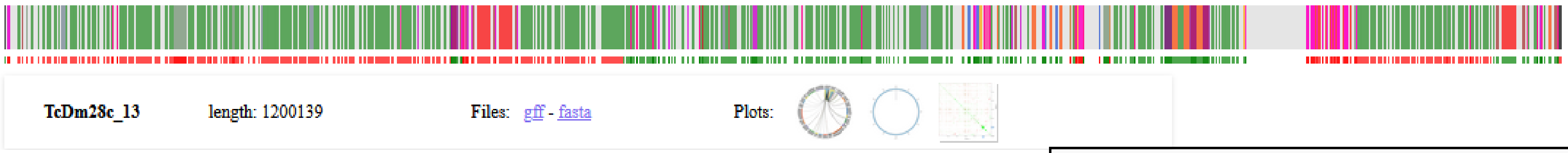

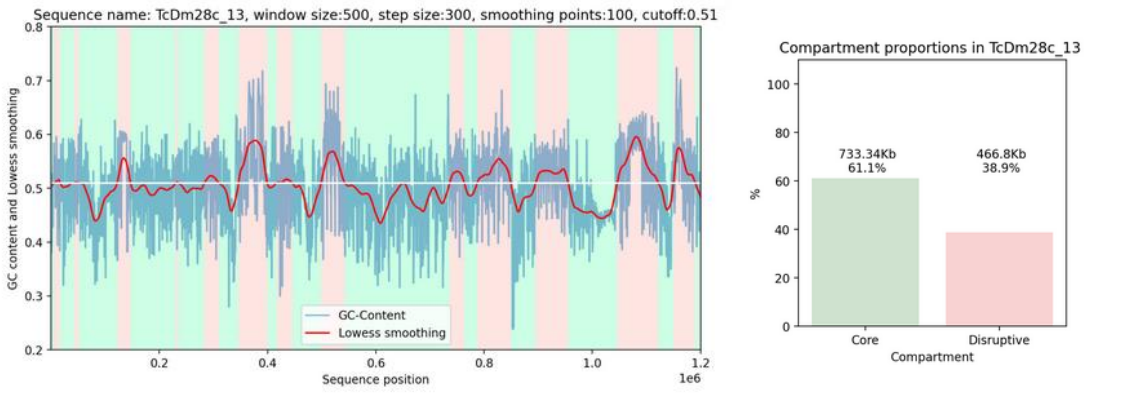

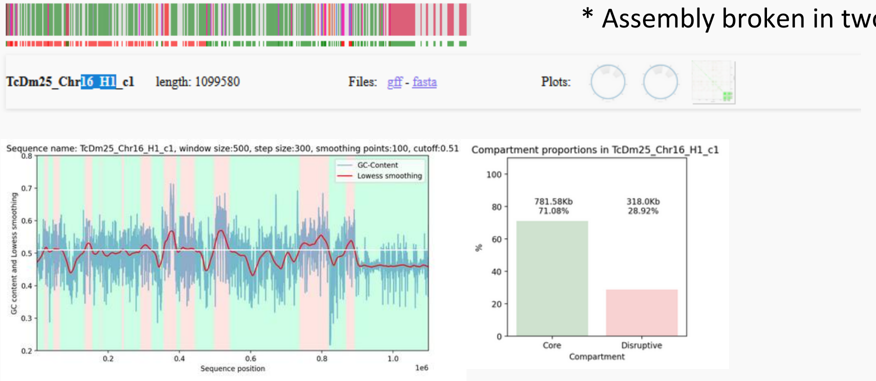

* Assembly broken in two contig. Contig 1

Dm25

Chromosome 14

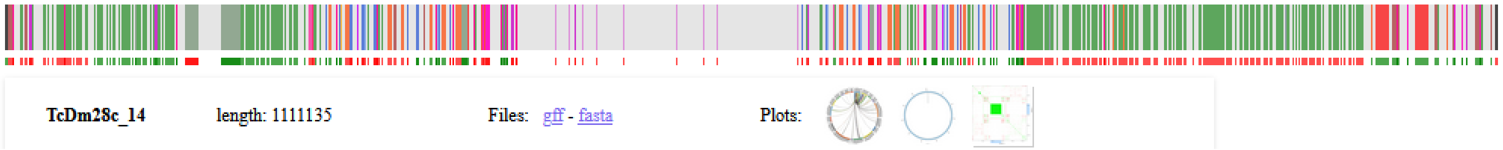

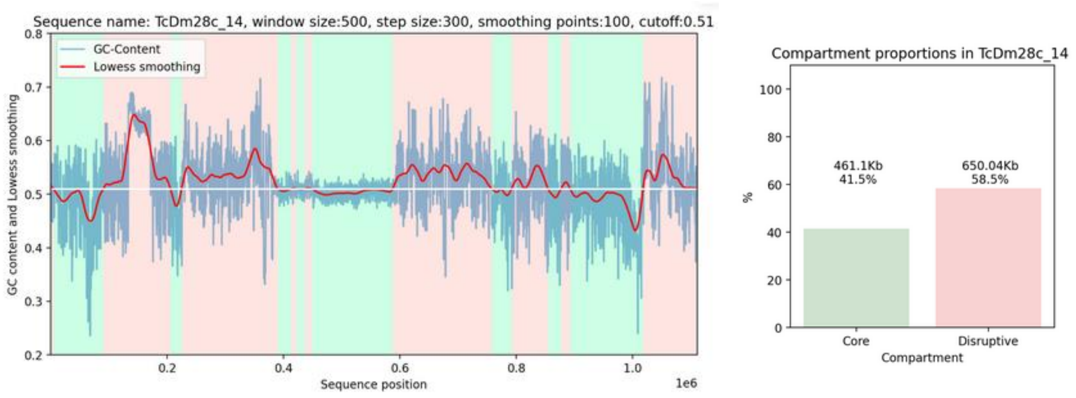

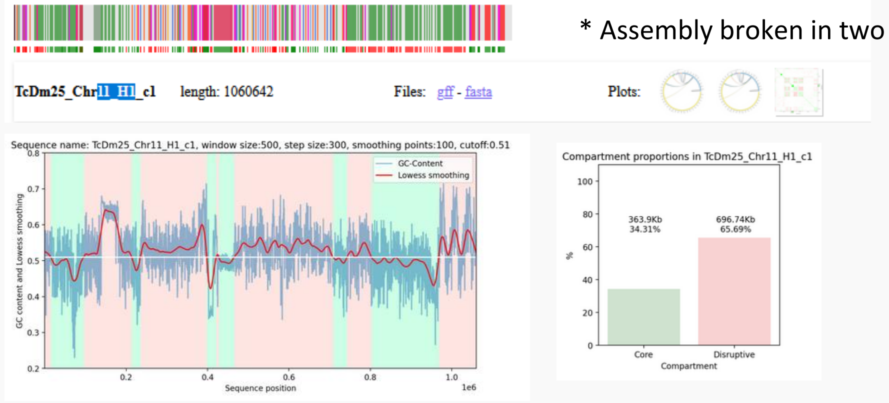

* Assembly broken in two contig. Contig 1

Dm25

Chromosome 15

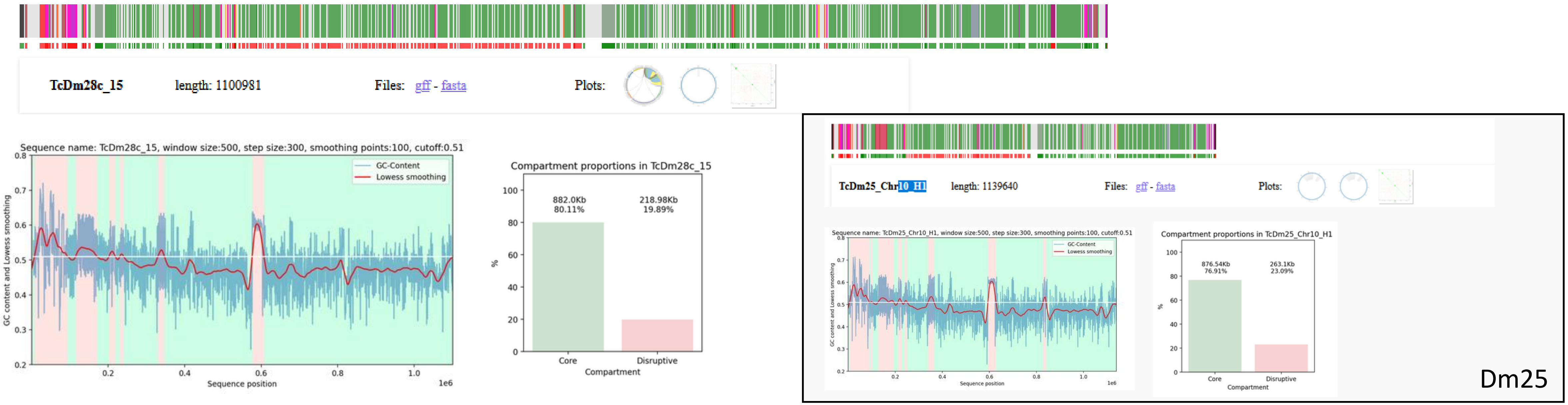

Chromosome 16

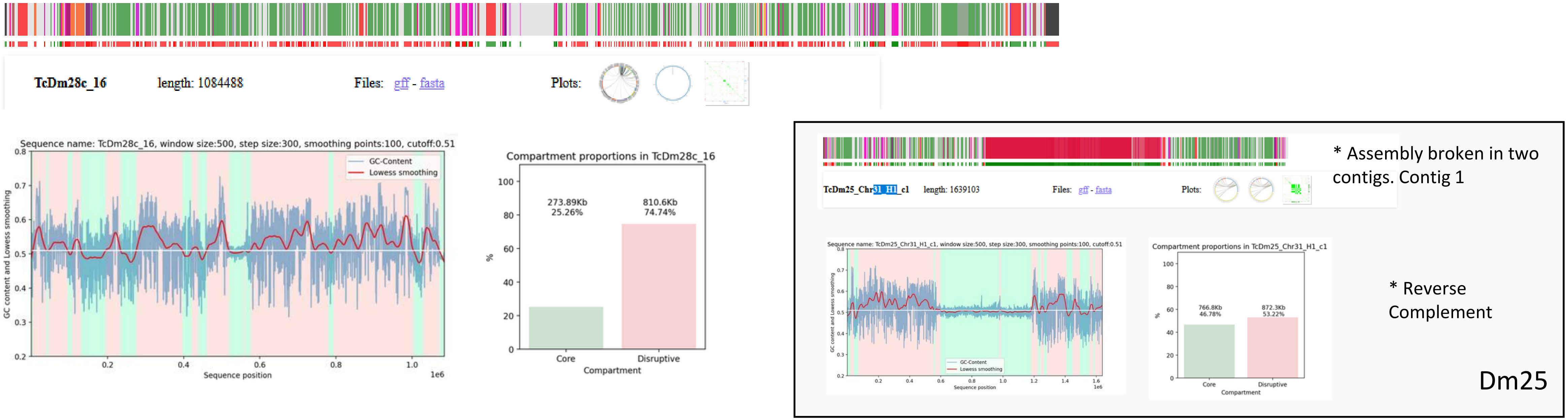

Chromosome 17

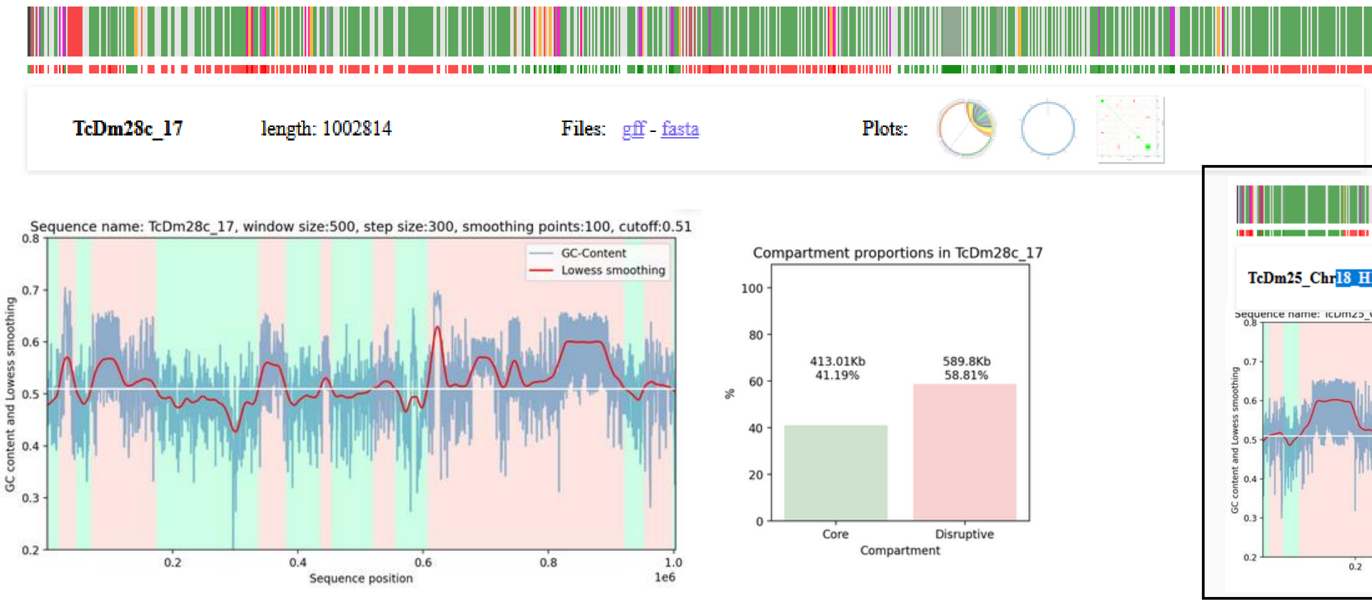

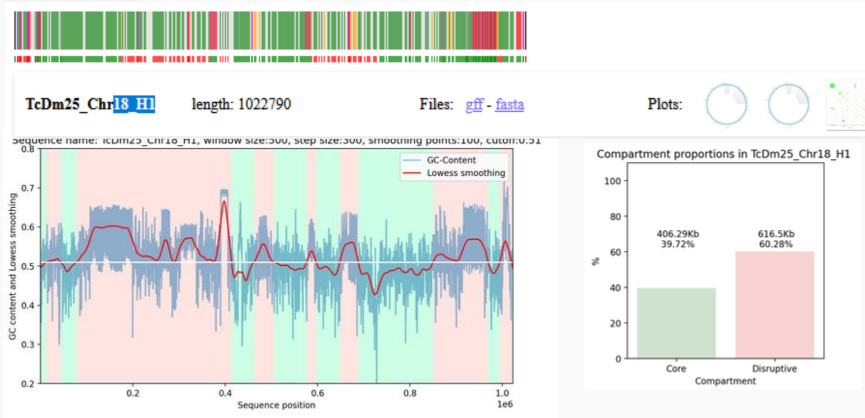

* Reverse Complement

Dm25

Chromosome 18

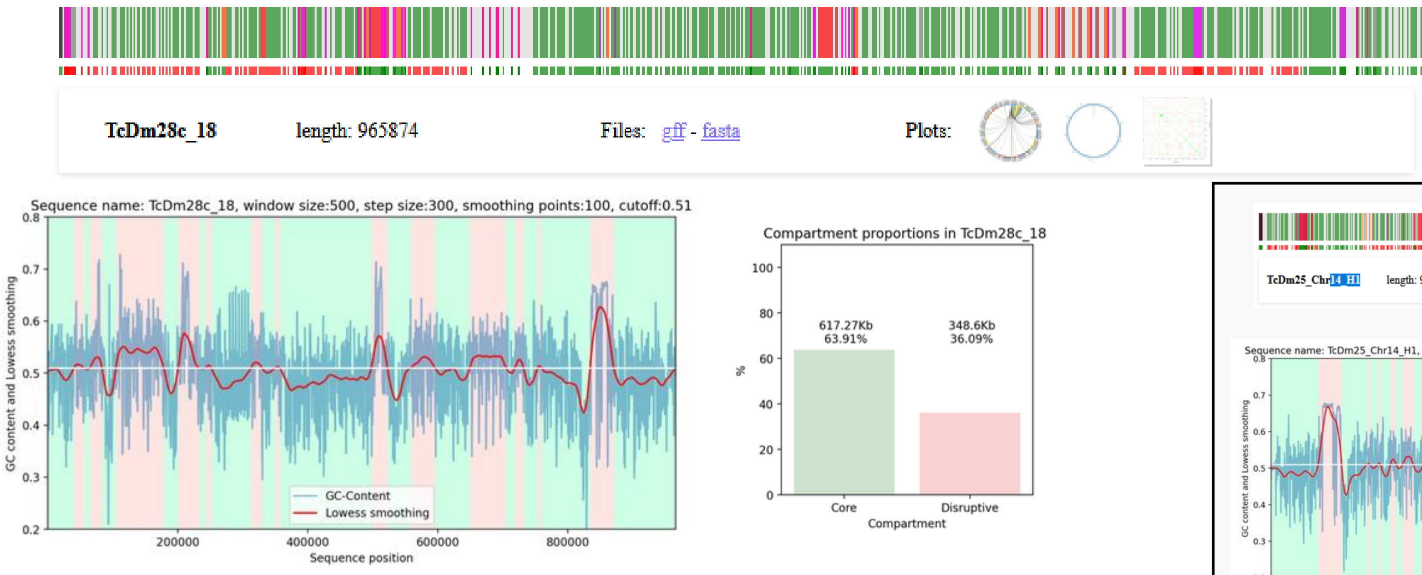

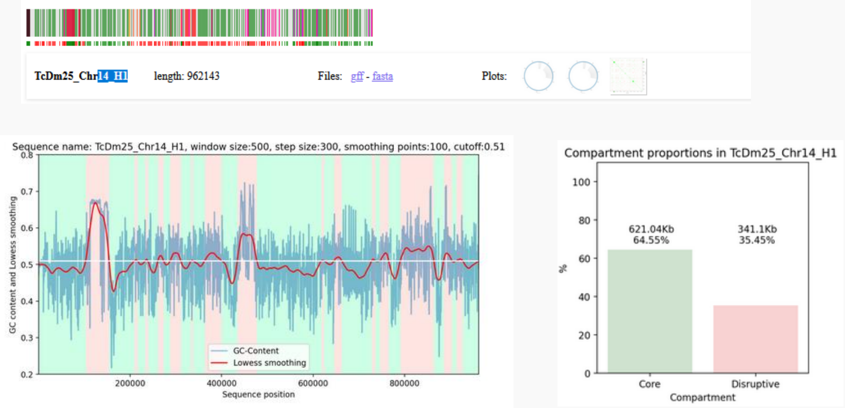

* Reverse Complement

Dm25

Chromosome 19

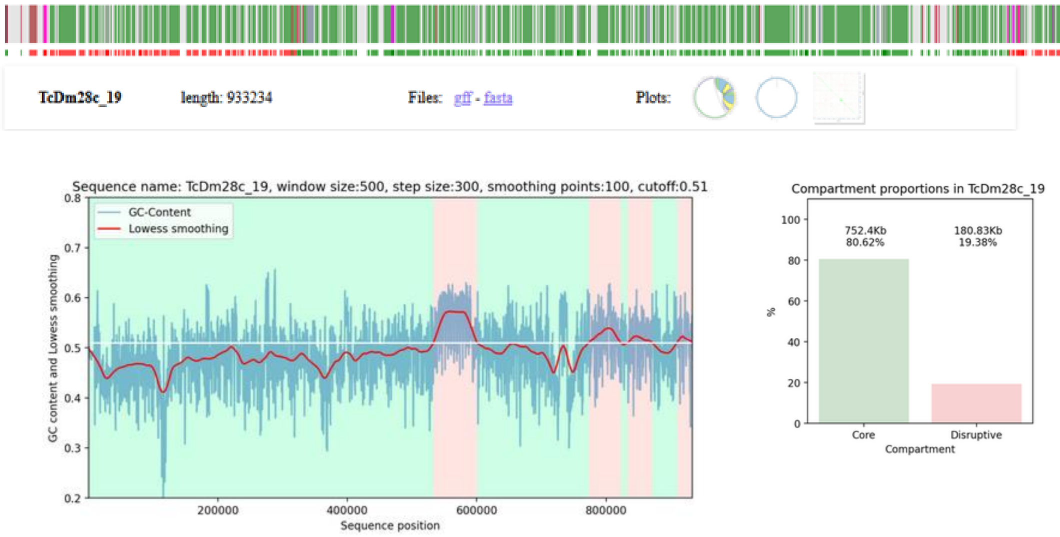

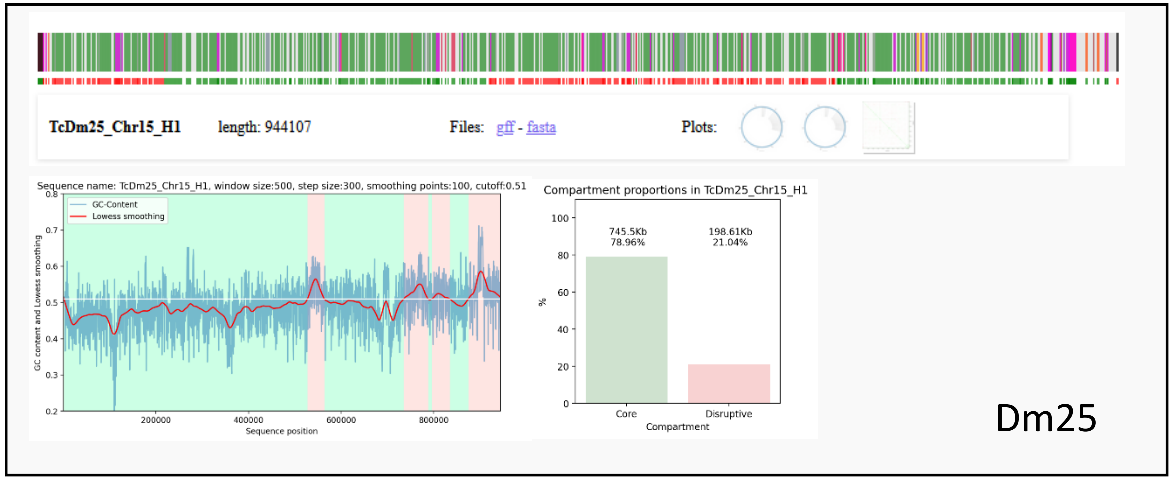

Chromosome 20

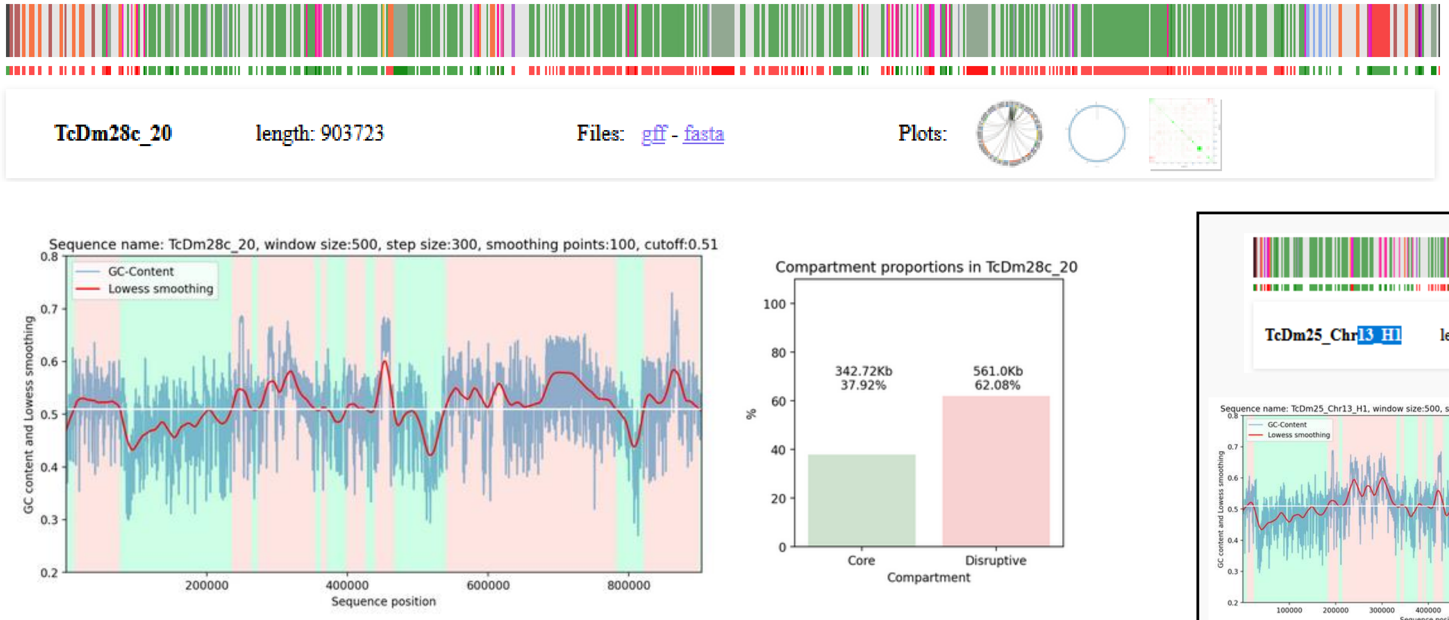

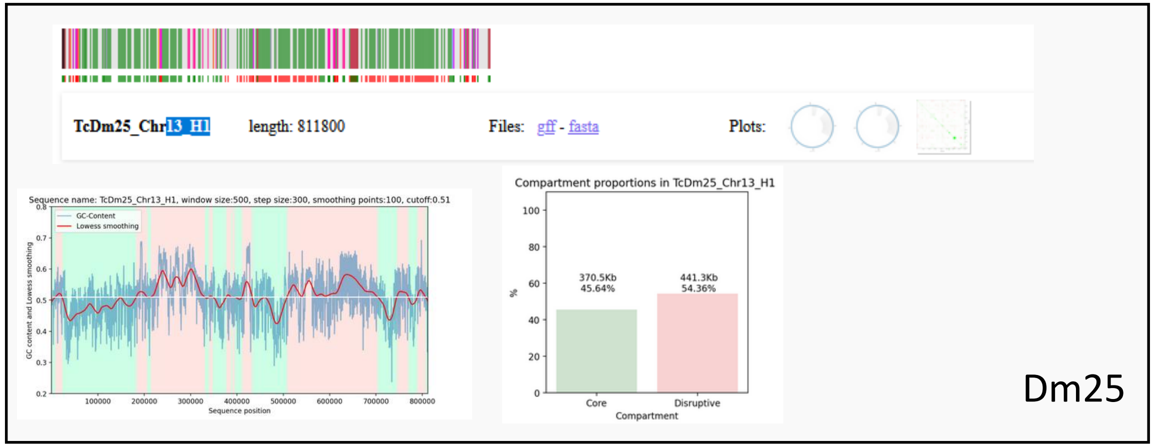

Chromosome 21

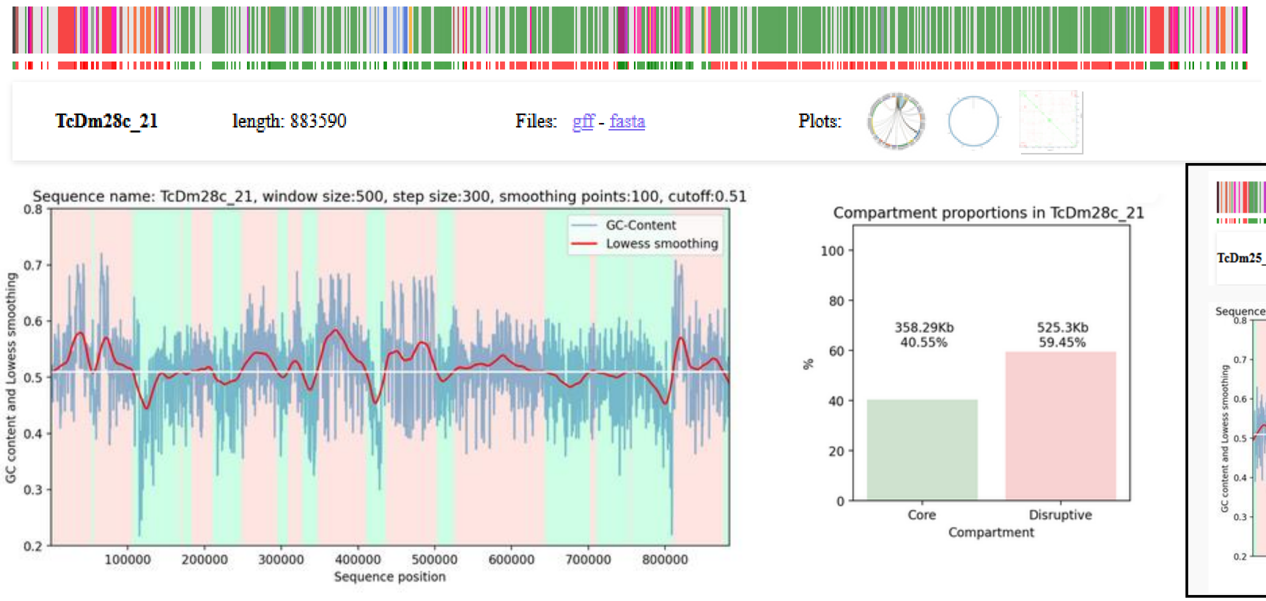

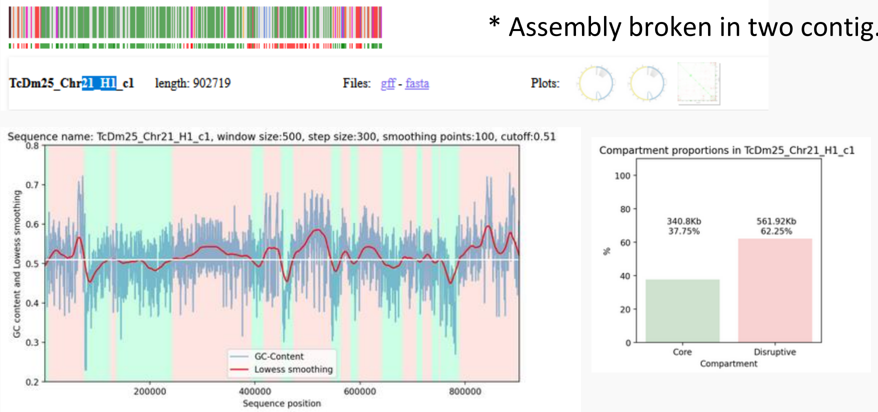

* Assembly broken in two contig. Contig 1

* Reverse Complement

Dm25

Chromosome 22

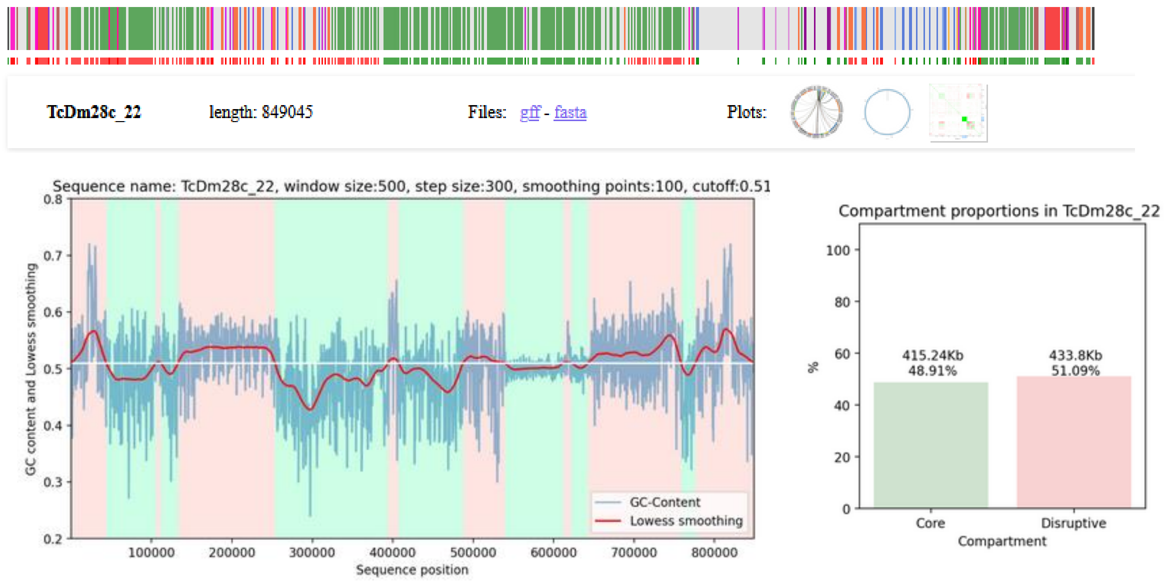

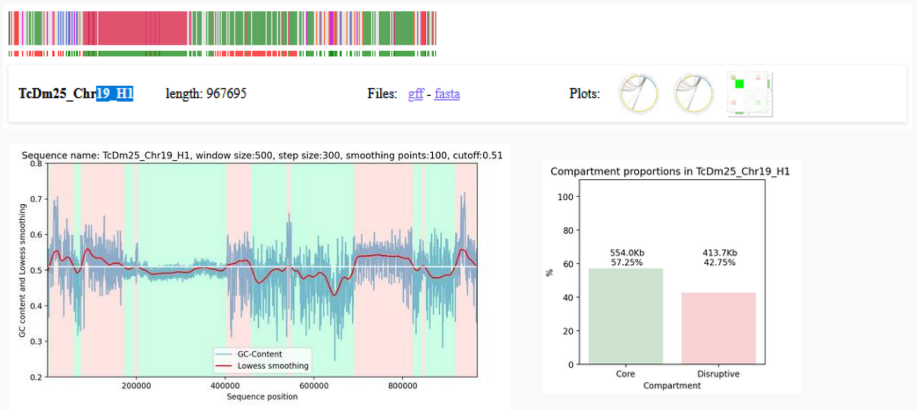

* Reverse Complement

Dm25

Chromosome 23

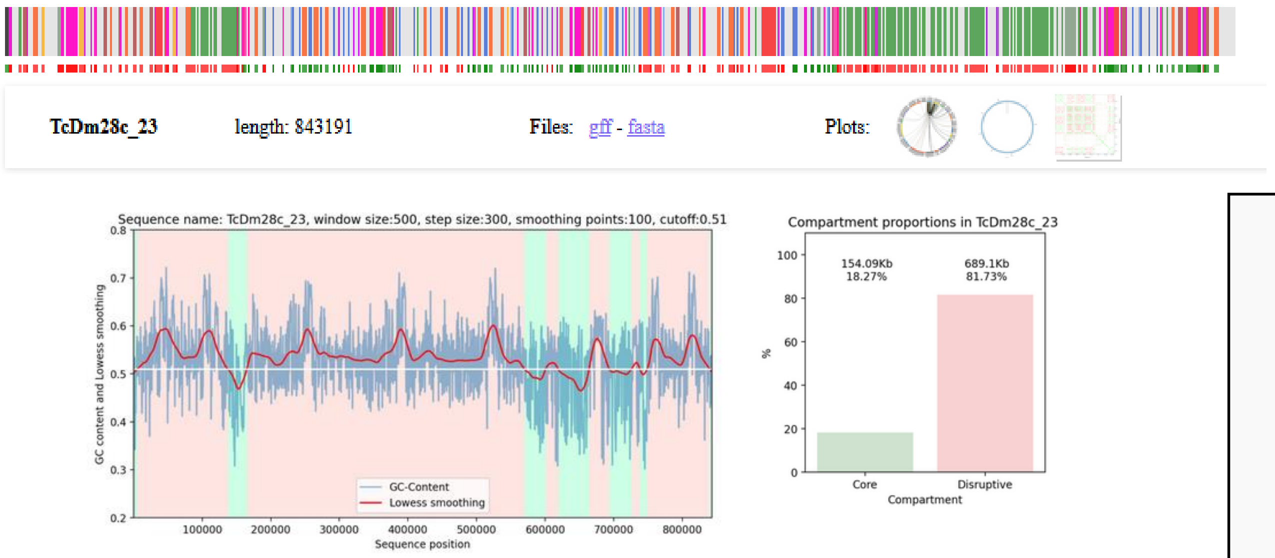

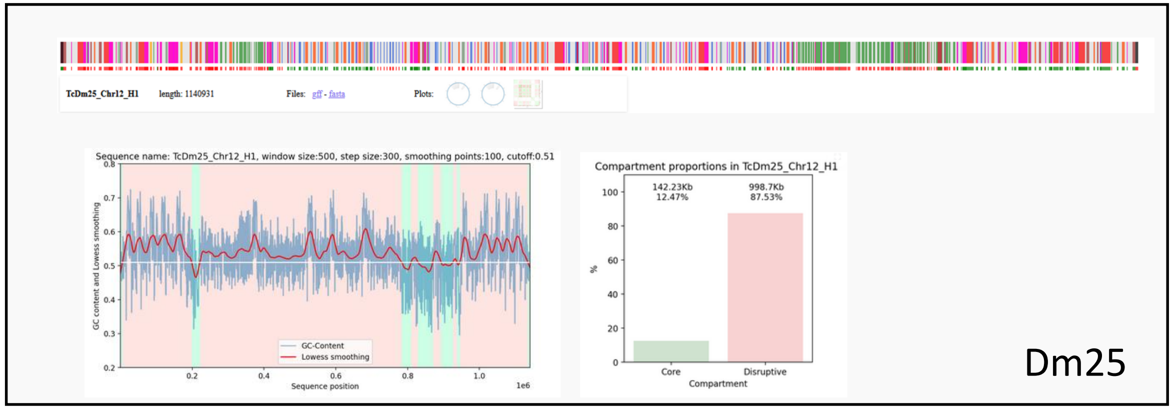

Chromosome 24

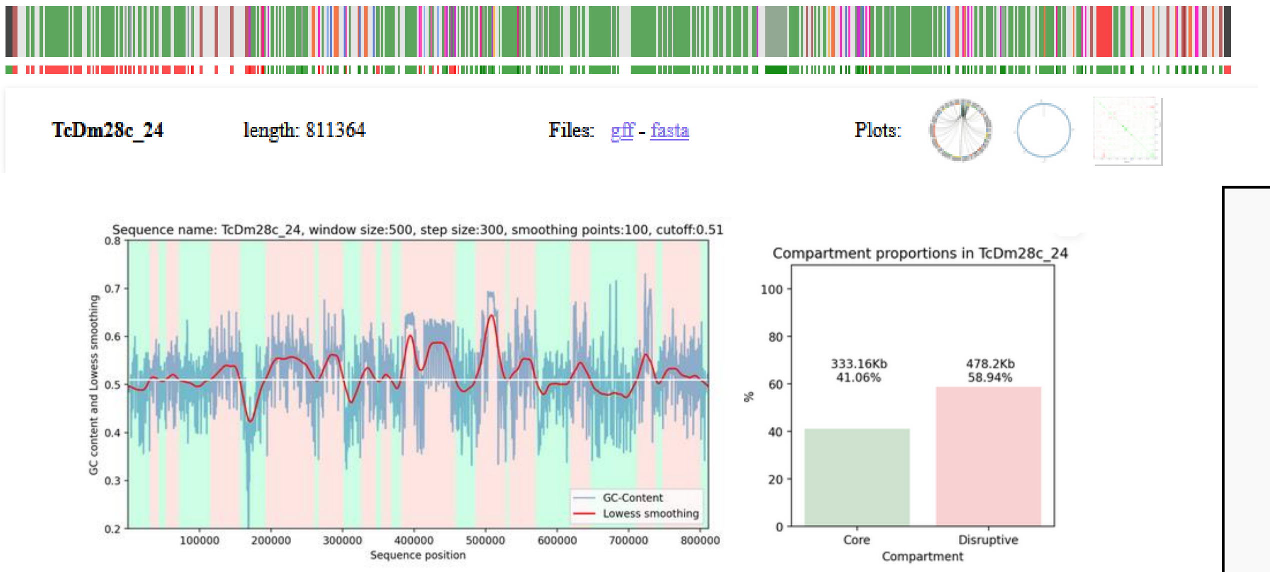

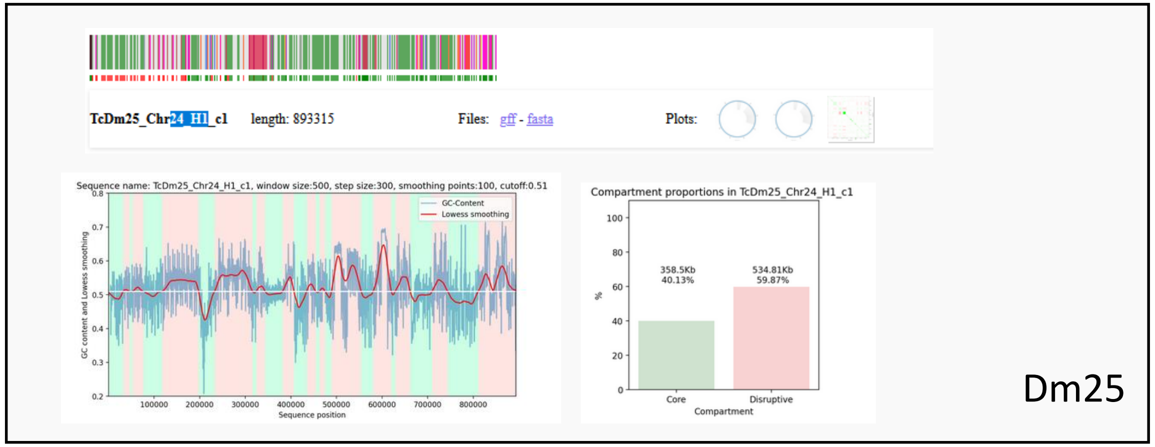

Chromosome 25

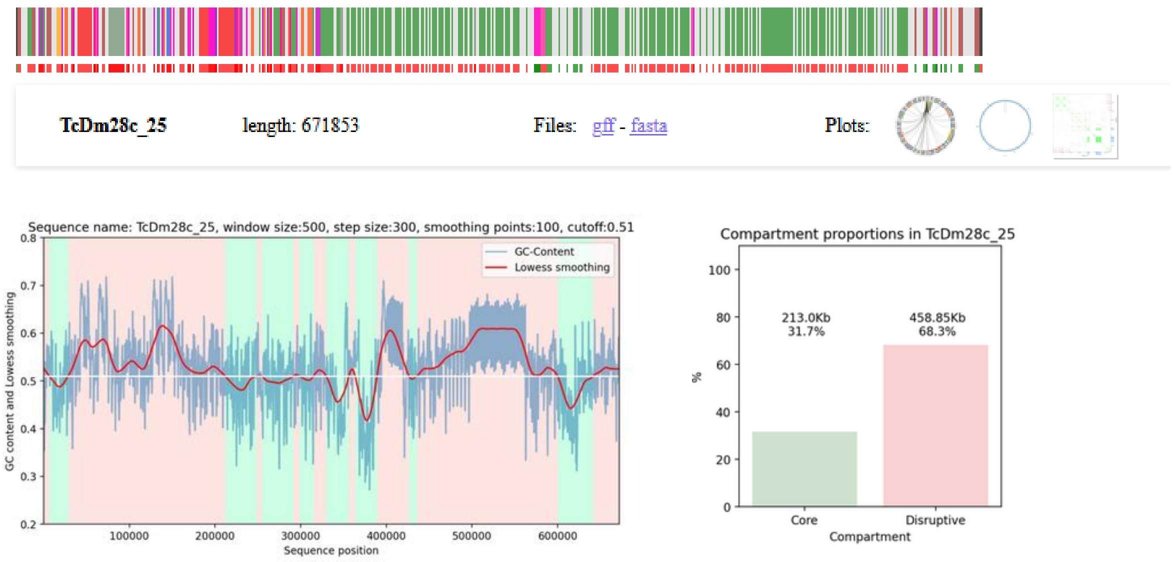

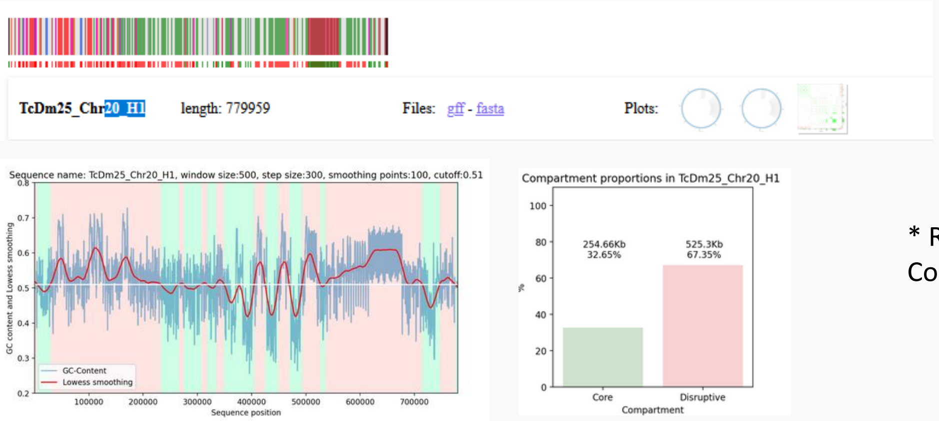

* Reverse Complement

Dm25

Chromosome 26

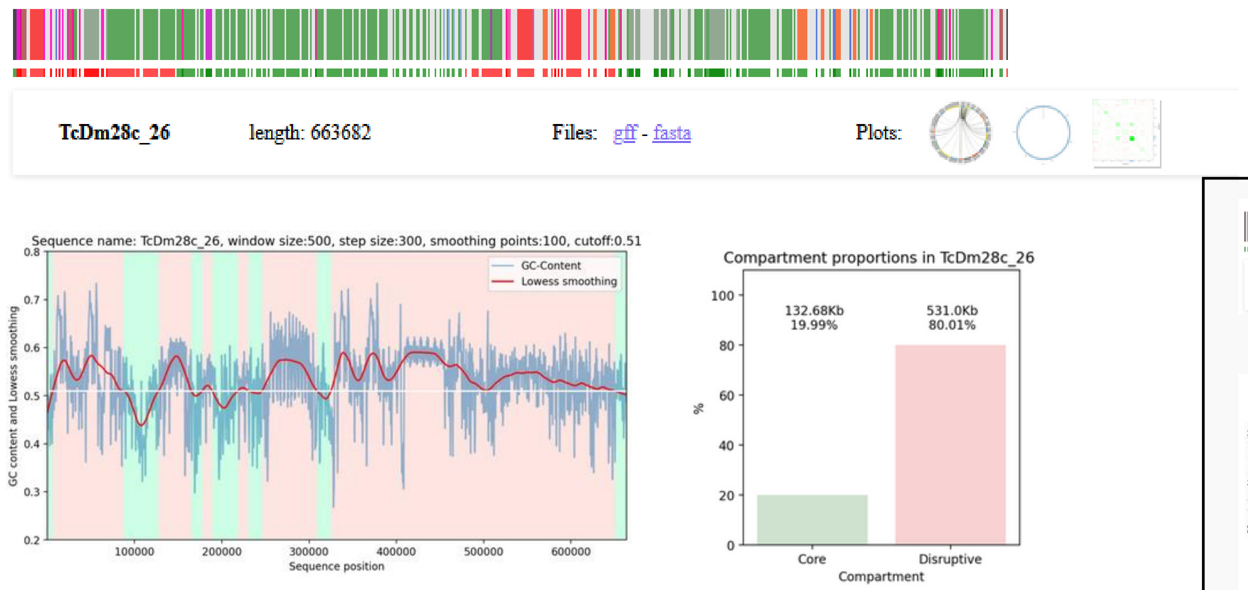

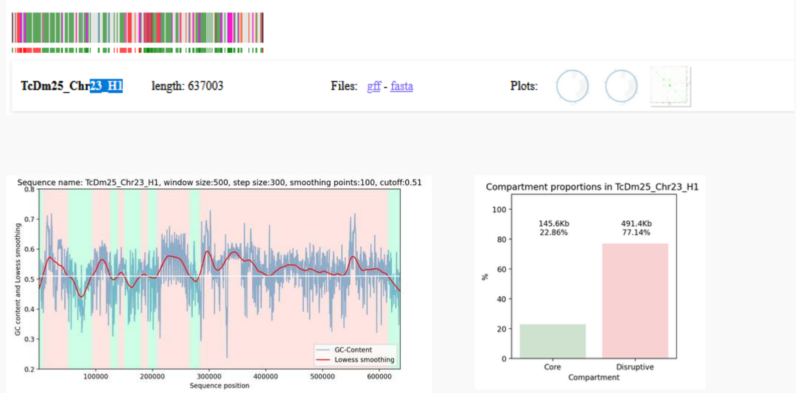

Dm25

Chromosome 27

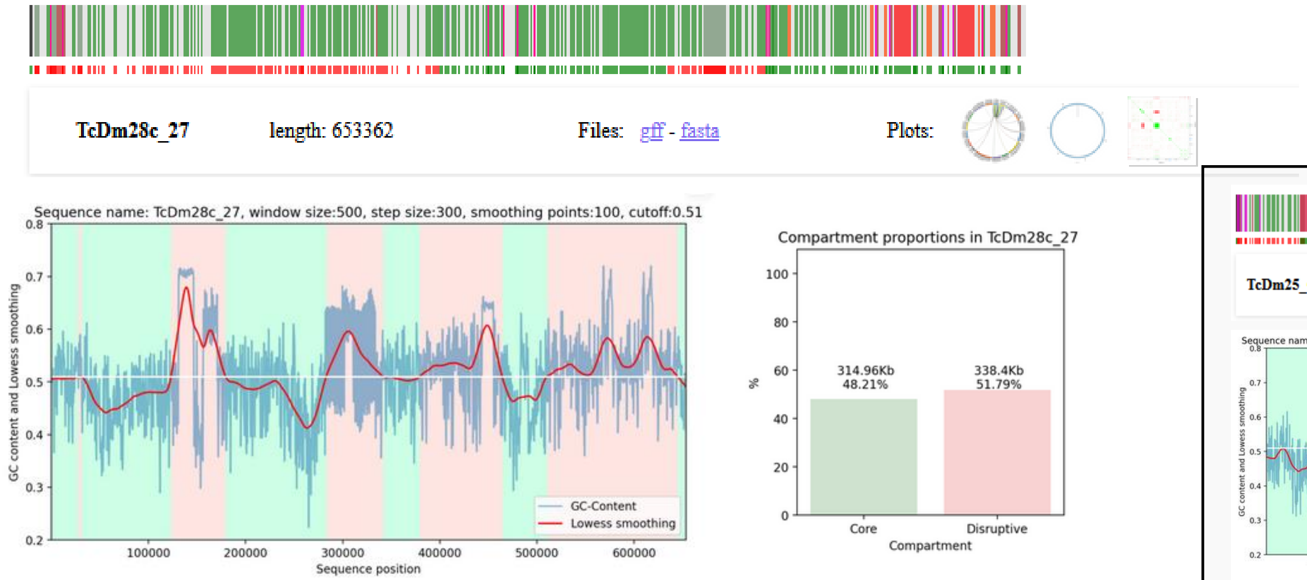

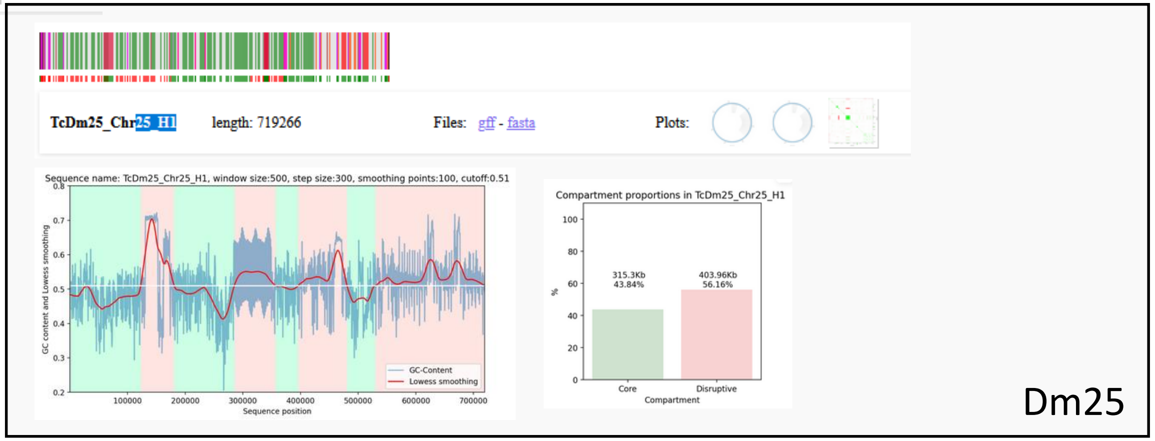

Chromosome 28

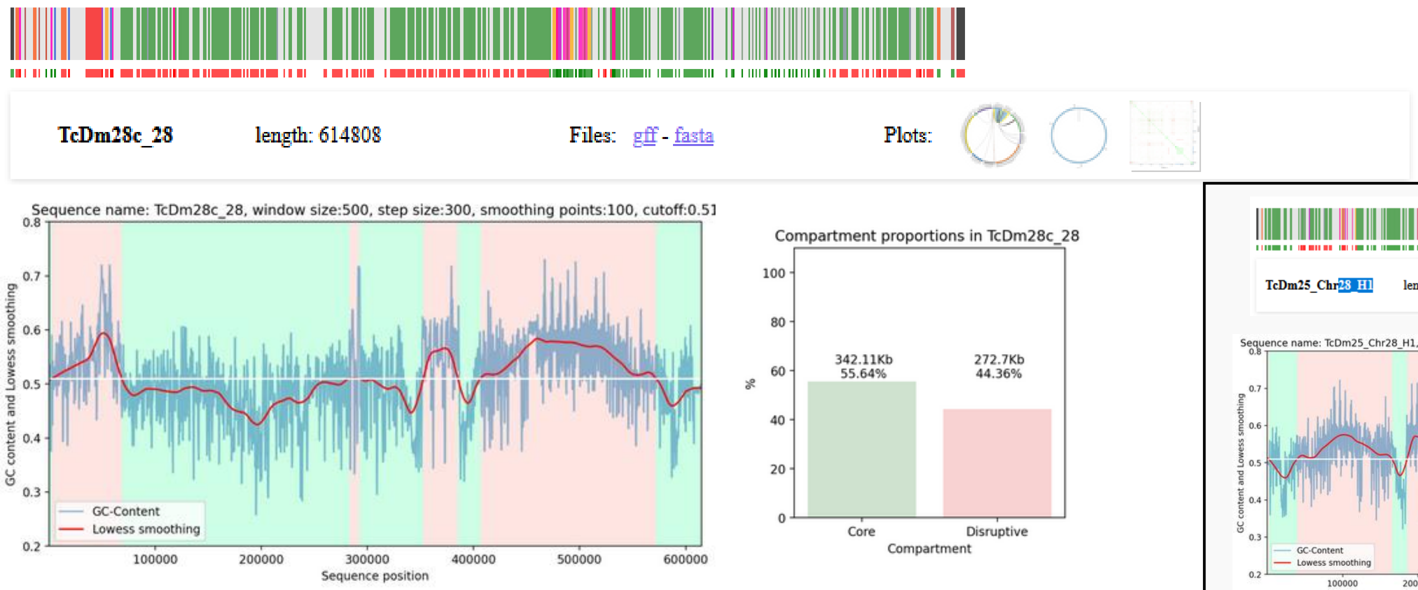

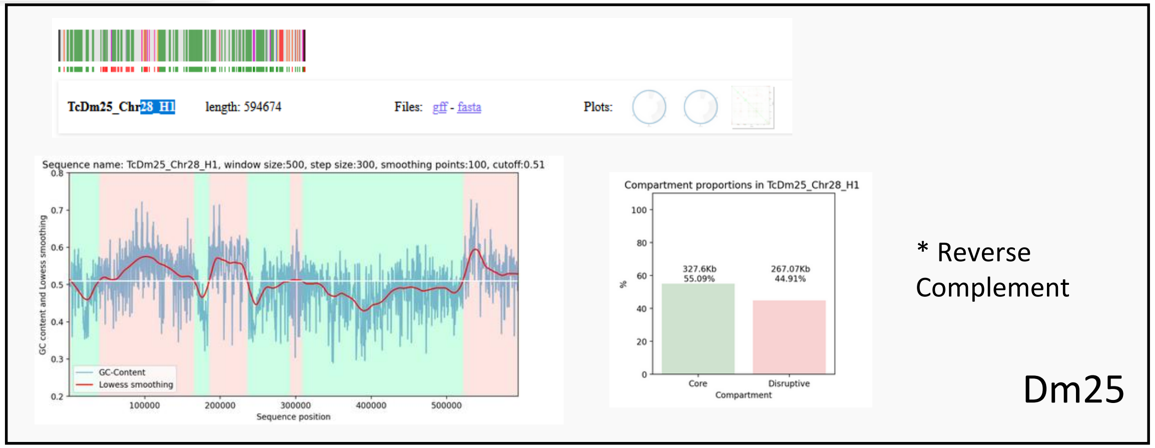

Chromosome 29

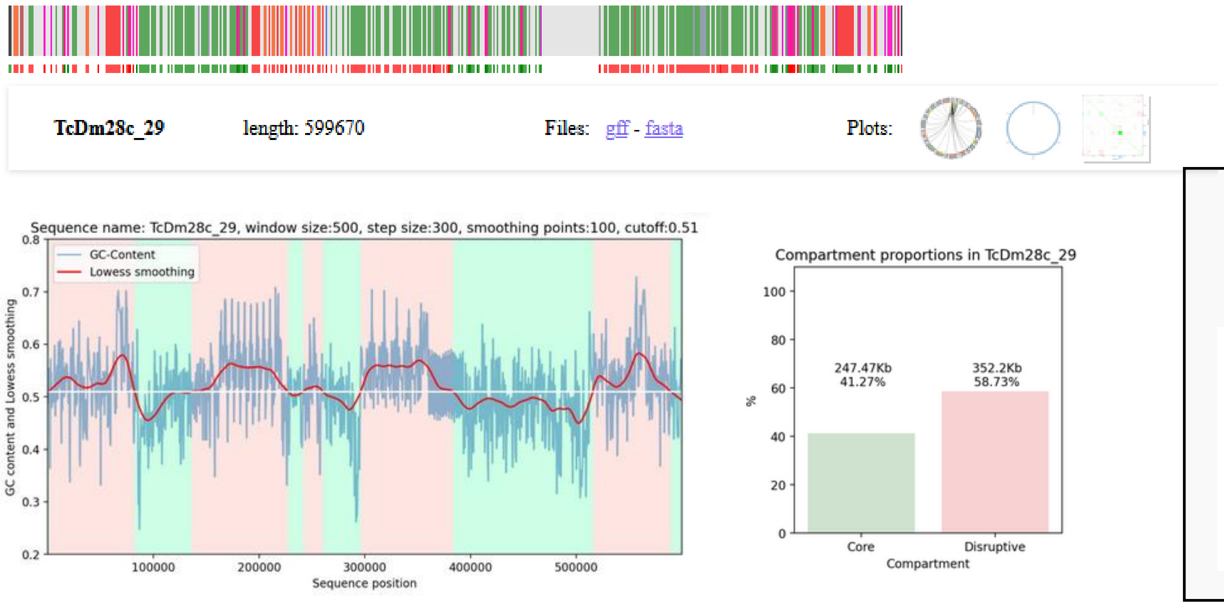

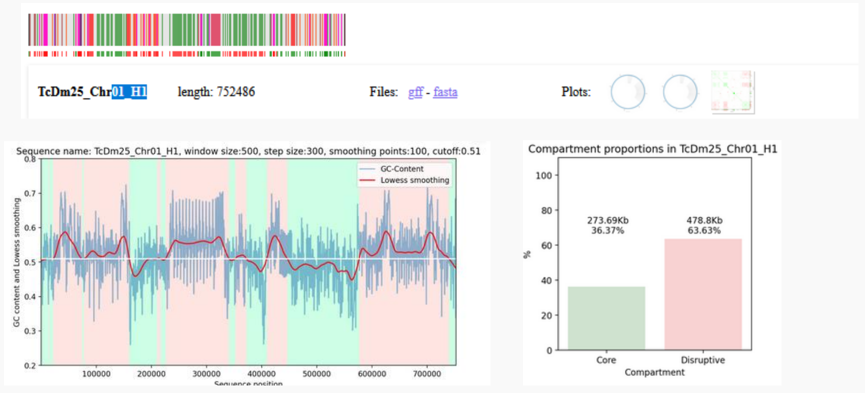

Dm25

Chromosome 30

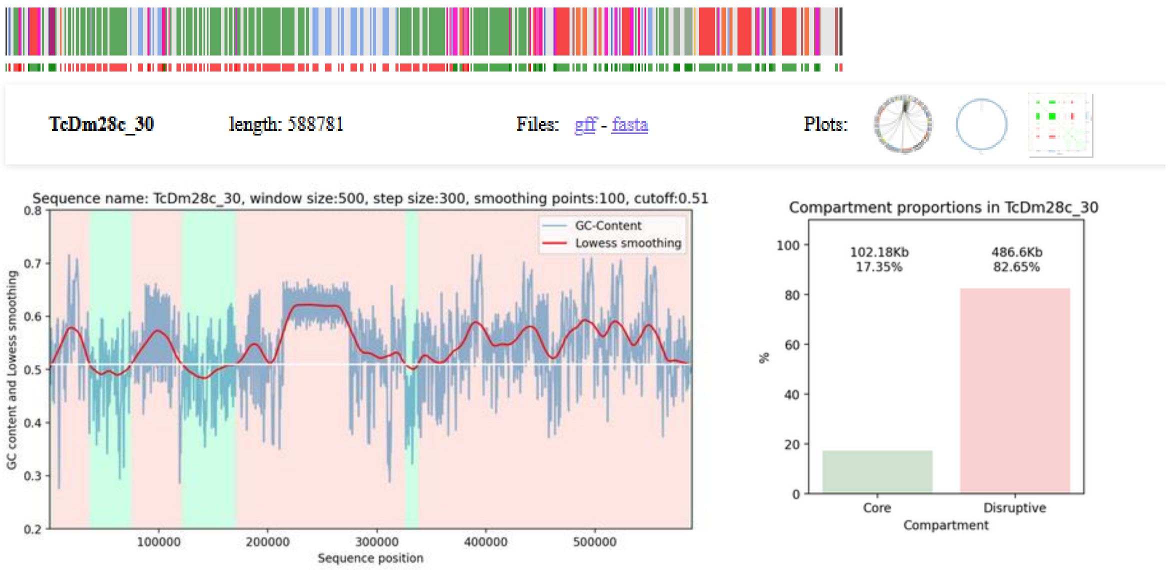

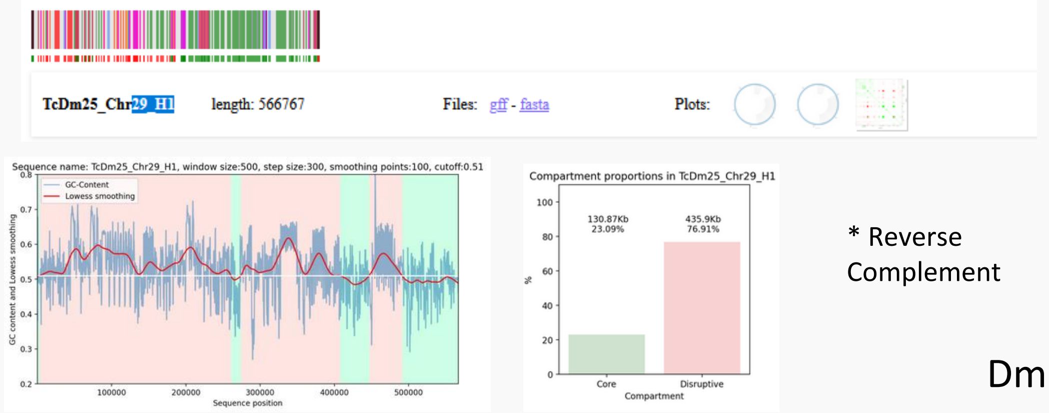

* Reverse Complement

Dm25

Chromosome 31

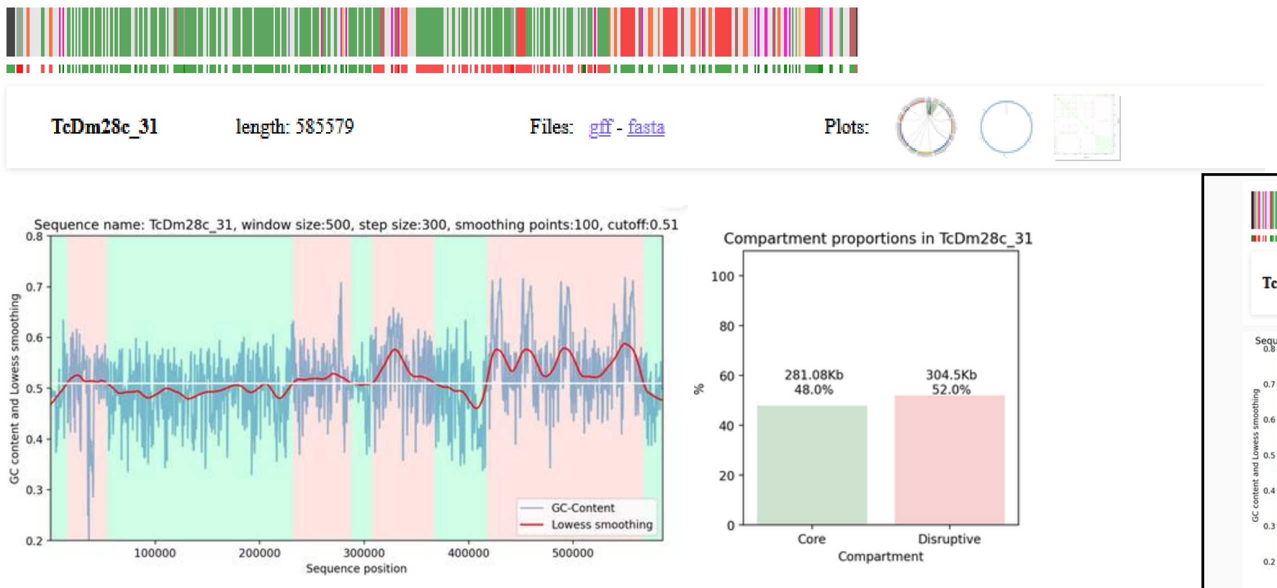

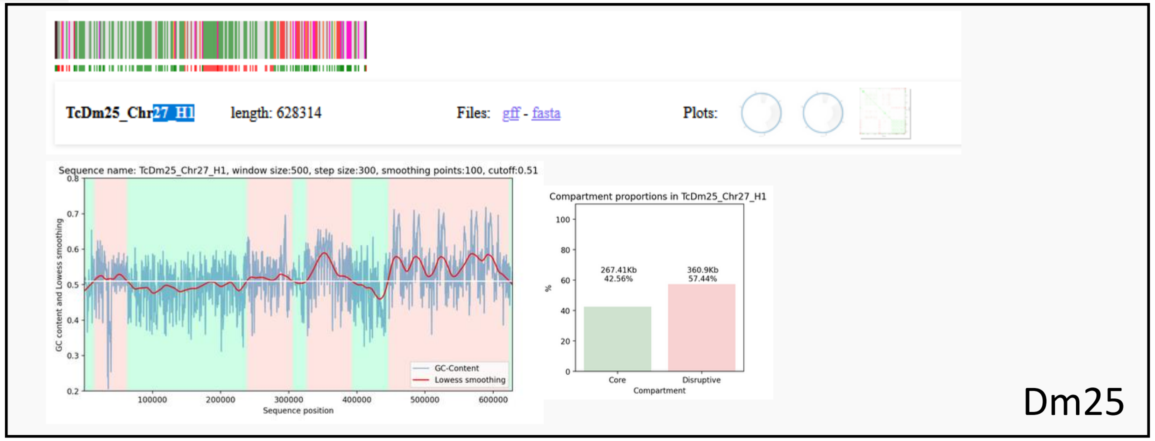

Chromosome 32

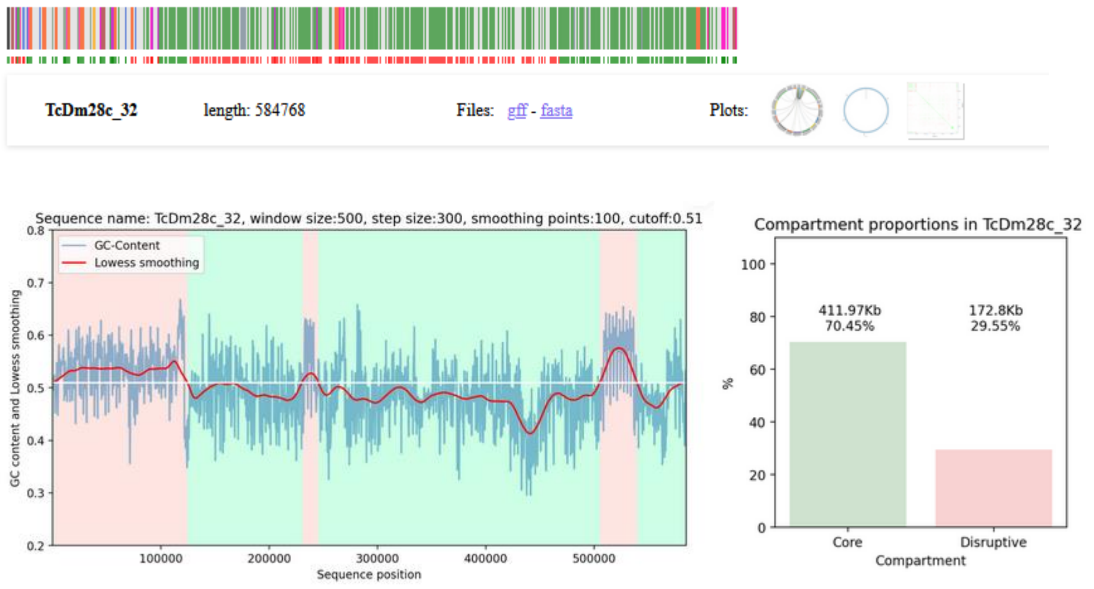

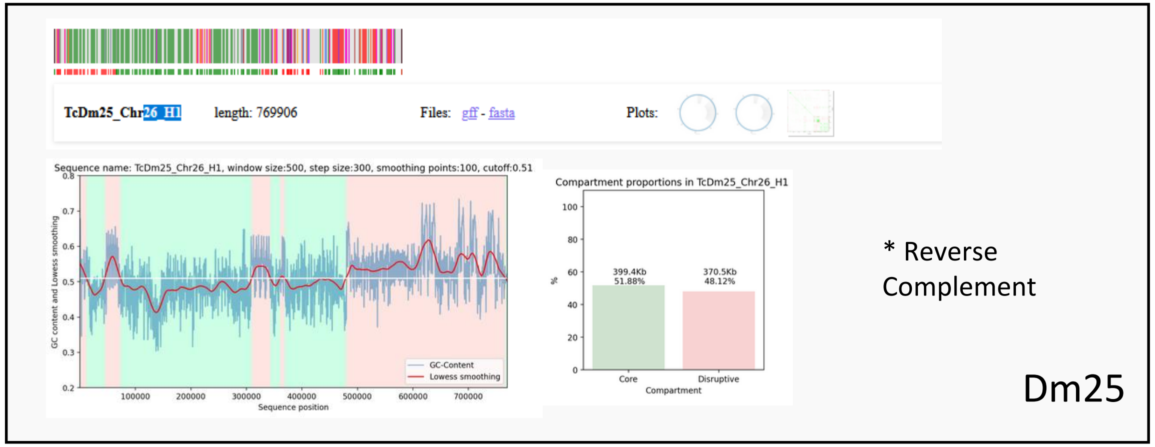

Supplement: Supplementary file 11 — Supplementary Material 11. [file 12864_2025_12482_MOESM11_ESM.pdf]
