# Supplementary material for: The complete genome of Trypanosoma cruzi reveals 32 chromosomes and three genomic compartments
Source: BMC Genomics. 2026 Jan 8;27:159. doi: 10.1186/s12864-025-12482-0 (PMC12879350; doi:10.1186/s12864-025-12482-0)
Supplement: Supplementary file 12 — Supplementary Material 12. [file 12864_2025_12482_MOESM12_ESM.pdf]

Supplementary Figure 6. Circos plot obtained with Symap 5.0, comparing Dm28c and Dm25 strain.

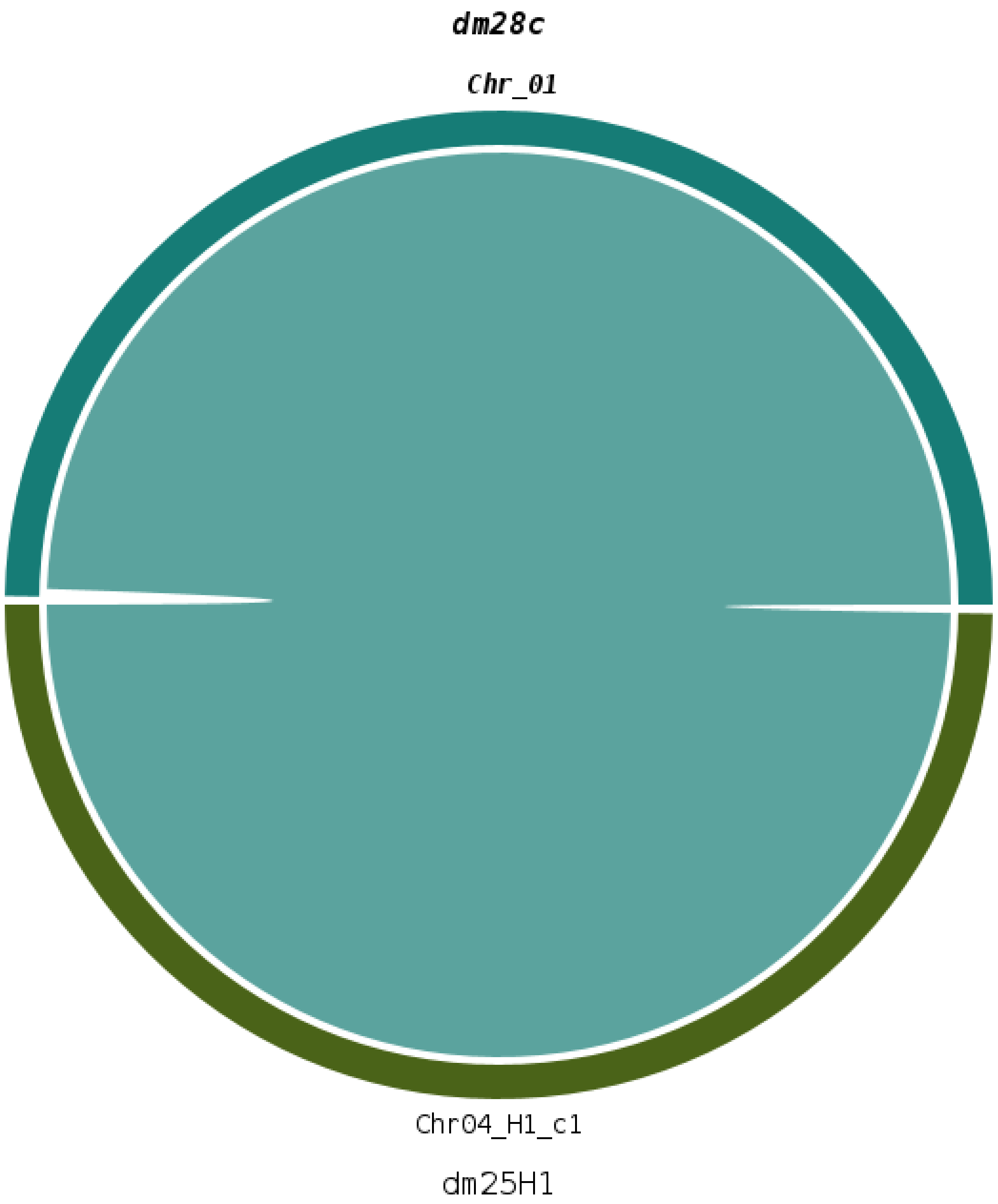
dm28c
Chr_01
Chr04_H1_c1
dm25H1

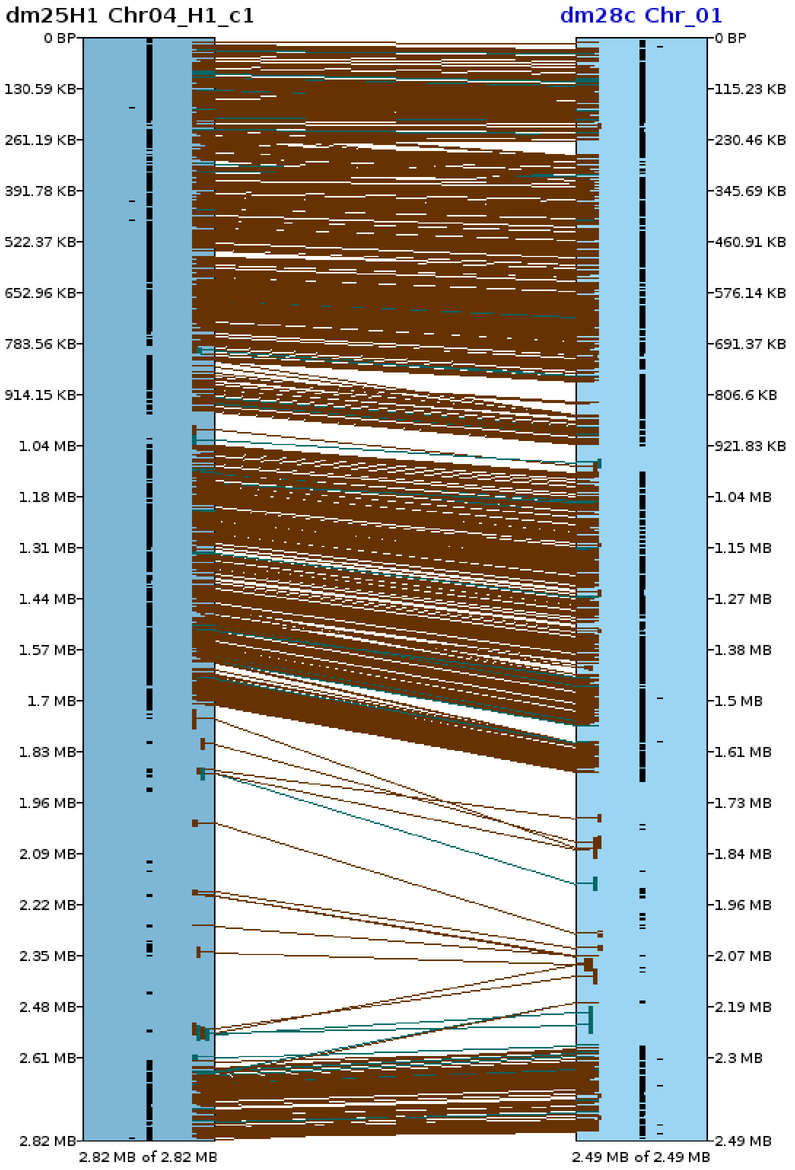
dm25H1 Chr04_H1_c1
dm28c Chr_01
0 BP
130.59 KB
261.19 KB
391.78 KB
522.37 KB
652.96 KB
783.56 KB
914.15 KB
1.04 MB
1.18 MB
1.31 MB
1.44 MB
1.57 MB
1.7 MB
1.83 MB
1.96 MB
2.09 MB
2.22 MB
2.35 MB
2.48 MB
2.61 MB
2.82 MB
0 BP
115.23 KB
230.46 KB
345.69 KB
460.91 KB
576.14 KB
691.37 KB
806.6 KB
921.83 KB
1.04 MB
1.15 MB
1.27 MB
1.38 MB
1.5 MB
1.61 MB
1.73 MB
1.84 MB
1.96 MB
2.07 MB
2.19 MB
2.3 MB
2.49 MB
2.82 MB of 2.82 MB
2.49 MB of 2.49 MB

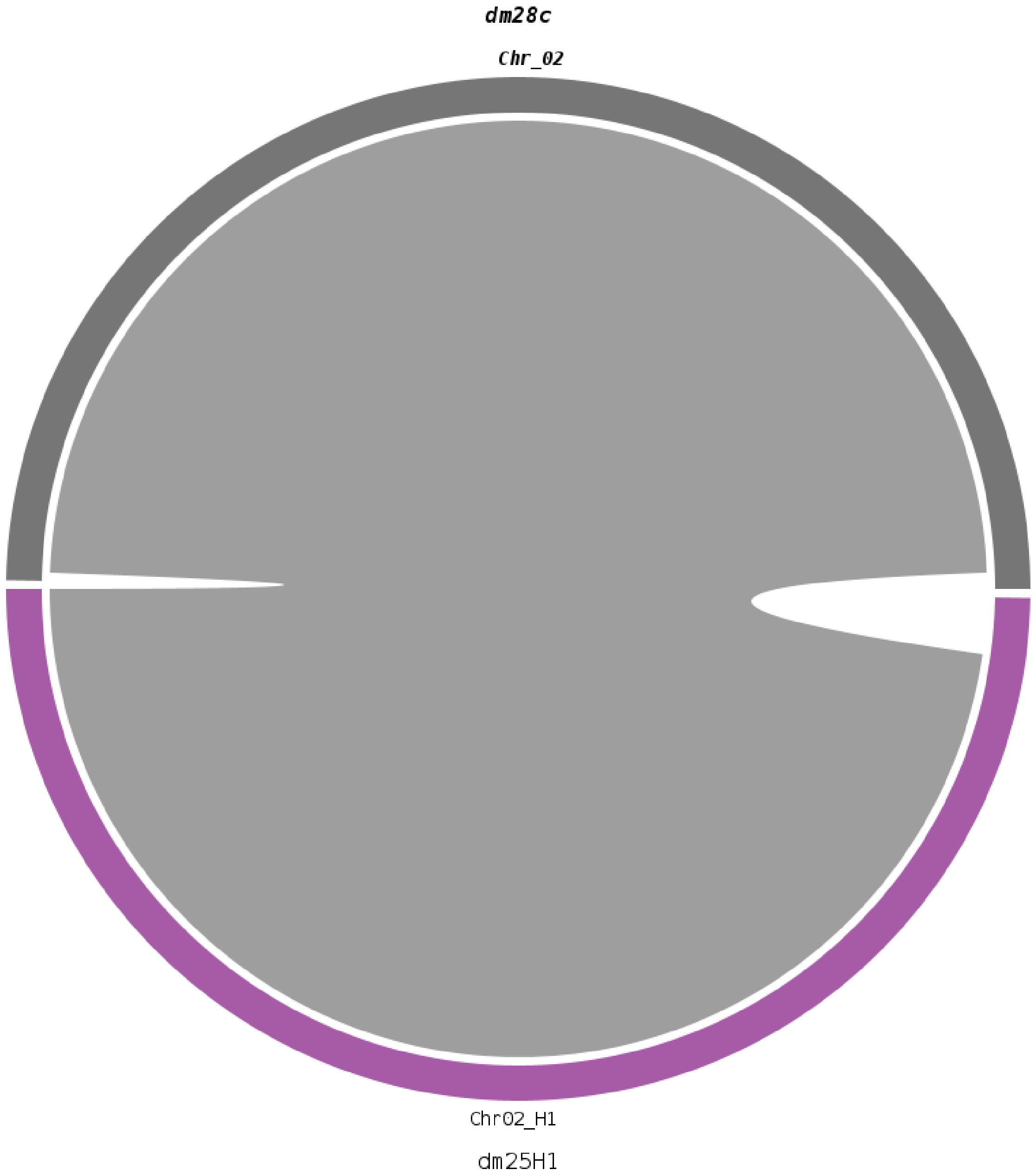
dm28c
Chr_02
Chr02_H1
dm25H1

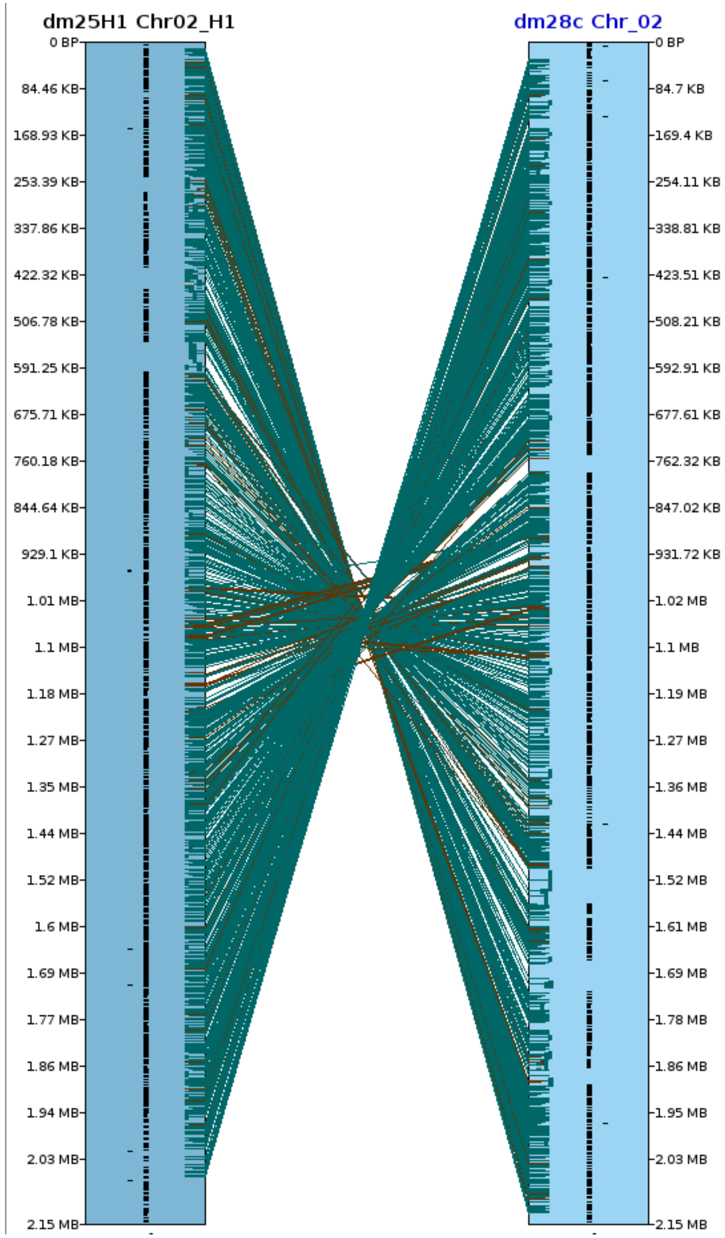
dm25H1 Chr02_H1
dm28c Chr_02
0 BP
84.46 KB
168.93 KB
253.39 KB
337.86 KB
422.32 KB
506.78 KB
591.25 KB
675.71 KB
760.18 KB
844.64 KB
929.1 KB
1.01 MB
1.1 MB
1.18 MB
1.27 MB
1.35 MB
1.44 MB
1.52 MB
1.6 MB
1.69 MB
1.77 MB
1.86 MB
1.94 MB
2.03 MB
2.15 MB
0 BP
84.7 KB
169.4 KB
254.11 KB
338.81 KB
423.51 KB
508.21 KB
592.91 KB
677.61 KB
762.32 KB
847.02 KB
931.72 KB
1.02 MB
1.1 MB
1.19 MB
1.27 MB
1.36 MB
1.44 MB
1.52 MB
1.61 MB
1.69 MB
1.78 MB
1.86 MB
1.95 MB
2.03 MB
2.15 MB

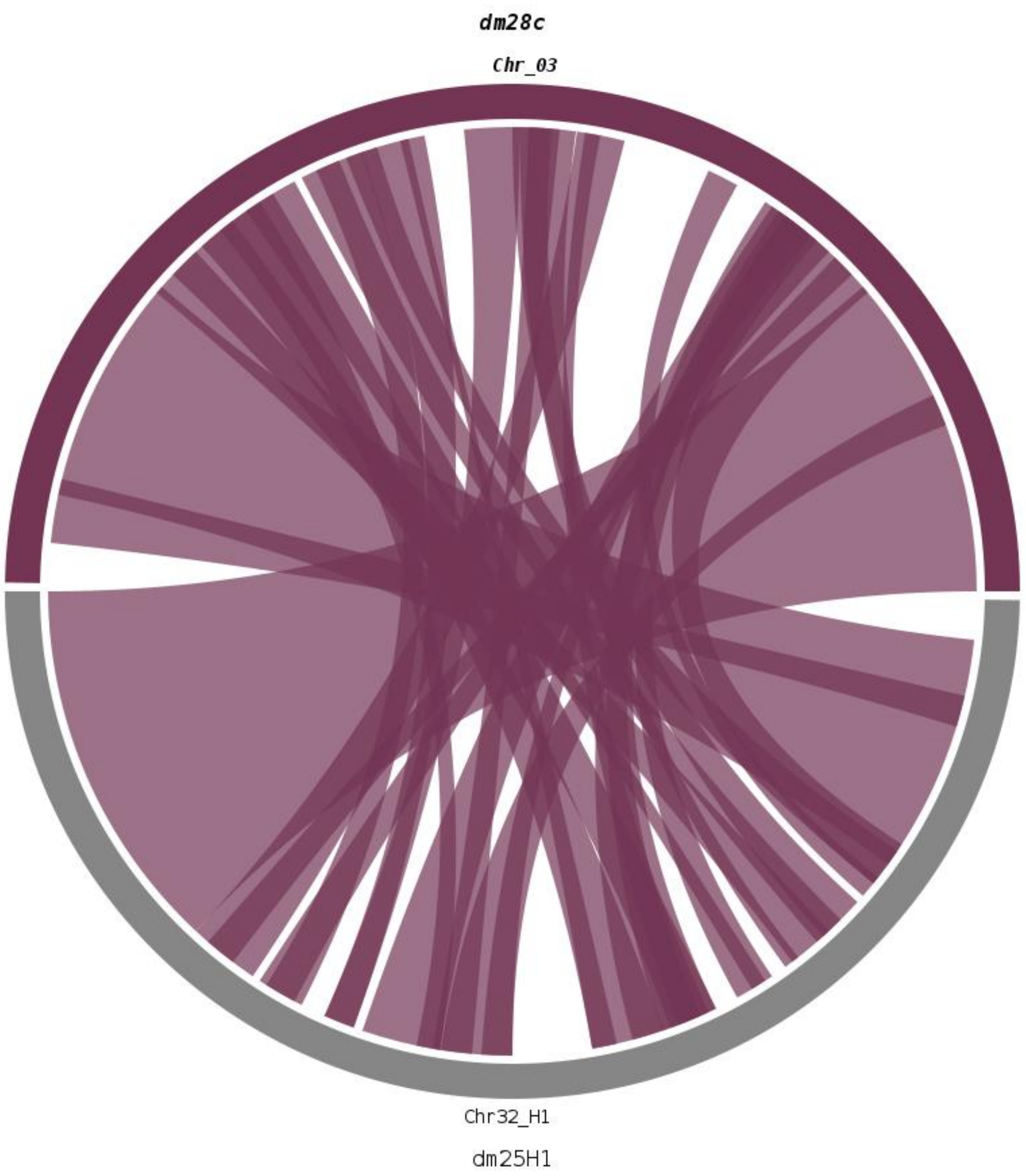
dm28c
Chr_03
Chr32_H1
dm25H1

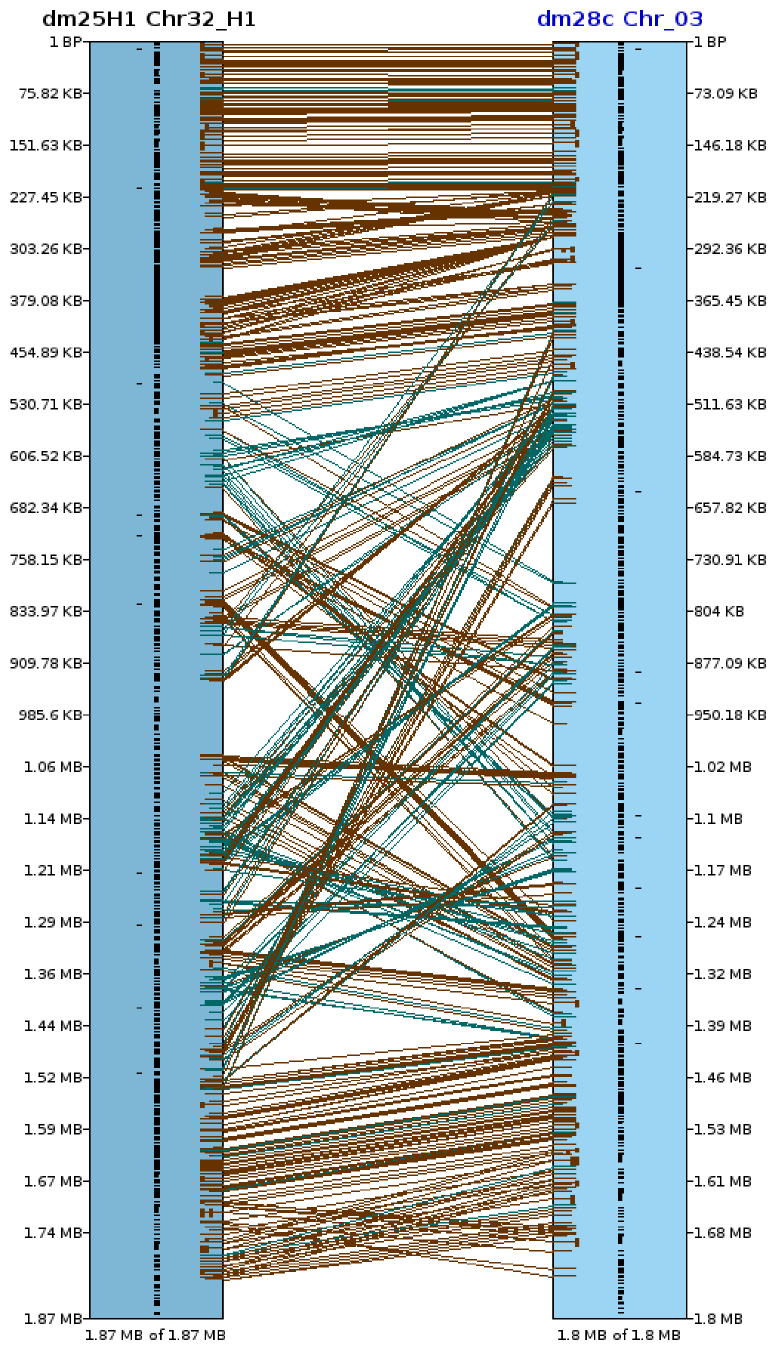
dm25H1 Chr32_H1
dm28c Chr_03
1 BP
75.82 KB
151.63 KB
227.45 KB
303.26 KB
379.08 KB
454.89 KB
530.71 KB
606.52 KB
682.34 KB
758.15 KB
833.97 KB
909.78 KB
985.6 KB
1.06 MB
1.14 MB
1.21 MB
1.29 MB
1.36 MB
1.44 MB
1.52 MB
1.59 MB
1.67 MB
1.74 MB
1.87 MB
1.87 MB of 1.87 MB
1 BP
73.09 KB
146.18 KB
219.27 KB
292.36 KB
365.45 KB
438.54 KB
511.63 KB
584.73 KB
657.82 KB
730.91 KB
804 KB
877.09 KB
950.18 KB
1.02 MB
1.1 MB
1.17 MB
1.24 MB
1.32 MB
1.39 MB
1.46 MB
1.53 MB
1.61 MB
1.68 MB
1.8 MB
1.8 MB of 1.8 MB

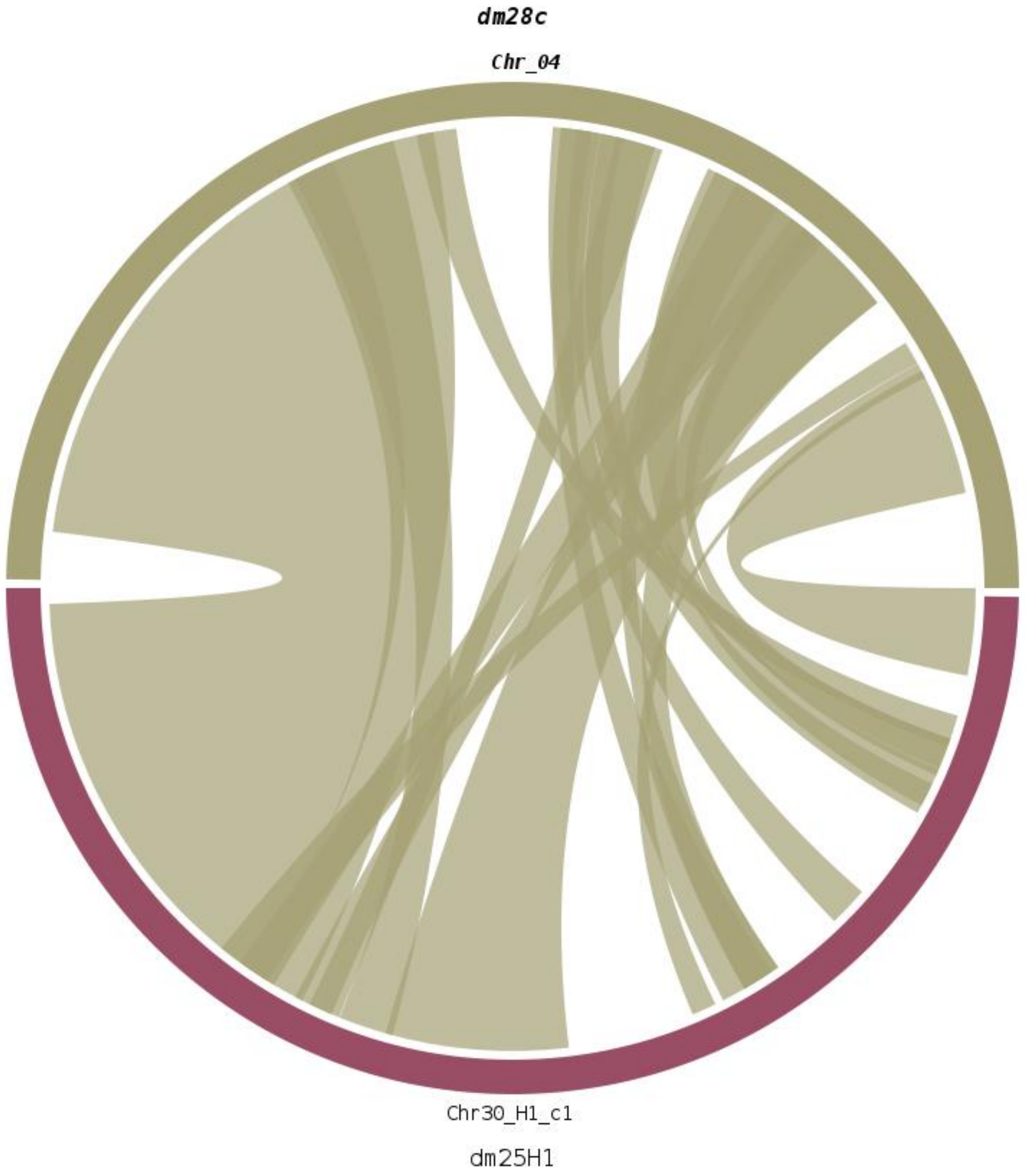
dm28c
Chr_04
Chr30_H1_c1
dm25H1

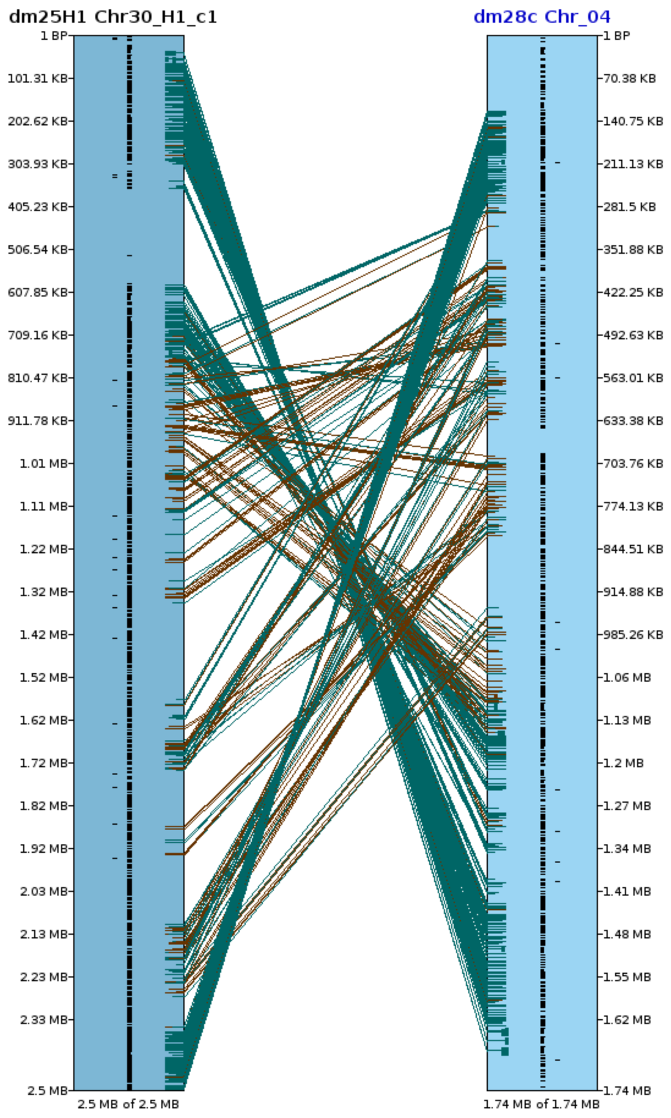
dm25H1 Chr30_H1_c1
dm28c Chr_04
1 BP
101.31 KB
202.62 KB
303.93 KB
405.23 KB
506.54 KB
607.85 KB
709.16 KB
810.47 KB
911.78 KB
1.01 MB
1.11 MB
1.22 MB
1.32 MB
1.42 MB
1.52 MB
1.62 MB
1.72 MB
1.82 MB
1.92 MB
2.03 MB
2.13 MB
2.23 MB
2.33 MB
2.5 MB
2.5 MB of 2.5 MB
1 BP
70.38 KB
140.75 KB
211.13 KB
281.5 KB
351.88 KB
422.25 KB
492.63 KB
563.01 KB
633.38 KB
703.76 KB
774.13 KB
844.51 KB
914.88 KB
985.26 KB
1.06 MB
1.13 MB
1.2 MB
1.27 MB
1.34 MB
1.41 MB
1.48 MB
1.55 MB
1.62 MB
1.74 MB
1.74 MB of 1.74 MB

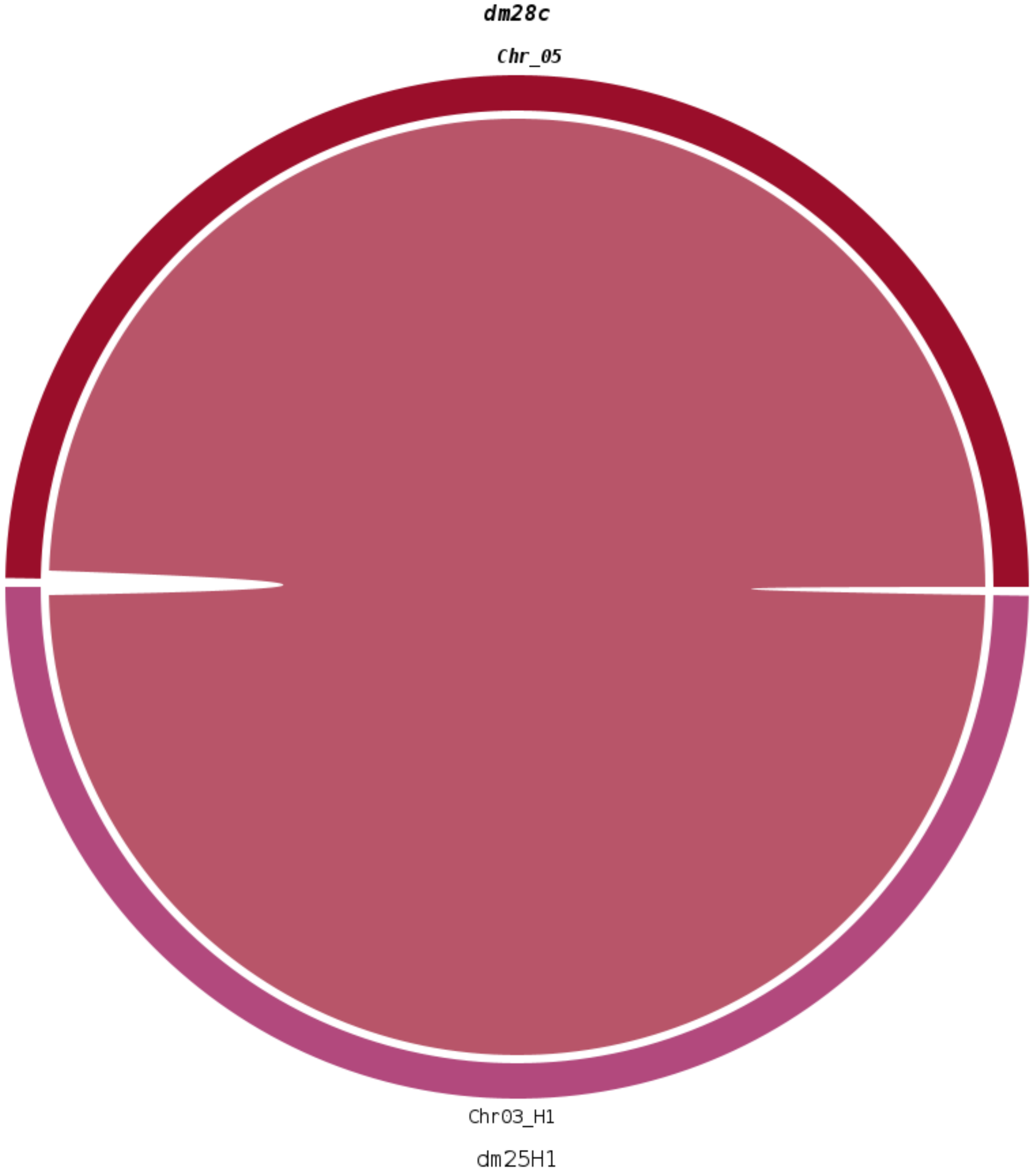
dm28c
Chr_05
Chr03_H1
dm25H1

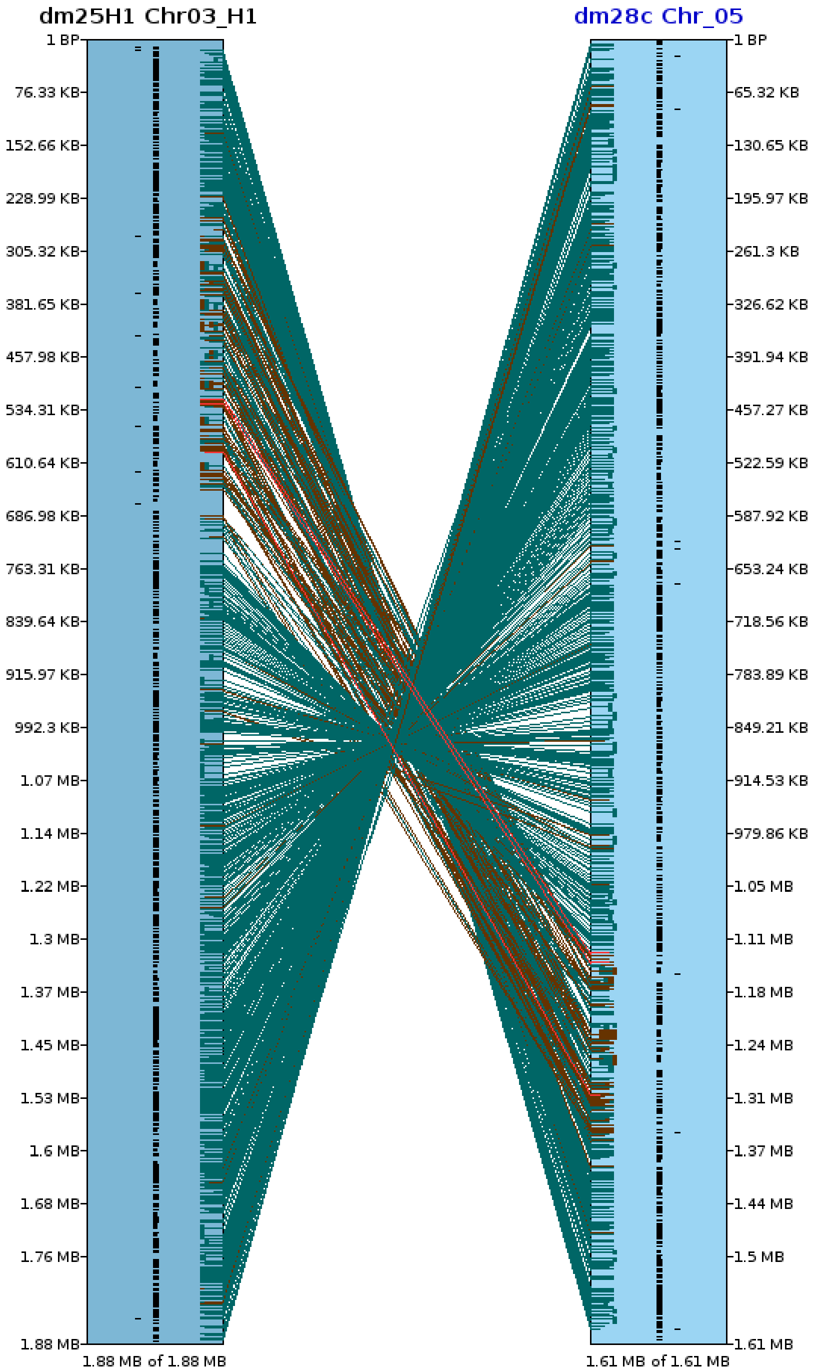
dm25H1 Chr03_H1
dm28c Chr_05
1 BP
76.33 KB
152.66 KB
228.99 KB
305.32 KB
381.65 KB
457.98 KB
534.31 KB
610.64 KB
686.98 KB
763.31 KB
839.64 KB
915.97 KB
992.3 KB
1.07 MB
1.14 MB
1.22 MB
1.3 MB
1.37 MB
1.45 MB
1.53 MB
1.6 MB
1.68 MB
1.76 MB
1.88 MB
1.88 MB of 1.88 MB
1 BP
65.32 KB
130.65 KB
195.97 KB
261.3 KB
326.62 KB
391.94 KB
457.27 KB
522.59 KB
587.92 KB
653.24 KB
718.56 KB
783.89 KB
849.21 KB
914.53 KB
979.86 KB
1.05 MB
1.11 MB
1.18 MB
1.24 MB
1.31 MB
1.37 MB
1.44 MB
1.5 MB
1.61 MB
1.61 MB of 1.61 MB

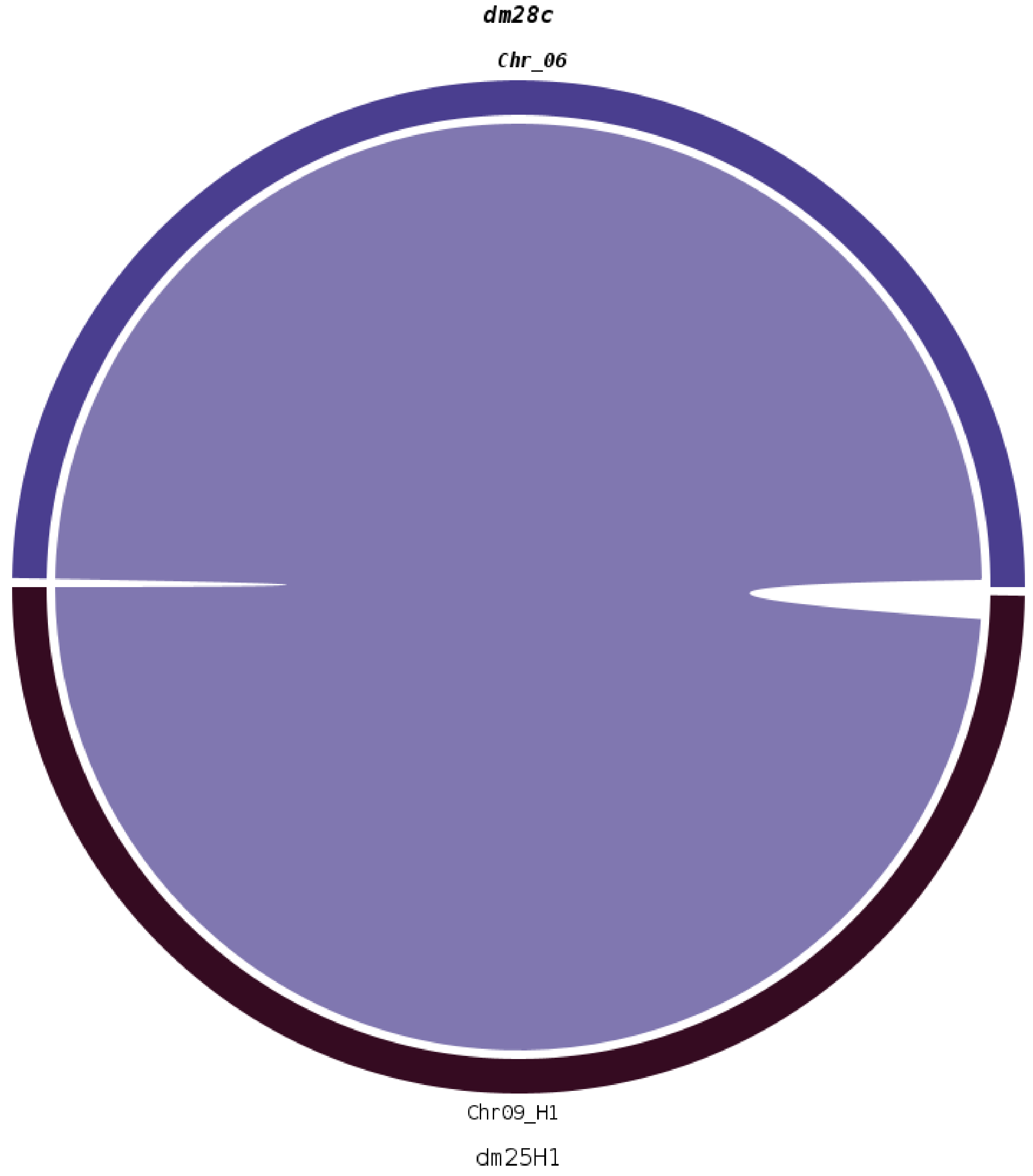
dm28c
Chr_06
Chr09_H1
dm25H1

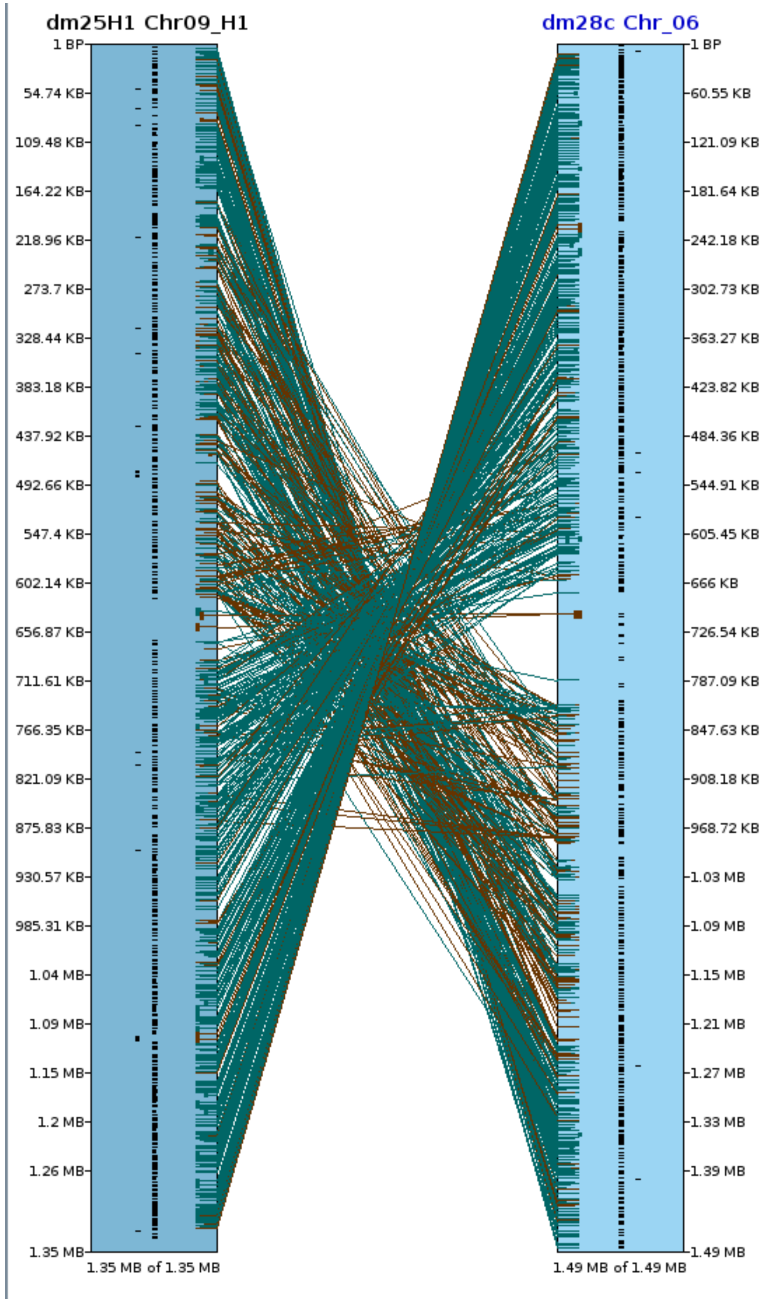
dm25H1 Chr09_H1
dm28c Chr_06
1 BP
54.74 KB
109.48 KB
164.22 KB
218.96 KB
273.7 KB
328.44 KB
383.18 KB
437.92 KB
492.66 KB
547.4 KB
602.14 KB
656.87 KB
711.61 KB
766.35 KB
821.09 KB
875.83 KB
930.57 KB
985.31 KB
1.04 MB
1.09 MB
1.15 MB
1.2 MB
1.26 MB
1.35 MB
1.35 MB of 1.35 MB
1 BP
60.55 KB
121.09 KB
181.64 KB
242.18 KB
302.73 KB
363.27 KB
423.82 KB
484.36 KB
544.91 KB
605.45 KB
666 KB
726.54 KB
787.09 KB
847.63 KB
908.18 KB
968.72 KB
1.03 MB
1.09 MB
1.15 MB
1.21 MB
1.27 MB
1.33 MB
1.39 MB
1.49 MB
1.49 MB of 1.49 MB

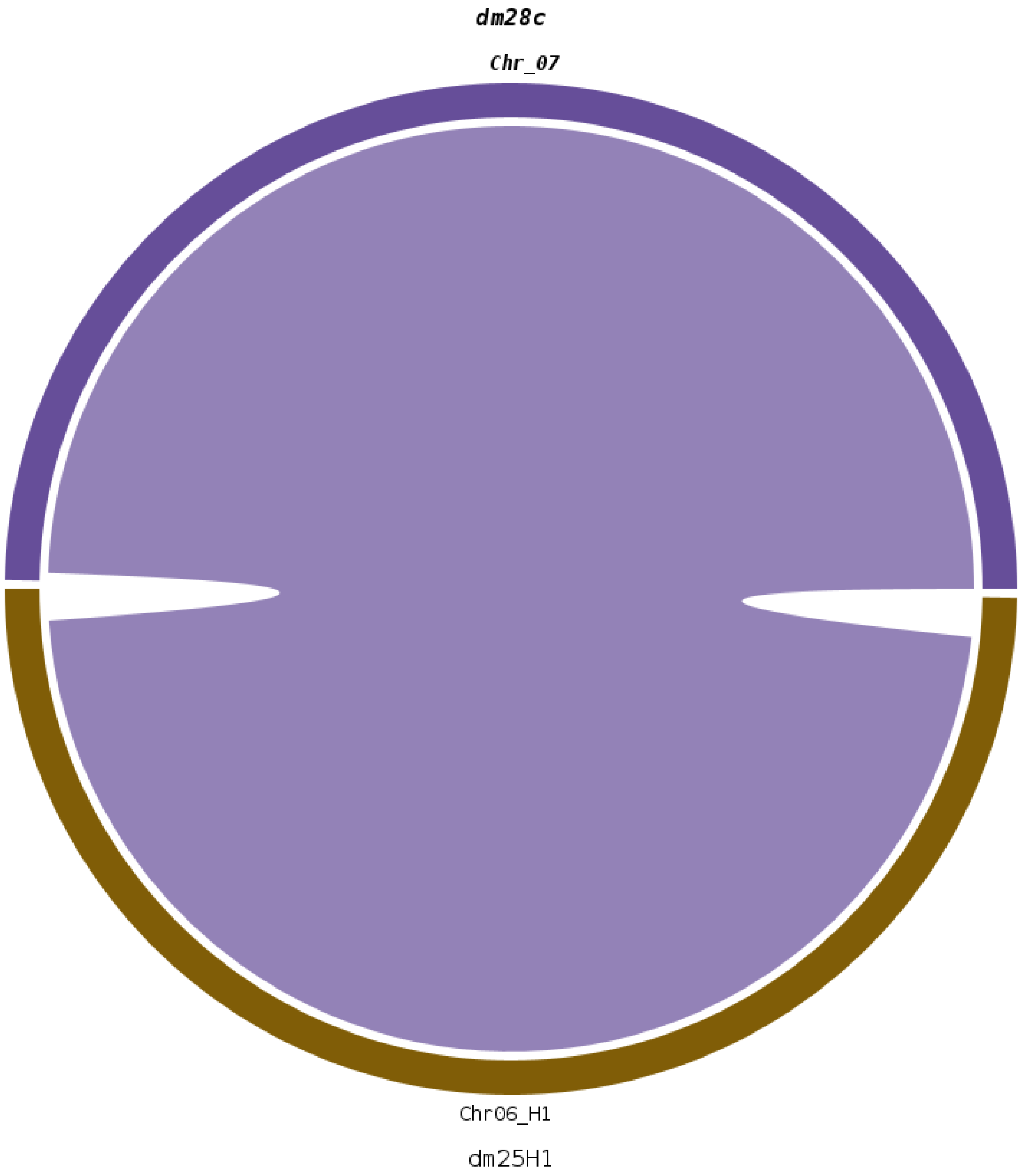
dm28c
Chr_07
Chr06_H1
dm25H1

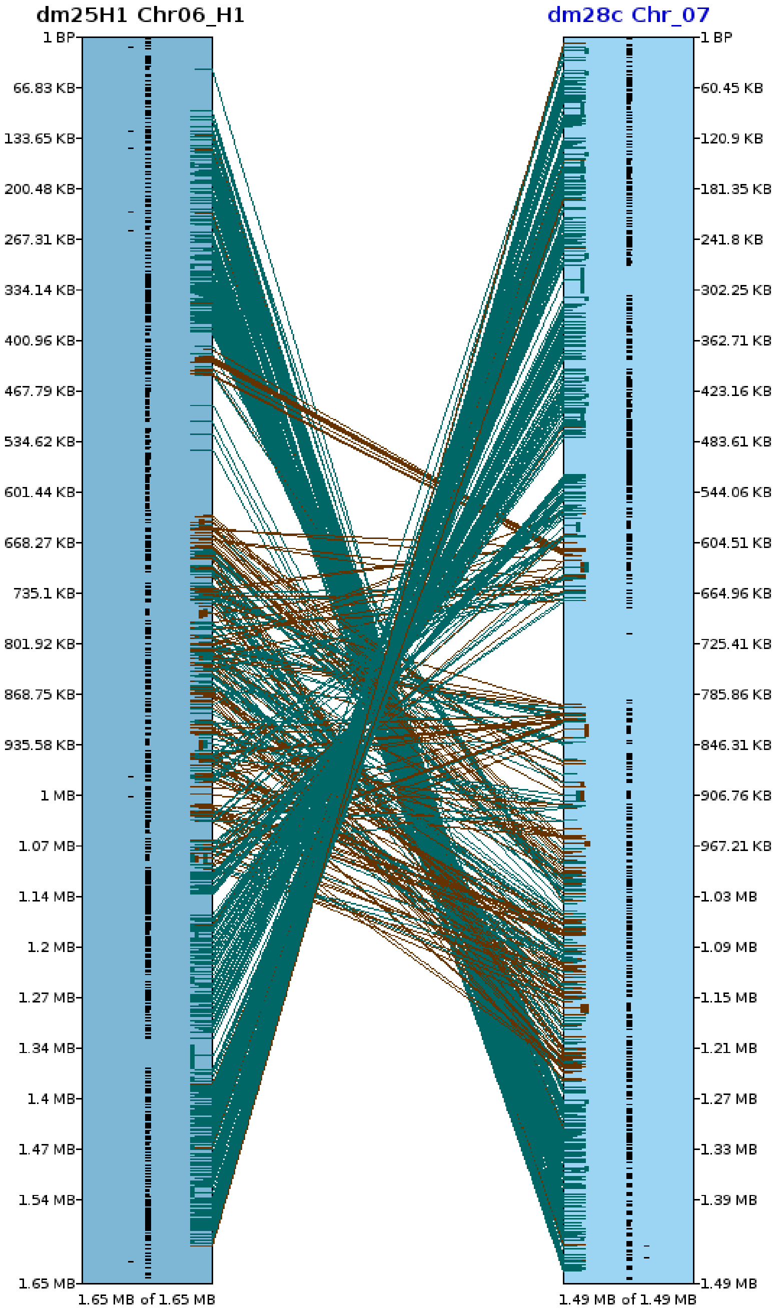
dm25H1 Chr06_H1
dm28c Chr_07
1 BP
66.83 KB
133.65 KB
200.48 KB
267.31 KB
334.14 KB
400.96 KB
467.79 KB
534.62 KB
601.44 KB
668.27 KB
735.1 KB
801.92 KB
868.75 KB
935.58 KB
1 MB
1.07 MB
1.14 MB
1.2 MB
1.27 MB
1.34 MB
1.4 MB
1.47 MB
1.54 MB
1.65 MB
1.65 MB of 1.65 MB
1 BP
60.45 KB
120.9 KB
181.35 KB
241.8 KB
302.25 KB
362.71 KB
423.16 KB
483.61 KB
544.06 KB
604.51 KB
664.96 KB
725.41 KB
785.86 KB
846.31 KB
906.76 KB
967.21 KB
1.03 MB
1.09 MB
1.15 MB
1.21 MB
1.27 MB
1.33 MB
1.39 MB
1.49 MB
1.49 MB of 1.49 MB

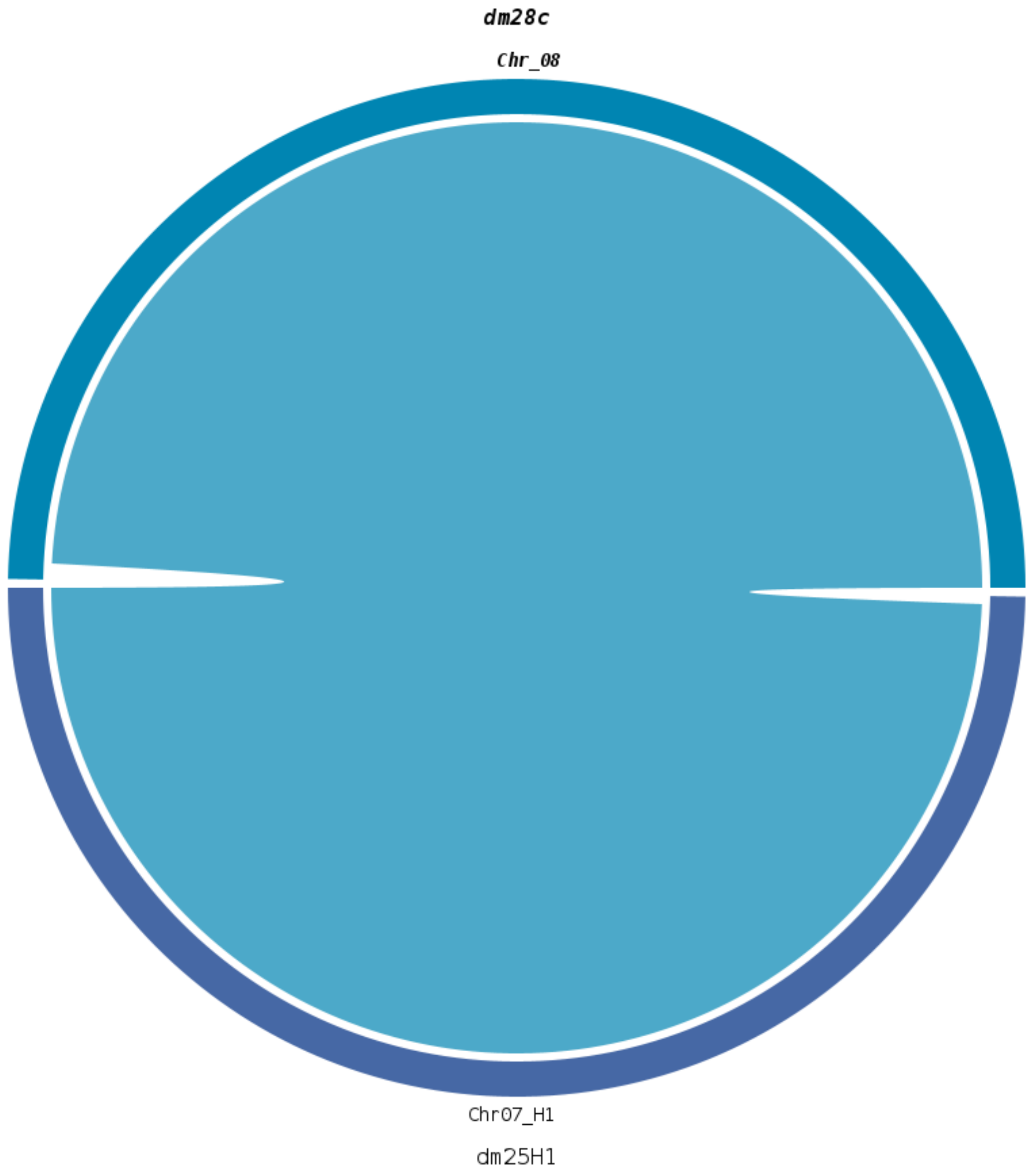
dm28c
Chr_08
Chr07_H1
dm25H1

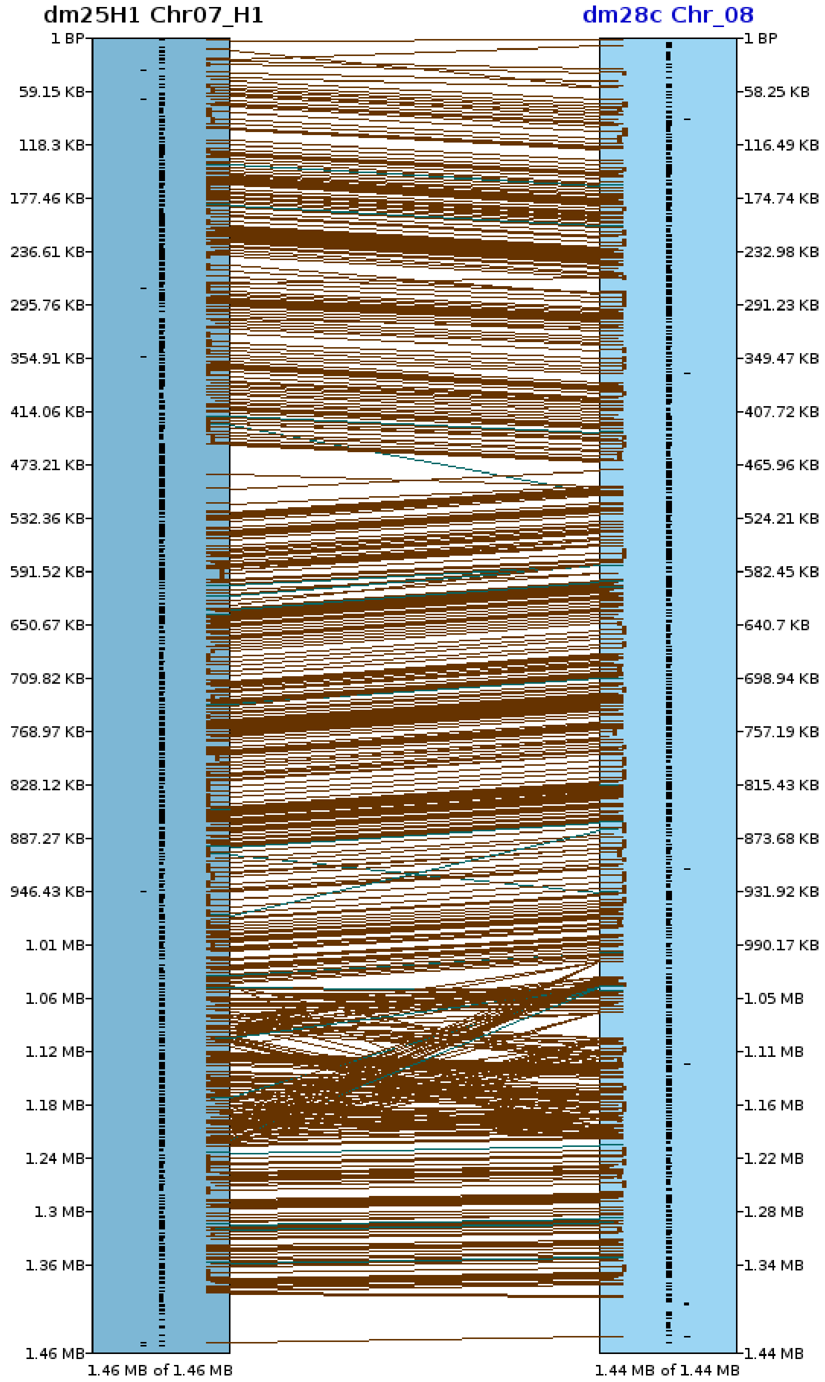
dm25H1 Chr07_H1
dm28c Chr_08
1 BP
59.15 KB
118.3 KB
177.46 KB
236.61 KB
295.76 KB
354.91 KB
414.06 KB
473.21 KB
532.36 KB
591.52 KB
650.67 KB
709.82 KB
768.97 KB
828.12 KB
887.27 KB
946.43 KB
1.01 MB
1.06 MB
1.12 MB
1.18 MB
1.24 MB
1.3 MB
1.36 MB
1.46 MB
1 BP
58.25 KB
116.49 KB
174.74 KB
232.98 KB
291.23 KB
349.47 KB
407.72 KB
465.96 KB
524.21 KB
582.45 KB
640.7 KB
698.94 KB
757.19 KB
815.43 KB
873.68 KB
931.92 KB
990.17 KB
1.05 MB
1.11 MB
1.16 MB
1.22 MB
1.28 MB
1.34 MB
1.44 MB
1.46 MB of 1.46 MB
1.44 MB of 1.44 MB

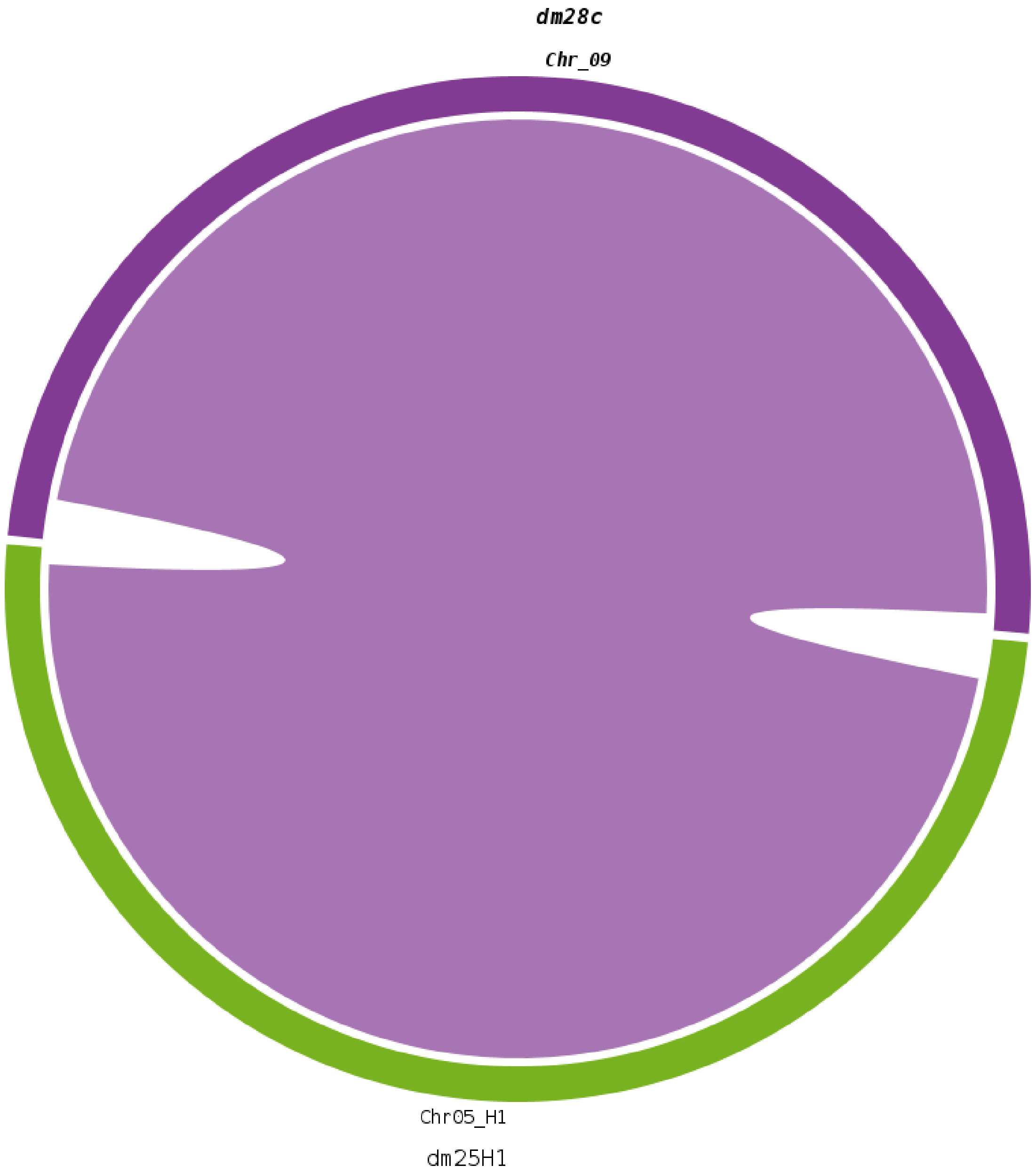
dm28c
Chr_09
Chr05_H1
dm25H1

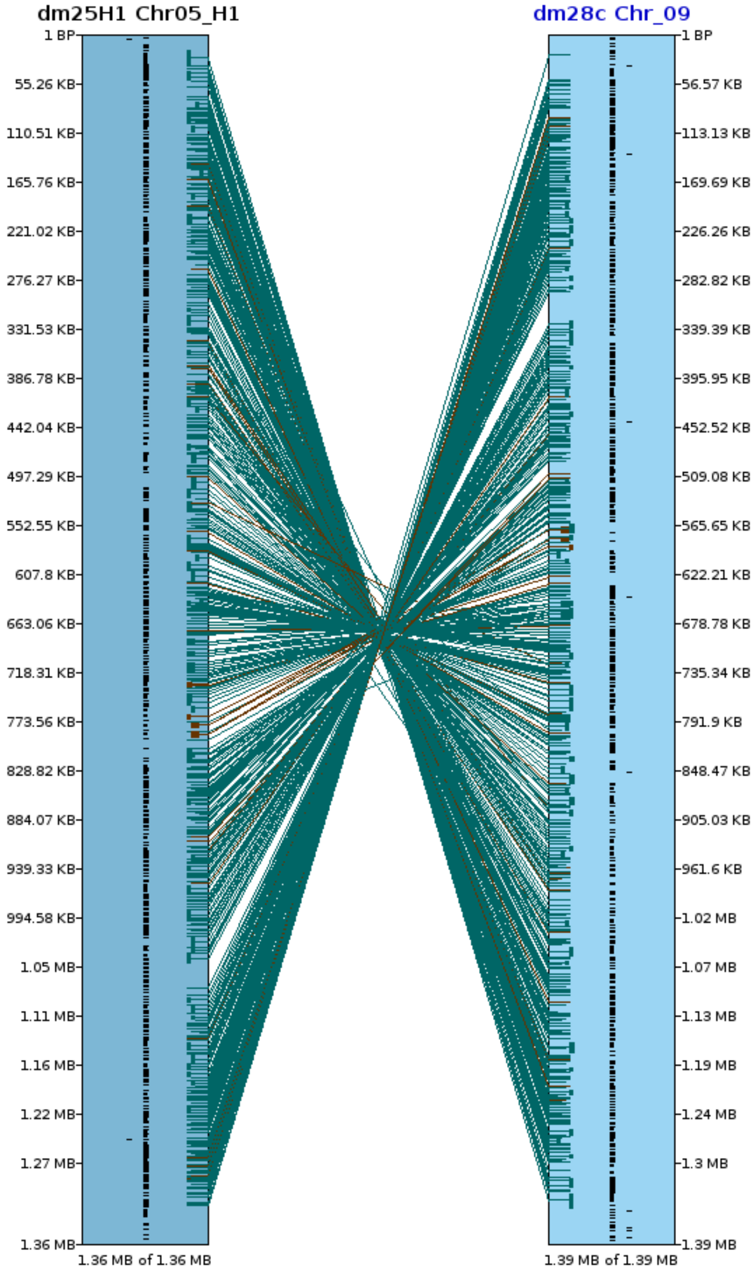
dm25H1 Chr05_H1
dm28c Chr_09
1 BP
55.26 KB
110.51 KB
165.76 KB
221.02 KB
276.27 KB
331.53 KB
386.78 KB
442.04 KB
497.29 KB
552.55 KB
607.8 KB
663.06 KB
718.31 KB
773.56 KB
828.82 KB
884.07 KB
939.33 KB
994.58 KB
1.05 MB
1.11 MB
1.16 MB
1.22 MB
1.27 MB
1.36 MB
1.36 MB of 1.36 MB
1 BP
56.57 KB
113.13 KB
169.69 KB
226.26 KB
282.82 KB
339.39 KB
395.95 KB
452.52 KB
509.08 KB
565.65 KB
622.21 KB
678.78 KB
735.34 KB
791.9 KB
848.47 KB
905.03 KB
961.6 KB
1.02 MB
1.07 MB
1.13 MB
1.19 MB
1.24 MB
1.3 MB
1.39 MB
1.39 MB of 1.39 MB

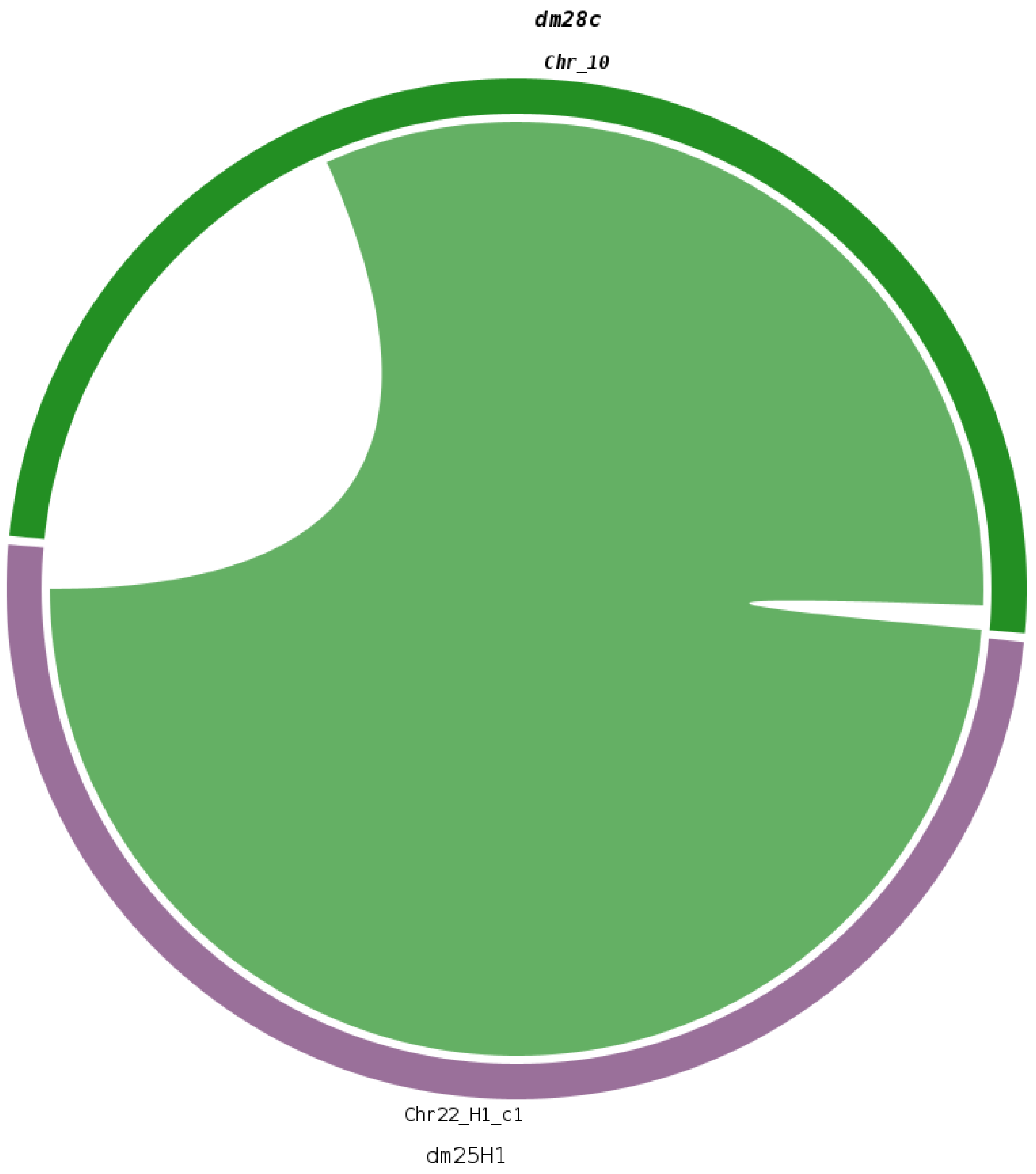

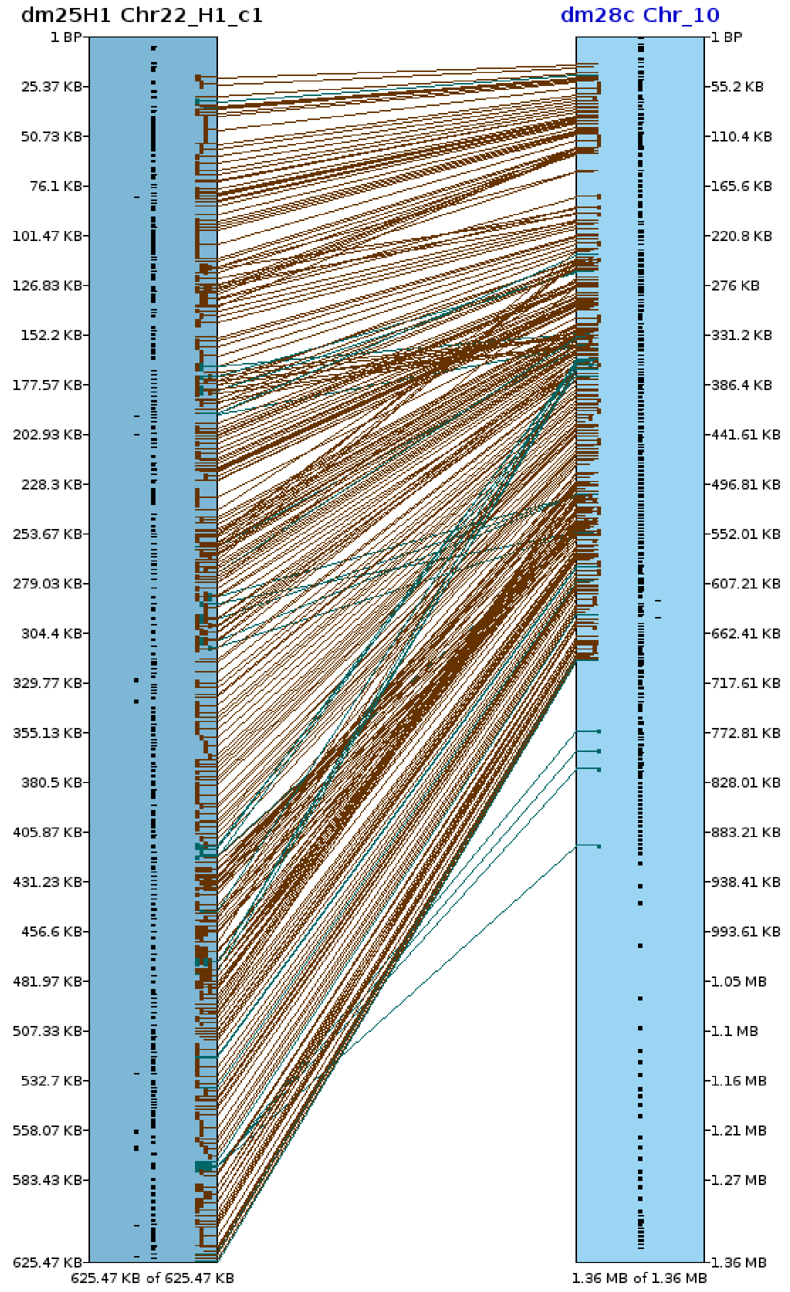

No esta el Chr22_H1_c2 en ncbi

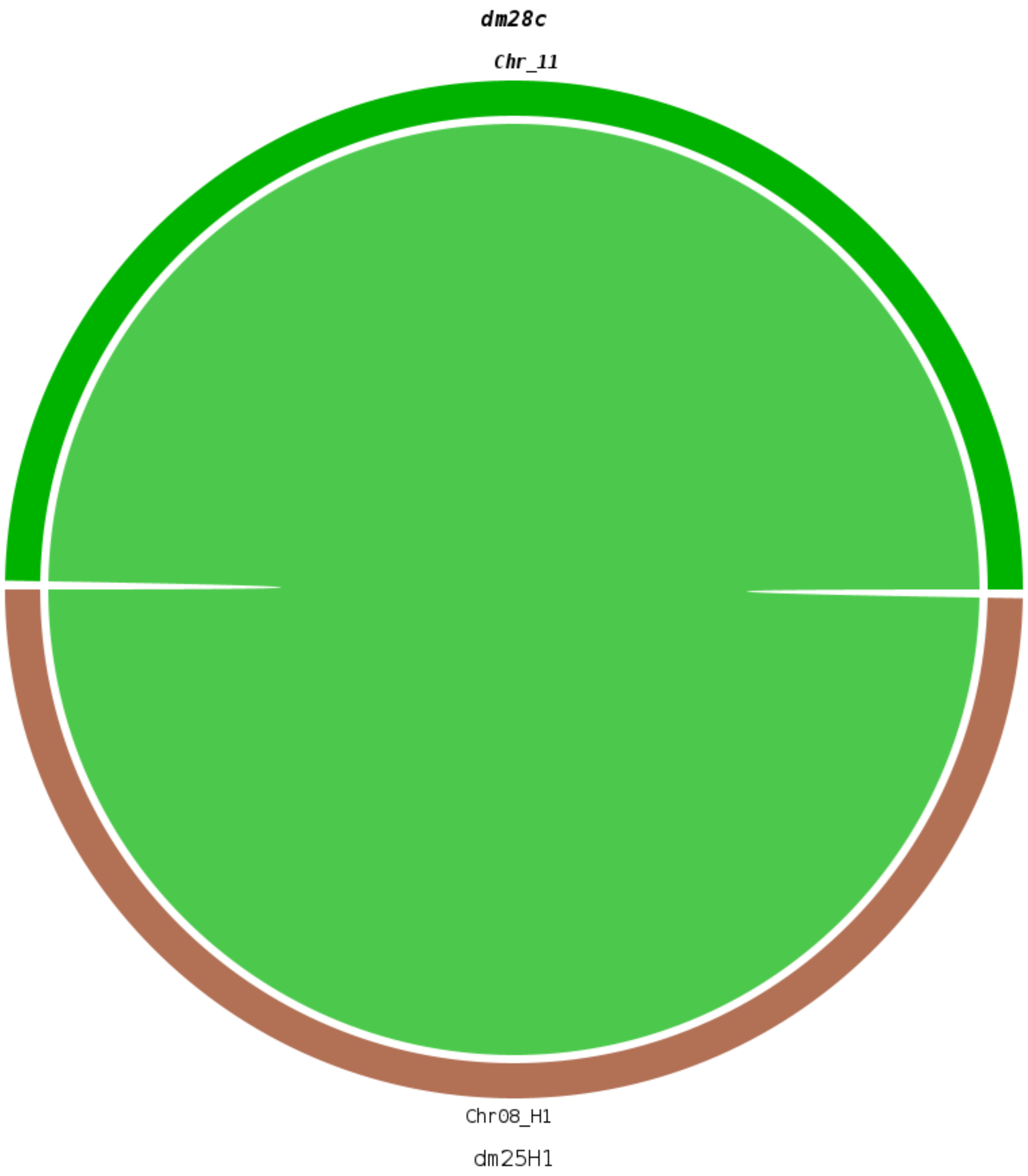
dm28c
Chr_11
Chr08_H1
dm25H1

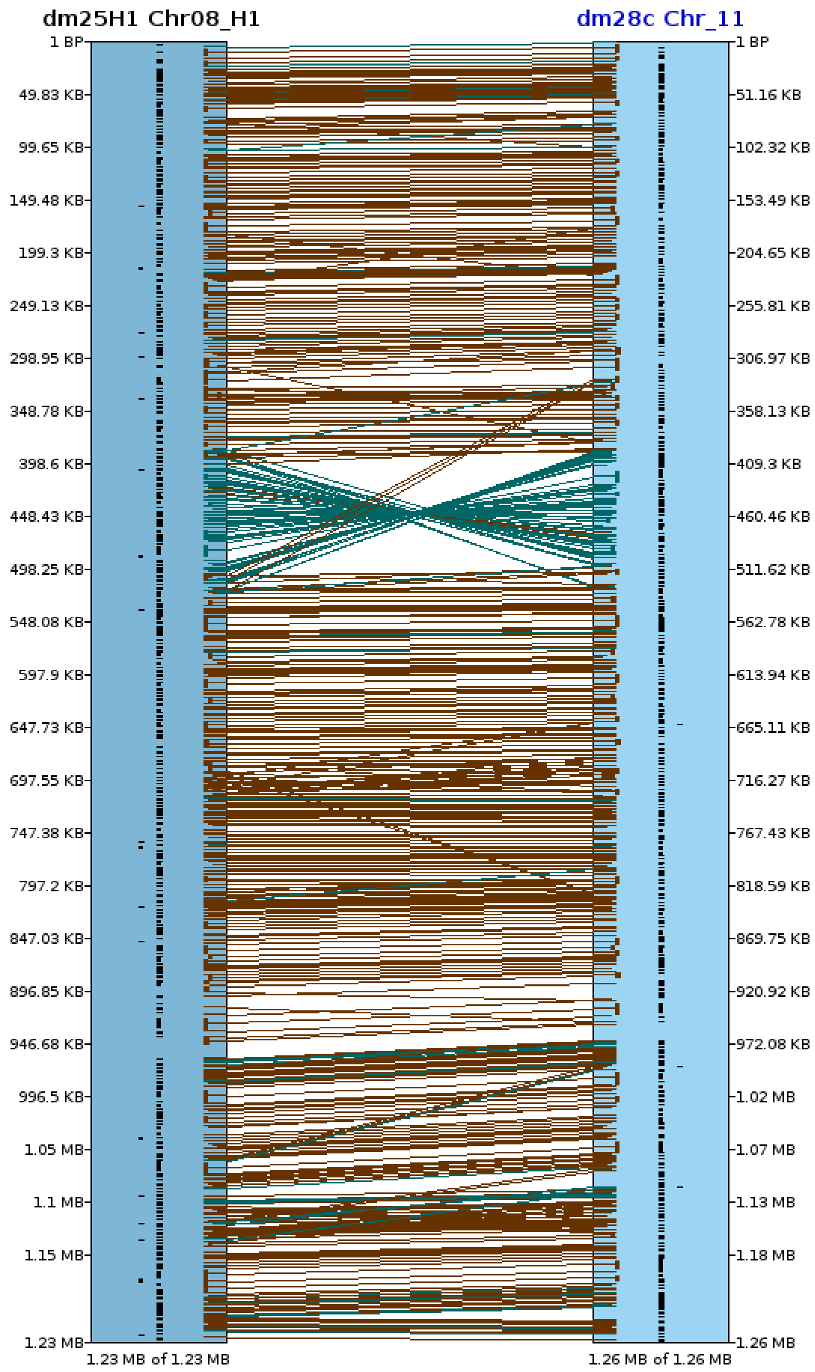
dm25H1 Chr08_H1
dm28c Chr_11
1 BP
49.83 KB
99.65 KB
149.48 KB
199.3 KB
249.13 KB
298.95 KB
348.78 KB
398.6 KB
448.43 KB
498.25 KB
548.08 KB
597.9 KB
647.73 KB
697.55 KB
747.38 KB
797.2 KB
847.03 KB
896.85 KB
946.68 KB
996.5 KB
1.05 MB
1.1 MB
1.15 MB
1.23 MB
1 BP
51.16 KB
102.32 KB
153.49 KB
204.65 KB
255.81 KB
306.97 KB
358.13 KB
409.3 KB
460.46 KB
511.62 KB
562.78 KB
613.94 KB
665.11 KB
716.27 KB
767.43 KB
818.59 KB
869.75 KB
920.92 KB
972.08 KB
1.02 MB
1.07 MB
1.13 MB
1.18 MB
1.26 MB
1.23 MB of 1.23 MB
1.26 MB of 1.26 MB

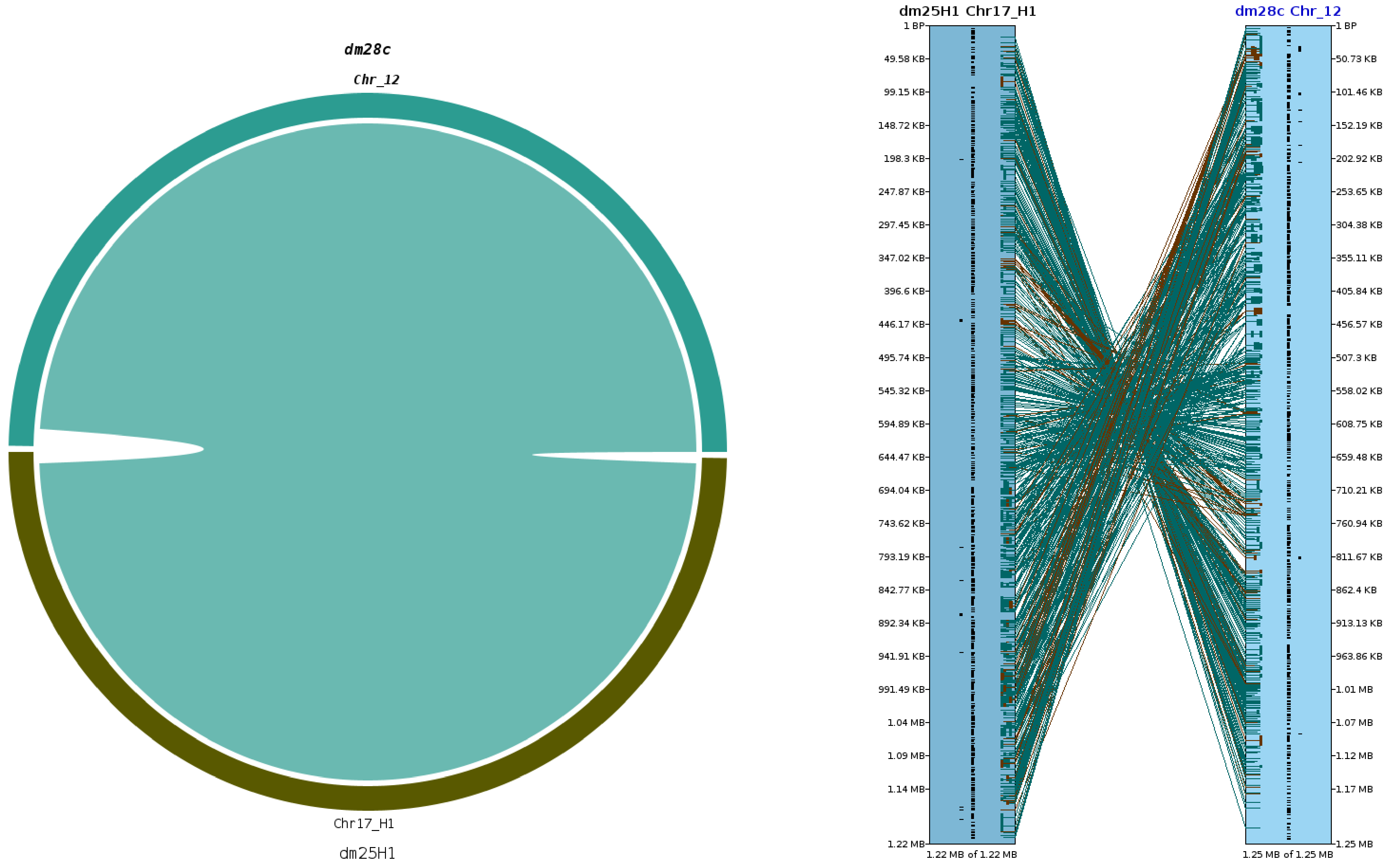
dm28c
Chr_12
Chr17_H1
dm25H1
dm25H1 Chr17_H1
dm28c Chr_12
1 BP
49.58 KB
99.15 KB
148.72 KB
198.3 KB
247.87 KB
297.45 KB
347.02 KB
396.6 KB
446.17 KB
495.74 KB
545.32 KB
594.89 KB
644.47 KB
694.04 KB
743.62 KB
793.19 KB
842.77 KB
892.34 KB
941.91 KB
991.49 KB
1.04 MB
1.09 MB
1.14 MB
1.22 MB
1.22 MB of 1.22 MB
1 BP
50.73 KB
101.46 KB
152.19 KB
202.92 KB
253.65 KB
304.38 KB
355.11 KB
405.84 KB
456.57 KB
507.3 KB
558.02 KB
608.75 KB
659.48 KB
710.21 KB
760.94 KB
811.67 KB
862.4 KB
913.13 KB
963.86 KB
1.01 MB
1.07 MB
1.12 MB
1.17 MB
1.25 MB
1.25 MB of 1.25 MB

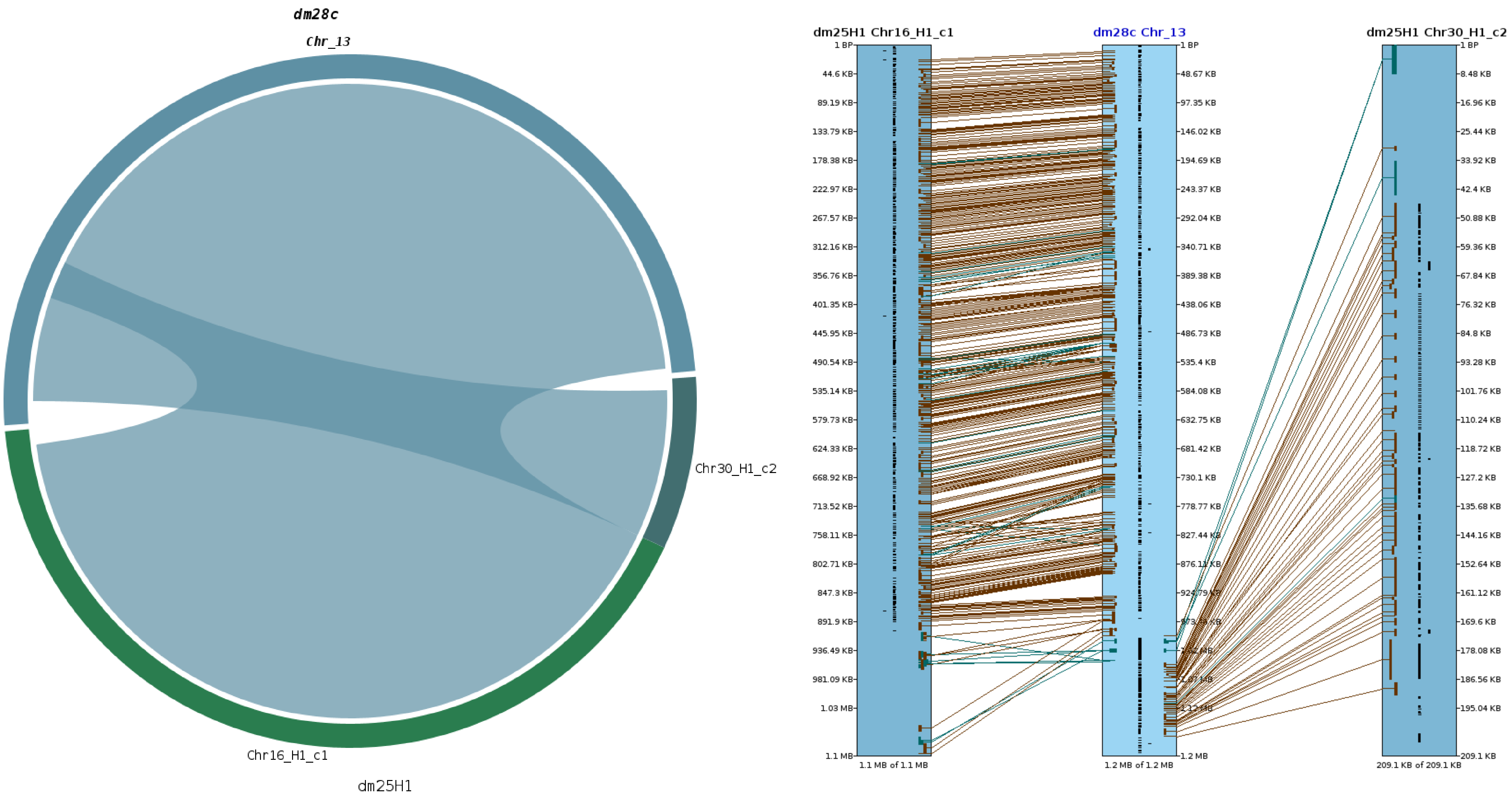

dm28c
Chr_13
Chr30_H1_c2
Chr16_H1_c1
dm25H1
dm25H1 Chr16_H1_c1
dm28c Chr_13
dm25H1 Chr30_H1_c2
1 BP
44.6 KB
89.19 KB
133.79 KB
178.38 KB
222.97 KB
267.57 KB
312.16 KB
356.76 KB
401.35 KB
445.95 KB
490.54 KB
535.14 KB
579.73 KB
624.33 KB
668.92 KB
713.52 KB
758.11 KB
802.71 KB
847.3 KB
891.9 KB
936.49 KB
981.09 KB
1.03 MB
1.1 MB
1.1 MB of 1.1 MB
1 BP
48.67 KB
97.35 KB
146.02 KB
194.69 KB
243.37 KB
292.04 KB
340.71 KB
389.38 KB
438.06 KB
486.73 KB
535.4 KB
584.08 KB
632.75 KB
681.42 KB
730.1 KB
778.77 KB
827.44 KB
876.11 KB
1.2 MB
1.2 MB of 1.2 MB
1 BP
8.48 KB
16.96 KB
25.44 KB
33.92 KB
42.4 KB
50.88 KB
59.36 KB
67.84 KB
76.32 KB
84.8 KB
93.28 KB
101.76 KB
110.24 KB
118.72 KB
127.2 KB
135.68 KB
144.16 KB
152.64 KB
161.12 KB
169.6 KB
178.08 KB
186.56 KB
195.04 KB
209.1 KB
209.1 KB of 209.1 KB

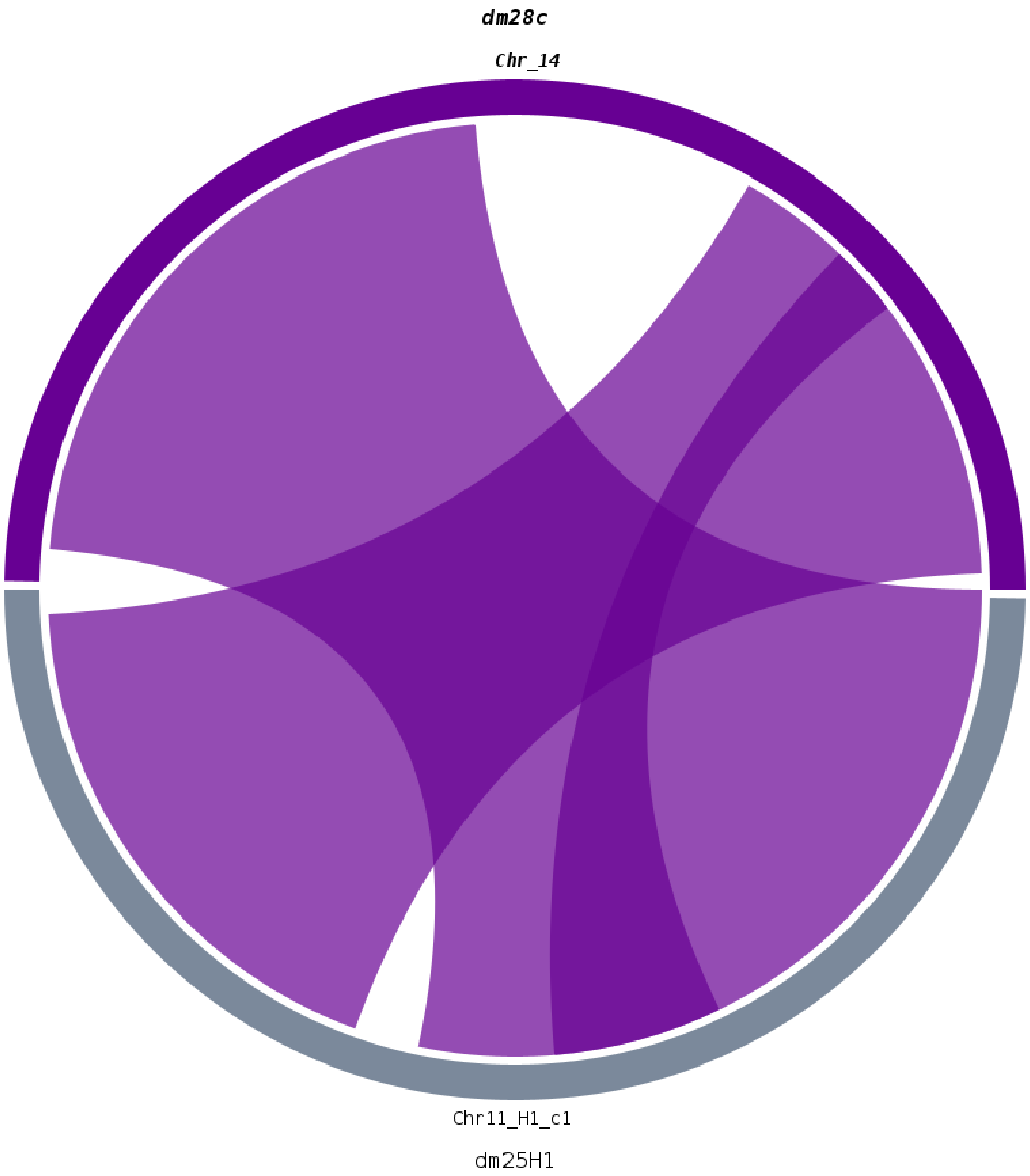
dm28c
Chr_14
Chr11_H1_c1
dm25H1

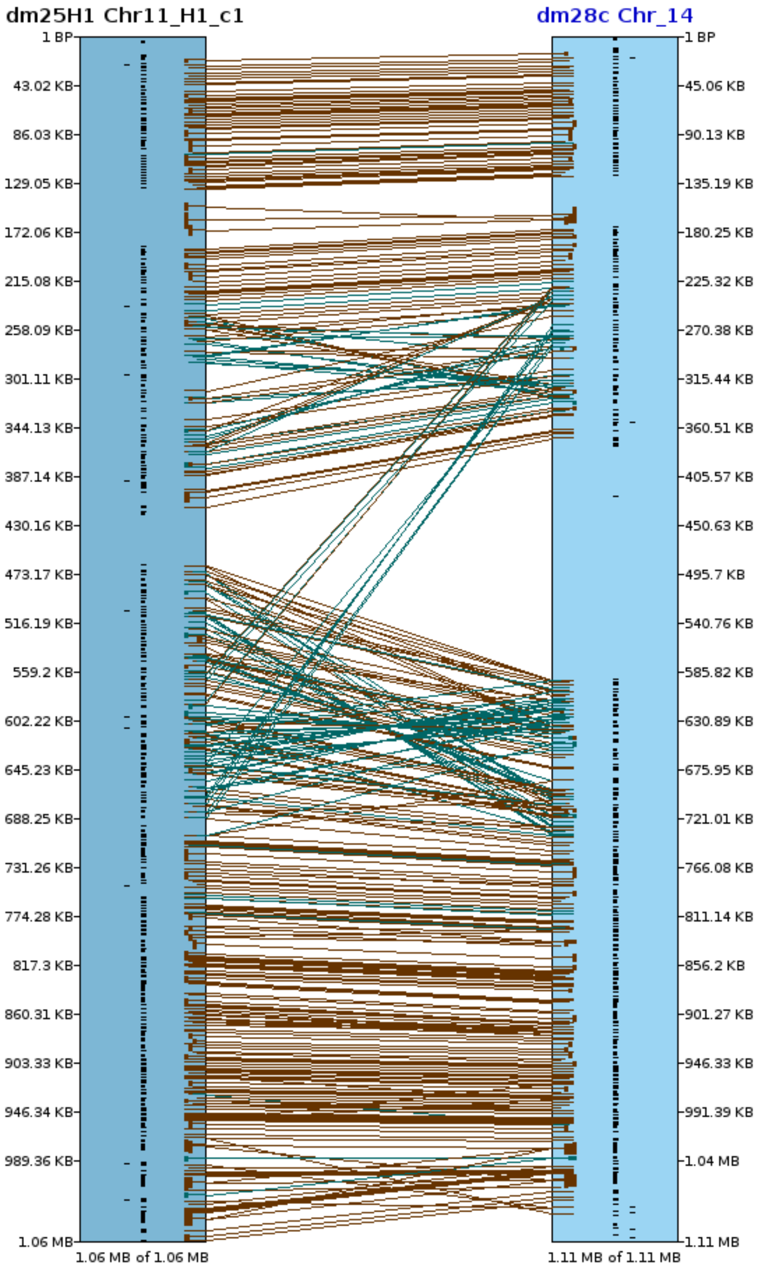
dm25H1 Chr11_H1_c1
dm28c Chr_14
1 BP
43.02 KB
86.03 KB
129.05 KB
172.06 KB
215.08 KB
258.09 KB
301.11 KB
344.13 KB
387.14 KB
430.16 KB
473.17 KB
516.19 KB
559.2 KB
602.22 KB
645.23 KB
688.25 KB
731.26 KB
774.28 KB
817.3 KB
860.31 KB
903.33 KB
946.34 KB
989.36 KB
1.06 MB
1.06 MB of 1.06 MB
1 BP
45.06 KB
90.13 KB
135.19 KB
180.25 KB
225.32 KB
270.38 KB
315.44 KB
360.51 KB
405.57 KB
450.63 KB
495.7 KB
540.76 KB
585.82 KB
630.89 KB
675.95 KB
721.01 KB
766.08 KB
811.14 KB
856.2 KB
901.27 KB
946.33 KB
991.39 KB
1.04 MB
1.11 MB
1.11 MB of 1.11 MB

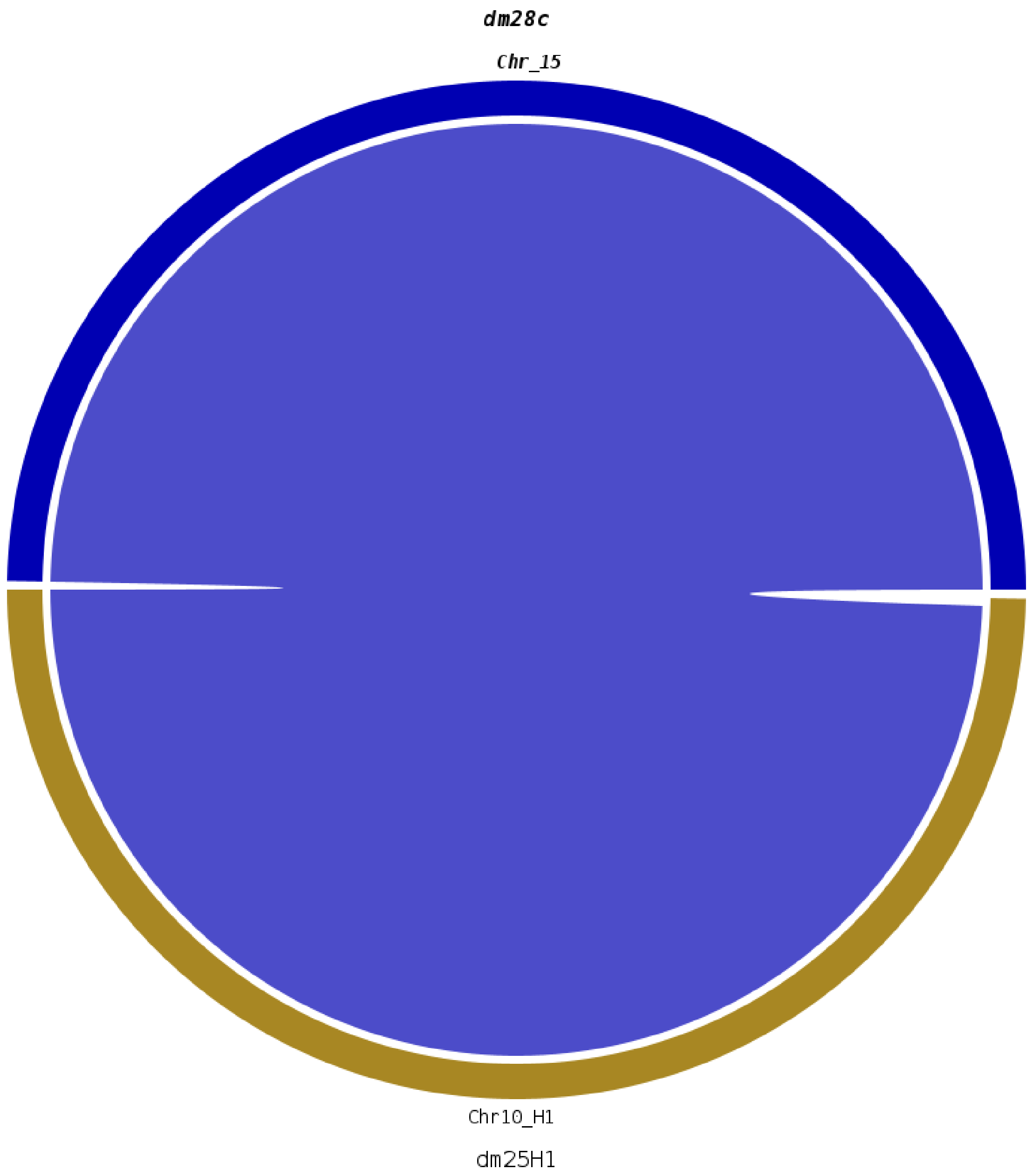
dm28c
Chr_15
Chr10_H1
dm25H1

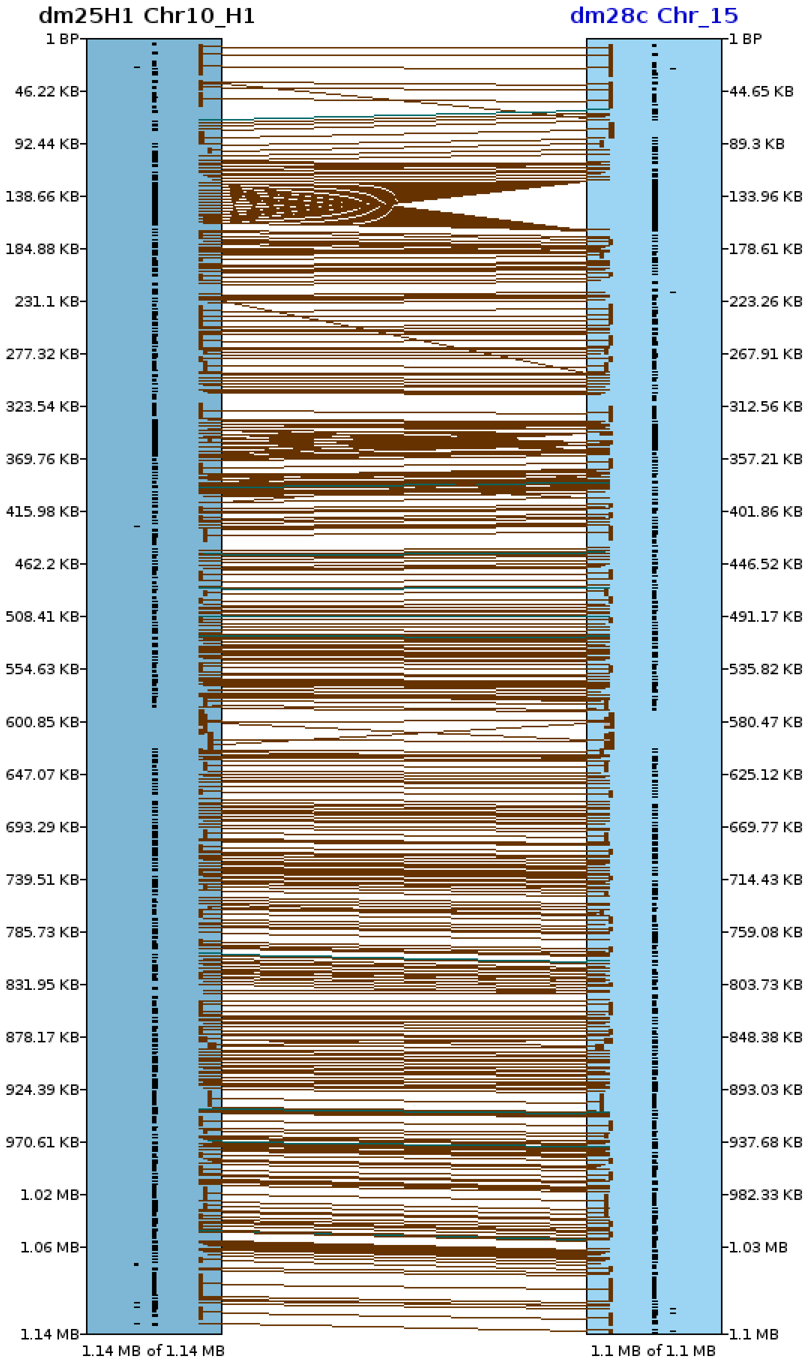
dm25H1 Chr10_H1
dm28c Chr_15
1 BP
46.22 KB
92.44 KB
138.66 KB
184.88 KB
231.1 KB
277.32 KB
323.54 KB
369.76 KB
415.98 KB
462.2 KB
508.41 KB
554.63 KB
600.85 KB
647.07 KB
693.29 KB
739.51 KB
785.73 KB
831.95 KB
878.17 KB
924.39 KB
970.61 KB
1.02 MB
1.06 MB
1.14 MB
1 BP
44.65 KB
89.3 KB
133.96 KB
178.61 KB
223.26 KB
267.91 KB
312.56 KB
357.21 KB
401.86 KB
446.52 KB
491.17 KB
535.82 KB
580.47 KB
625.12 KB
669.77 KB
714.43 KB
759.08 KB
803.73 KB
848.38 KB
893.03 KB
937.68 KB
982.33 KB
1.03 MB
1.1 MB
1.14 MB of 1.14 MB
1.1 MB of 1.1 MB

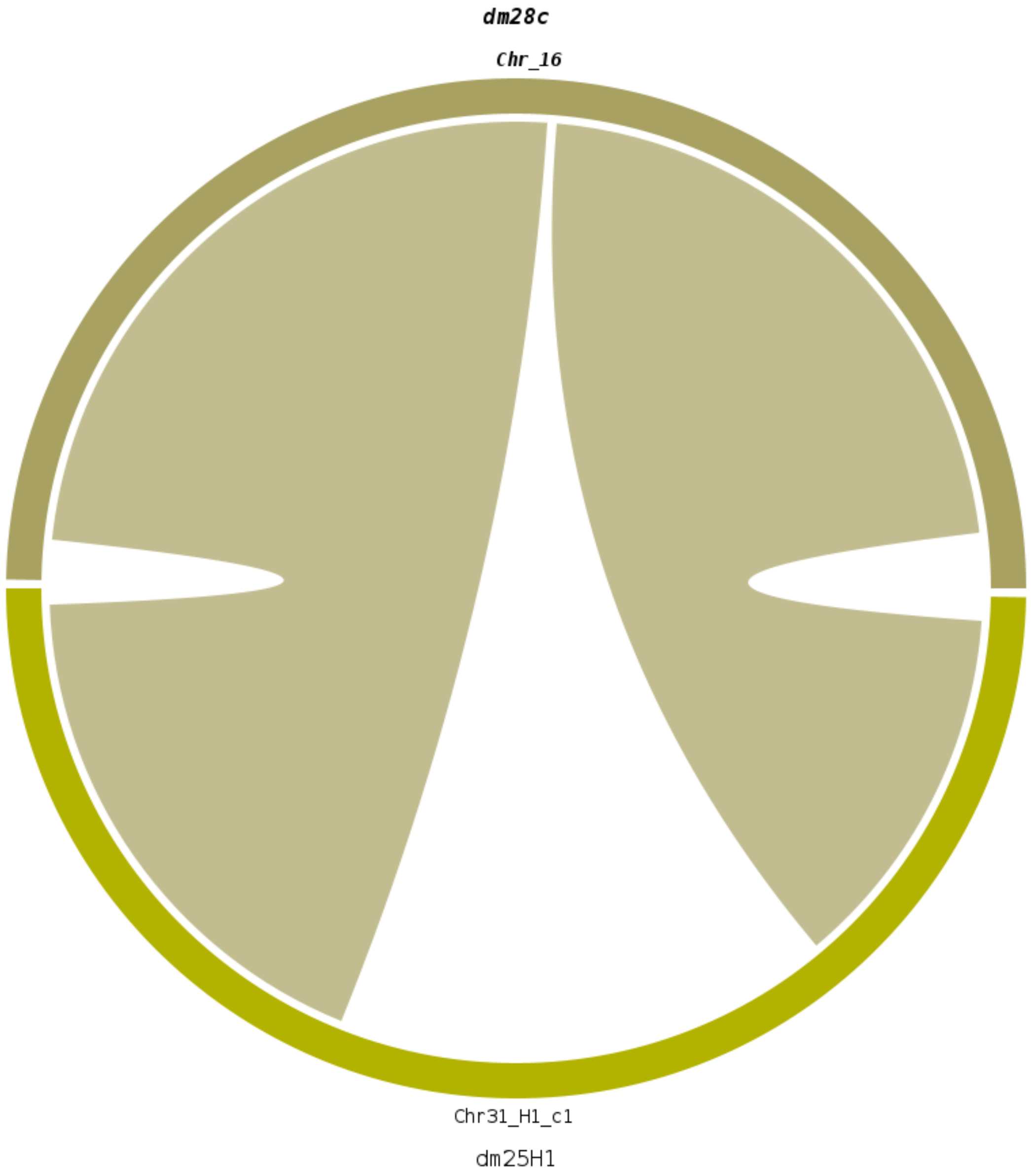
dm28c
Chr_16
Chr31_H1_c1
dm25H1

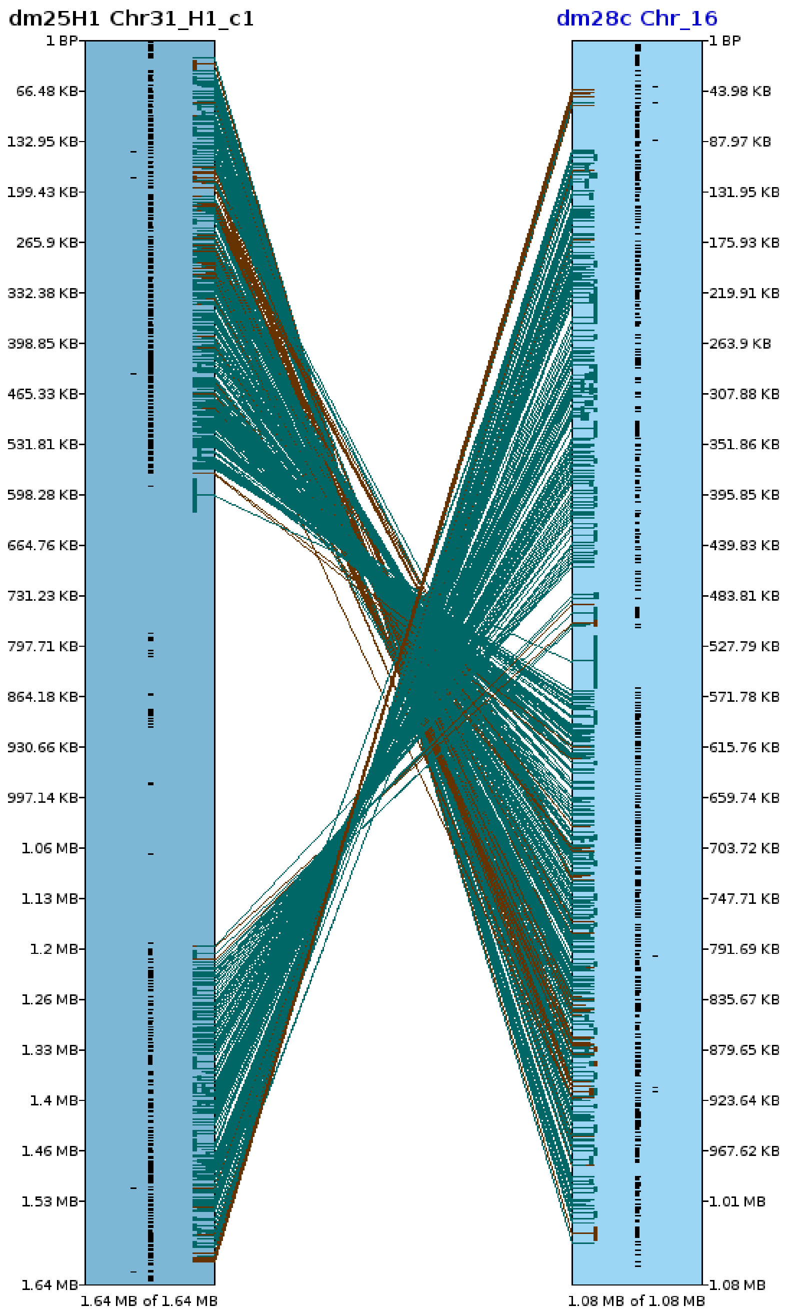
dm25H1 Chr31_H1_c1
dm28c Chr_16
1 BP
66.48 KB
132.95 KB
199.43 KB
265.9 KB
332.38 KB
398.85 KB
465.33 KB
531.81 KB
598.28 KB
664.76 KB
731.23 KB
797.71 KB
864.18 KB
930.66 KB
997.14 KB
1.06 MB
1.13 MB
1.2 MB
1.26 MB
1.33 MB
1.4 MB
1.46 MB
1.53 MB
1.64 MB
1.64 MB of 1.64 MB
1 BP
43.98 KB
87.97 KB
131.95 KB
175.93 KB
219.91 KB
263.9 KB
307.88 KB
351.86 KB
395.85 KB
439.83 KB
483.81 KB
527.79 KB
571.78 KB
615.76 KB
659.74 KB
703.72 KB
747.71 KB
791.69 KB
835.67 KB
879.65 KB
923.64 KB
967.62 KB
1.01 MB
1.08 MB
1.08 MB of 1.08 MB

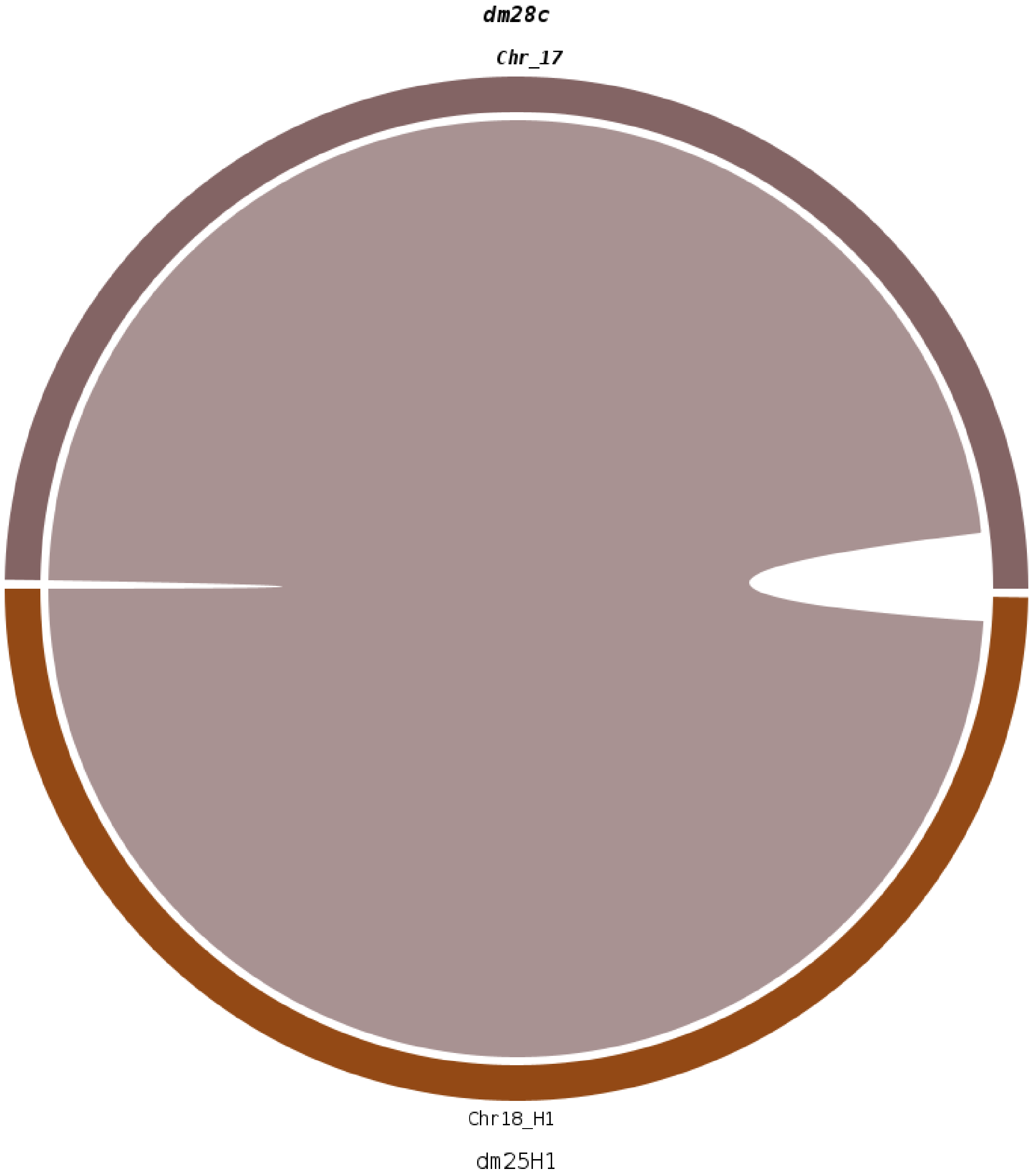
dm28c
Chr_17
Chr18_H1
dm25H1

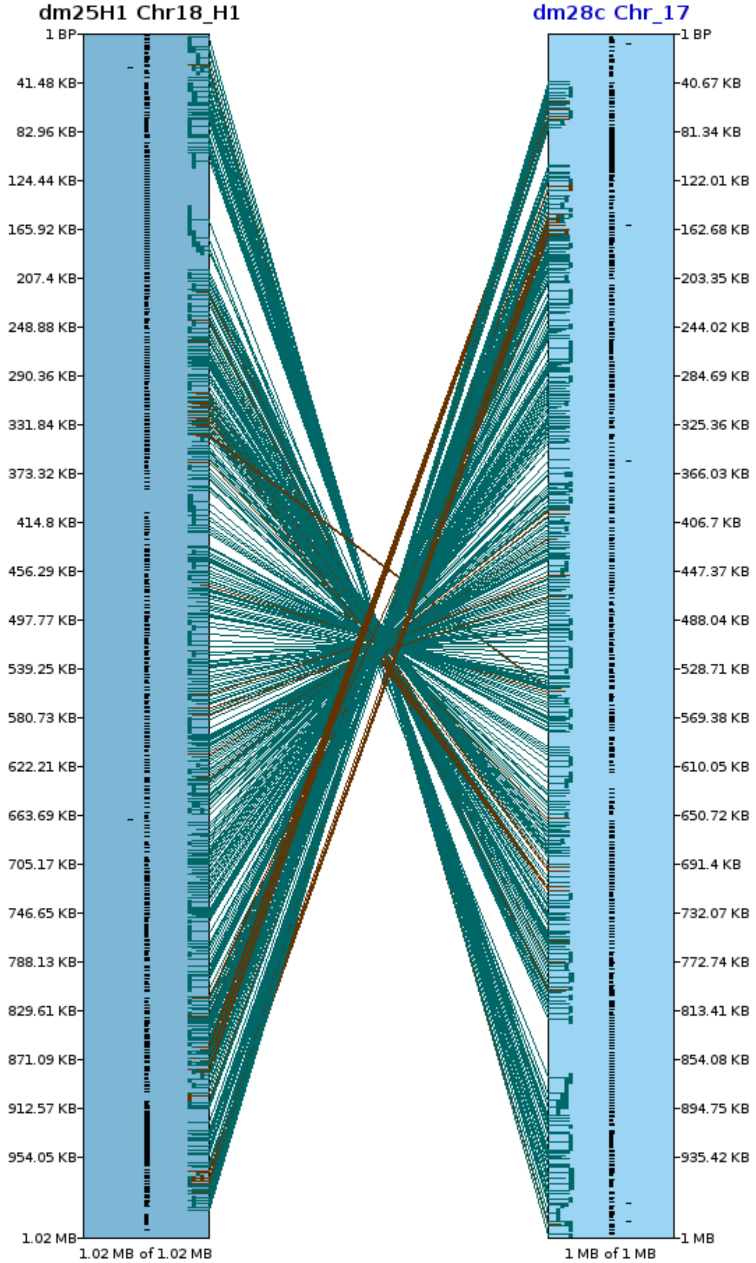
dm25H1 Chr18_H1
dm28c Chr_17
1 BP
41.48 KB
82.96 KB
124.44 KB
165.92 KB
207.4 KB
248.88 KB
290.36 KB
331.84 KB
373.32 KB
414.8 KB
456.29 KB
497.77 KB
539.25 KB
580.73 KB
622.21 KB
663.69 KB
705.17 KB
746.65 KB
788.13 KB
829.61 KB
871.09 KB
912.57 KB
954.05 KB
1.02 MB
1.02 MB of 1.02 MB
1 BP
40.67 KB
81.34 KB
122.01 KB
162.68 KB
203.35 KB
244.02 KB
284.69 KB
325.36 KB
366.03 KB
406.7 KB
447.37 KB
488.04 KB
528.71 KB
569.38 KB
610.05 KB
650.72 KB
691.4 KB
732.07 KB
772.74 KB
813.41 KB
854.08 KB
894.75 KB
935.42 KB
1 MB
1 MB of 1 MB

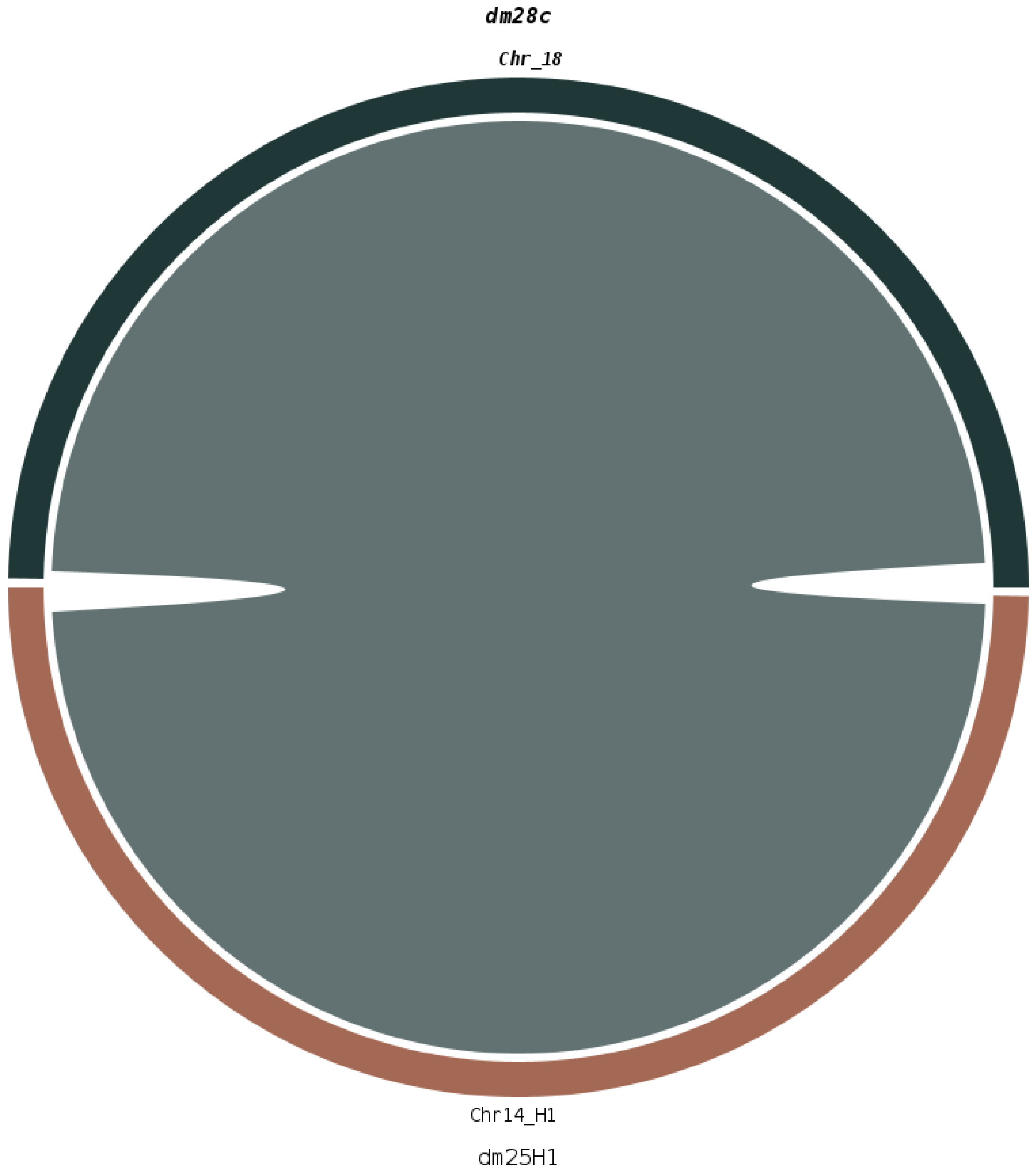
dm28c
Chr_18
Chr14_H1
dm25H1

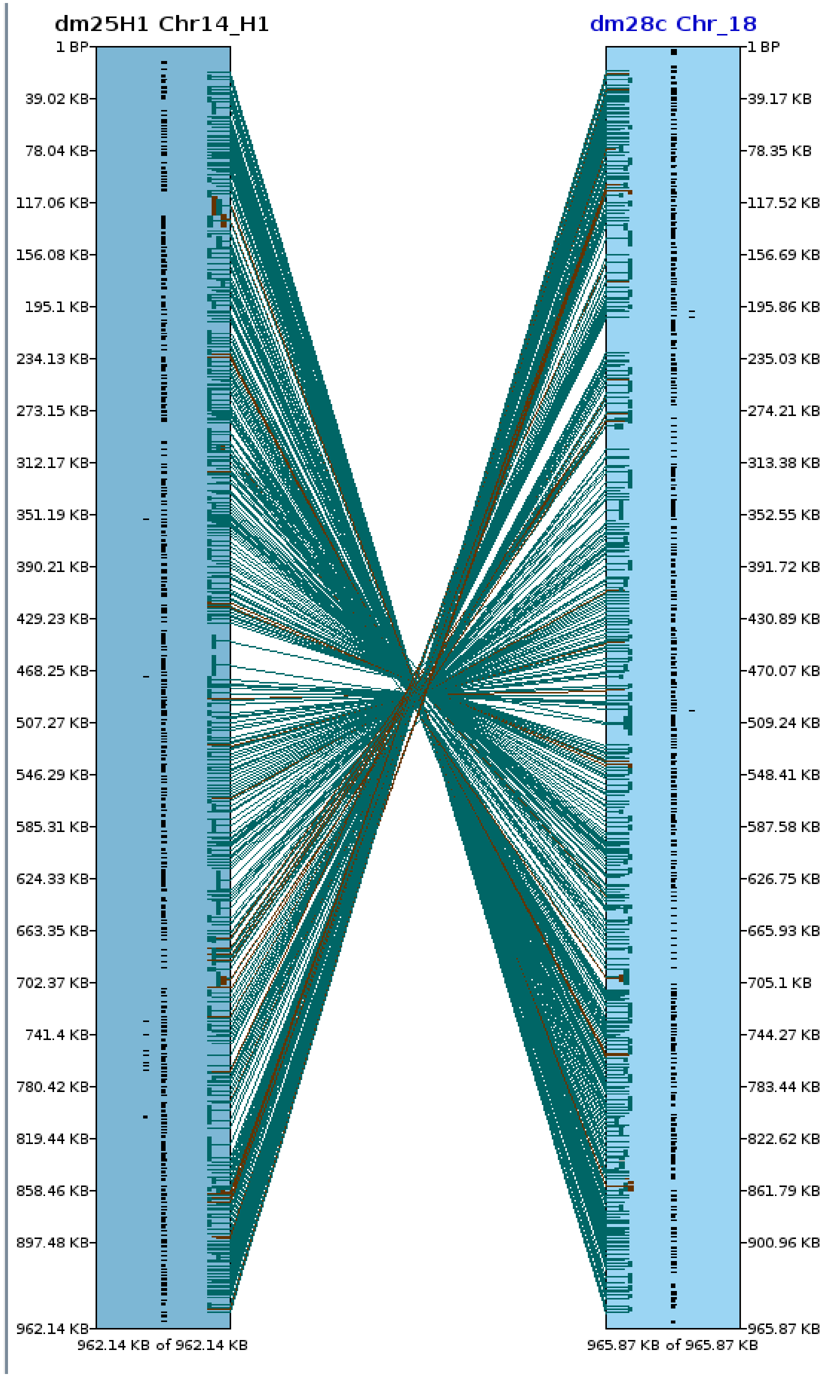
dm25H1 Chr14_H1
dm28c Chr_18
1 BP
39.02 KB
78.04 KB
117.06 KB
156.08 KB
195.1 KB
234.13 KB
273.15 KB
312.17 KB
351.19 KB
390.21 KB
429.23 KB
468.25 KB
507.27 KB
546.29 KB
585.31 KB
624.33 KB
663.35 KB
702.37 KB
741.4 KB
780.42 KB
819.44 KB
858.46 KB
897.48 KB
962.14 KB
962.14 KB of 962.14 KB
1 BP
39.17 KB
78.35 KB
117.52 KB
156.69 KB
195.86 KB
235.03 KB
274.21 KB
313.38 KB
352.55 KB
391.72 KB
430.89 KB
470.07 KB
509.24 KB
548.41 KB
587.58 KB
626.75 KB
665.93 KB
705.1 KB
744.27 KB
783.44 KB
822.62 KB
861.79 KB
900.96 KB
965.87 KB
965.87 KB of 965.87 KB

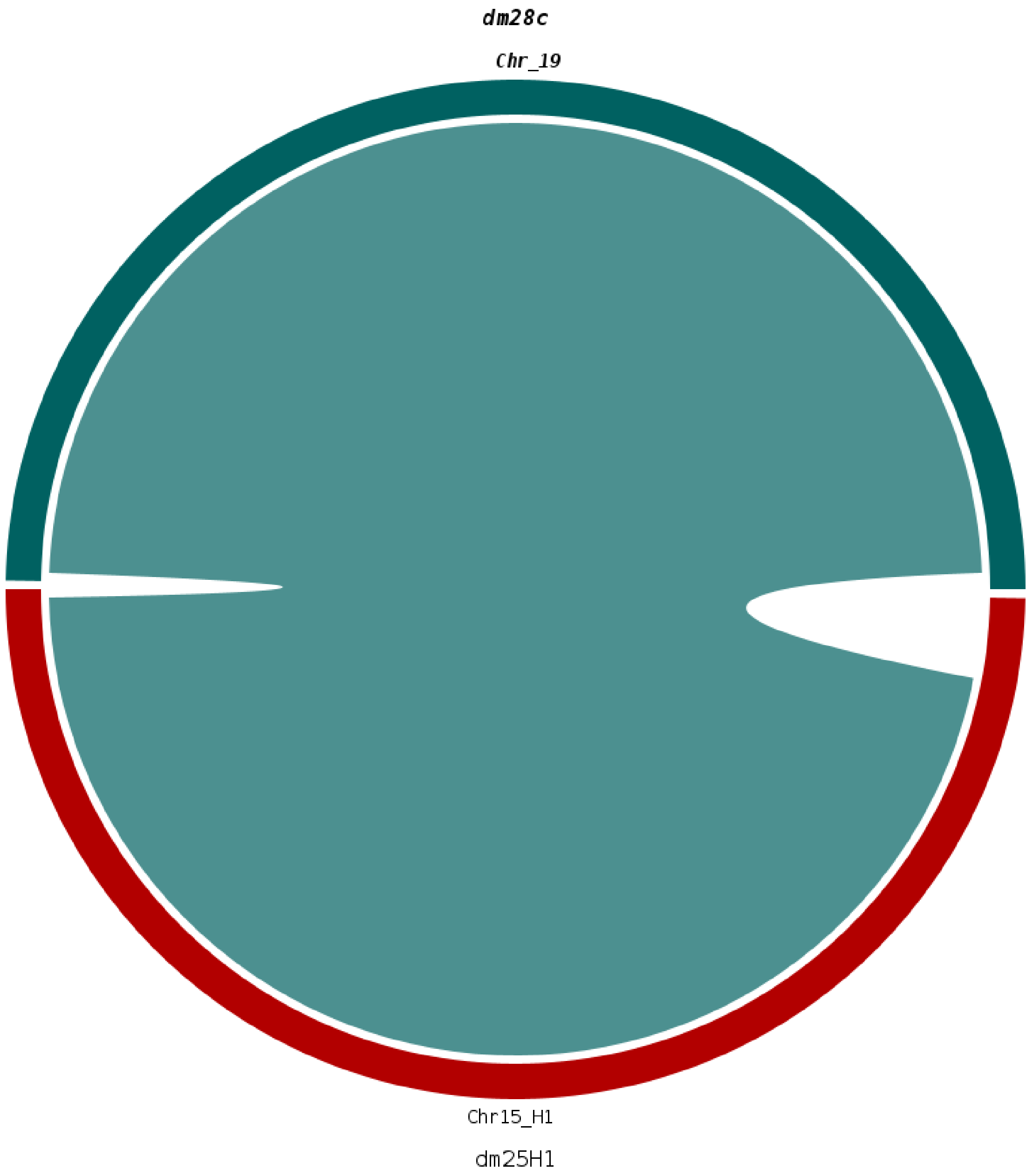
dm28c
Chr_19
Chr15_H1
dm25H1

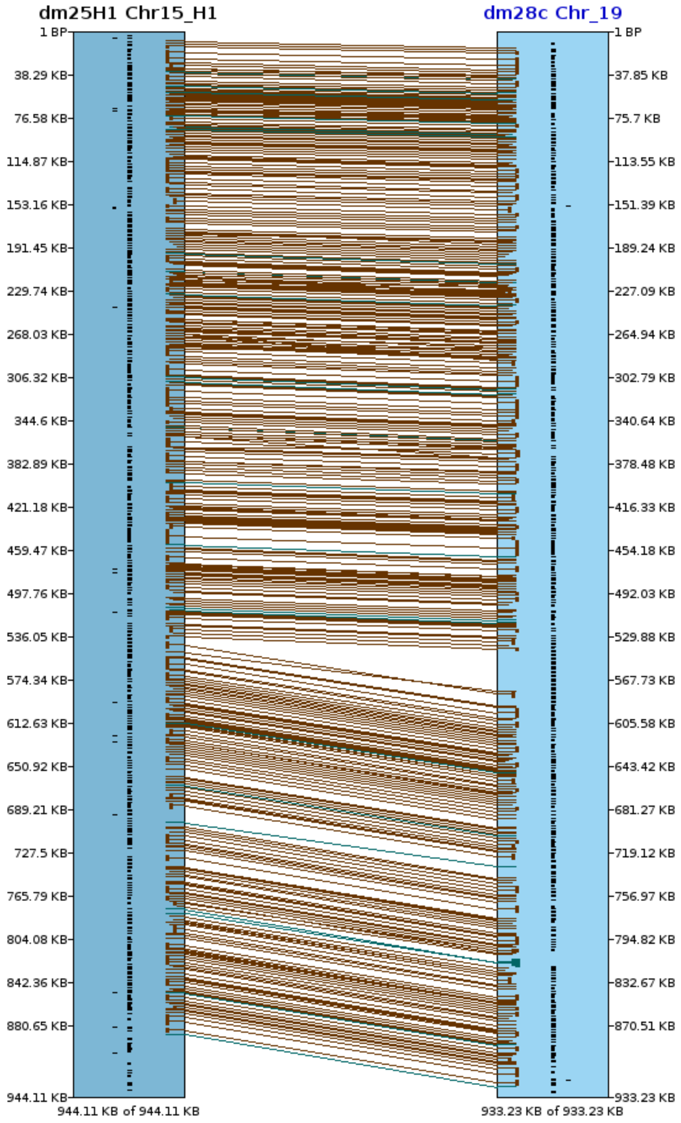
dm25H1 Chr15_H1
dm28c Chr_19
1 BP
38.29 KB
76.58 KB
114.87 KB
153.16 KB
191.45 KB
229.74 KB
268.03 KB
306.32 KB
344.6 KB
382.89 KB
421.18 KB
459.47 KB
497.76 KB
536.05 KB
574.34 KB
612.63 KB
650.92 KB
689.21 KB
727.5 KB
765.79 KB
804.08 KB
842.36 KB
880.65 KB
944.11 KB
944.11 KB of 944.11 KB
1 BP
37.85 KB
75.7 KB
113.55 KB
151.39 KB
189.24 KB
227.09 KB
264.94 KB
302.79 KB
340.64 KB
378.48 KB
416.33 KB
454.18 KB
492.03 KB
529.88 KB
567.73 KB
605.58 KB
643.42 KB
681.27 KB
719.12 KB
756.97 KB
794.82 KB
832.67 KB
870.51 KB
933.23 KB
933.23 KB of 933.23 KB

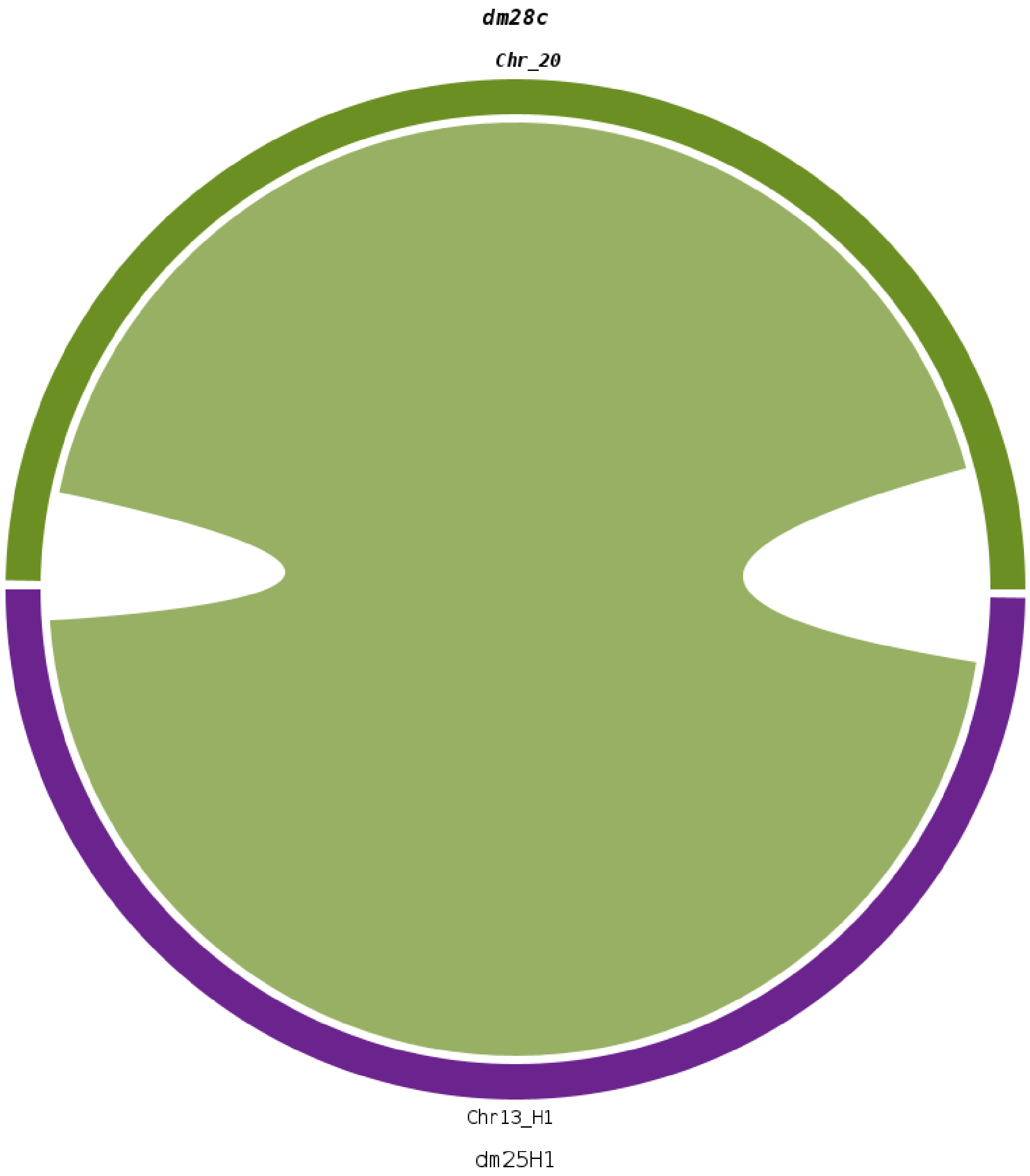
dm28c
Chr_20
Chr13_H1
dm25H1

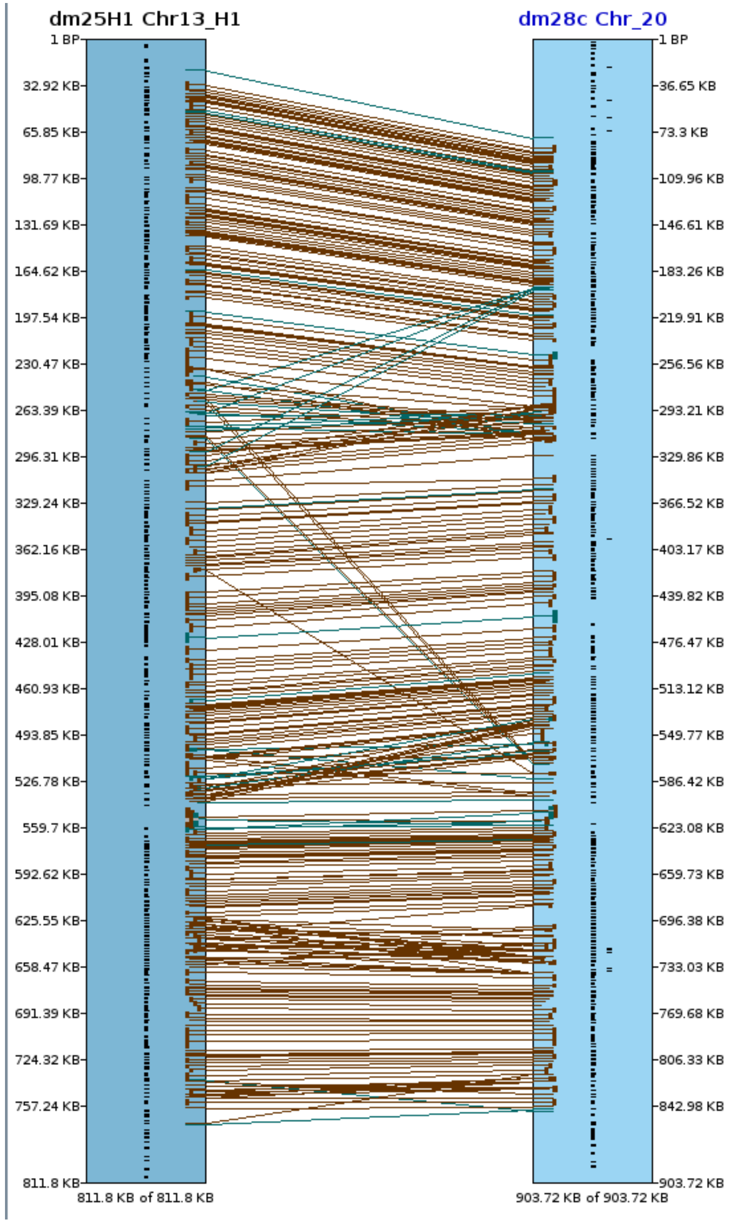
dm25H1 Chr13_H1
dm28c Chr_20
1 BP
32.92 KB
65.85 KB
98.77 KB
131.69 KB
164.62 KB
197.54 KB
230.47 KB
263.39 KB
296.31 KB
329.24 KB
362.16 KB
395.08 KB
428.01 KB
460.93 KB
493.85 KB
526.78 KB
559.7 KB
592.62 KB
625.55 KB
658.47 KB
691.39 KB
724.32 KB
757.24 KB
811.8 KB
1 BP
36.65 KB
73.3 KB
109.96 KB
146.61 KB
183.26 KB
219.91 KB
256.56 KB
293.21 KB
329.86 KB
366.52 KB
403.17 KB
439.82 KB
476.47 KB
513.12 KB
549.77 KB
586.42 KB
623.08 KB
659.73 KB
696.38 KB
733.03 KB
769.68 KB
806.33 KB
842.98 KB
903.72 KB
811.8 KB of 811.8 KB
903.72 KB of 903.72 KB

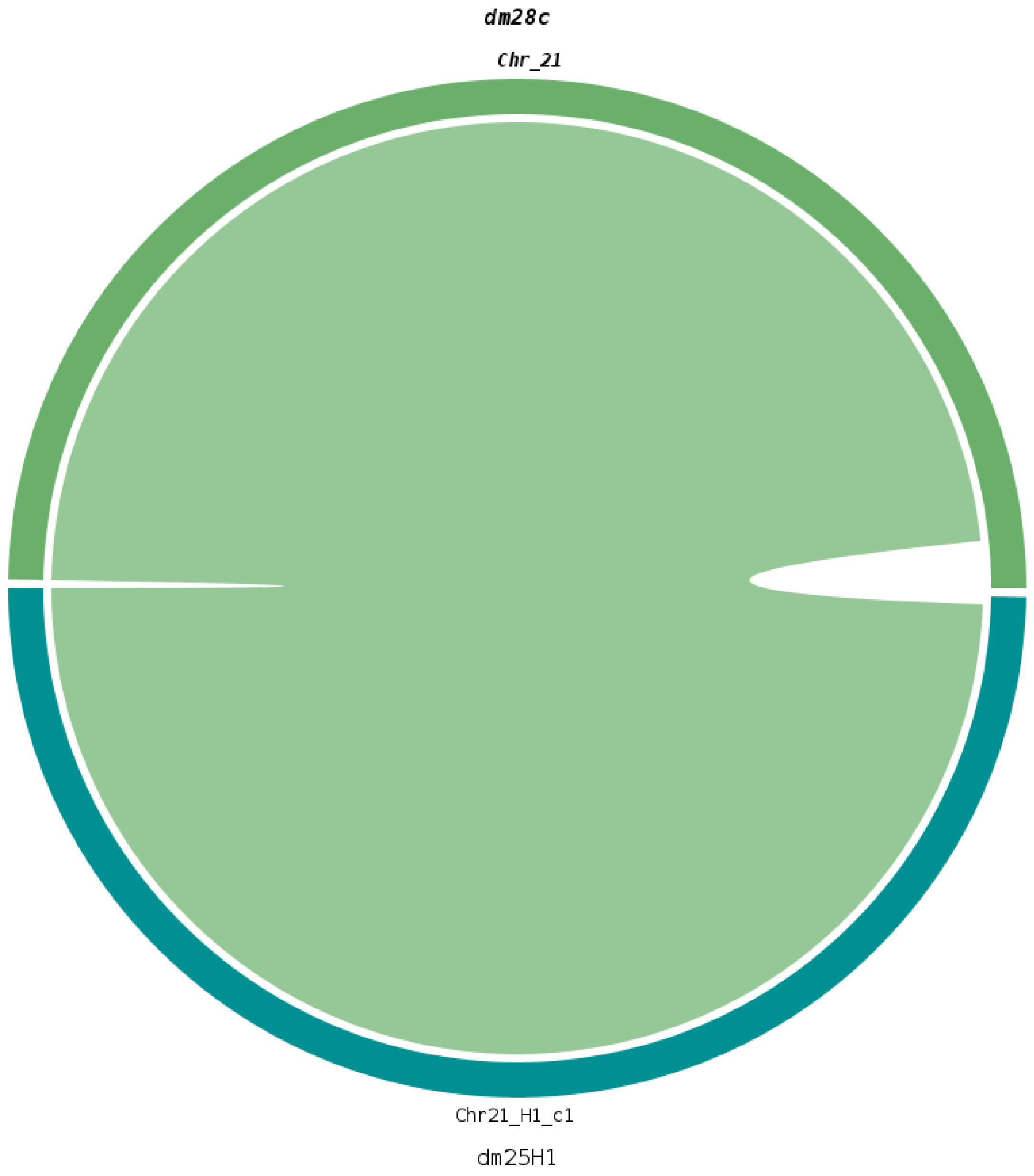
dm28c
Chr_21
Chr21_H1_c1
dm25H1

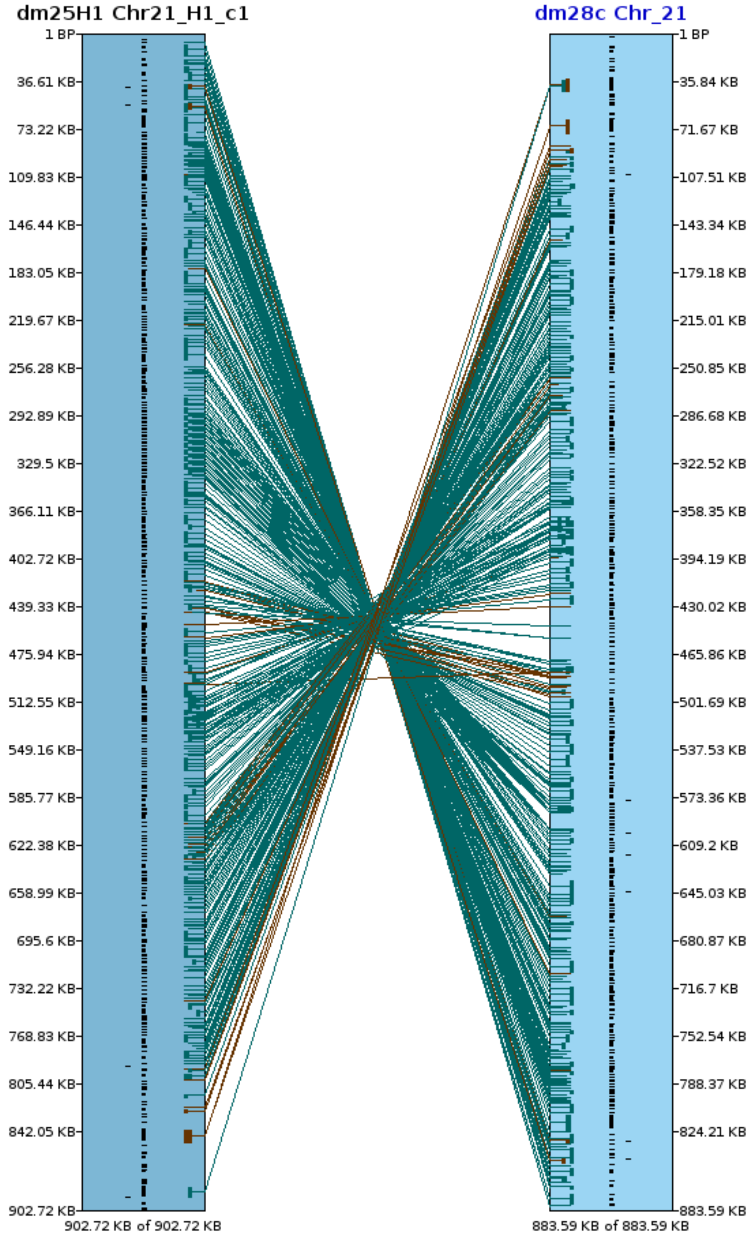
dm25H1 Chr21_H1_c1
dm28c Chr_21
1 BP
36.61 KB
73.22 KB
109.83 KB
146.44 KB
183.05 KB
219.67 KB
256.28 KB
292.89 KB
329.5 KB
366.11 KB
402.72 KB
439.33 KB
475.94 KB
512.55 KB
549.16 KB
585.77 KB
622.38 KB
658.99 KB
695.6 KB
732.22 KB
768.83 KB
805.44 KB
842.05 KB
902.72 KB
902.72 KB of 902.72 KB
1 BP
35.84 KB
71.67 KB
107.51 KB
143.34 KB
179.18 KB
215.01 KB
250.85 KB
286.68 KB
322.52 KB
358.35 KB
394.19 KB
430.02 KB
465.86 KB
501.69 KB
537.53 KB
573.36 KB
609.2 KB
645.03 KB
680.87 KB
716.7 KB
752.54 KB
788.37 KB
824.21 KB
883.59 KB
883.59 KB of 883.59 KB

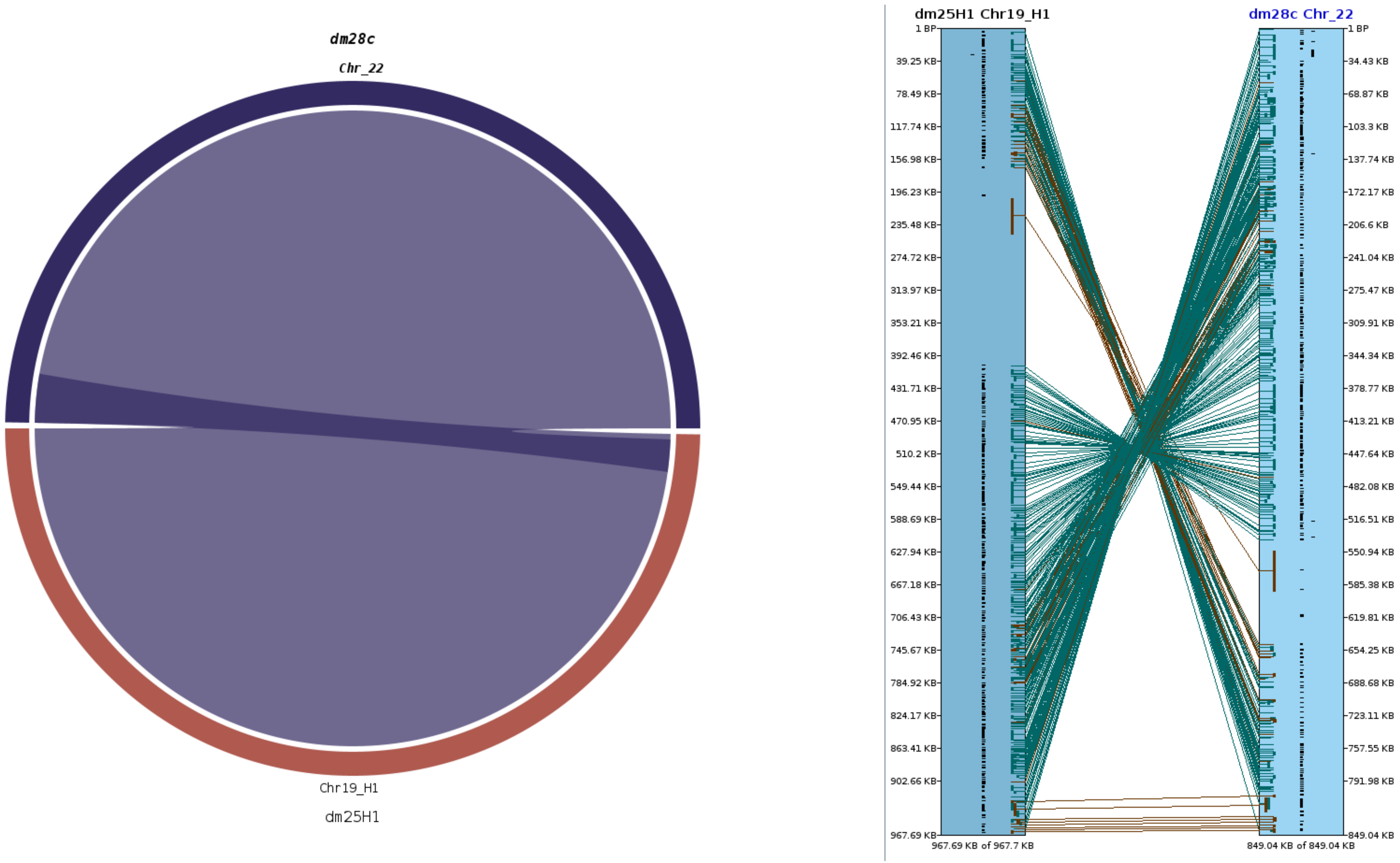

dm28c
Chr_22
Chr19_H1
dm25H1
dm25H1 Chr19_H1
dm28c Chr_22
1 BP
39.25 KB
78.49 KB
117.74 KB
156.98 KB
196.23 KB
235.48 KB
274.72 KB
313.97 KB
353.21 KB
392.46 KB
431.71 KB
470.95 KB
510.2 KB
549.44 KB
588.69 KB
627.94 KB
667.18 KB
706.43 KB
745.67 KB
784.92 KB
824.17 KB
863.41 KB
902.66 KB
967.69 KB
967.69 KB of 967.7 KB
1 BP
34.43 KB
68.87 KB
103.3 KB
137.74 KB
172.17 KB
206.6 KB
241.04 KB
275.47 KB
309.91 KB
344.34 KB
378.77 KB
413.21 KB
447.64 KB
482.08 KB
516.51 KB
550.94 KB
585.38 KB
619.81 KB
654.25 KB
688.68 KB
723.11 KB
757.55 KB
791.98 KB
849.04 KB
849.04 KB of 849.04 KB

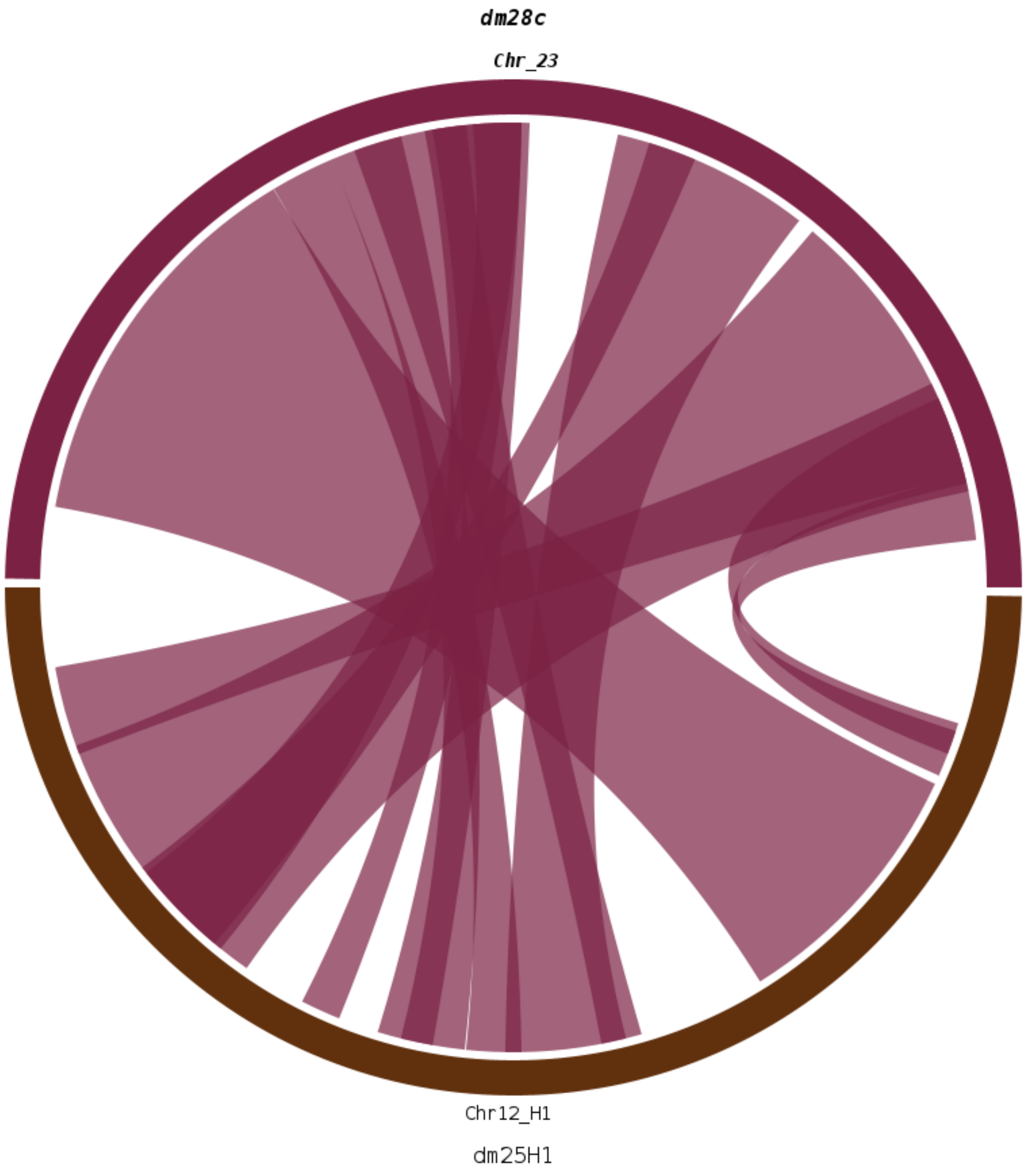
dm28c
Chr_23
Chr12_H1
dm25H1

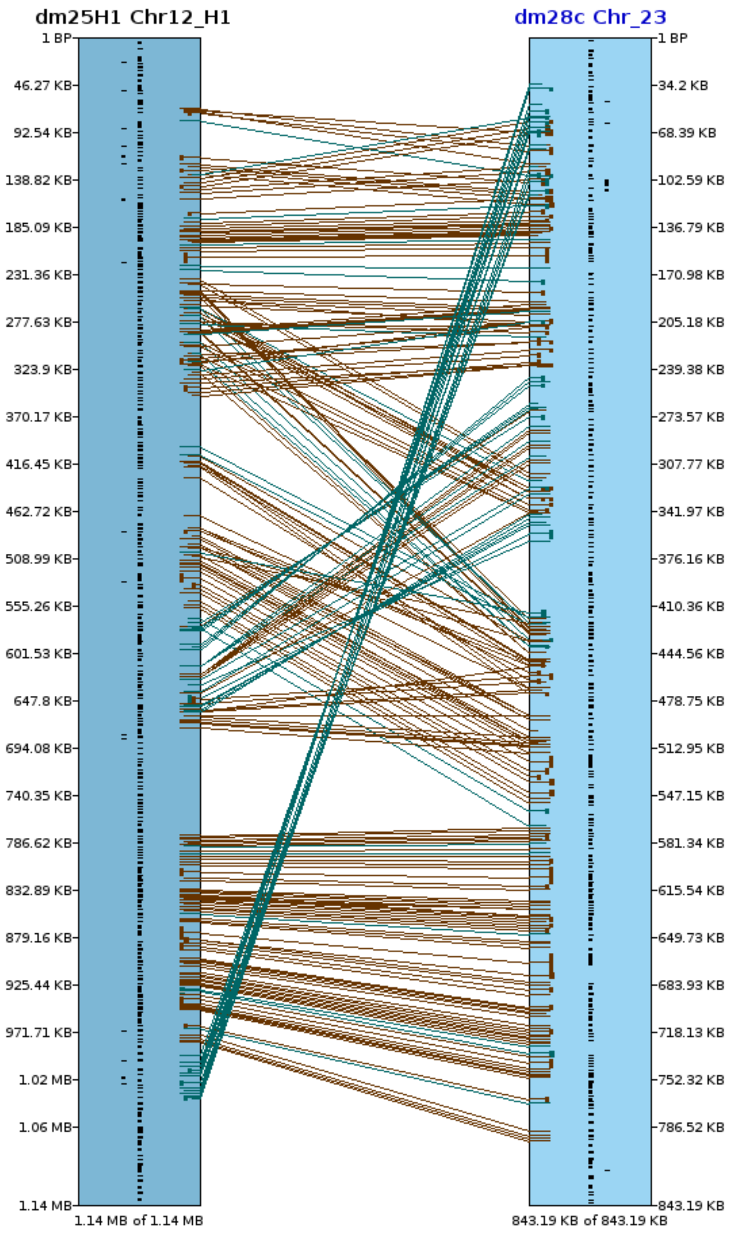
dm25H1 Chr12_H1
dm28c Chr_23
1 BP
46.27 KB
92.54 KB
138.82 KB
185.09 KB
231.36 KB
277.63 KB
323.9 KB
370.17 KB
416.45 KB
462.72 KB
508.99 KB
555.26 KB
601.53 KB
647.8 KB
694.08 KB
740.35 KB
786.62 KB
832.89 KB
879.16 KB
925.44 KB
971.71 KB
1.02 MB
1.06 MB
1.14 MB
1.14 MB of 1.14 MB
1 BP
34.2 KB
68.39 KB
102.59 KB
136.79 KB
170.98 KB
205.18 KB
239.38 KB
273.57 KB
307.77 KB
341.97 KB
376.16 KB
410.36 KB
444.56 KB
478.75 KB
512.95 KB
547.15 KB
581.34 KB
615.54 KB
649.73 KB
683.93 KB
718.13 KB
752.32 KB
786.52 KB
843.19 KB
843.19 KB of 843.19 KB

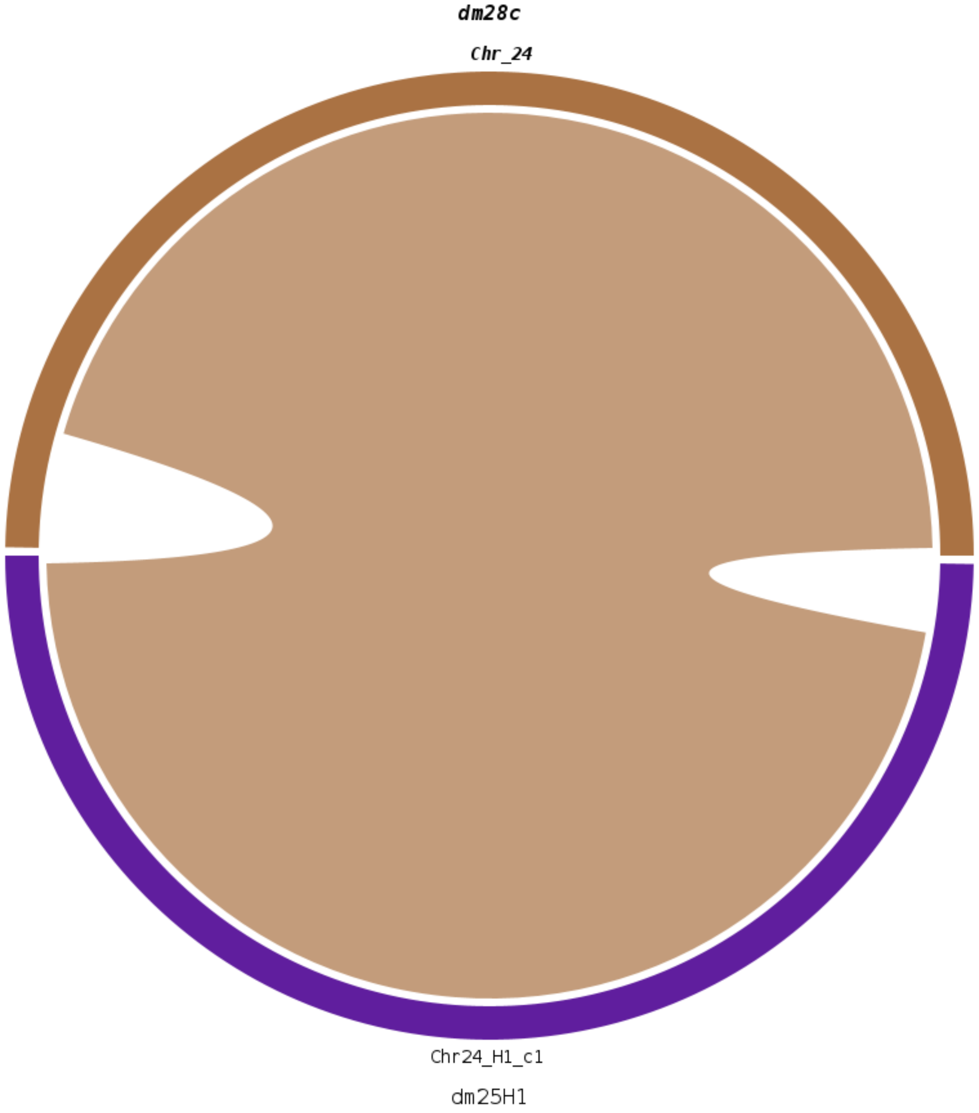
dm28c
Chr_24
Chr24_H1_c1
dm25H1

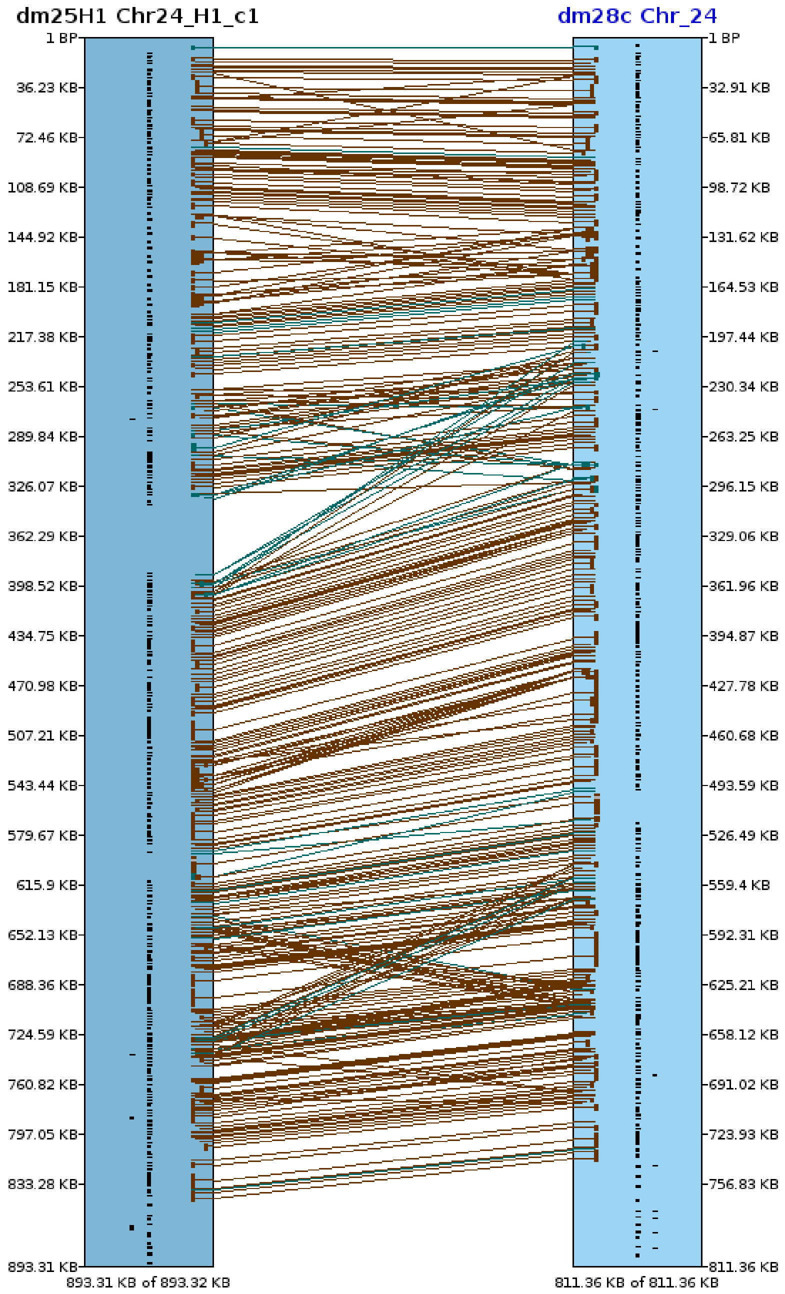
dm25H1 Chr24_H1_c1
dm28c Chr_24
1 BP
36.23 KB
72.46 KB
108.69 KB
144.92 KB
181.15 KB
217.38 KB
253.61 KB
289.84 KB
326.07 KB
362.29 KB
398.52 KB
434.75 KB
470.98 KB
507.21 KB
543.44 KB
579.67 KB
615.9 KB
652.13 KB
688.36 KB
724.59 KB
760.82 KB
797.05 KB
833.28 KB
893.31 KB
1 BP
32.91 KB
65.81 KB
98.72 KB
131.62 KB
164.53 KB
197.44 KB
230.34 KB
263.25 KB
296.15 KB
329.06 KB
361.96 KB
394.87 KB
427.78 KB
460.68 KB
493.59 KB
526.49 KB
559.4 KB
592.31 KB
625.21 KB
658.12 KB
691.02 KB
723.93 KB
756.83 KB
811.36 KB
893.31 KB of 893.32 KB
811.36 KB of 811.36 KB

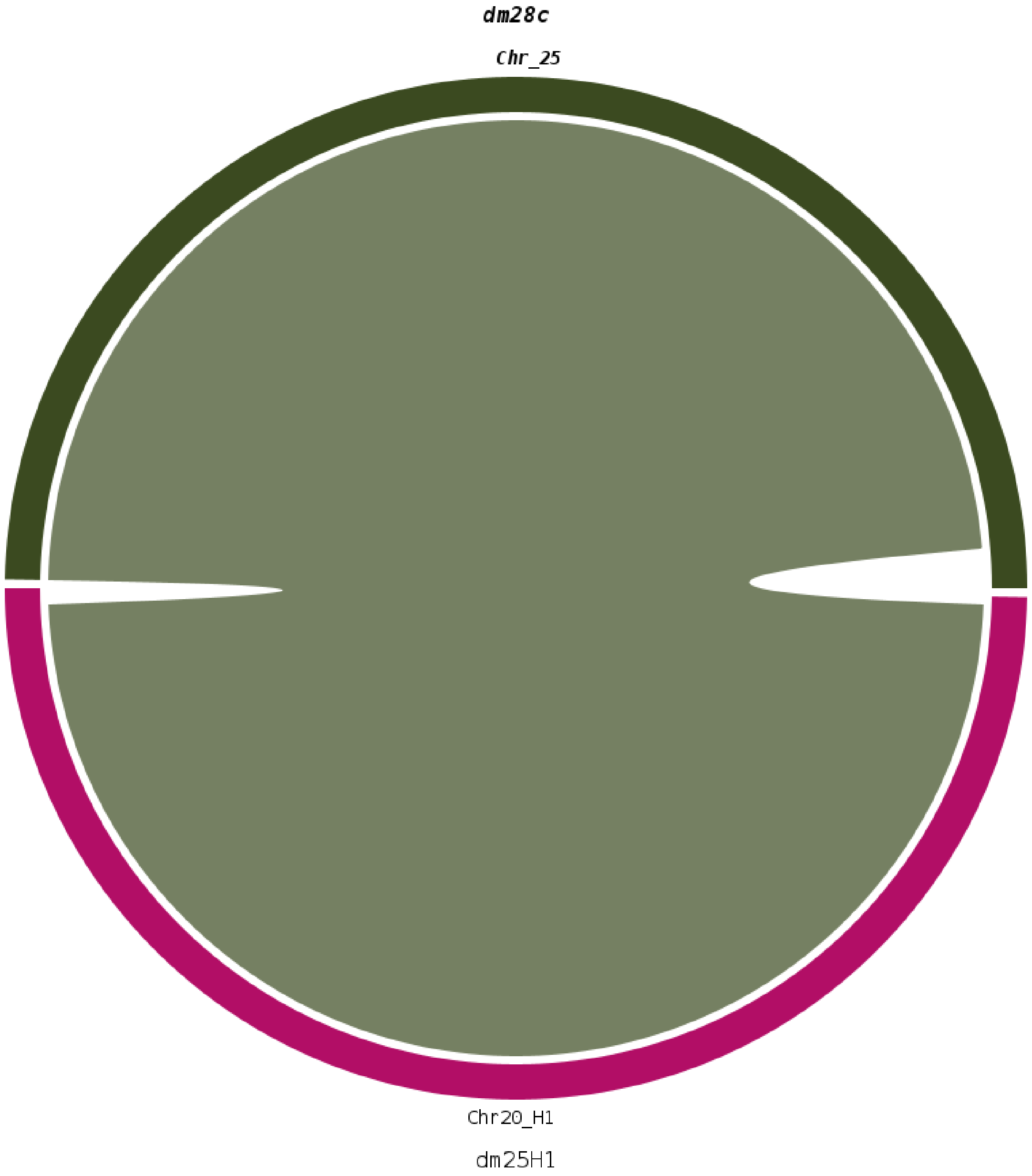
dm28c
Chr_25
Chr20_H1
dm25H1

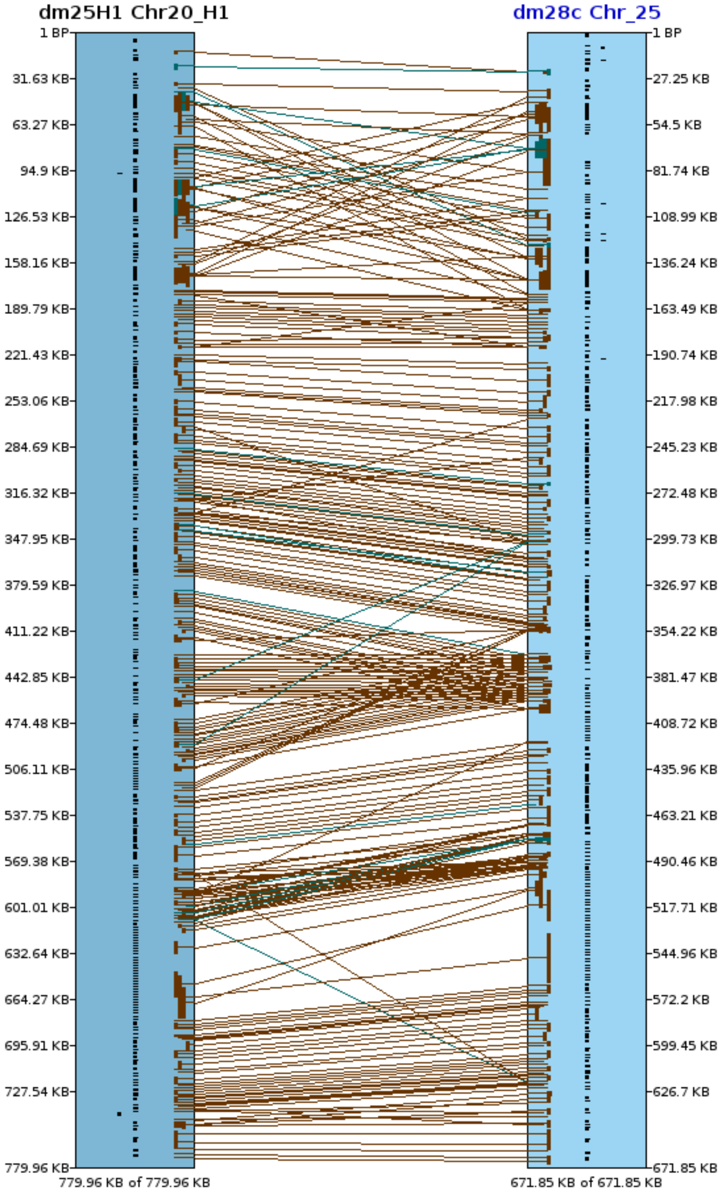
dm25H1 Chr20_H1
dm28c Chr_25
1 BP
31.63 KB
63.27 KB
94.9 KB
126.53 KB
158.16 KB
189.79 KB
221.43 KB
253.06 KB
284.69 KB
316.32 KB
347.95 KB
379.59 KB
411.22 KB
442.85 KB
474.48 KB
506.11 KB
537.75 KB
569.38 KB
601.01 KB
632.64 KB
664.27 KB
695.91 KB
727.54 KB
779.96 KB
779.96 KB of 779.96 KB
1 BP
27.25 KB
54.5 KB
81.74 KB
108.99 KB
136.24 KB
163.49 KB
190.74 KB
217.98 KB
245.23 KB
272.48 KB
299.73 KB
326.97 KB
354.22 KB
381.47 KB
408.72 KB
435.96 KB
463.21 KB
490.46 KB
517.71 KB
544.96 KB
572.2 KB
599.45 KB
626.7 KB
671.85 KB
671.85 KB of 671.85 KB

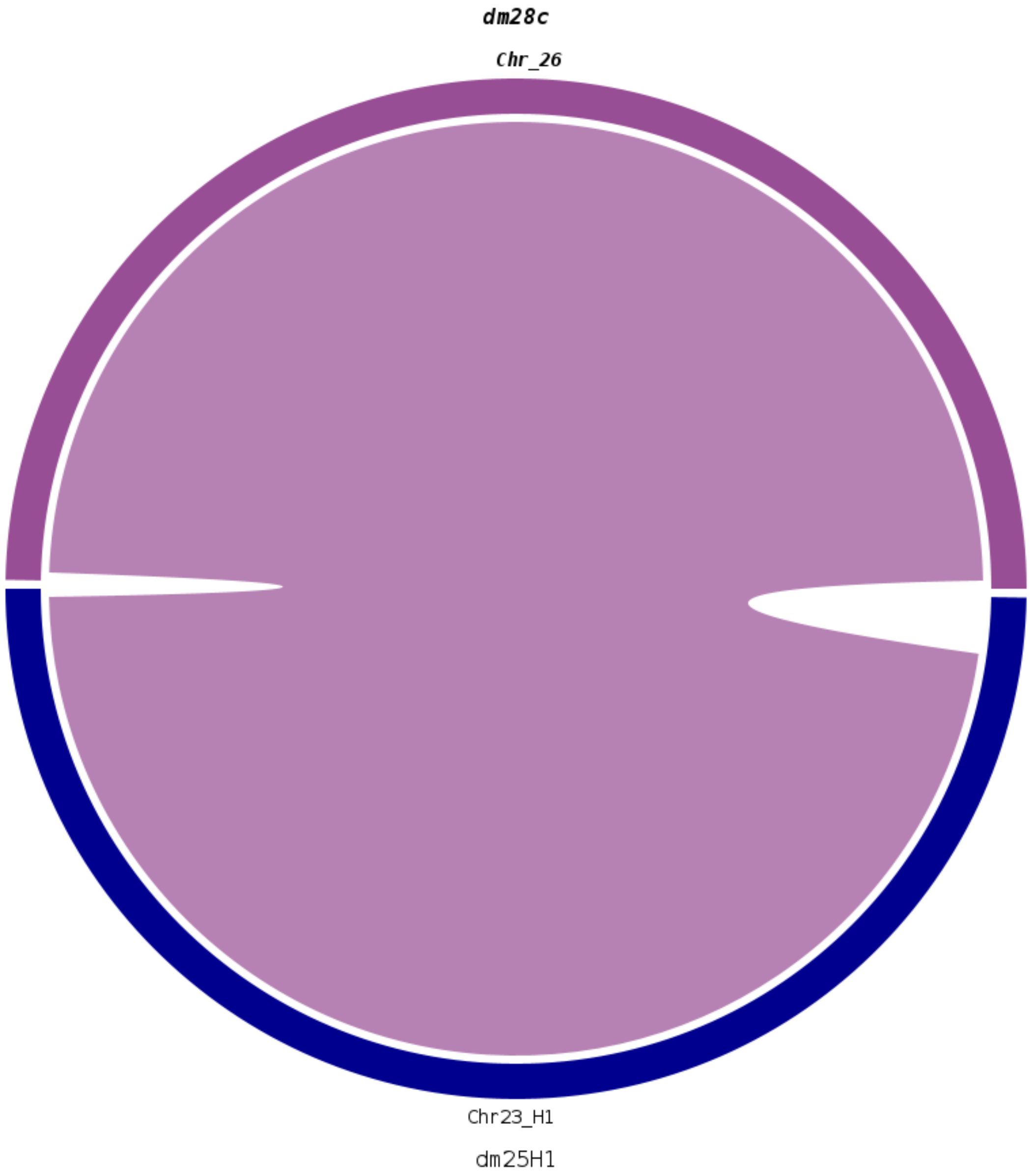
dm28c
Chr_26
Chr23_H1
dm25H1

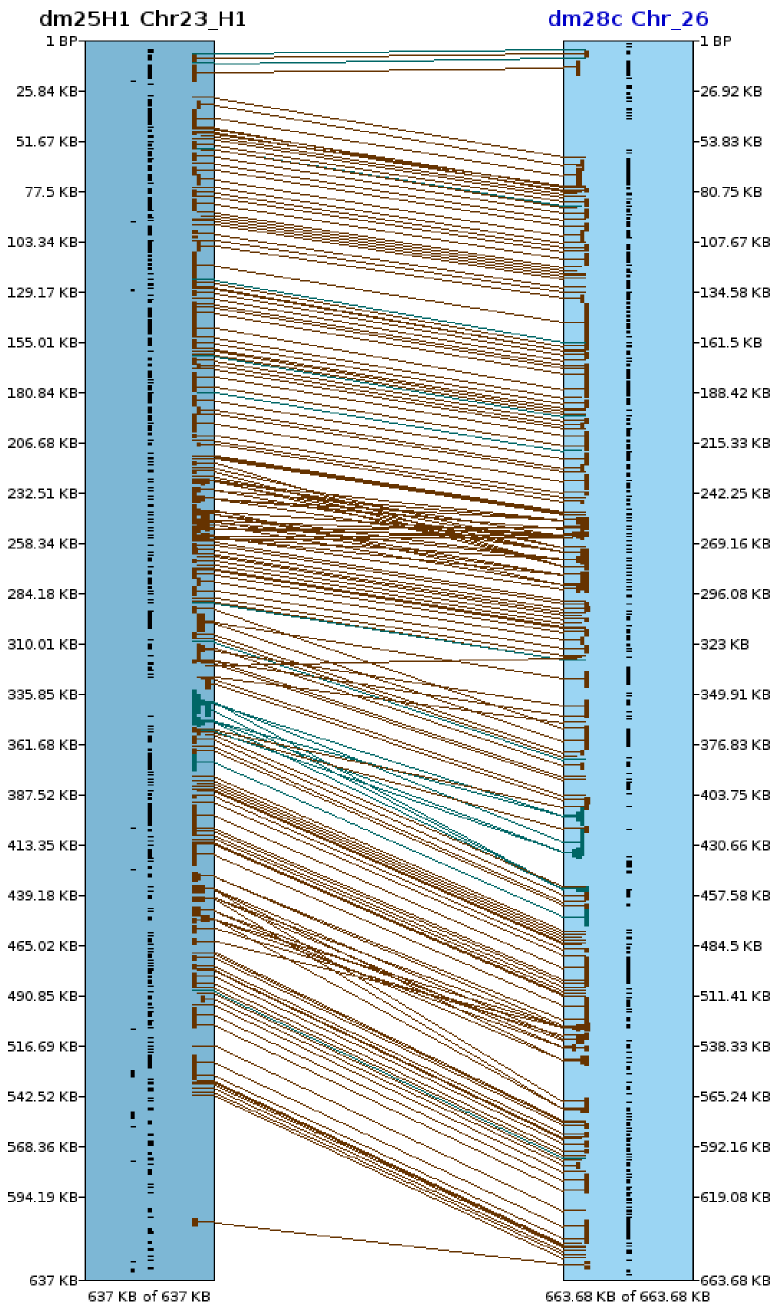
dm25H1 Chr23_H1
dm28c Chr_26
1 BP
25.84 KB
51.67 KB
77.5 KB
103.34 KB
129.17 KB
155.01 KB
180.84 KB
206.68 KB
232.51 KB
258.34 KB
284.18 KB
310.01 KB
335.85 KB
361.68 KB
387.52 KB
413.35 KB
439.18 KB
465.02 KB
490.85 KB
516.69 KB
542.52 KB
568.36 KB
594.19 KB
637 KB
637 KB of 637 KB
1 BP
26.92 KB
53.83 KB
80.75 KB
107.67 KB
134.58 KB
161.5 KB
188.42 KB
215.33 KB
242.25 KB
269.16 KB
296.08 KB
323 KB
349.91 KB
376.83 KB
403.75 KB
430.66 KB
457.58 KB
484.5 KB
511.41 KB
538.33 KB
565.24 KB
592.16 KB
619.08 KB
663.68 KB
663.68 KB of 663.68 KB

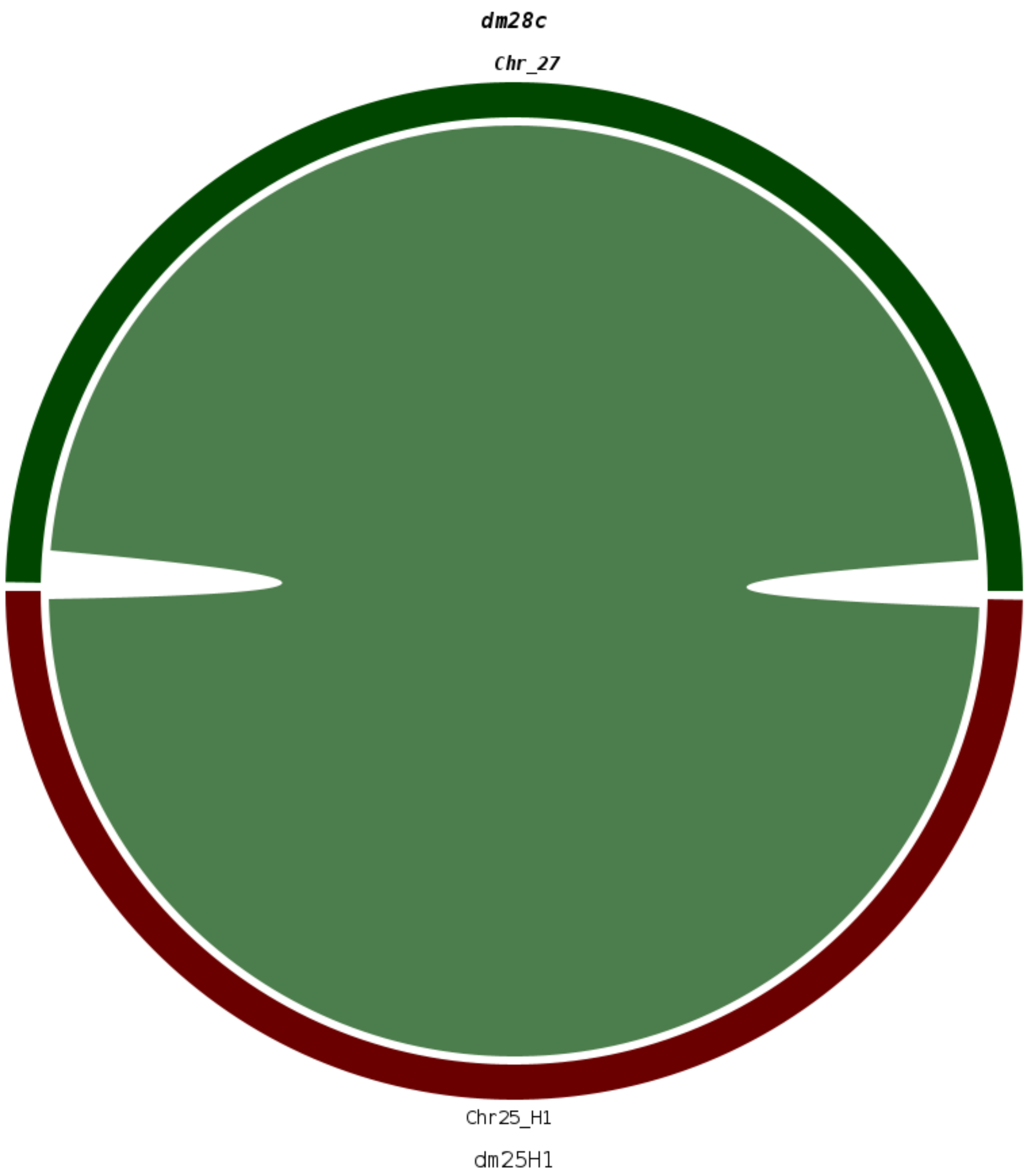
dm28c
Chr_27
Chr25_H1
dm25H1

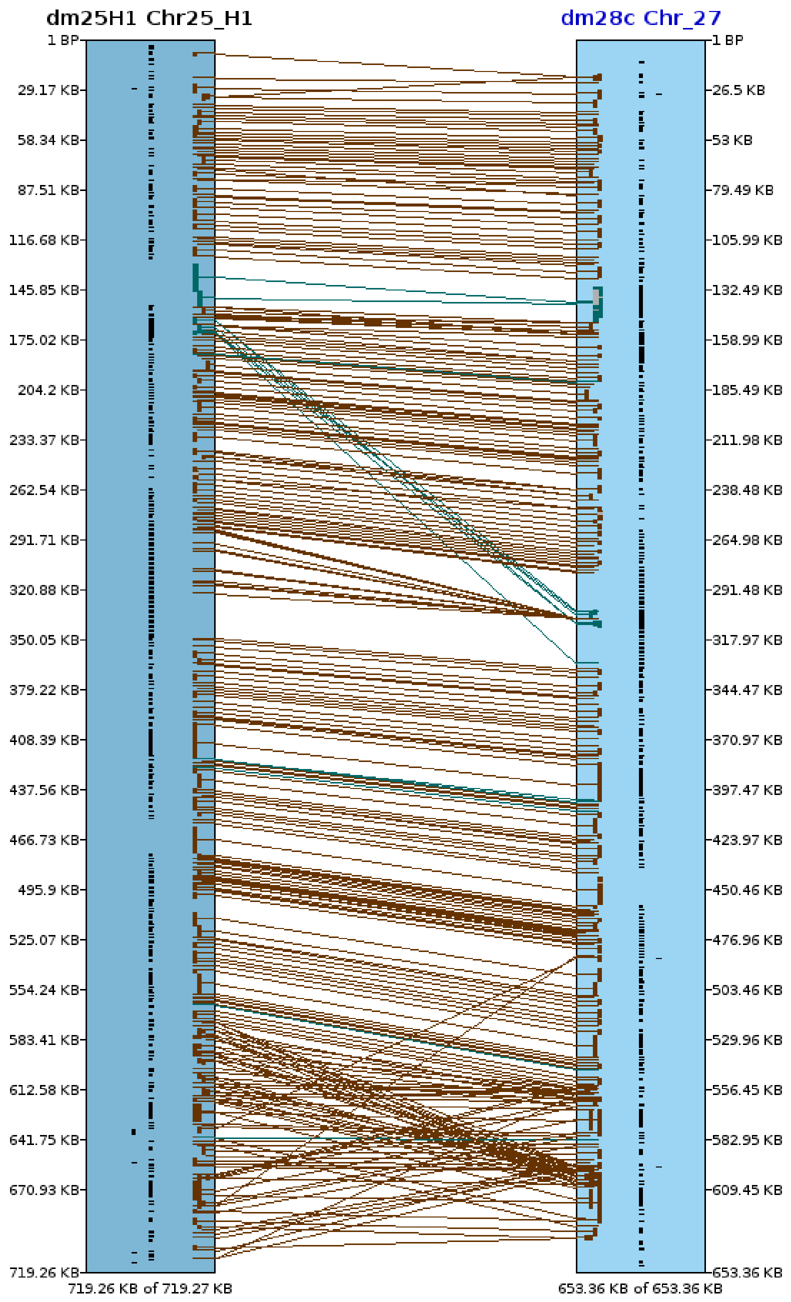
dm25H1 Chr25_H1
dm28c Chr_27
1 BP
29.17 KB
58.34 KB
87.51 KB
116.68 KB
145.85 KB
175.02 KB
204.2 KB
233.37 KB
262.54 KB
291.71 KB
320.88 KB
350.05 KB
379.22 KB
408.39 KB
437.56 KB
466.73 KB
495.9 KB
525.07 KB
554.24 KB
583.41 KB
612.58 KB
641.75 KB
670.93 KB
719.26 KB
719.26 KB of 719.27 KB
1 BP
26.5 KB
53 KB
79.49 KB
105.99 KB
132.49 KB
158.99 KB
185.49 KB
211.98 KB
238.48 KB
264.98 KB
291.48 KB
317.97 KB
344.47 KB
370.97 KB
397.47 KB
423.97 KB
450.46 KB
476.96 KB
503.46 KB
529.96 KB
556.45 KB
582.95 KB
609.45 KB
653.36 KB
653.36 KB of 653.36 KB

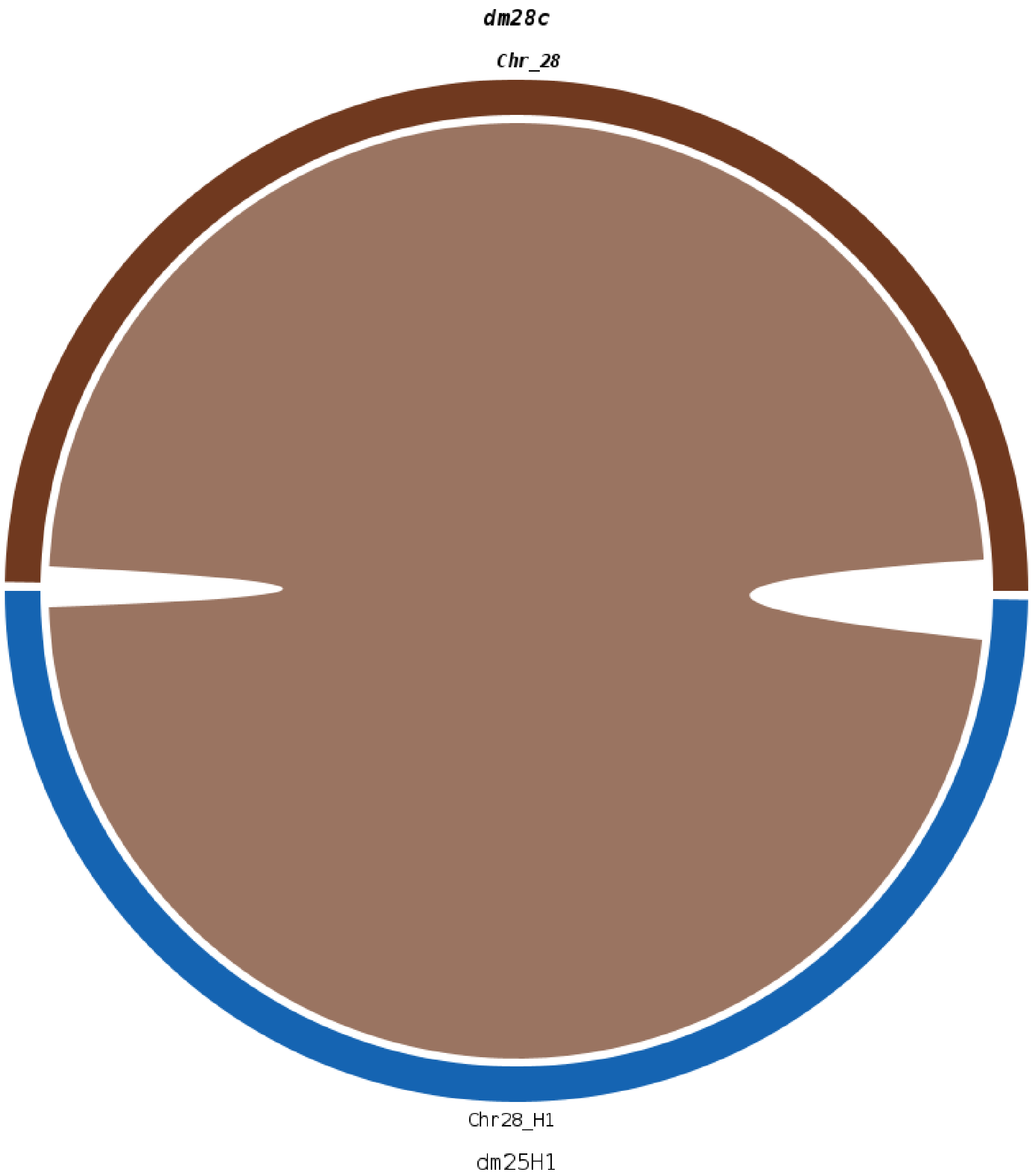
dm28c
Chr_28
Chr28_H1
dm25H1

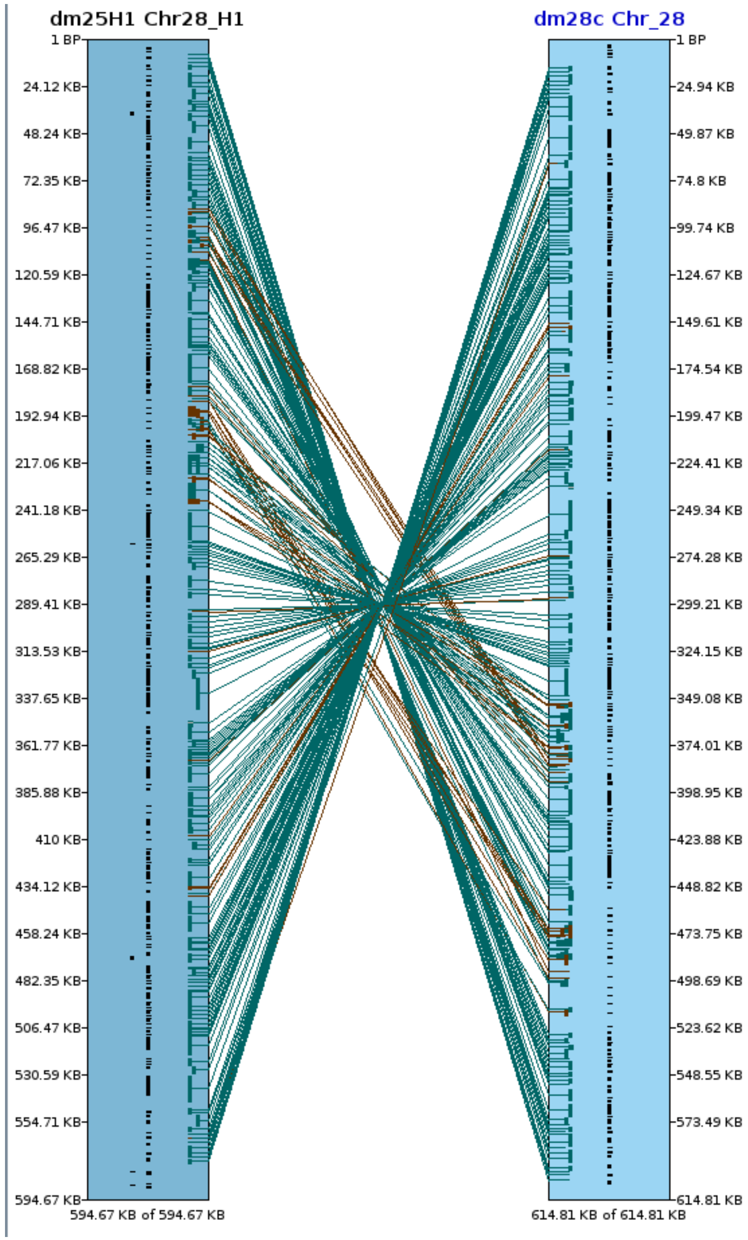
dm25H1 Chr28_H1
dm28c Chr_28
1 BP
24.12 KB
48.24 KB
72.35 KB
96.47 KB
120.59 KB
144.71 KB
168.82 KB
192.94 KB
217.06 KB
241.18 KB
265.29 KB
289.41 KB
313.53 KB
337.65 KB
361.77 KB
385.88 KB
410 KB
434.12 KB
458.24 KB
482.35 KB
506.47 KB
530.59 KB
554.71 KB
594.67 KB
594.67 KB of 594.67 KB
1 BP
24.94 KB
49.87 KB
74.8 KB
99.74 KB
124.67 KB
149.61 KB
174.54 KB
199.47 KB
224.41 KB
249.34 KB
274.28 KB
299.21 KB
324.15 KB
349.08 KB
374.01 KB
398.95 KB
423.88 KB
448.82 KB
473.75 KB
498.69 KB
523.62 KB
548.55 KB
573.49 KB
614.81 KB
614.81 KB of 614.81 KB

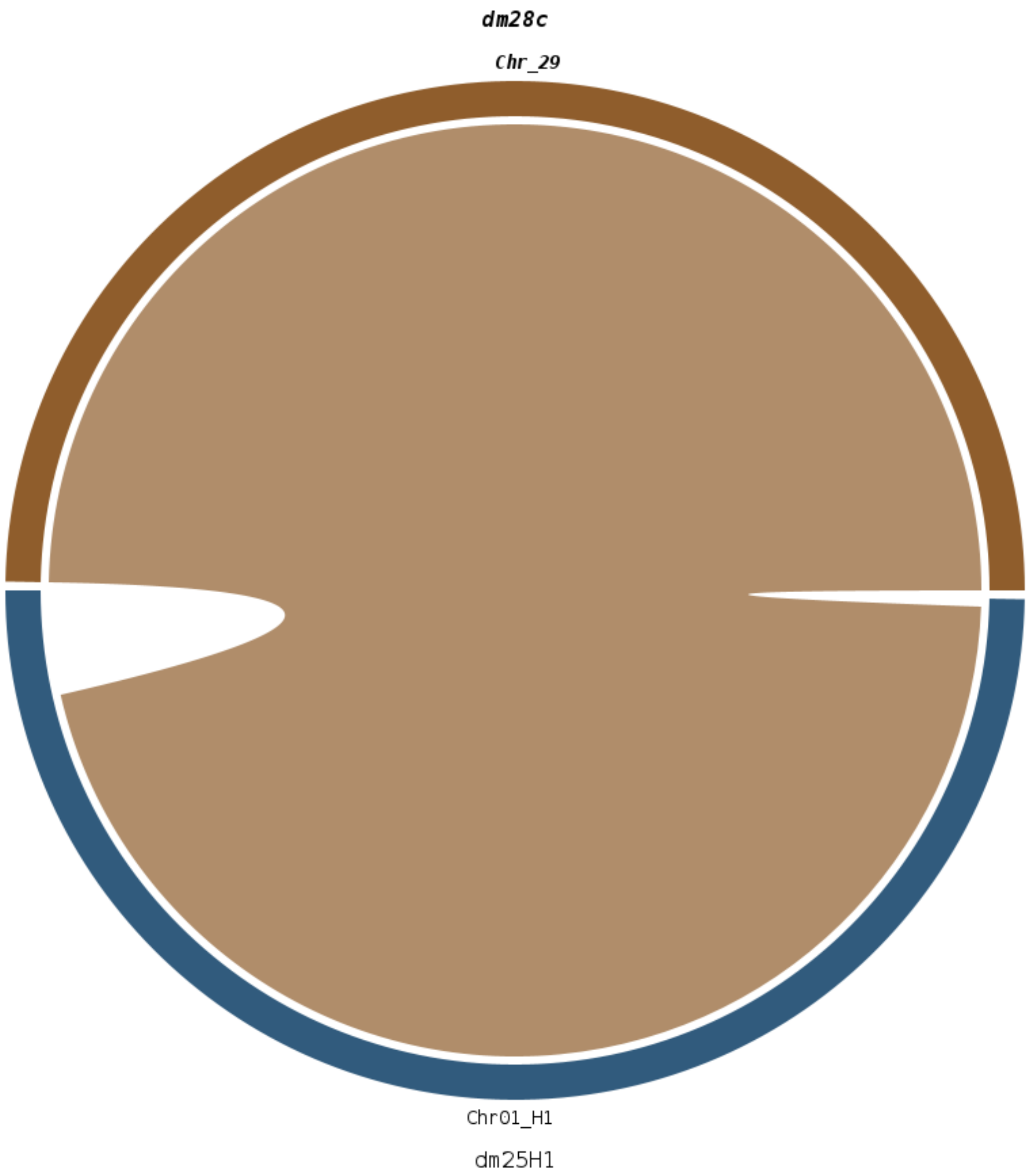

dm28c
Chr_29
Chr01_H1
dm25H1

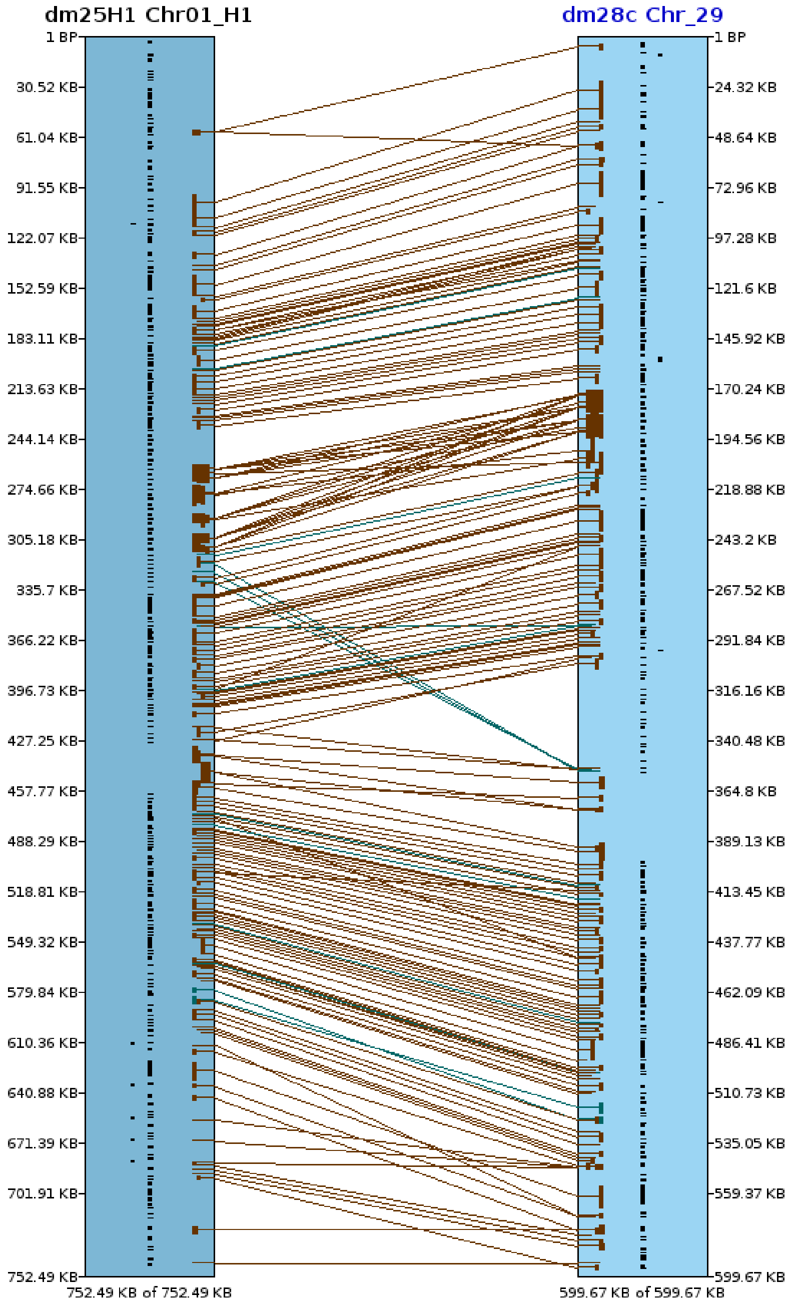

dm25H1 Chr01_H1
dm28c Chr_29
1 BP
30.52 KB
61.04 KB
91.55 KB
122.07 KB
152.59 KB
183.11 KB
213.63 KB
244.14 KB
274.66 KB
305.18 KB
335.7 KB
366.22 KB
396.73 KB
427.25 KB
457.77 KB
488.29 KB
518.81 KB
549.32 KB
579.84 KB
610.36 KB
640.88 KB
671.39 KB
701.91 KB
752.49 KB
752.49 KB of 752.49 KB
1 BP
24.32 KB
48.64 KB
72.96 KB
97.28 KB
121.6 KB
145.92 KB
170.24 KB
194.56 KB
218.88 KB
243.2 KB
267.52 KB
291.84 KB
316.16 KB
340.48 KB
364.8 KB
389.13 KB
413.45 KB
437.77 KB
462.09 KB
486.41 KB
510.73 KB
535.05 KB
559.37 KB
599.67 KB
599.67 KB of 599.67 KB

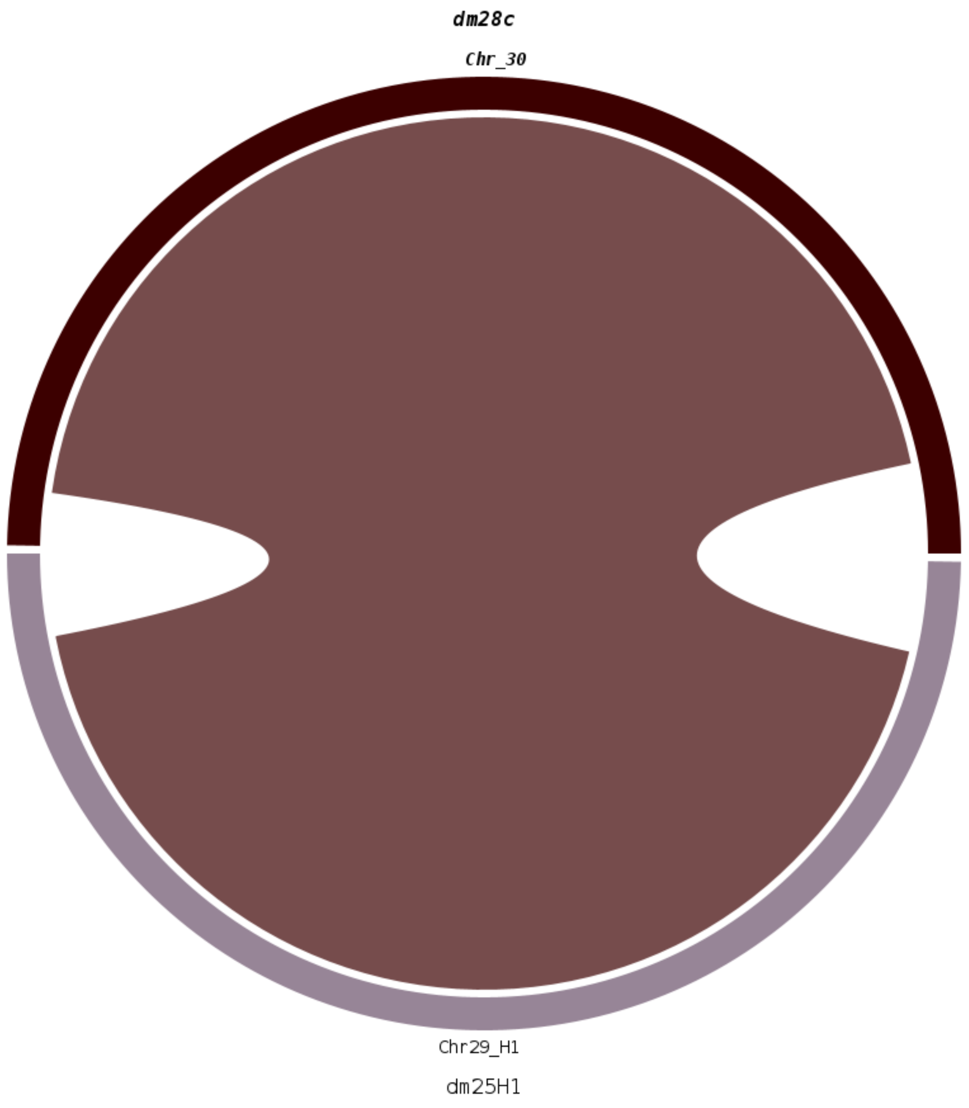
dm28c
Chr_30
Chr29_H1
dm25H1

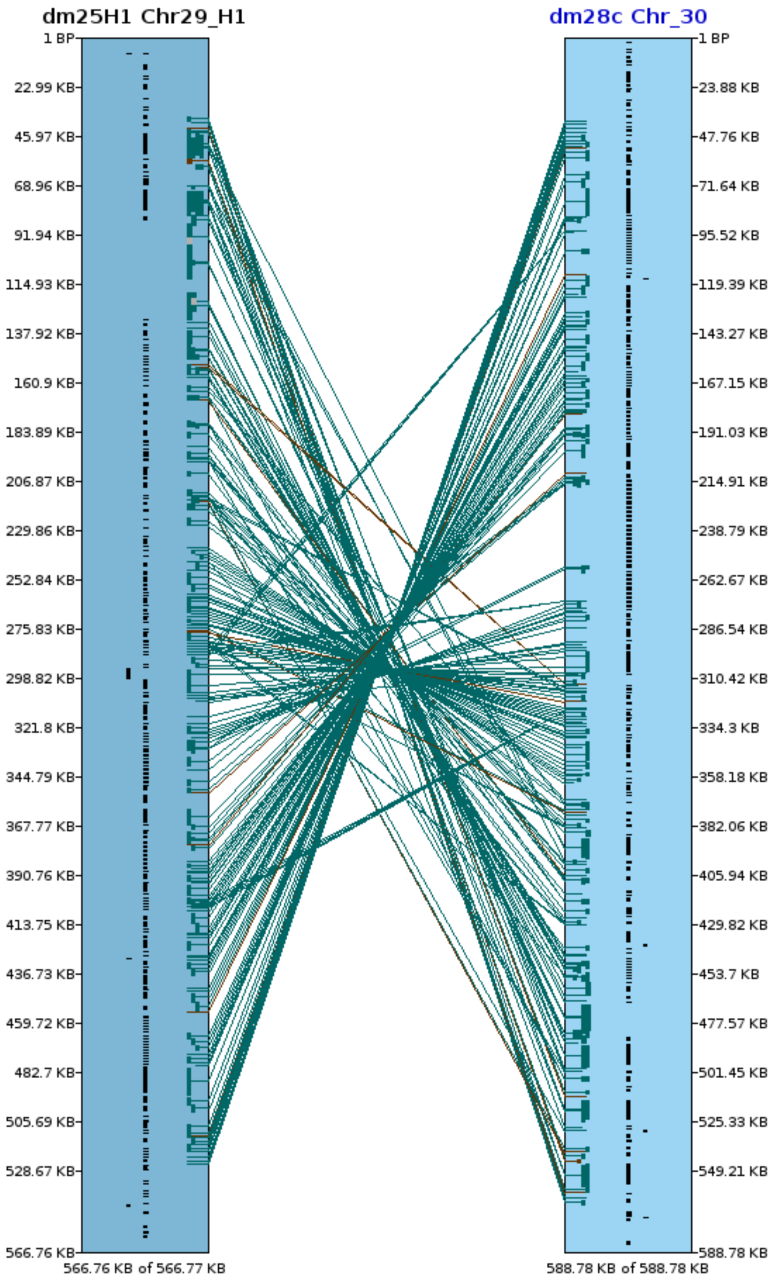
dm25H1 Chr29_H1
dm28c Chr_30
1 BP
22.99 KB
45.97 KB
68.96 KB
91.94 KB
114.93 KB
137.92 KB
160.9 KB
183.89 KB
206.87 KB
229.86 KB
252.84 KB
275.83 KB
298.82 KB
321.8 KB
344.79 KB
367.77 KB
390.76 KB
413.75 KB
436.73 KB
459.72 KB
482.7 KB
505.69 KB
528.67 KB
566.76 KB
566.76 KB of 566.77 KB
1 BP
23.88 KB
47.76 KB
71.64 KB
95.52 KB
119.39 KB
143.27 KB
167.15 KB
191.03 KB
214.91 KB
238.79 KB
262.67 KB
286.54 KB
310.42 KB
334.3 KB
358.18 KB
382.06 KB
405.94 KB
429.82 KB
453.7 KB
477.57 KB
501.45 KB
525.33 KB
549.21 KB
588.78 KB
588.78 KB of 588.78 KB

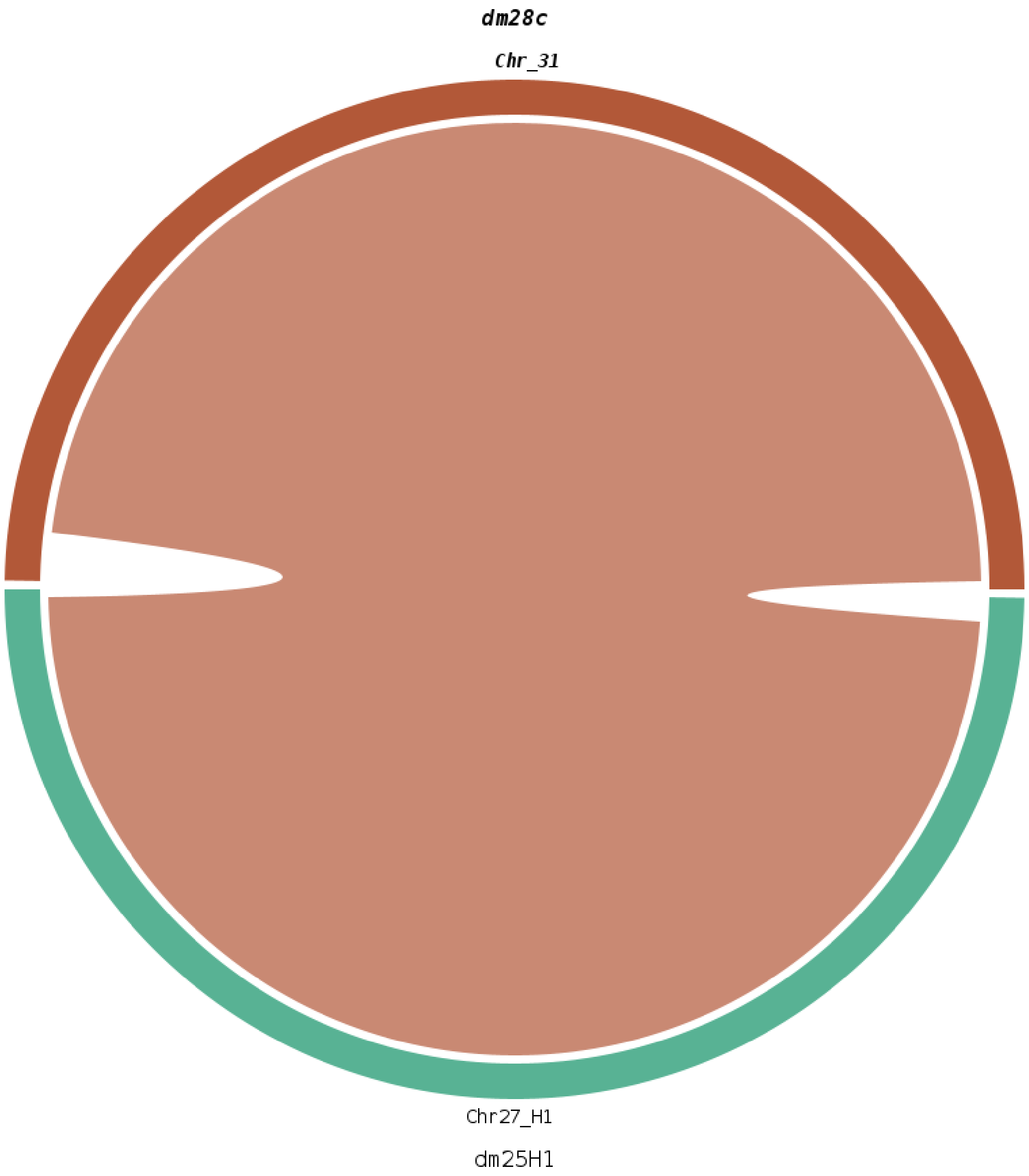
dm28c
Chr_31
Chr27_H1
dm25H1

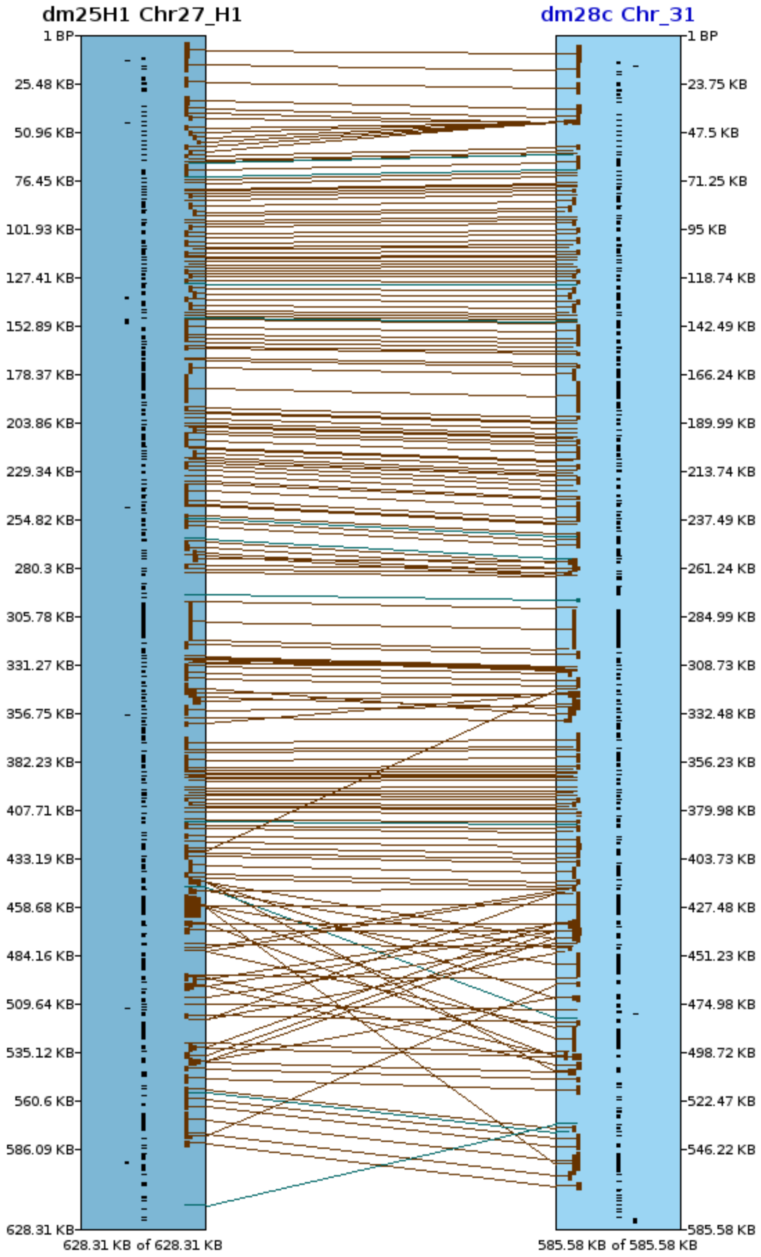
dm25H1 Chr27_H1
dm28c Chr_31
1 BP
25.48 KB
50.96 KB
76.45 KB
101.93 KB
127.41 KB
152.89 KB
178.37 KB
203.86 KB
229.34 KB
254.82 KB
280.3 KB
305.78 KB
331.27 KB
356.75 KB
382.23 KB
407.71 KB
433.19 KB
458.68 KB
484.16 KB
509.64 KB
535.12 KB
560.6 KB
586.09 KB
628.31 KB
628.31 KB of 628.31 KB
1 BP
23.75 KB
47.5 KB
71.25 KB
95 KB
118.74 KB
142.49 KB
166.24 KB
189.99 KB
213.74 KB
237.49 KB
261.24 KB
284.99 KB
308.73 KB
332.48 KB
356.23 KB
379.98 KB
403.73 KB
427.48 KB
451.23 KB
474.98 KB
498.72 KB
522.47 KB
546.22 KB
585.58 KB
585.58 KB of 585.58 KB

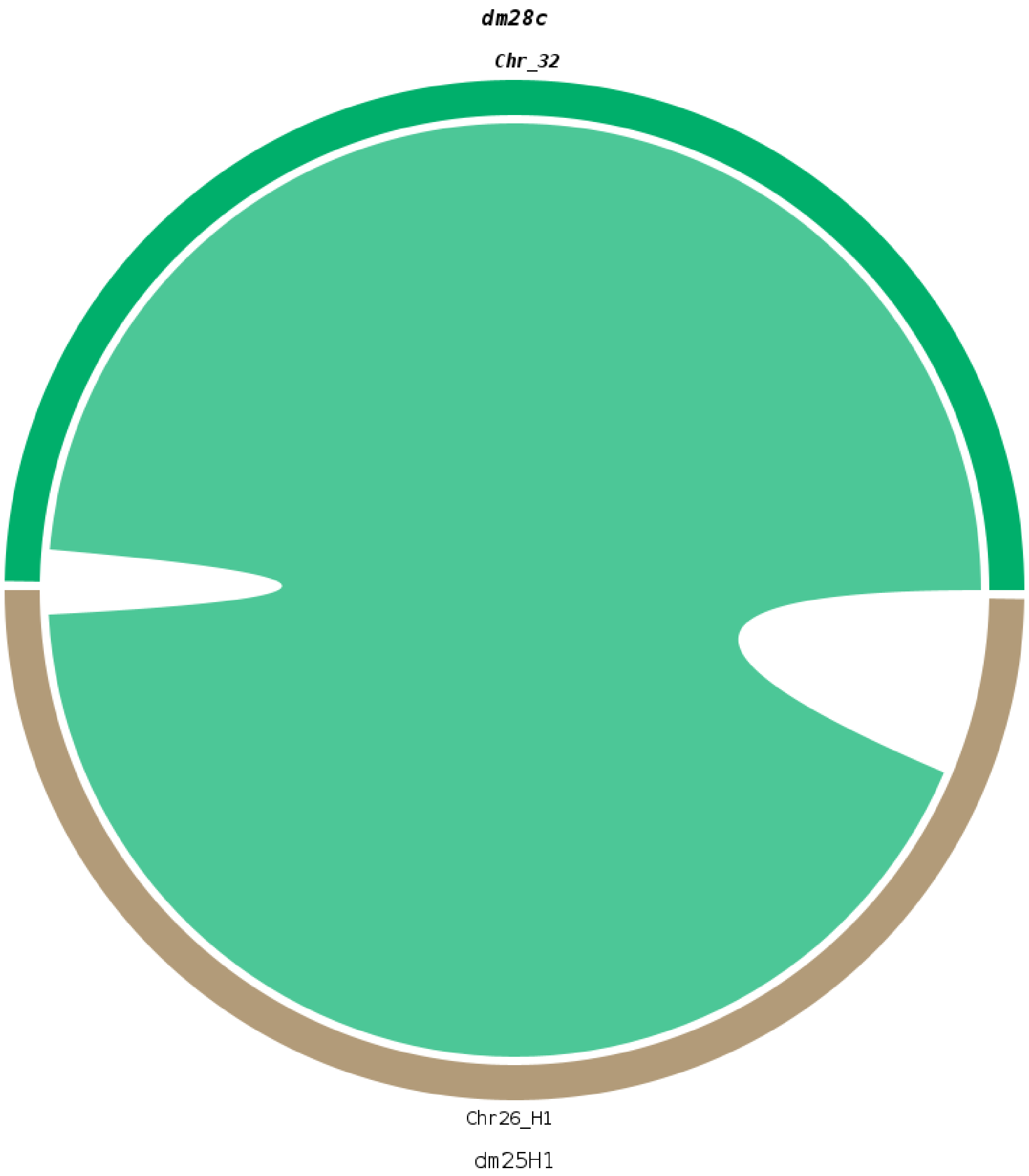
dm28c
Chr_32
Chr26_H1
dm25H1

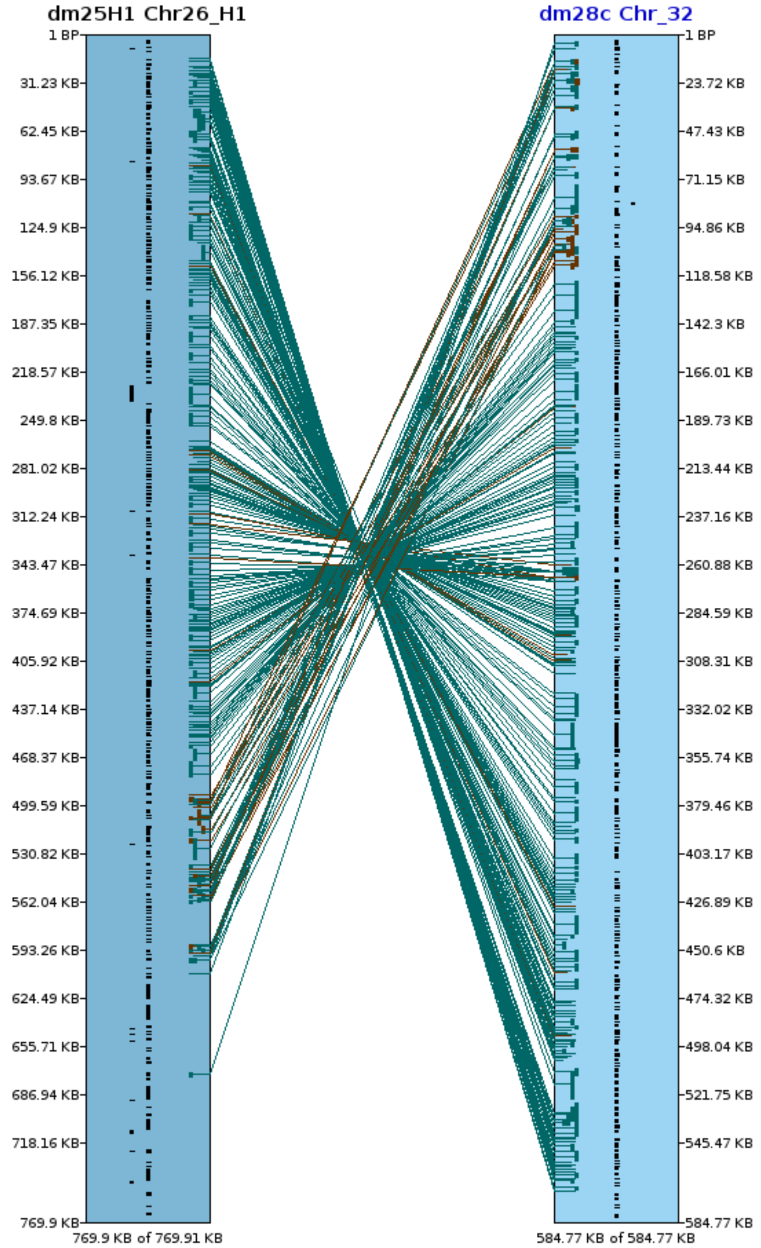
dm25H1 Chr26_H1
dm28c Chr_32
1 BP
31.23 KB
62.45 KB
93.67 KB
124.9 KB
156.12 KB
187.35 KB
218.57 KB
249.8 KB
281.02 KB
312.24 KB
343.47 KB
374.69 KB
405.92 KB
437.14 KB
468.37 KB
499.59 KB
530.82 KB
562.04 KB
593.26 KB
624.49 KB
655.71 KB
686.94 KB
718.16 KB
769.9 KB
769.9 KB of 769.91 KB
1 BP
23.72 KB
47.43 KB
71.15 KB
94.86 KB
118.58 KB
142.3 KB
166.01 KB
189.73 KB
213.44 KB
237.16 KB
260.88 KB
284.59 KB
308.31 KB
332.02 KB
355.74 KB
379.46 KB
403.17 KB
426.89 KB
450.6 KB
474.32 KB
498.04 KB
521.75 KB
545.47 KB
584.77 KB
584.77 KB of 584.77 KB
