# Supplementary material for: The complete genome of Trypanosoma cruzi reveals 32 chromosomes and three genomic compartments
Source: BMC Genomics. 2026 Jan 8;27:159. doi: 10.1186/s12864-025-12482-0 (PMC12879350; doi:10.1186/s12864-025-12482-0)

Supplementary Figure 12. Dm28c step-by-step assembly process:

PacBio HiFi reads (301047 raw reads)

↓ Hifiasm assembler

51 contigs (1 circular contig)

↓

50 linear contigs

↓ Telomere_finder.py

25 T2T contigs = chromosomes
25 contigs with one or non telomere

↓

Manual assembly steps:

- Comparison with T2T Dm25 (circos and dotplot)
- Dotplot analysis of contigs to be joined to detect overlap
- Dotplot analysis of contig joined
- Validation using new Sylvio genome

51 contigs (1 circular contig) → Contig 49c (50Kb) = Maxicircle sequence

Dotplot vs itself

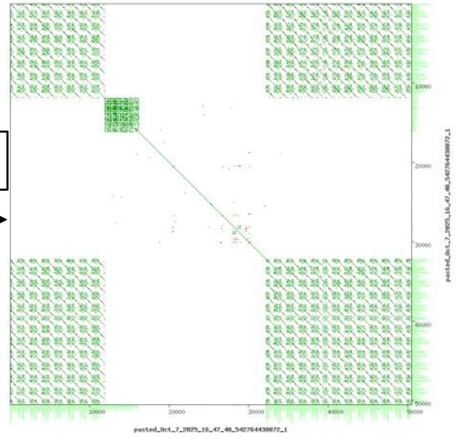

Dotplot vs Maxicircle Dm28c (PMC:34437557)

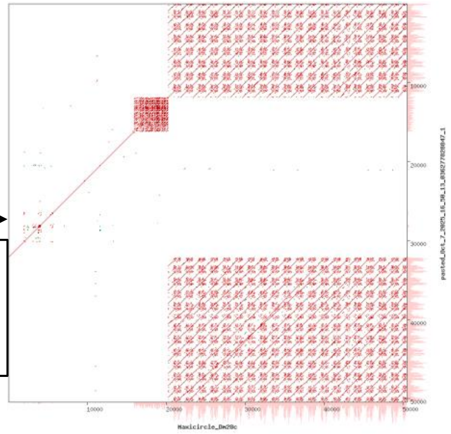

Dm28c step-by-step assembly process. Comparison with Dm25 strain and manual curation

25 contigs T2T: 18 have homologous chromsome assembly T2T in Dm25 strain	6 Contigs T2T in Dm25 used to join contigs in Dm28c strain:

Summary of results chromosome by chromosome:

- chr1 = Contigs 029l + 01l (Dm25 chr04 no T2T) *[1]
- **chr2 = contig 06l T2T (Dm25 chr02 T2T)**
- chr3 = contigs 046l + 033l (Chr09 Dm25 T2T) *[2]
- **chr4= contig 017l T2T** (contigs 025l and 28l haplotypic regions and **Dm25 Chr32 T2T**) &[1]
- **chr5= contig 047l T2T** (contig 024l haplotipic, disruptive)=**Dm25 Chr30 no T2T**
- **chr6= contig 08l T2T (Dm25 chr03 T2T)**
- **chr7= contig 015l (With 5' Telomere, correspond to Dm25 chr05 T2T –missing only 3' telomere región-)** *[3]
- **chr8= contig 03l T2T (Dm25 chr07 T2T)**
- **chr9= contig 022l T2T (Dm25 chr05 T2T,** contigs 50l y 51l haplotypes D) &[2]
- **chr10= contig 04l T2T** (Fragmented in contigs in Dm25 –chr22- and contig 30l haplotype D región)
- **chr11= contig 43l (Dm25 chr08 T2T)** *[4]
- **chr12= contig 19l T2T (Dm25 chr17 T2T)**
- **chr13= contig 37l T2T** (contig 32l haplotype, 2 contigs in Dm25 chr30) &[3]
- **chr14= contig 14l T2T** (Dm25 chr11 2 contigs)
- **chr15= contig 31l (Dm25 chr10 T2T)** *[5]
- **chr16= contig 07l T2T** (contigs 10l and 34l haplotypic regions D, Dm25 Chr31 fragmented) &[4]
- **chr17= contig 02l T2T (Dm25 chr08 T2T)**
- chr18= join contig 11l + 42l **(Dm25 chr14 T2T)** *[6]
- **chr19= contig 41l T2T (Dm25 chr15 T2T)**
- **chr20= contig 23l T2T (Dm25 chr13 T2T)**

- T2T in Dm28c and Dm25
- T2T only in Dm28c
- T2T only in Dm25
- Particular cases

* Manual assembled chromosomes
& Chromosomes with haplotipic contigs.

Dm28c step-by-step assembly process. Comparison with Dm25 strain and manual curation

25 contigs T2T: 18 have homologous chromsome assembly T2T in Dm25 strain

6 Contigs T2T in Dm25 used to join contigs in Dm28c strain:

Summary of results chromosome by chromosome:

- ● **chr21 = contig 12l T2T** (contig 09l haplotype, Dm25 chr21 fragmented) &5
- ● **chr22 = contig 40l T2T** (contig 18l haplotype, **Dm25 chr19 T2T**) &6
- ● **chr23 = contig 26l T2T (Dm25 chr12 T2T)**
- ● **chr24 = contig 27l T2T** (Dm25 chr 24 Dm25 fragmented) &7
- ● **chr25 = contig 20l T2T (Dm25 chr20 T2T)**
- ● **chr26 = contig 36l T2T** (contig 16l haplotype, **Dm25 chr23 T2T**) &8
- ● **chr27 = contig 45l T2T (Dm25 chr25 T2T)**
- ● **chr28 = contig 35l T2T (Dm25 chr28 T2T)**
- ● **chr29 = contig 05l T2T (Dm25 chr01 T2T)**
- ● chr30 = contig 39l T2T (Dm25 chr29 Dm25, missing only 5' telomere) *7
- ● **chr31 = contig 44l T2T (Dm25 chr27 T2T)**
- ● **chr32 = contig 13l T2T (Dm25 chr26 T2T)**
- ● **Maxicircle=contig 49c (complete)**

● T2T in Dm28c and Dm25
● T2T only in Dm28c
● T2T only in Dm25
● Particular cases

* Manual assembled chromosomes
& Chromosomes with haplotipic contigs.

Manual assembly :

chr1 = Contigs 029l + 01l (Dm25 chr04 no T2T) *[1]

1. Circos Dm25 vs all contigs Dm28c: contig 029 with telomere 5’ and contig 01 with telomere 3’

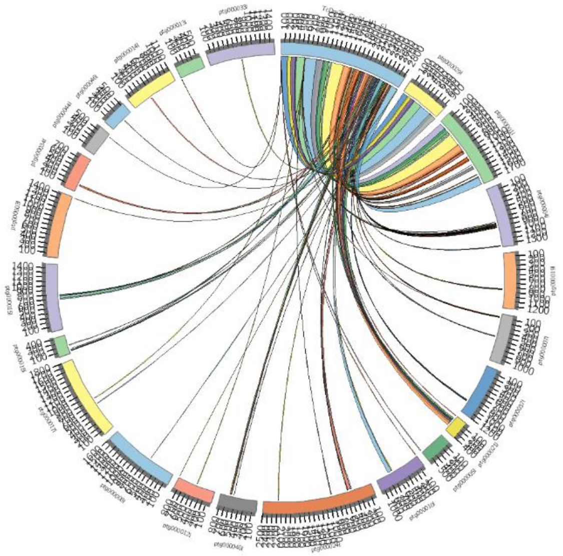

2. Dotplot analysis:
contigs Dm28c 029 and 01 vs Chr04 Dm25

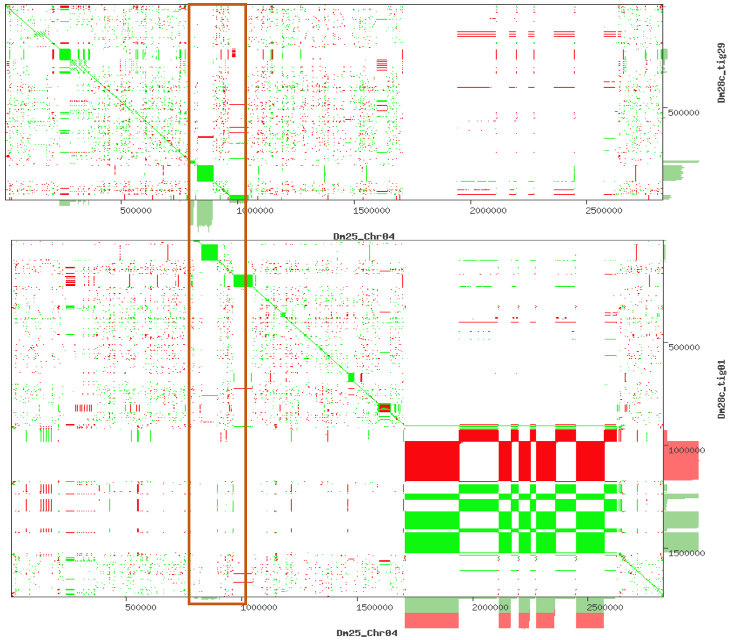

3. Dotplot analysis tig 01 vs tig 29

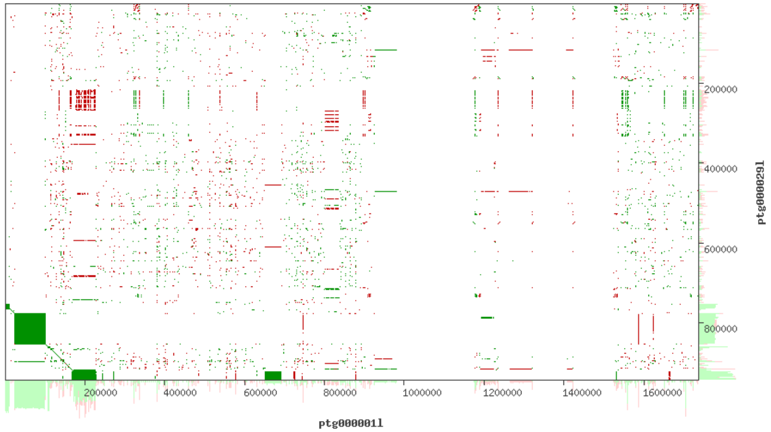

4. Dotplot analysis assembled chr1 vs chr 04 Dm25

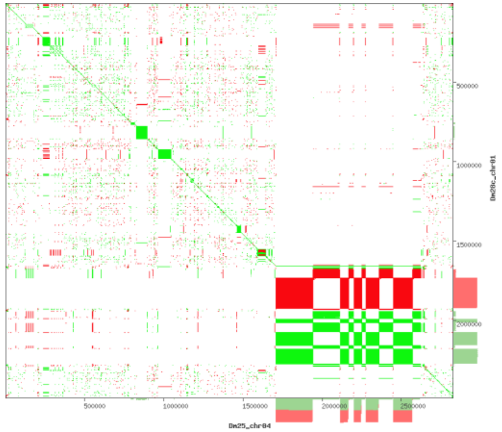

5. Validation using new 2025 Sylvio T2T

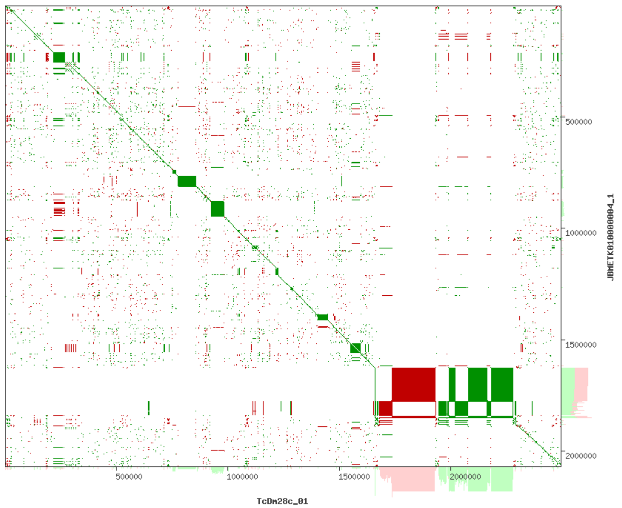

Manual assembly:

chr3 = contigs 046l + 033l (Chr09 Dm25 T2T) *2

1. Circos Dm25 vs all contigs Dm28c: tig046l with telomere 5’ and tig033l with telomere 3’

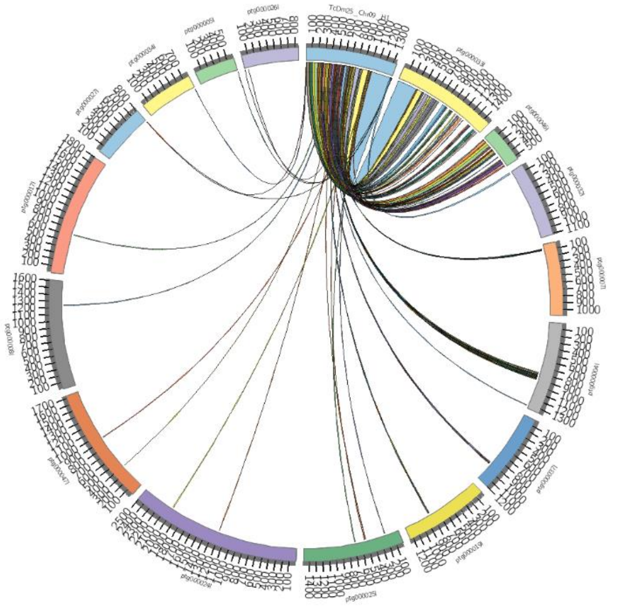

2. Dotplot analysis:
contigs Dm28c tig046 and 033 vs Chr09 Dm25

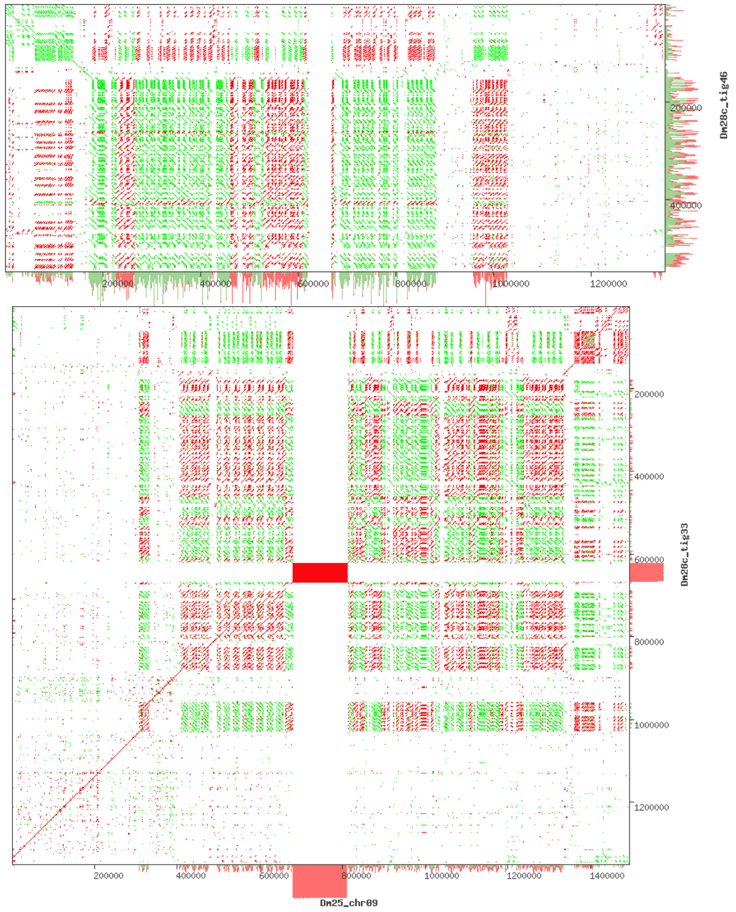

3. Dotplot analysis tig046l vs tig033l

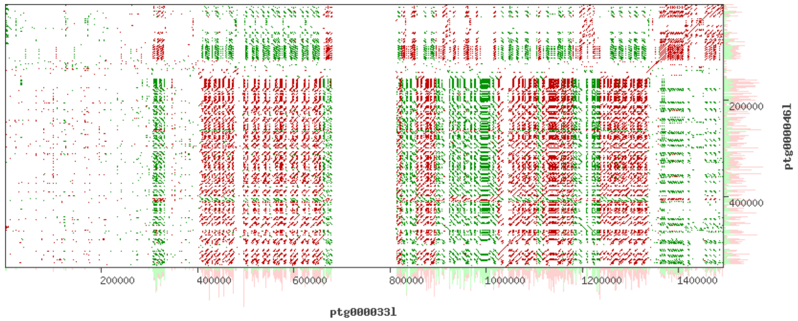

4. Dotplot analysis assembled chr3 vs chr09 Dm25

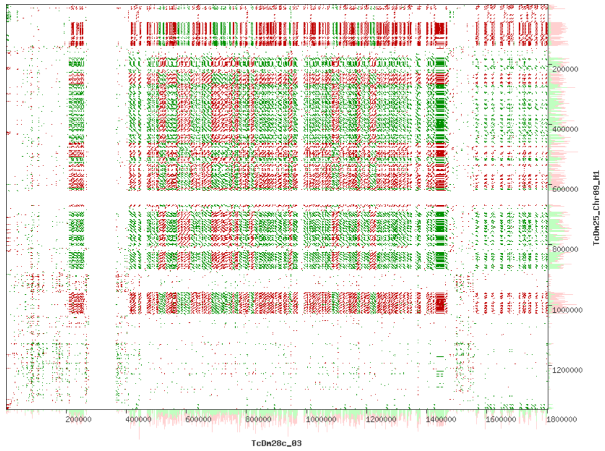

5. Validation using new 2025 Sylvio T2T

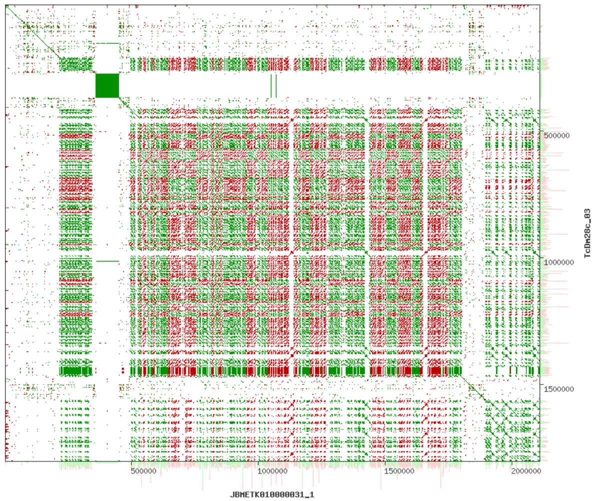

Manual assembly:

chr7= contig 015l (With 5’ Telomere, correspond to Dm25 chr05 T2T –missing only 3’ telomere región-) *3

1. Circos Dm25 vs all contigs Dm28c: tig015l with telomere 5’

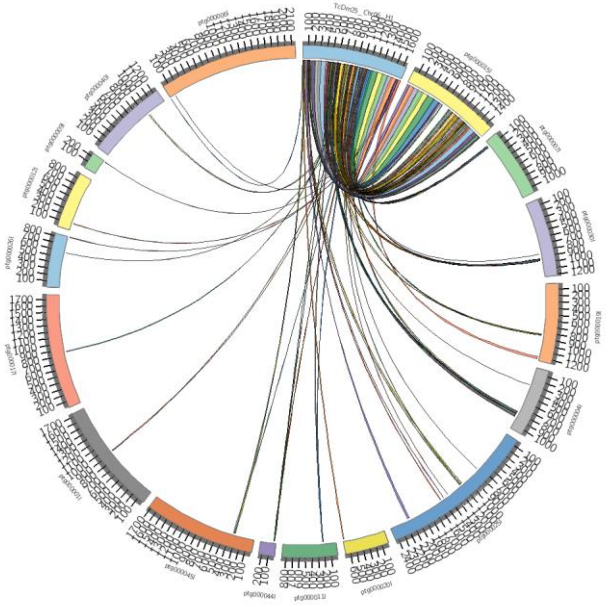

2. Dotplot analysis:
contigs Dm28c tig015 vs Chr06 Dm25

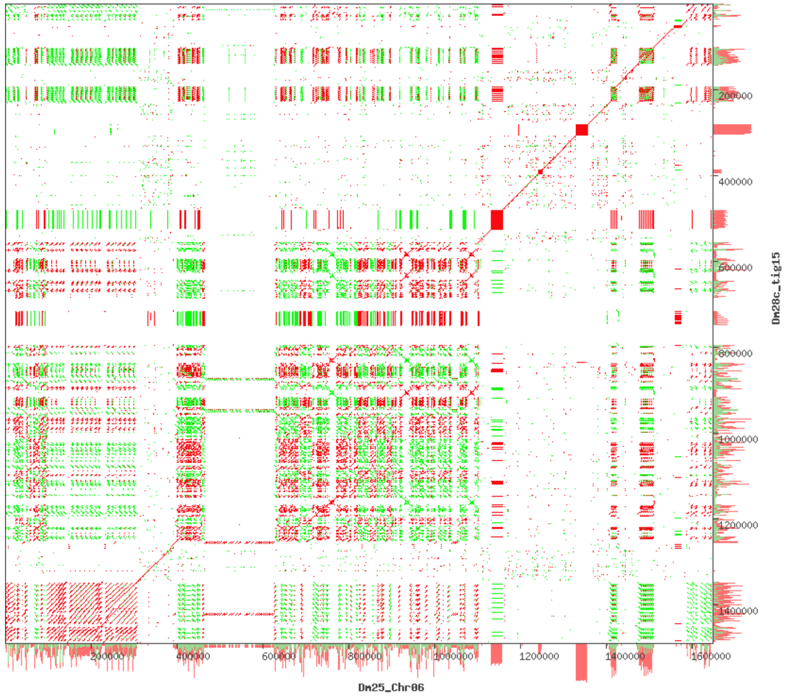

3. Validation using new 2025 Sylvio T2T (Reverse Complement)

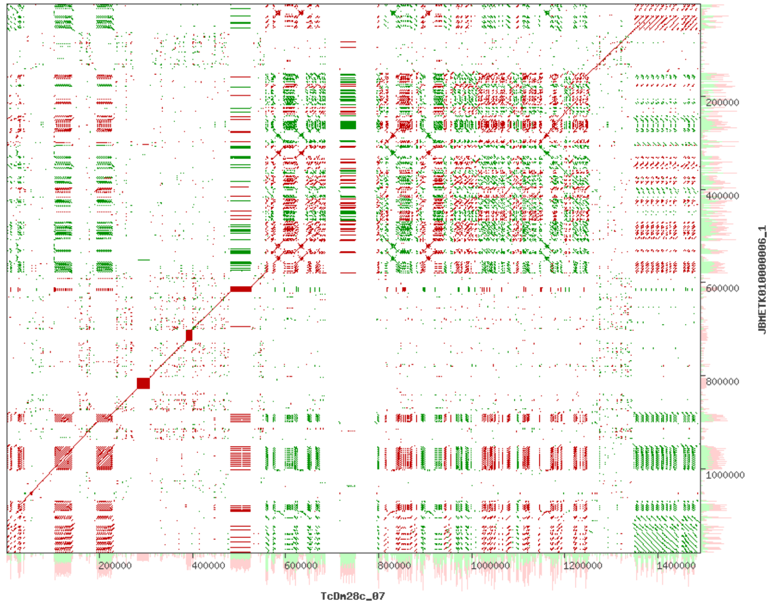

Manual assembly:

chr11= contig 43l (Dm25 chr08 T2T) *4

1. Circos Dm25 vs all contigs Dm28c: tig043l

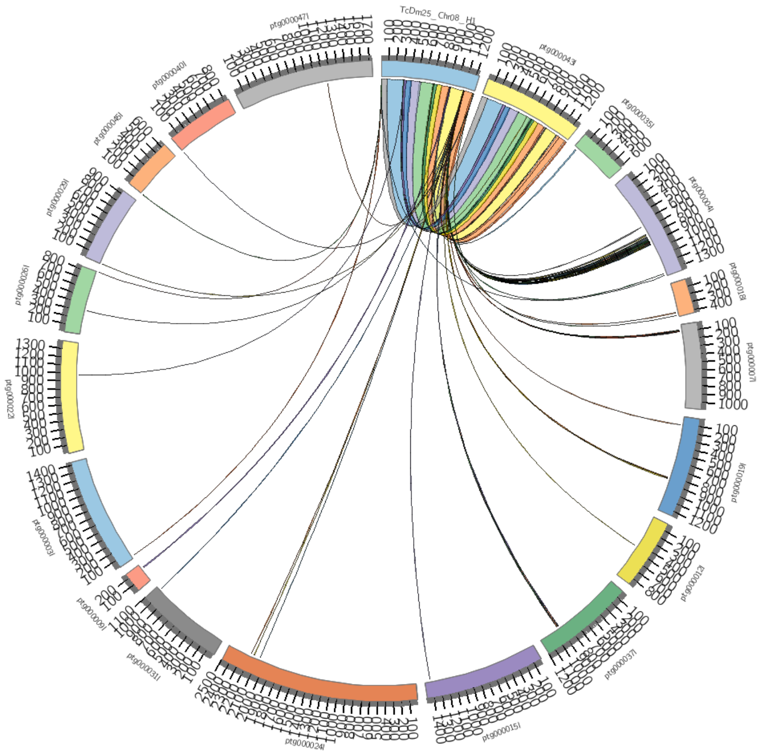

2. Dotplot analysis:
contigs Dm28c tig043 vs Chr08 Dm25

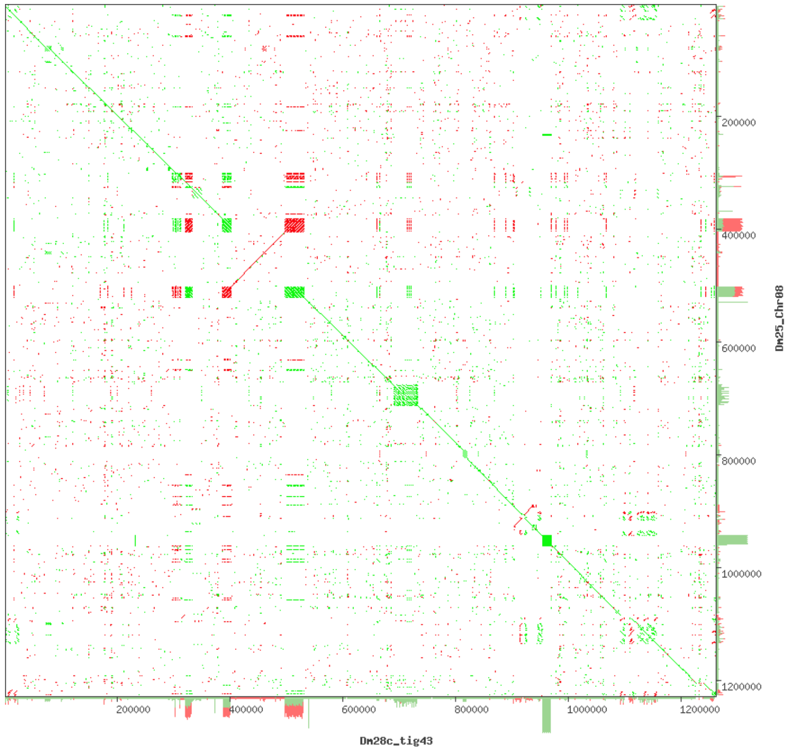

3. Validation using new 2025 Sylvio T2T

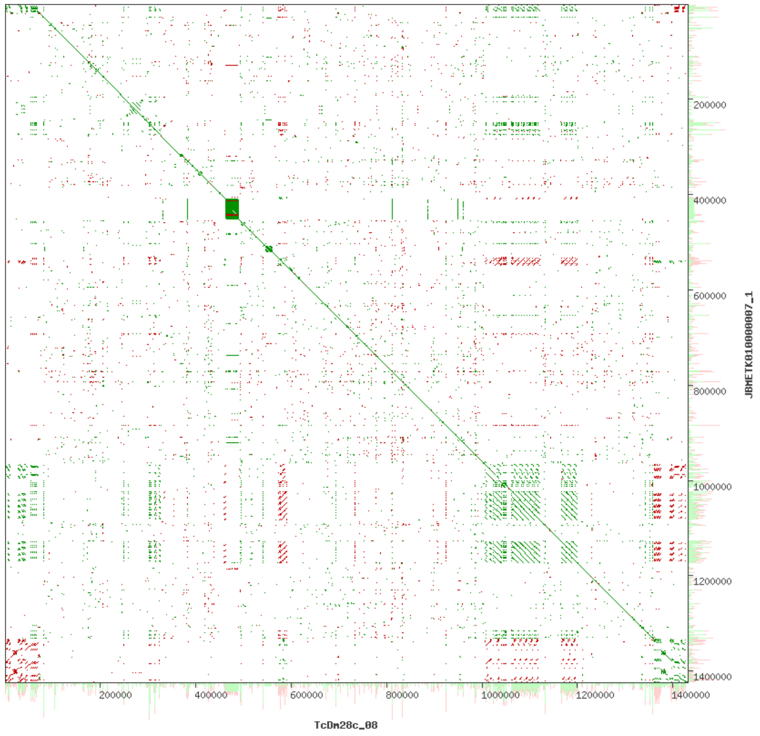

Manual assembly:

chr15= contig 31l (Dm25 chr10 T2T) *5

1. Circos Dm25 vs all contigs Dm28c: tig031l

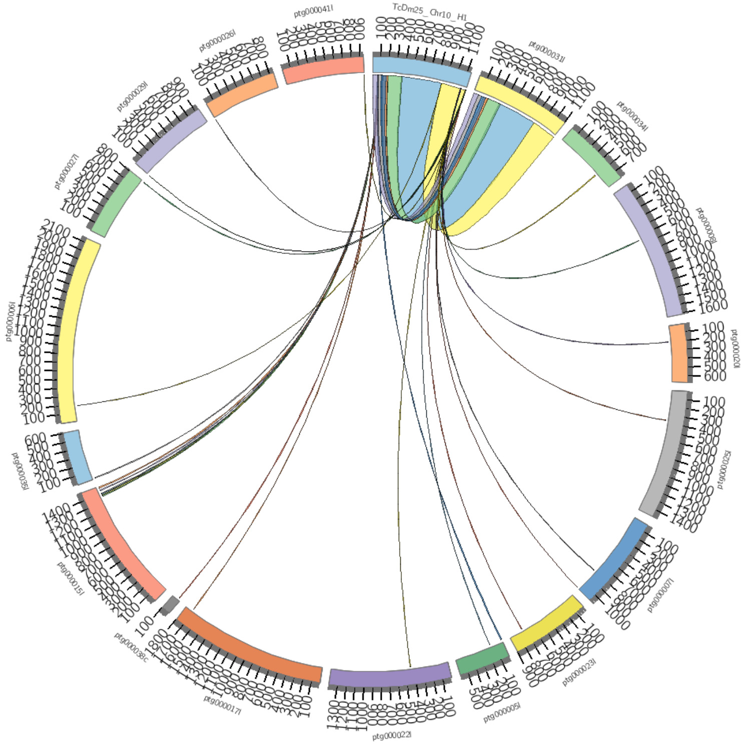

2. Dotplot analysis:
contigs Dm28c tig031 vs Chr10 Dm25

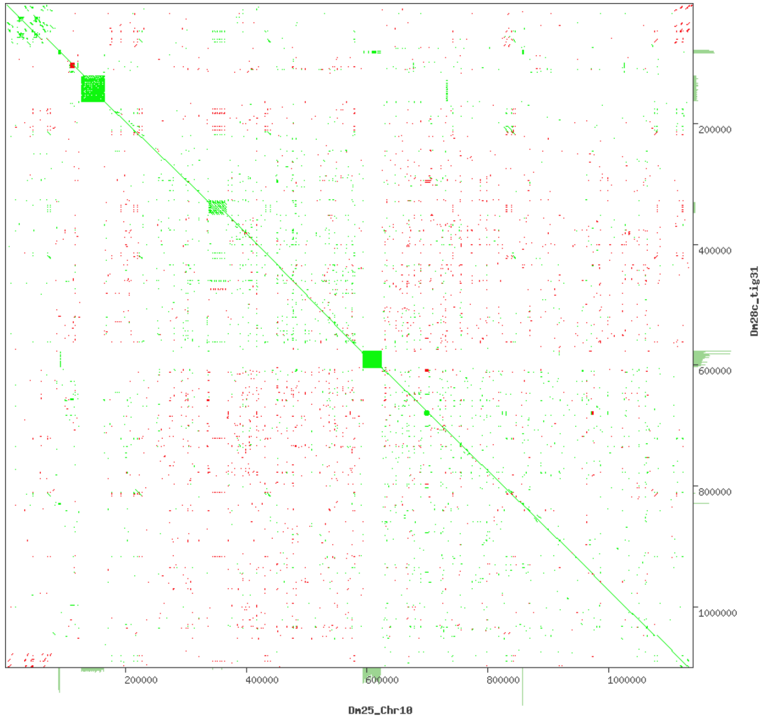

3. Validation using new 2025 Sylvio T2T

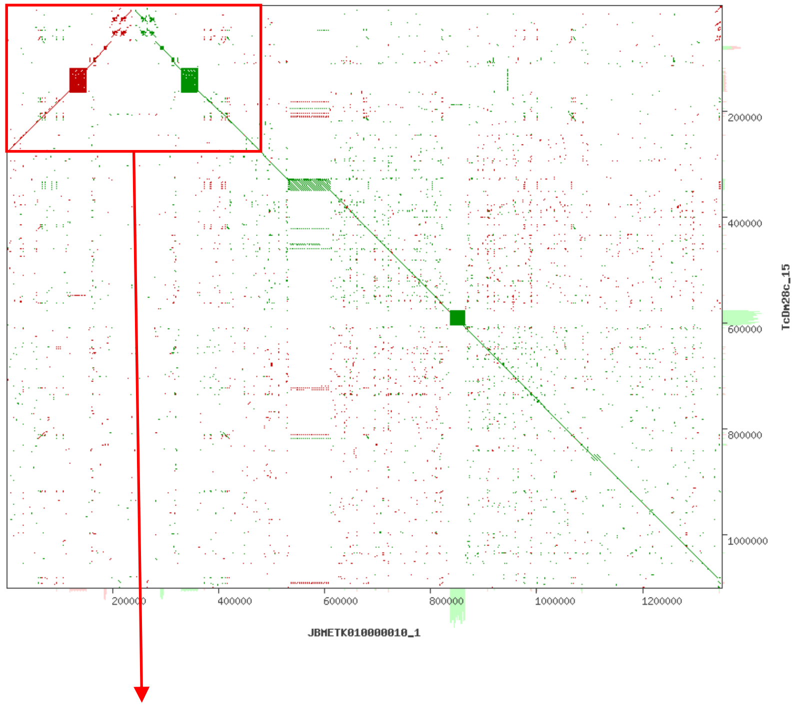

Posible error in Sylvio T2T assembly

Manual assembly:

chr18= join contig 11l + 42l (Dm25 chr14 T2T) *6

1. Circos Dm25 vs all contigs Dm28c: tig011l + tig42l

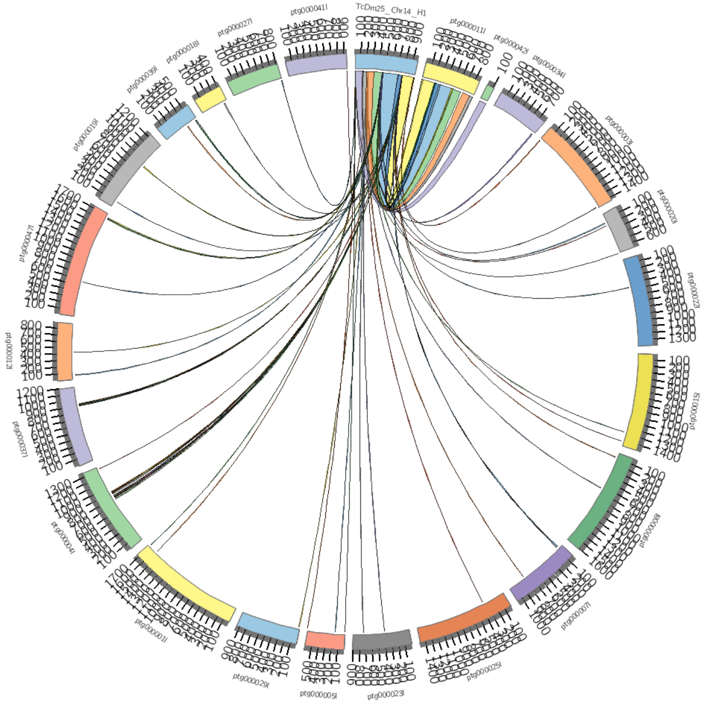

2. Dotplot analysis: contigs Dm28c tig011l and 42l vs Chr14 Dm25

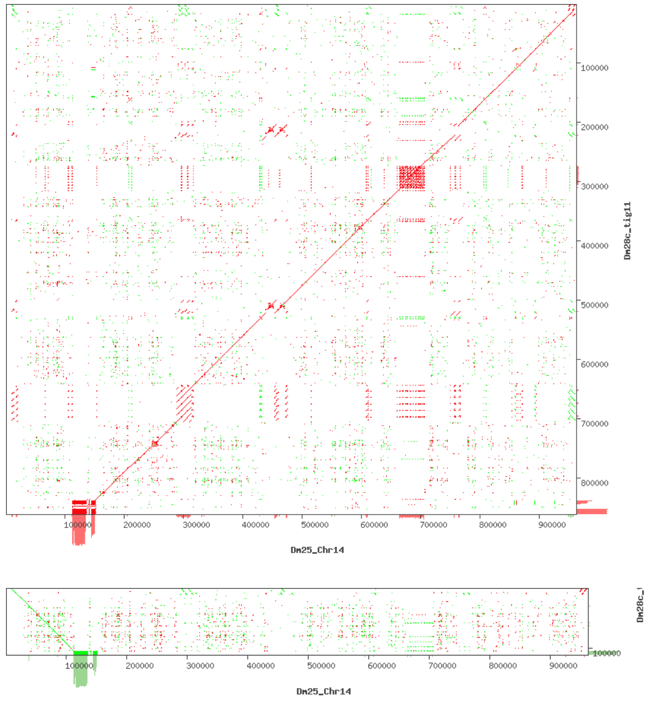

3. Dotplot analysis: Dm28c tig011l vs 42l (RC)

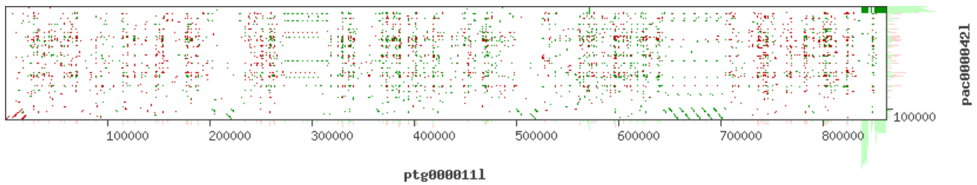

4. Validation using new 2025 Sylvio T2T

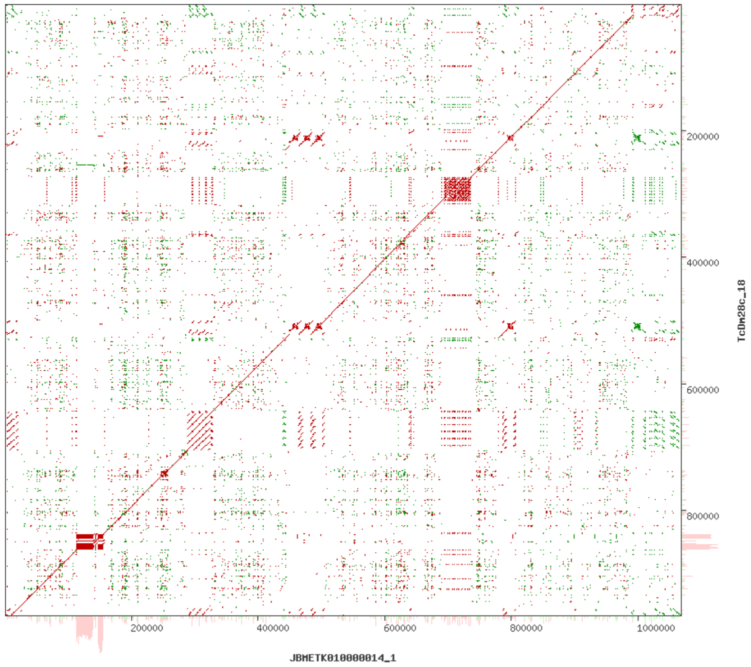

Manual assembly:

chr30 = contig 39l (Dm25 chr29 T2T) *7

1. Circos Dm25 vs all contigs Dm28c: tig047 (and haplotype tig24)

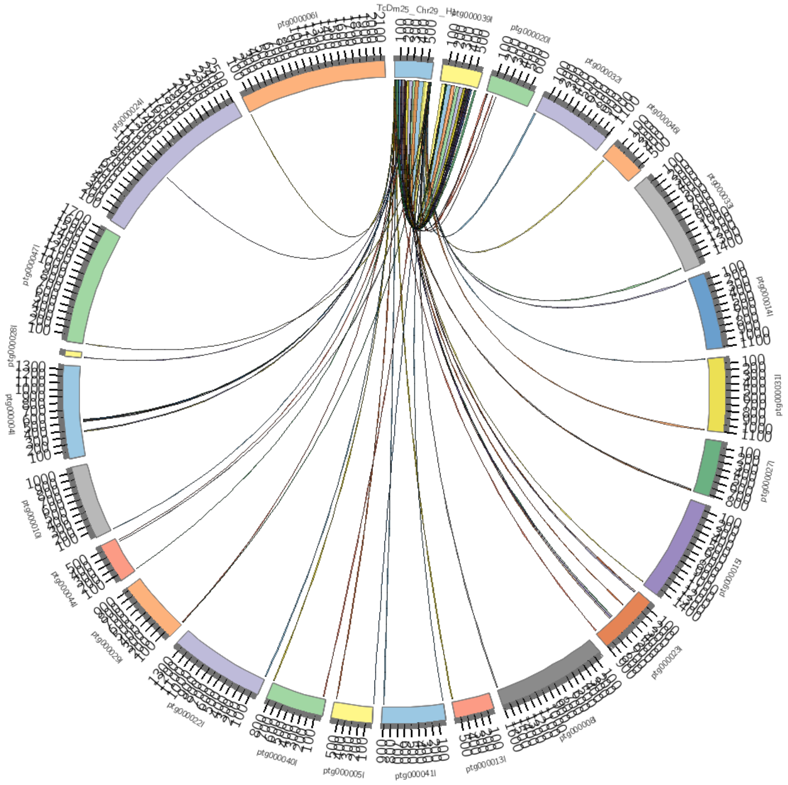

2. Dotplot analysis:
contig Dm28c tig039l vs Chr29 Dm25

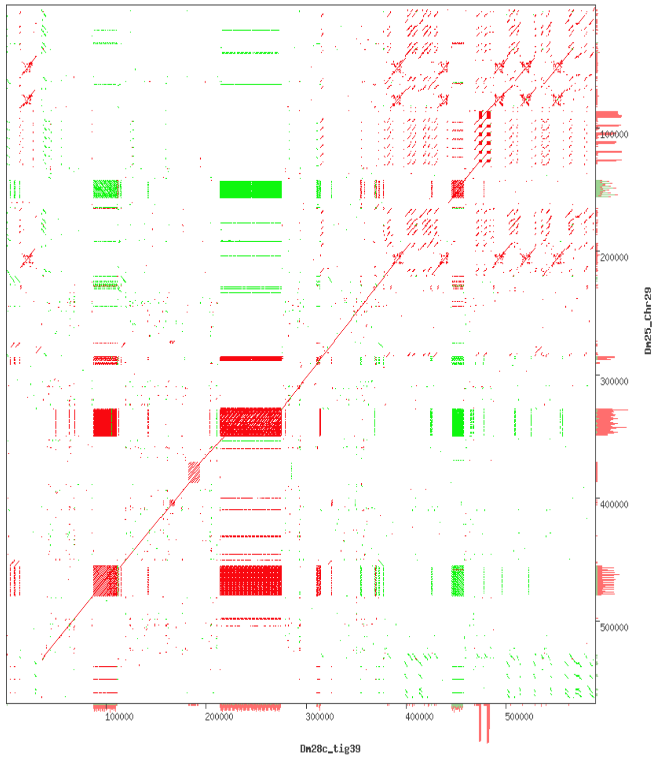

3. Validation using new 2025 Sylvio T2T

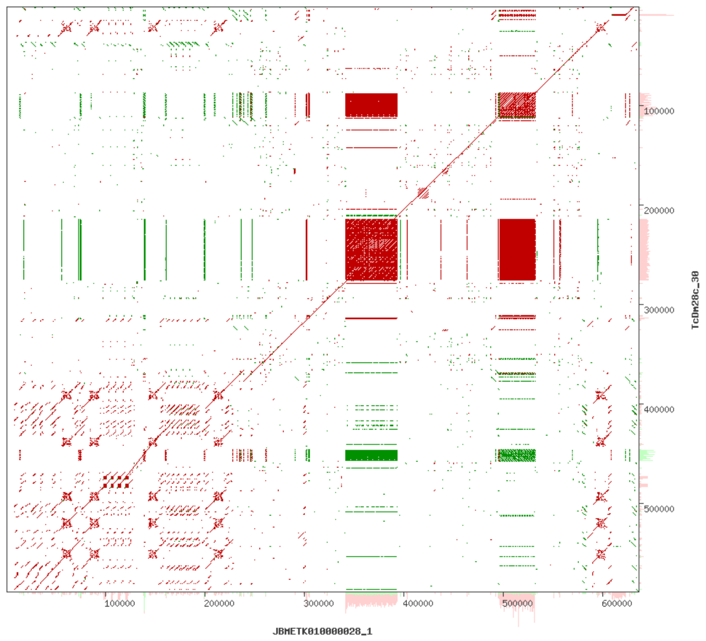

Supplement: Supplementary file 14 — Supplementary Material 14. [file 12864_2025_12482_MOESM14_ESM.pdf]
